# A combinatorial approach to improving the performance of azoarene photoswitches

**DOI:** 10.3762/bjoc.15.266

**Published:** 2019-11-14

**Authors:** Joaquin Calbo, Aditya R Thawani, Rosina S L Gibson, Andrew J P White, Matthew J Fuchter

**Affiliations:** 1 Department of Materials, Imperial College London, London SW7 2AZ, United Kingdom; 2 Department of Chemistry, Molecular Sciences Research Hub, White City Campus, Imperial College London, W12 0BZ, United Kingdom

**Keywords:** arylazopyrazoles, azobenzenes, molecular switches, *ortho*-substitution, photoswitches, thermal half-life

## Abstract

Azoarenes remain privileged photoswitches – molecules that can be interconverted between two states using light – enabling a huge range of light addressable multifunctional systems and materials. Two key innovations to improve the addressability and *Z*-isomer stability of the azoarenes have been *ortho*-substitution of the benzene ring(s) or replacement of one of the benzenes for a pyrazole (to give arylazopyrazole switches). Here we study the combination of such high-performance features within a single switch architecture. Through computational analysis and experimental measurements of representative examples, we demonstrate that *ortho*-benzene substitution of the arylazopyrazoles drastically increases the *Z*-isomer stability and allows further tuning of their addressability. This includes the discovery of new azopyrazoles with a *Z*-isomer thermal half-life of ≈46 years. Such results therefore define improved designs for high performance azo switches, which will allow for high precision optically addressable applications using such components.

## Introduction

Photoswitches are molecules that are capable of being reversibly interconverted between (at least) two states by means of light irradiation. The incorporation of photoswitches into multifunctional systems has huge relevance to next-generation materials, with a plethora of applications that range from photopharmacology [1–2] and optochemical genetics [3] to data storage [4]. Numerous classes of photochromic molecules exist, each with their own unique characteristics. For example, spiropyrans [5–6] may exhibit significant changes in solubility upon photoswitching, whilst the photoswitching of diarylethenes [7] is accompanied by large variations in their absorption spectra. However, azobenzenes remain one of the most popular photoswitches owing to their stability, reliability and tunability: azobenzenes provide high extinction coefficients and quantum yields, allowing switching between *Z*- and *E-*isomers with low-intensity light, and are stable to repeated switching cycles. Of the several performance metrics that can be used to judge azo switches, however, there are two predominant ones that can prove problematic for azoarenes: 1) the completeness of switching at a given wavelength of light, and 2) the thermal stability of the *Z-*isomer.

**Figure 1:**
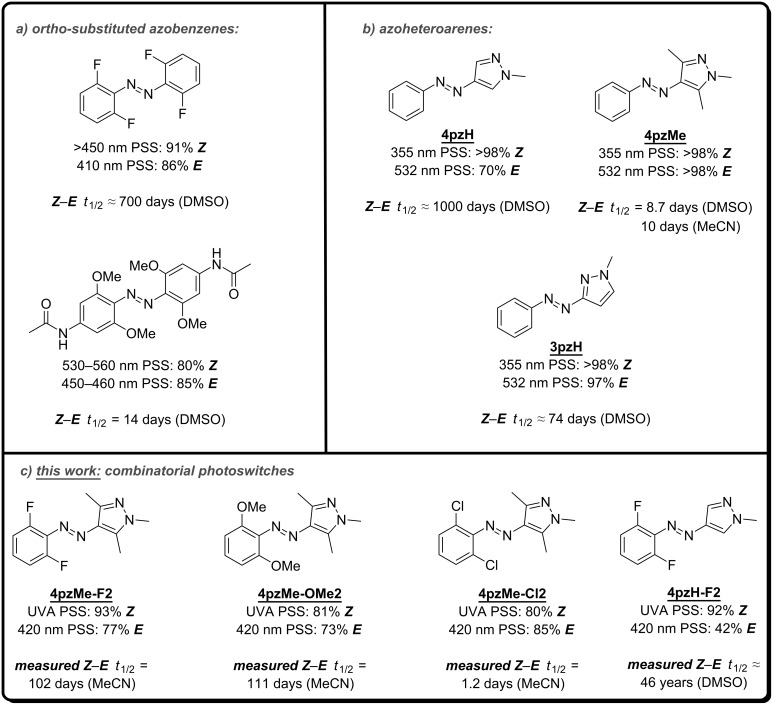
a) Tetra *ortho*-substituted azobenzenes represent a significant advance in terms of *Z*-isomer stability and completeness of photoswitching over azobenzene. b) Azoheteroarenes, developed by Fuchter and co-workers, offer quantitative, bidirectional photoswitching and long *Z*-isomer half-lives. c) This work seeks to understand the impact of these two approaches, when combined.

Despite the huge body of structure–property relationship studies known for substituted azobenzenes [8–9], it is still common to observe azo photoswitches that undergo incomplete photoswitching and/or possess low *Z*-isomer thermal stability. Perhaps the most important advancement to tackle these limitations in recent times has been the discovery that tetra-*ortho* substitution of the azobenzene unit can lead to a significant improvement of the photoswitching properties (Figure 1a). Specifically, *o*-methoxy [10–11] and *o*-thio [12] analogues reported by Woolley and co-workers demonstrate slow thermal *Z–E* relaxation and the potential to switch with red light, while the *o*-fluoro compounds reported by Hecht and co-workers [13–14] offer excellent two-way isomerization with visible light and the longest thermal half-life reported for an azobenzene molecule (≈700 days at 25 °C) to date.

An emerging alternative approach to tune the properties of azoarene photoswitches is to replace one or both of the benzene rings with a heteroaromatic ring [15–16]. While several useful heteroaromatic azo scaffolds have been reported, we previously identified such photoswitches containing a pyrazole ring, so-called arylazopyrazoles, which have excellent potential against the current state of the art (Figure 1b) [17–18]. Specifically, arylazopyrazole **4pzMe** can be near quantitatively (>98%) photoswitched in both directions, and **4pzH** demonstrated an exceptionally long thermal *Z*-isomer half-life (≈1000 days at 25 °C); one of the most stable azo photoswitches reported to date. We further extended the family of arylazopyrazoles with the help of theoretical modelling and discovered **3pzH** to be near quantitatively (>98%) switched back and forth between isomers, with a long thermal isomerization half-life (*t*_1/2_ = 74 days at 25 °C). The photochemical addressability of the azopyrazoles can further be complemented by other stimuli, for example chemical switching using acid [19]. Given their excellent performance, arylazopyrazoles are replacing azobenzenes in optically addressable applications including imaging applications [20], photopharmacology [21], supramolecular chemistry [22–23], responsive foams [24], coordination chemistry [25] and DNA nanotechnology [26–27].

Whilst the azopyrazoles have excellent properties for use in a variety of photo-addressable applications, it remains frustrating that in order to improve the photochemical addressability of the *Z*-isomer of these molecules (as is observed in **4pzMe** and **3pzH**) we needed to sacrifice thermal stability (**4pzH** vs **4pzMe** and **3pzH**), (Figure 1b) [17–18]. One parameter that was not explored in our previous structure–property relationship study, was substitution on the benzene ring [18]. Since our work, Venkataramani and co-workers [28] have explored how substitution on the benzene ring of azopyrazoles can impact their properties. However, in their study they focused on dimethylpyrazoles not methylated on the pyrazole ring N–H; compounds which have fast thermal *Z*–*E* conversion through mechanisms such as tautomerization. Furthermore, the study of Venkataramani and co-workers did not particularly focus on substitution patterns known to give high performance in azobenzene switches. We therefore considered whether specifically combining the two high performance designs of state-of-the-art azo photoswitches – *N*-methylated pyrazole plus an *o*-substituted benzene – would be advantageous for further improving the properties of the azopyrazoles (Figure 1c). It is important to note that Jan Ravoo and co-workers [23] have reported a bis-*ortho*-fluoroazopyrazole as part of their study of the supramolecular chemistry of these systems. Irradiating their compound at 365 nm enables near quantitative *E****→****Z* conversion, however, the 520 nm PSS provides 55% of the *E-*isomer. In water, this compound possesses a thermal half-life of >11 days. Herein, we present theoretical and representative experimental data concerning the performance for **4pzH** and **4pzMe** derivatives upon aryl mono- and di-*ortho*-substitution. Both computational and experimental measurements indicate the addition of *ortho*-substituents to the benzene ring of arylazopyrazoles has the potential to drastically increase the isomerization half-life (to months or years) and allow further tuning of the photoaddressability of each isomer. We believe that the structure–property relationships described will guide further development of azoheteroarene photoswitches (particularly arylazopyrazoles), and their use in a wide array of light-addressable applications.

## Results and Discussion

### The effect of *ortho*-substitution on half-life

We first assessed the theoretical half-lives for a series of compounds with *ortho*-substitution on the benzene ring of previously reported azopyrazoles **4pzH** and **4pzMe** (Table 1). Electron-poor (F and Cl) and electron-rich moieties (methoxy and pyrrolidine abbreviated as OMe and Pyr, respectively) were considered to analyze the effect of these *ortho*-groups on the thermal stability of *Z*-arylazopyrazoles (Table 1). Such substitution has proved useful in the improvement of azobenzene performance: *o*-F [13–14], *o*-Cl [8,29–30], *o*-OMe [10,29,31], or *o*-Pyr [32]. For the sake of comparison, both mono- and di-*ortho*-substitutions were considered.

**Table 1:** Theoretical half-lives (*t*_1/2, theor_ in hours and days) calculated at the PBE0-D3/6-31G** level of theory for *ortho*-substituted arylazopyrazoles **4pzH-X** and **4pzMe-X**. Experimental *t*_1/2, exp_ values are indicated.

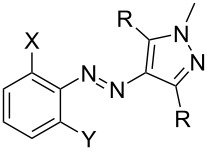						

R	X	Y	Compound	*t*_1/2, theor_ (h)	*t*_1/2, theor_ (d)	*t*_1/2, exp_

H	H	H	**4pzH**	**31000**	**1300**	**≈1000 d**^a,b^
H	H	F	**4pzH-F1**	55000	2300	
H	F	F	**4pzH-F2**	97000	4100	46 y^b^
H	H	Cl	**4pzH-Cl1**	2200	92	
H	Cl	Cl	**4pzH-Cl2**	120	5.1	
H	H	OMe	**4pzH-OMe1**	54000	2200	
H	OMe	OMe	**4pzH-OMe2**	350000	15000	
H	H	Pyr	**4pzH-Pyr1**	810000	34000	
H	Pyr	Pyr	**4pzH-Pyr2**	1800	76	

Me	H	H	**4pzMe**	**9.1**	**0.38**	**10 d**^a,c^
Me	H	F	**4pzMe-F1**	94	3.9	
Me	F	F	**4pzMe-F2**	620	26	102 d^c^
Me	H	Cl	**4pzMe-Cl1**	2.1	0.087	
Me	Cl	Cl	**4pzMe-Cl2**	0.025	0.0010	1.2 d^c^
Me	H	OMe	**4pzMe-OMe1**	160	6.5	
Me	OMe	OMe	**4pzMe-OMe2**	630	26	111 d^c^
Me	H	Pyr	**4pzMe-Pyr1**	200	8.3	
Me	Pyr	Pyr	**4pzMe-Pyr2**	380	16	

^a^Previously reported value [17]. ^b^Measured in DMSO-*d*_6_ at elevated temperatures and then extrapolated to 25 °C using an Eyring plot. ^c^Measured in MeCN-*d*_3_. d = days, y = years.

Theoretical half-lives (*t*_1/2_) were calculated within the density functional theory (DFT) framework, according to the protocol reported in our prior paper [18]. Briefly, the different possible pathways for thermal *Z–E* isomerization were calculated, and each process was weighted by the relative *Z*-isomer ground state energy and transition state (TS) energy barrier for all possible conformers (see Experimental for details). Focusing first on the **4pzH** scaffold, the theoretical calculations at the PBE0-D3/6-31G** level indicate that an insertion of electron-poor fluorine atoms in the *ortho*-position of the aryl ring (**4pzH-F1** and **4pzH-F2**) leads to an increase in the half-life from ca. 1000 days in **4pzH** to 2000 days in **4pzH-F1** and to 4000 days in **4pzH-F2** (Table 1). In contrast, chloro-substituted analogues present a significant decrease in the computed half-lives: 90 days for **4pzH-Cl1** and 5 days for **4pzH-Cl2**. The inclusion of methoxy and pyrrolidine groups in the *ortho*-position leads to an enhanced *Z*-isomer thermal stability compared to unsubstituted **4pzH**, with *t*_1/2_ = 2000 and 15000 days for **4pzH-OMe1** and **4pzH-OMe2**, respectively, and 34000 days for **4pzH-Pyr1** (Table 1). Unexpectedly, the inclusion of two bulky pyrrolidine groups in *ortho* (**4pzH-Pyr2**) leads to a decrease in *t*_1/2_ (76 days).

For the arylazopyrazole scaffold **4pzMe**, in line with the trend above, theoretical calculations indicate that the addition of *ortho*-fluoro atoms leads to higher *Z*-isomer stability, whereas the opposite effect is found upon chlorine *ortho*-substitution (Table 1). The insertion of one and two electron-donating OMe and Pyr groups systematically improve the *Z*-isomer stability compared to **4pzMe**, with *t*_1/2_ = 7 and 26 days for **4pzMe-OMe1** and **4pzMe-OMe2**, and *t*_1/2_ = 8 and 16 days for **4pzMe-Pyr1** and **4pzMe-Pyr2**, respectively.

**Figure 2:**
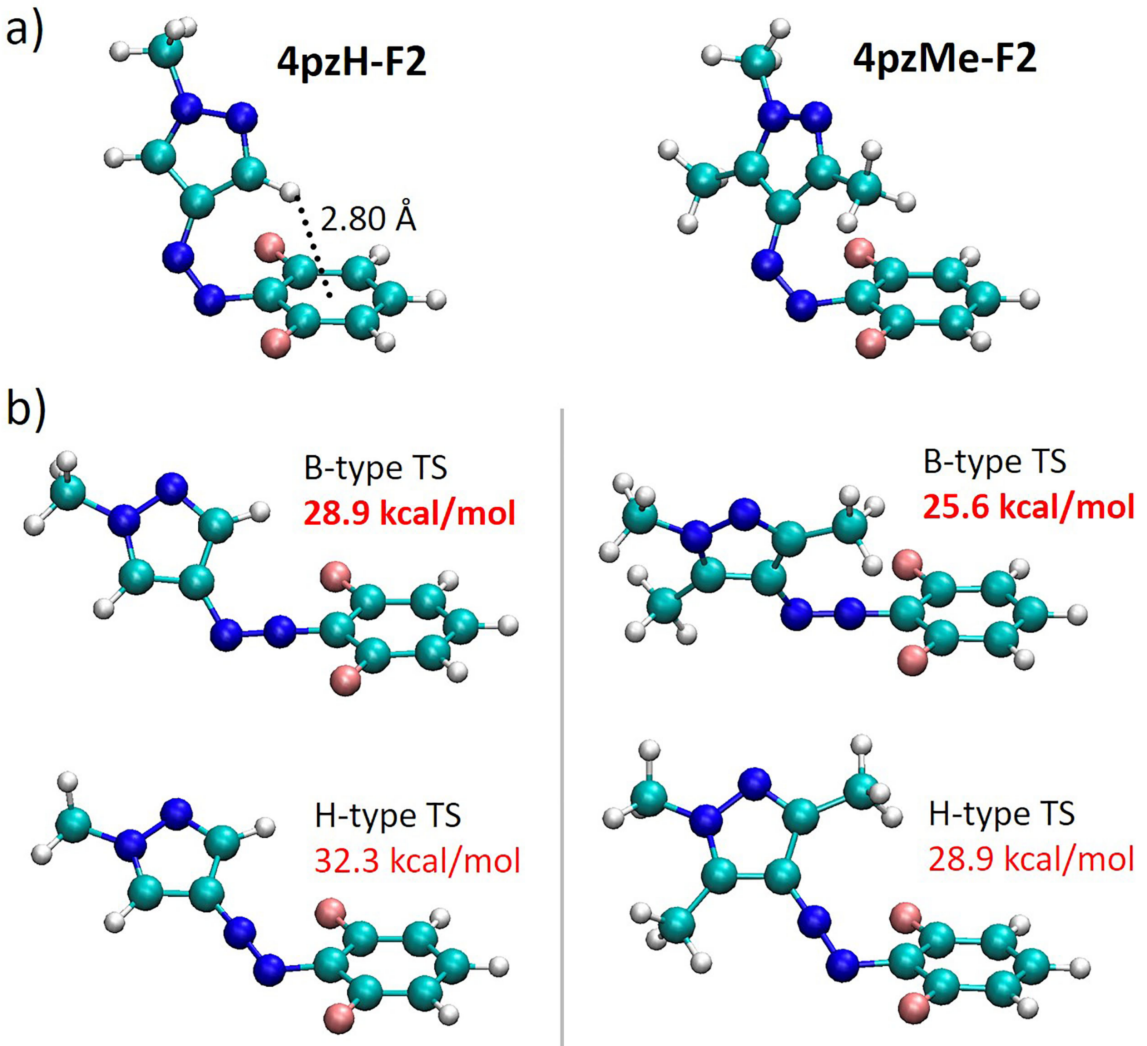
Minimum-energy geometry calculated for a) the *Z*-isomer ground state and b) the transition states with inversion of the N atom next to benzene (B-type) or the N atom next to heteroring (H-type) moiety, in arylazopyrazoles **4pzH-F2** (left) and **4pzMe-F2** (right). Free energy barriers calculated with respect to the corresponding ground-state *Z*-isomer are shown in red. Atom color coding: C in cyan, H in white, N in blue and F in pink.

It is important to note that, consistent with our previous results [18] and other recent reports [33], thermal *Z–E* isomerization for **4pzH-X** and **4pzMe-X** derivatives are predicted to occur through a transition state in which the N atom next to the benzene ring linearizes in an inversion mechanism (see B-type transition states in Figure 2 for **4pzH-F2** and **4pzMe-F2**, and Figure S1 in Supporting Information File 1 for the rest of di-*ortho*-substituted photoswitches). The inversion mechanism through linearization of the N atom next to the heteroring (H-type) is computed with a larger energy barrier in all cases (Figure 2 and Figure S1, Supporting Information File 1), and no low-energy rotation mechanism is predicted, in agreement with other reports [34]. Also consistent with our previous studies on **4pzH** compared to **4pzMe** [18], significantly larger half-lives are predicted for **4pzH-X** derivatives compared to the dimethylated **4pzMe-X** derivatives, which can be rationalized by the stabilizing short CH···π interactions in the T-shaped conformation of *Z*-**4pzH-X** (Figure 2).

In order to confirm our predictions of the *ortho*-substitution effect on the thermal *Z–E* isomerization process, photoswitches **4pzMe-F2**, **4pzMe-Cl2**, **4pzMe-OMe2** and **4pzH-F2** were synthesized (see Supporting Information File 1). Following irradiation to the PSS (see further details below), the thermal *Z*–*E* conversion was monitored by ^1^H NMR spectroscopy (Table 1). While the results do not quantitatively match the theoretical predictions (as found previously [18]), the trends are faithfully reproduced: **4pzMe-F2** (*t*_1/2_ = 102 days), and **4pzMe-OMe2** (*t*_1/2_ = 111 days) have increased thermal stability over **4pzMe** (*t*_1/2_ = 10 days), whereas **4pzMe-Cl2** (*t*_1/2_ ≈ 1 day) exhibits a reduction in half-life. Additionally, **4pzH-F2** boasts a vastly increased thermal half-life of 46 years over **4pzH** (*t*_1/2_ = 1000 days) making it the longest-lived azoheteroarene photoswitch reported in the literature to date. This result demonstrates that it is indeed possible to combinatorially integrate two high performing azo switch fragments to discover switches with very long thermal half-lives (i.e., months to years).

**Figure 3:**
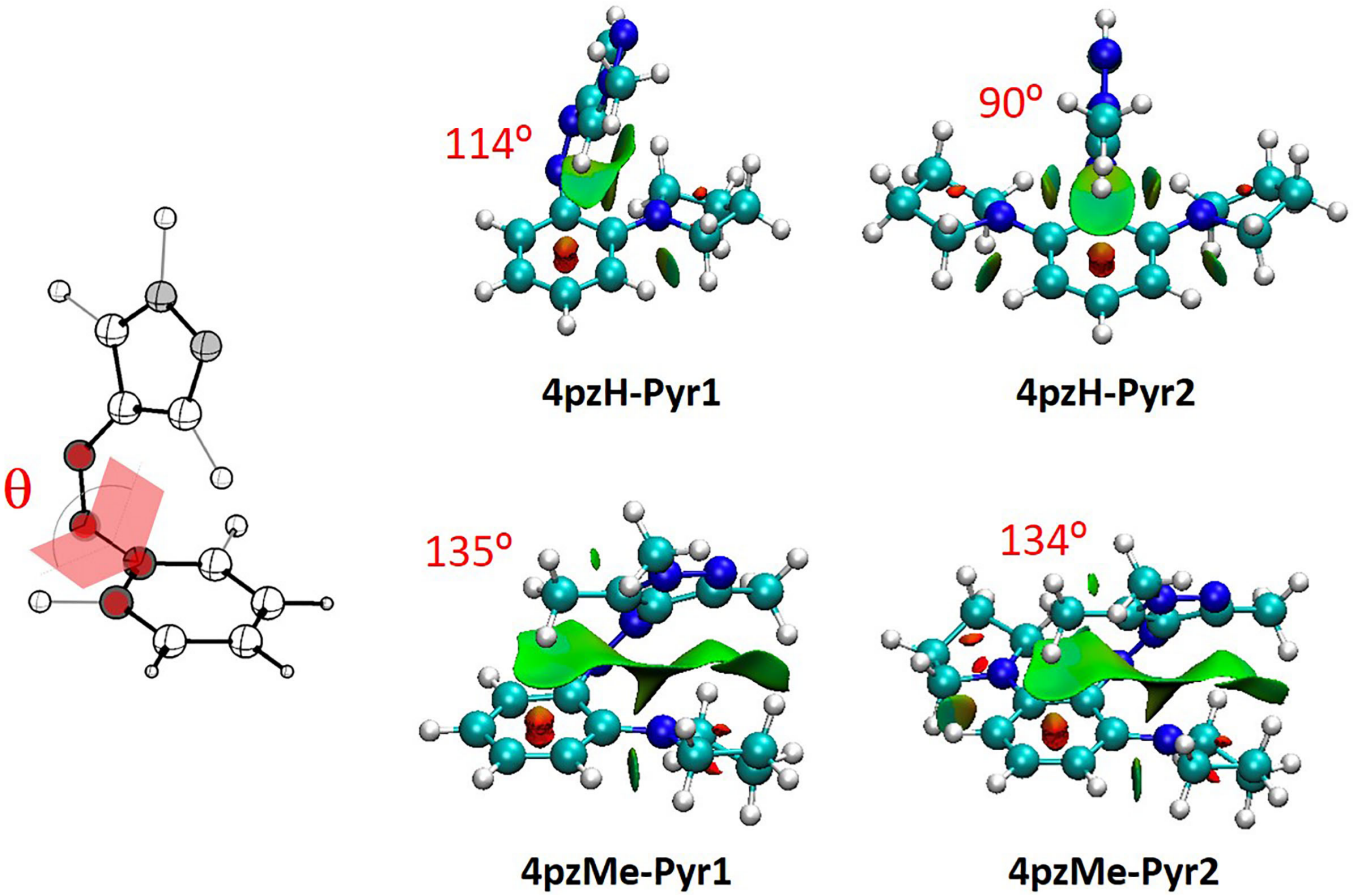
Noncovalent index (NCI) surfaces calculated for representative pyrrolidine-based *ortho*-substituted arylazopyrazoles. The definition of the CCNN dihedral angle between the aryl group and the heteroring moiety (θ) is shown, and the corresponding values for the derivatives are displayed in red.

We sought to further explain the trends observed through theoretical analysis of the minimum-energy geometries and noncovalent interactions. The energy-minimized structures for the *Z*-isomer of representative photoswitches are shown in Figure S2 (Supporting Information File 1). The proximity of the *ortho*-benzene substituents to the heteroaryl ring in the *Z*-isomer either stabilizes the *Z*-isomer ground state via noncovalent interactions, or destabilizes it, via steric clashes. In contrast, these effects are significantly diminished for the transition state geometry (Figure S3 in Supporting Information File 1), at which the pyrazole moiety and the *ortho* groups remain far apart. A particularly interesting case in point are the **4pzH-Pyr** compounds: the inclusion of one pyrrolidine group in the *ortho*-position (**4pzH-Pyr1**) is predicted to lead to a massively enhanced *Z*-isomer thermal stability (≈92 years), whereas two bulky pyrrolidine groups (**4pzH-Pyr2**) decrease stability, with a comparably modest *t*_1/2_ = 76 d (Table 1). Theoretical calculations of the noncovalent index (NCI) surfaces indicate that dispersion forces exist between pyrrolidine groups and the heteroaromatic ring (green surfaces in Figure 3). The inclusion of one pyrrolidine (**4pzH-Pyr1**) promotes weak but stabilizing noncovalent interactions between the *ortho* group and the pyrazole moiety, causing a tilting of the heteroring away from orthogonality with respect to the benzene plane (from θ = 90° in **4pzH** to 114° in **4pzH-Pyr1**), and therefore supporting the long theoretical half-life through *Z*-ground state stabilization (Table 1). However, for the highly congested doubly substituted **4pzH-Pyr2**, a perfect T-shape conformation is predicted as the minimum-energy structure (θ = 90°; Figure 3). The steric interaction between the second pyrrolidine group with the azo group (CH···azo-N distance of only 2.2 Å) prevents the favorable tilting observed for **4pzH-Pyr1**. For the ***Z*-4pzMe-X** analogues (Figure 3), their twisted arrangements allow for a large array of weak and stabilizing noncovalent interactions between the *ortho*-groups and the methyl-substituted pyrazole ring (θ ca. 135°), potentially explaining the increase in *t*_1/2_ upon systematic introduction of pyrrolidine moieties (Table 1).

**Figure 4:**
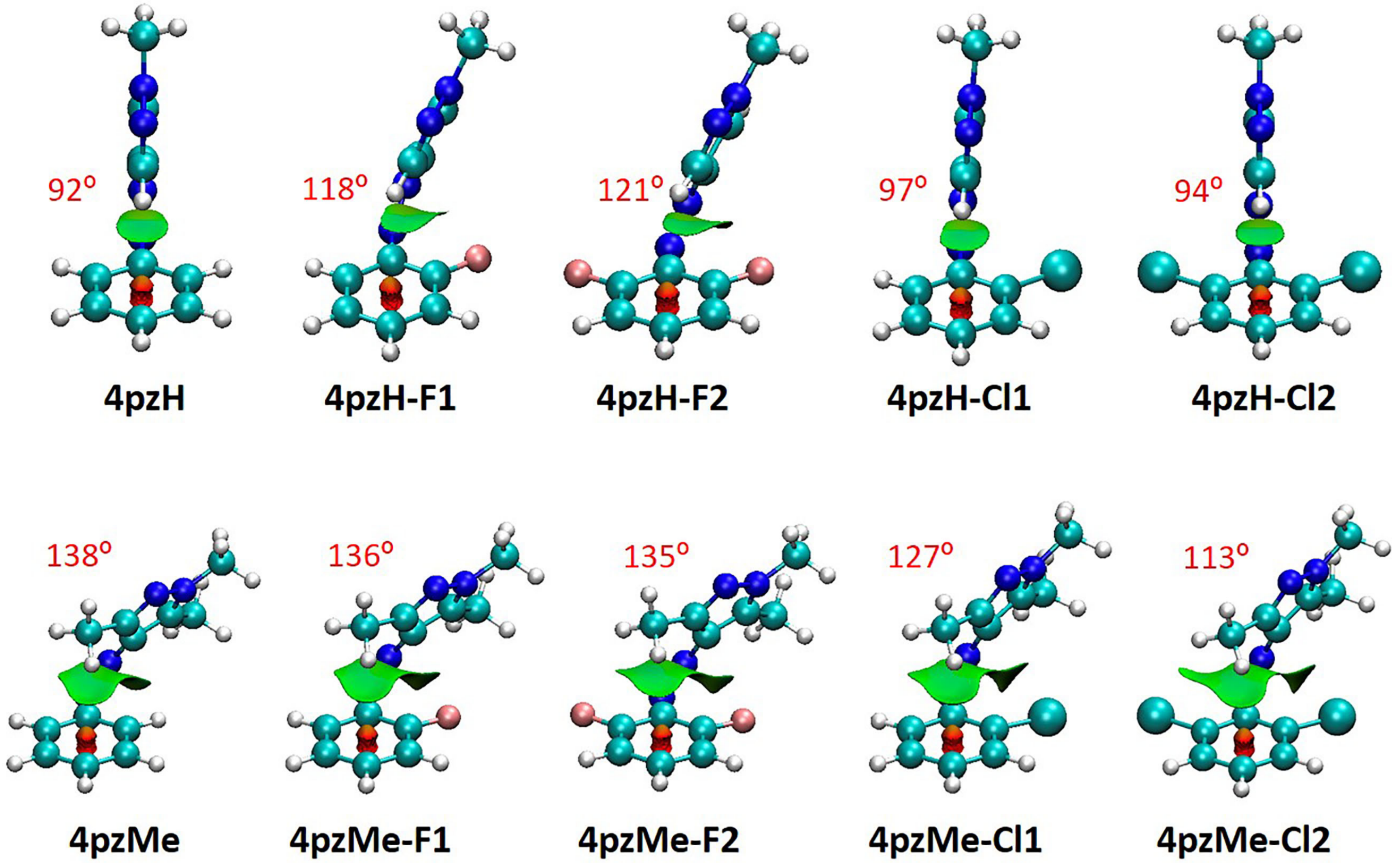
Noncovalent index (NCI) surfaces and θ dihedral angles (in red) calculated for the minimum-energy geometry of *ortho*-halogenated *Z*-arylazopyrazoles.

The *ortho*-halogen-substituted photoswitches also present an unexpected behavior, where very large differences in half-lives are predicted depending on the nature of the halogen atom (from 5 days in **4pzH-Cl2** to ca. 4000 days in **4pzH-F2**, or from 2 minutes in **4pzMe-Cl2** to 26 days in **4pzMe-F2**, Table 1). The analysis of the NCI surfaces indicates that inclusion of F atoms in the *ortho*-position promotes stabilizing dispersion interactions with the pyrazole ring, provoking a tilting of the heteroring from 92° in **4pzH** to 118° in **4pzH-F1** and to 121° in **4pzH-F2** (Figure 4). Stabilizing F···pyrazole noncovalent forces are also predicted in the twisted *Z-*isomers of the **4pzMe-X** family, for which θ remains approximately constant. Furthermore, the atomic charge of fluorine is calculated to be ca. −0.30*e*, which allows for electrostatic interactions with positively charged C atoms (ca. +0.40*e*) of the pyrazole ring in both **4pzH-X** and **4pzMe-X** families (Figure S7, Supporting Information File 1). These favorable dispersion and electrostatic interactions stabilize the *Z*-isomer ground state compared to the corresponding transition state, which in part explains the increase in energy barriers and in turn half-lives upon *ortho*-F substitution. In sharp contrast, the insertion of bulkier chlorine atoms at the *ortho* position(s) induces destabilizing steric clashes, as well as negligible electrostatic forces (chlorine atomic charge of +0.04*e*; Figure S8 in Supporting Information File 1). Somewhat similar to the **4pzH-Pyr** compounds, the destabilizing nature of the Cl···pyrazole interaction is evidenced by the absence of heteroring tilting in the T-shaped *Z*-isomers of the **4pzH-X** family (θ = 97° and 94° for **4pzH-Cl1** and **4pzH-Cl2**), and the systematic decrease in θ for the **4pzMe-X** series in going from **4pzMe** (138°) to **4pzMe-Cl1** (127°) and to **4pzMe-Cl2** (113°; Figure 4).

### The effect of *ortho*-substitution on photoconversion efficiency

To a first approximation, in order to achieve high *Z–E* photoconversion efficiency, the two isomers of an azo photoswitch must offer well-separated absorption bands. Most frequently, *E*–*Z* photoisomerization is achieved by irradiating in the region of the high-energy π–π* band for the *E*-isomer, whereas *Z–E* photoisomerization occurs through irradiation in the low-energy *n*–π* band of the *Z*-isomer. However, an overlap in the absorbances between *E*/*Z* isomers causes incomplete photoswitching.

**Figure 5:**
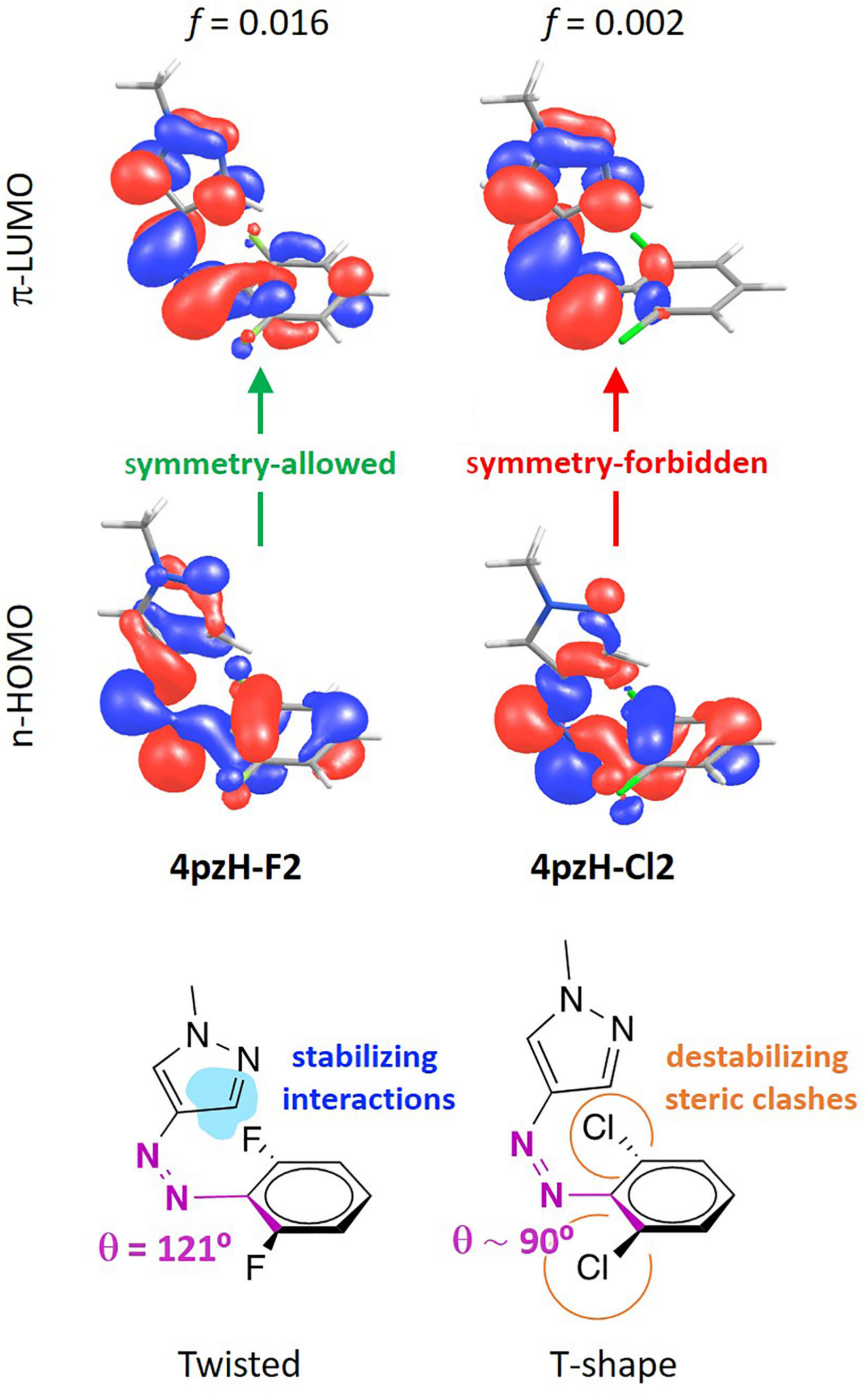
Description of the lowest-lying *n*–π* excitation for the *Z-*isomers of halogenated **4pzH-F2** and **4pzH-Cl2** photoswitches.

Table 2 summarizes the theoretical excitation energy separation (in nm) between the characteristic *n–*π* and π–π* transitions in the *ortho*-substituted arylazopyrazoles under study. Theoretical TD-DFT calculations indicate that the family of *ortho*-substituted **4pzH-X** presents well-separated *n–*π* transitions between *Z* and *E* (>30 nm), with the exception of pyrrolidine derivatives (Table 2). As such, the addition of *ortho*-substitution may provide a means to improve the addressability of the longer half-live **4pzH** compounds. In contrast, most of the **4pzMe-X** analogues show a relatively small band separation of the *n–*π* transition between the *Z*- and *E*-isomers, for which a less efficient *Z*-to-*E* photoconversion is expected. Note that the lowest-lying *n–*π* transition in the *Z*-isomer originates from a HOMO to LUMO monoelectronic excitation (Figure 5). The energy gap between these two molecular orbitals highly depends on the molecular conformation, significantly increasing for T-shaped structures (**4pzH-X**) compared to twisted arrangements (**4pzMe-X**), and thus leading to larger *n–*π* band separation for the **4pzH-X** series [18]. On the other hand, the intense π–π* transition of the *Z*-isomer is generally found higher in energy and lower in intensity compared to the *E*-isomer (Tables S1 and S2 in Supporting Information File 1), which is common for azo switches. For the **4pzH-X** family, large π–π* band separations of >50 nm are predicted between *Z* and *E*, whereas smaller values (>30 nm) are calculated for **4pzMe-X** (Table 2). In both series, di-*ortho*-chlorination is predicted to provide the higher overlap in π–π* bands between *Z* and *E*, for which a less efficient *E*-to-*Z* photoconversion is therefore expected.

**Table 2:** Theoretical energy separation (in nm) and oscillator strength (*f*, in au) of the characteristic electronic excitations in *ortho*-substituted arylazopyrazoles **4pzH-X** and **4pzMe-X**.^a^

	*n*–π* (*E*–*Z*)	π–π* (*E*–*Z*)	*n–*π* *f* (*Z*)	*n*–π* *f* (*E*)

**4pzH**	38	58	0.0020	0.0000
**4pzH-F1**	33	64	0.0129	0.0000
**4pzH-F2**	44	58	0.0157	0.0000
**4pzH-Cl1**	51	62	0.0024	0.0074
**4pzH-Cl2**	46	43	0.0018	0.0129
**4pzH-OMe1**	35	81	0.0110	0.0000
**4pzH-OMe2**	46	66	0.0069	0.0204
**4pzH-Pyr1**	−26	118	0.0309	0.0290
**4pzH-Pyr2**	−10	81	0.0038	0.0575

**4pzMe**	−15	30	0.0404	0.0000
**4pzMe-F1**	4	34	0.0420	0.0008
**4pzMe-F2**	26	33	0.0426	0.0025
**4pzMe-Cl1**	5	35	0.0371	0.0085
**4pzMe-Cl2**	5	23	0.0314	0.0147
**4pzMe-OMe1**	12	53	0.0404	0.0000
**4pzMe-OMe2**	29	47	0.0371	0.0124
**4pzMe-Pyr1**	−34	94	0.0557	0.0728
**4pzMe-Pyr2**	−36	59	0.0407	0.0865

^a^Excitation energies and intensities are averaged over the different conformers (see Tables S1 and S2 in Supporting Information File 1).

In addition to the band separation, the *n*–π* transition is required to be relatively intense for the *Z-*isomer compared to *E* to allow for efficient *Z–E* photoswitching. Theoretical calculations indicate that the family of *ortho*-substituted **4pzH-X** photoswitches provide a weak *n*–π* transition, with oscillator strengths *f* < 0.020. In contrast, **4pzMe-X** derivatives show a relatively intense *n*–π* excitation, with *f* > 0.035. The intensity of the *n*–π* transition is directly related with the dihedral angle between the benzene moiety and the heteroring (Figure S9, Supporting Information File 1), as we have shown previously [18]. The *n*–π* excitation is symmetry-forbidden for a complete (θ = 90°) T-shape conformation. Thus, a tilting of the pyrazole moiety away from orthogonality with respect to the plane generated by the aryl ring leads the *n*–π* excitation to be symmetry-allowed, with the corresponding increase in intensity. For example, the heteroring in fluorine-substituted **4pzH-F2** photoswitch is tilted with a θ = 121°, and presents a moderately intense *n*–π* transition (*f* = 0.016), whereas the *n*–π* intensity in T-shaped *Z*-**4pzH-Cl2** is practically zero (Figure 5). On the other hand, methyl-based **4pzMe-X** derivatives show a twisted-like conformation for the minimum-energy geometry of the *Z*-isomer (Figure S2, Supporting Information File 1), promoting intense, symmetry-allowed *n–*π* excitations (Table 2).

**Figure 6:**
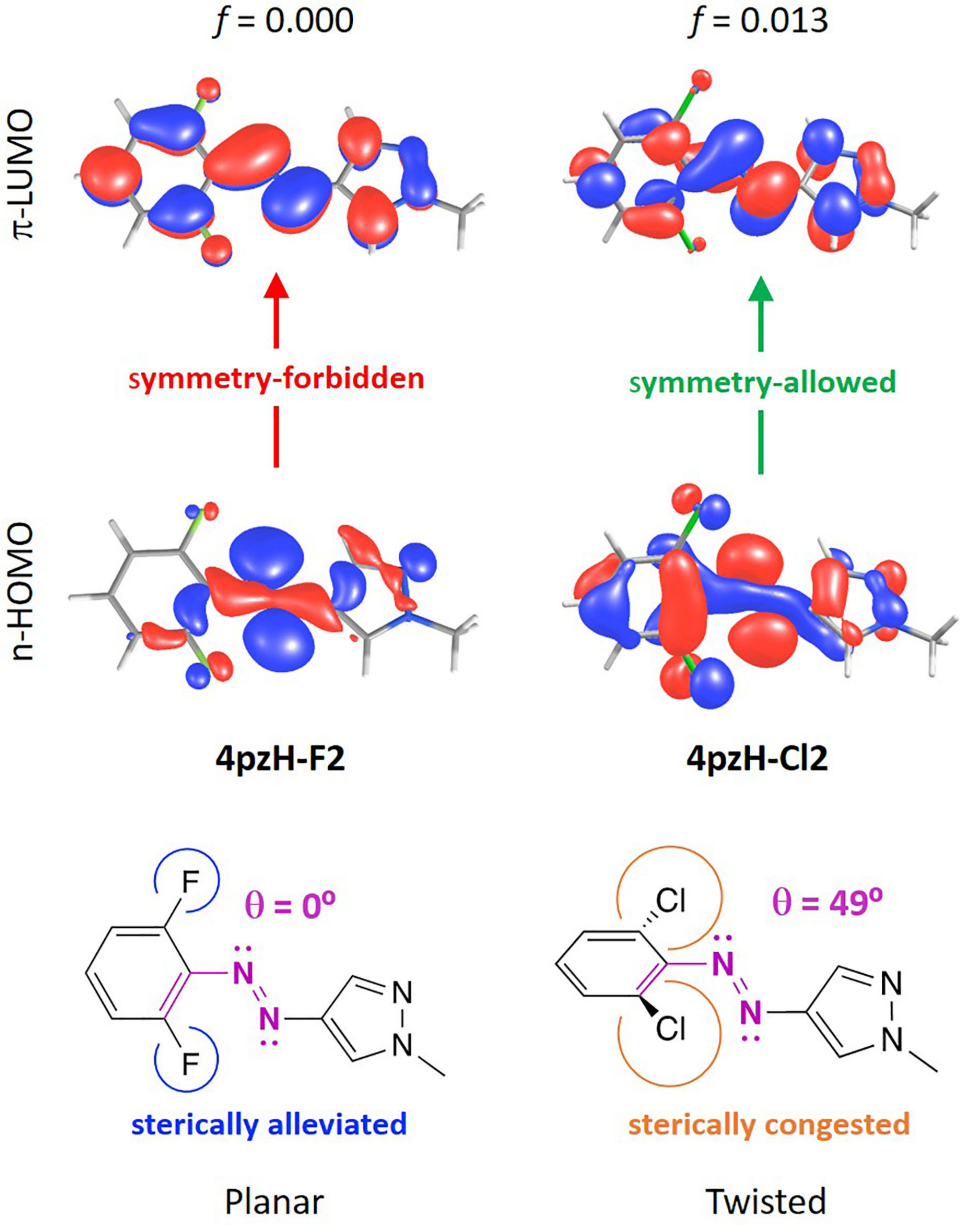
Description of the lowest-lying *n*–π* excitation for the *E-*isomers of halogenated **4pzH-F2** and **4pzH-Cl2** photoswitches.

The *n–*π* excitation in *E-*isomers is also symmetry-forbidden for a completely planar conformation in which the heteroring, the azo group and the benzene moiety are coplanar. Bulky groups introduced in the *ortho-*position of the benzene ring, such as methoxy, pyrrolidine or chlorine, promote steric interactions that disrupt planarity, with the corresponding increase in the *n–*π* transition intensity for the *E*-isomer of these compounds (*f* = 0.013, 0.020 and 0.058 for **4pzH-Cl2**, **4pzH-OMe2** and **4pzH-Pyr2**, and *f* = 0.015, 0.012 and 0.087 for **4pzMe-Cl2**, **4pzMe-OMe2** and **4pzMe-Pyr2**, respectively; see Figure 6 for **4pzH-F2** and **4pzH-Cl2**).

**Figure 7:**
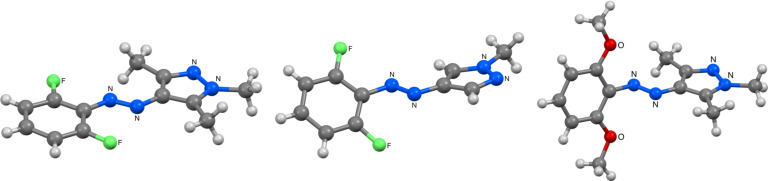
X-ray structures of **4pzMe-F2** (left), **4pzH-F2** (middle) and **4pzMe-OMe2** (right).

To experimentally compare the conformations of the *E-*isomers, we were able to characterize **4pzMe-F2**, **4pzMe-Cl2** and **4pzH-F2** by X-ray crystallography (Figure 7). In agreement with the calculations, the *E*-isomer of **4pzMe-F2** possesses a fully coplanar structure. Unexpectedly, for **4pzMe-OMe2** the pyrazole and benzene rings occupy two separate planes. Thus, in the solid state, the structure of the *E*-isomer **4pzMe-OMe2** becomes even more twisted (calculated dihedral angle of 49° versus 78° in the crystal structure). We attribute this to a packing effect in the solid state. To the best of our knowledge, an *E-*isomer azo photoswitch with this type of solid state packing is unprecedented and may open new possibilities in solid state photoswitchable materials applications. The benzene ring in **4pzH-F2** is also twisted away from the pyrazole ring with a calculated dihedral angle of approximately 42°. This is reminiscent of the *o-*methoxyazobenzenes, reported by Woolley and co-workers [10–11], where steric repulsion forces the *E*-isomer into a nonplanar mode. However, given the small size of the fluorine atoms, sterics are unlikely to play a role for **4pzH-F2** and we would expect a planar *E*-isomer, as noted by Hecht and co-workers in their *o*‑fluoroazobenzenes [13–14]. Thus, once again, solid state packing of these derivatives is surprising.

**Figure 8:**
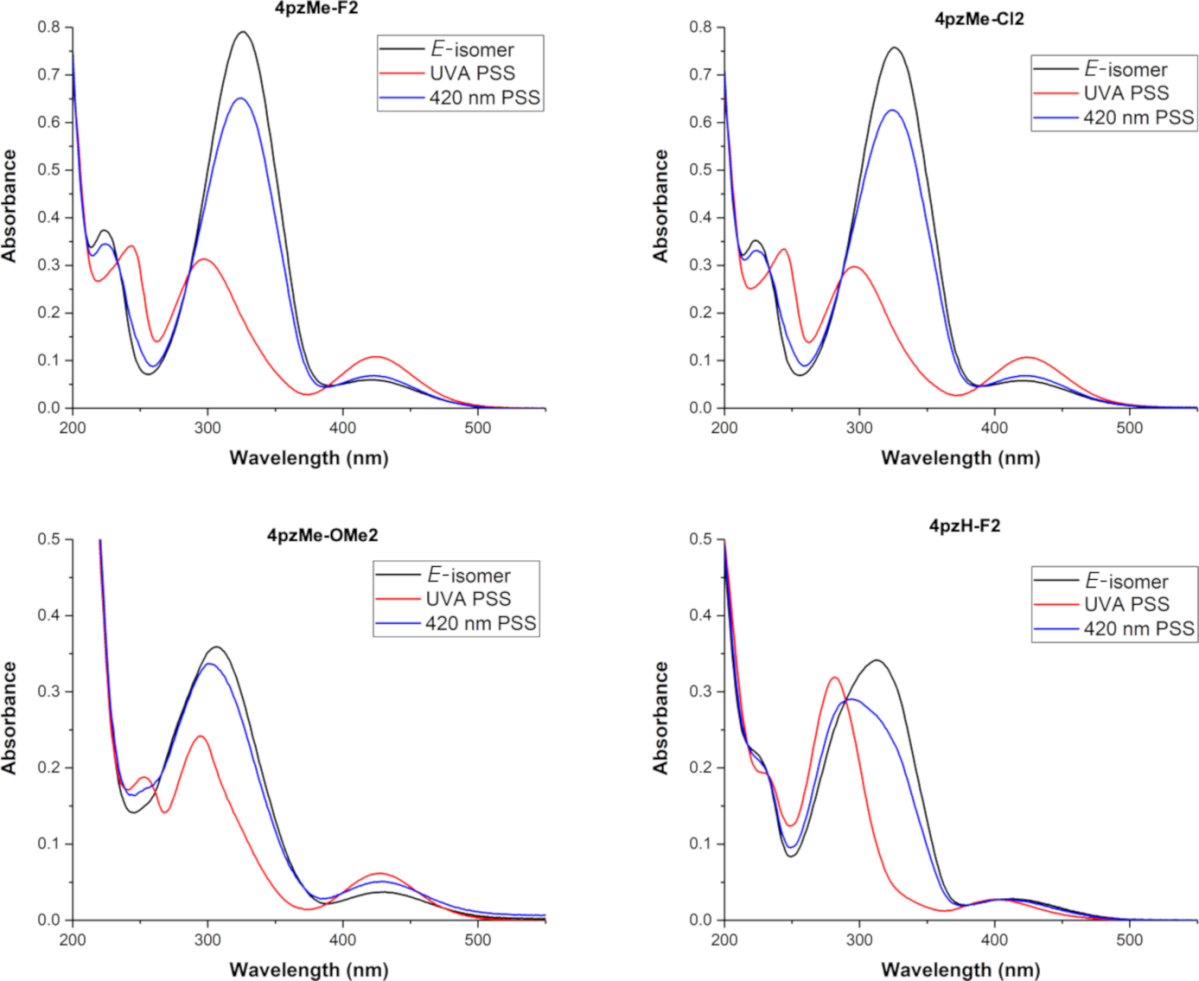
Experimental UV–vis spectra of **4pzMe-F2**, **4pzMe-Cl2**, **4pzMe-OMe2** and **4pzH-F2** in MeCN at 25 µM.

The experimental photoswitching performance of the synthesized **4pzMe-F2, 4pzMe-Cl2** and **4pzMe-OMe2** photoswitches was assessed and compared to the computational work (see Figure 8 and Table 3). In all cases, 420 nm light was used to promote *Z*–*E* switching and access a photostationary state (PSS) enriched with the *E-*isomer, whereas UVA light was used to obtain the *Z*-isomer-enriched PSS. Theoretical π–π* band separations for the *E-*isomers of **4pzMe-F2** and **4pzMe-Cl2** (Table 2) match very closely with the experimental data (Table 3), with the exception of the methoxy **4pzMe-OMe2** analogue. A large conformational space is expected for the twisted **4pzMe-OMe2** (vide supra) due to the *ortho*-methoxy groups, which likely explains the inaccuracy of the computational predictions for this compound. The experimental π–π* band for all compounds undergoes a blue shift upon isomerization *E* to *Z*, also in good accord with the theoretical predictions (Figure 8, Table S1 and S2 in Supporting Information File 1). Compared to **4pzMe**, photoswitches **4pzMe-Cl2** and **4pzMe-OMe2** show smaller π–π* band separations, which supports the reduced *E–Z* photoisomerization efficiency (80 and 81%, respectively) upon *E*–*Z* switching (Table 3). In contrast, **4pzMe-F2** and **4pzH-F2** still provide excellent *E–Z* photoisomerization of 93% and 92%, respectively, despite the relatively small π–π* band separation (30 and 33 nm, respectively).

**Table 3:** Experimental band separation (in nm) of the characteristic UV–vis absorption bands, calculated as the λ_max_ subtraction between *E* and *Z*-isomers, and photostationary-state-conversion efficiency (PSS in %).

	*n*–π* (*E*–*Z*)	π–π* (*E*–*Z*)	PSS (% *E*)^a^	PSS (% *Z*)^b^

**4pzH**^c^	14	53	70	>98
**4pzH-F2**	13	33	42	92
**4pzMe**^c^	−16	39	>98	>98
**4pzMe-F2**	−3	30	77	93
**4pzMe-Cl2**	−1	29	85	80
**4pzMe-OMe2**	−3	13	73	81

^a^Irradiation wavelength = 420 nm. ^b^UVA irradiation. ^c^Data extracted from reference [18].

Conversely, the experimental *n*–π* band separation is very small (near complete band overlap) for the **4pzMe-X** family (Figure 8), in contrast to the theoretical predictions. We note that we previously found much better correlation for the heteroazoaryl switches without *ortho-*substituents [18]. Although the computed *n*–π* band separations do not quantitatively reproduce the experimental data, they do qualitatively predict an increased band overlap in the **4pzMe-X** family compared to **4pzH** (Table 2). Furthermore, the comparably intense *n*–π* band of the Z-**4pzMe-X** analogues, a function of the twisted (i.e., non-T-shaped) conformation of these compounds, is in agreement between theory and experiment (Supporting Information File 1, Figure S9). A significant *n*–π* band overlap directly impacts the *Z*–*E* photoconversion efficiency, and thus lower PSSs of 77, 85 and 73% are recorded for **4pzMe-F2, 4pzMe-Cl2** and **4pzMe-OMe2**, respectively, versus >98% for **4pzMe**. Similarly, **4pzH-F2** possesses a lower PSS versus **4pzH** (42% versus 70%). While inaccuracies in the DFT calculations may explain the differences observed, differences in the excited-state profiles and quantum yields for the photoisomerization pathway may also contribute to the lack of correlation between band overlap and photoisomerization efficiency [35].

## Conclusion

We aimed to combine two features now known to significantly improve the performance of azoarene photoswitches – *ortho*-substitution of the benzene ring(s) and replacement of one of the benzenes for a pyrazole (to give an arylazopyrazole) – into a single switch unit. We show that *ortho*-benzene substitution of the arylazopyrazoles drastically increases the *Z*-isomer stability and allows further tuning of their addressability. This in turn has enabled us to discover **4pzH-F2**; a novel azoheteroarene photoswitch with the longest reported thermal half-life in the literature to date of ≈46 years. Many of the molecular features that we previously determined to be important for arylazopyrazole light absorption and *Z*-isomer half-life, such as the key role of conformation, hold true for these new derivatives. We believe our data provide important results for the further development of high-performance azo switches, which will open up possibilities in a large range of applications.

## Experimental

**Computational details**. Theoretical calculations were performed using the Gaussian 16 (revision A03) suite of programs [36]. Several rotational conformers of the studied compounds are available due to free rotation of the aromatic rings along single bonds linked to the azo bridge. All the possible conformers both in the *Z*- and *E*-forms were fully optimized by using the hybrid exchange-correlation PBE0 functional [37] including the Grimme’s dispersion correction in its latest version (D3) [38]. The split-valence Pople’s basis set 6-31G** was used throughout [39]. Transition states were optimized by using the Berny algorithm at the same level of theory [40]. Theoretical calculations were carried out in the gas phase. Theoretical kinetic studies were carried out at the PBE0-D3/6-31G** level of theory by considering all possible transition states and minimum-energy conformers for the *Z*-isomer. All the transition states were characterized by one imaginary frequency of approximately (440–520)i cm^−1^. The reliability of the single-determinant Kohn–Sham DFT approach to describe correctly the transition state energy/geometry was assessed by calculating the energy difference between the lowest-lying singlet excited state and the ground state at the transition state geometry for **4pzH** and **4pzMe**. Theoretical calculations at the PBE0-D3/6-31G** level indicate that the lowest-lying S1 state lies >1 eV above in energy with respect to the ground state, and therefore the DFT approach is accurate enough for TS analysis. We refer to our prior publication [18] for details of the theoretical protocol used to compute half-lives. The Boltzmann distribution was applied to the energy barriers [18] and to the relative energy differences between all possible *Z*-conformers. Note that half-lives are inversely proportional to the rate constant, and this is exponentially dependent on the free-energy barrier according to Eyring theory. Thus, a small variation in the energy barrier of <1 kcal/mol leads to a change of few orders of magnitude in half-life. Vertical electronic transition energies for the ground state geometries of both *Z*- and *E*-isomers were computed under the time-dependent density functional theory (TDDFT) approach [41–42]. The 20 lowest-lying singlet excited states were calculated in all conformers at the TD-PBE0/6-31G** level of theory in the gas phase. Solvent effects in conformational stability, half-life times and excitation energies, were analyzed under the polarizable continuum model (PCM) and acetonitrile as solvent (Tables S3 and S4 in Supporting Information File 1). Theoretical calculations indicate that, although the solvent model impacts quantitatively in the parameters analyzed, qualitative rankings are maintained. Atomic charges were calculated by performing a natural bond orbital (NBO) analysis [43]. Wiberg bond indeces (WI) were calculated by using the Gaussian NBO version 3.1 [44] as implemented in Gaussian 16 (revision A03) through the analysis of the SCF density calculated at the PBE0/6-31G** level of theory. The noncovalent index (NCI) for the different compounds was computed using the NCIPLOT program [45–46].

**Synthesis and characterization**. Details for the synthesis and experimental characterization of **4pzMe-F2, 4pzMe-Cl2, 4pzMe-OMe2** and **4pzH-F2** can be found in Supporting Information File 1.

## Supporting Information

Raw data can be found at doi:10.14469/hpc/6203

File 1 Theoretical calculations, synthetic methods, experimental characterization and X-ray crystallography data.

File 2 CIF files for **4pzMe-F2**, **4pzMe-OMe2**, **4pzMe-OMe2_diethylether** and **4pzH-F2**.
