# Supplementary material for: A combinatorial approach to improving the performance of azoarene photoswitches
Source: Beilstein J Org Chem. 2019 Nov 14;15:2753–64. doi: 10.3762/bjoc.15.266 (PMC6880842; doi:10.3762/bjoc.15.266)
Supplement: File 1 — Theoretical calculations, synthetic methods, experimental characterization and X-ray crystallography data. [file Beilstein_J_Org_Chem-15-2753-s001.pdf]

BEILSTEIN JOURNAL OF ORGANIC CHEMISTRY

Supporting Information

for

A combinatorial approach to improving the performance of azoarene photoswitches

Joaquin Calbo, Aditya R. Thawani, Rosina S. L. Gibson, Andrew J. P. White and Matthew J. Fuchter

Beilstein J. Org. Chem. **2019,** *15,* 2753–2764. doi:10.3762/bjoc.15.266

Theoretical calculations, synthetic methods, experimental characterization and X-ray crystallography data

License and Terms: This is a supporting information file under the terms of the Creative Commons Attribution License (http://creativecommons.org/licenses/by/4.0). Please note that the reuse, redistribution and reproduction in particular requires that the authors and source are credited.
The license is subject to the *Beilstein Journal of Organic Chemistry* terms and conditions: (https://www.beilstein-journals.org/bjoc)

Additional theoretical data

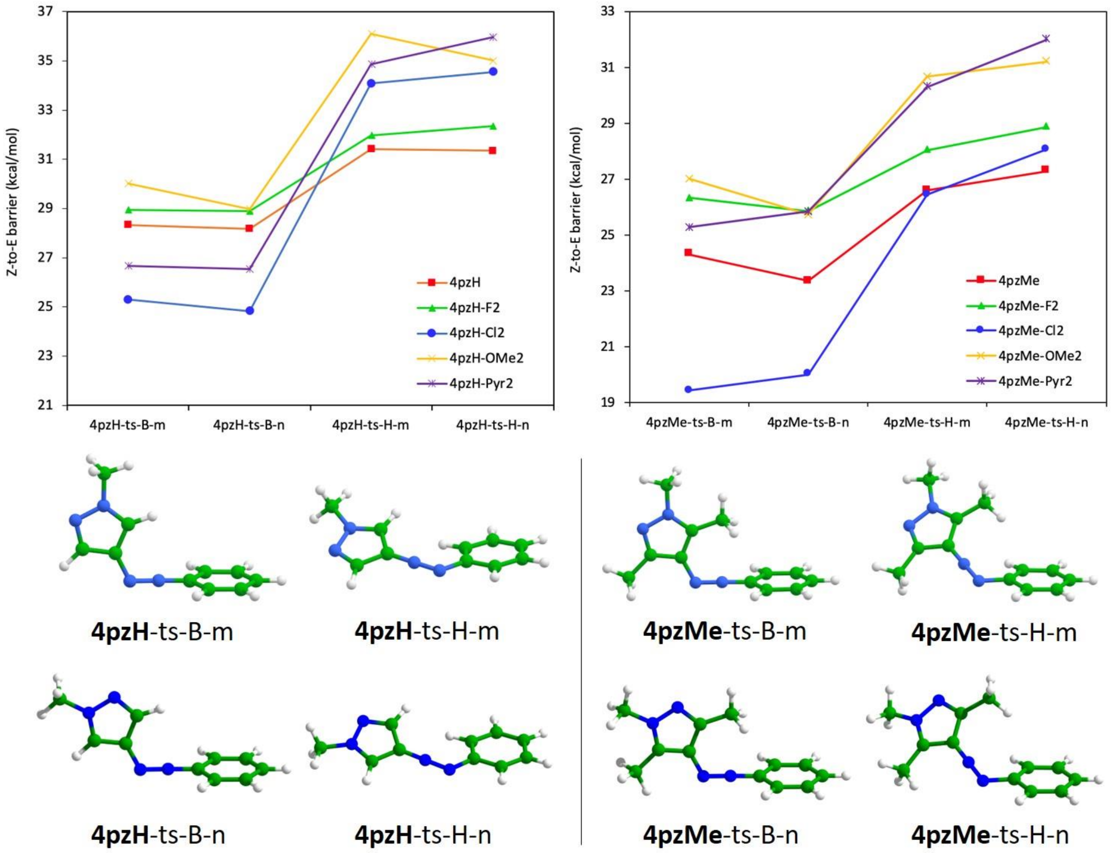

Figure S1: Free-energy barriers calculated for the *Z*–*E* thermal isomerization process in the different pathways considering all possible conformers. Representative transition-state geometries for **4pzH** and **4pzMe** photoswitches are displayed (bottom). “B” and “H” refer to transition states in which the azo N atom is inverted adjacent to the benzene (B) or the heteroring (H) moiety, respectively. The “m” and “n” labels refer to the relative orientation of the heteroring with respect to the benzene moiety: *N*-methyl “m” or =N– atom “n” close to the benzene ring.

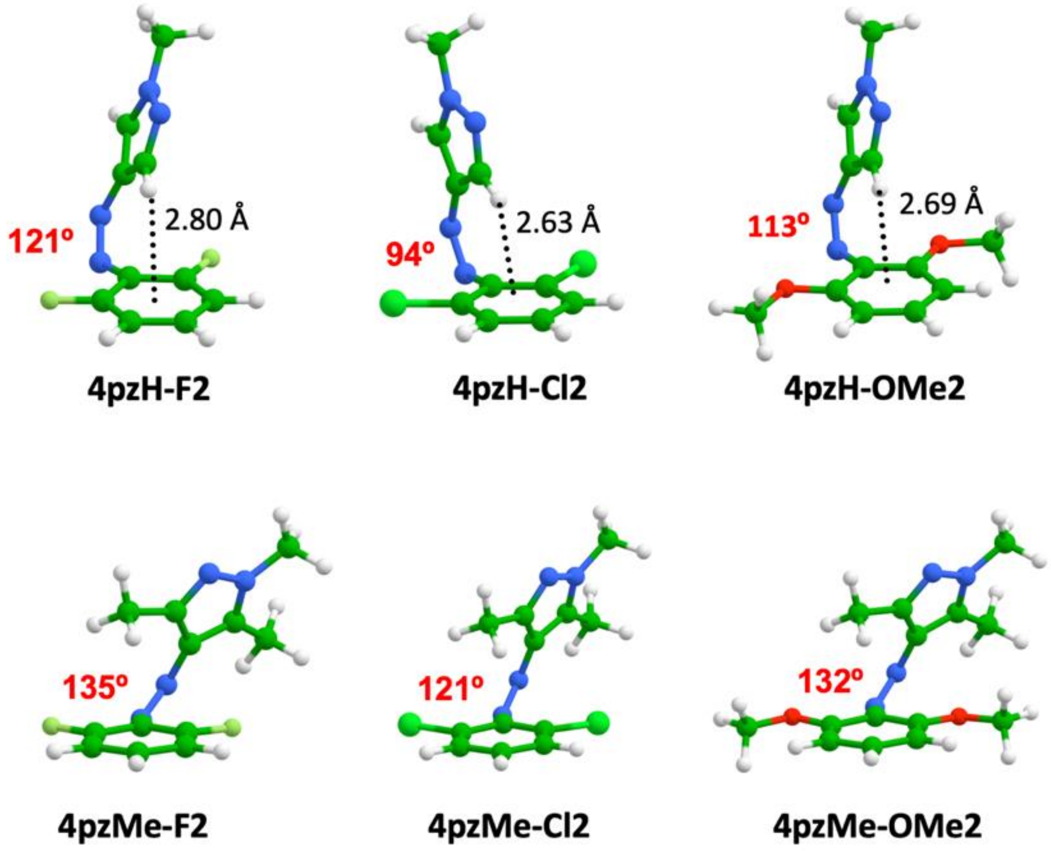

Figure S2: Minimum-energy structure calculated at the PBE0-D3/6-31G** level of theory for representative T-shaped and twisted *Z*-isomers of *ortho*-substituted **4pzH-X** and **4pzMe-X** photoswitches, respectively. Short CH···π distances and tilting dihedral angle θ as defined in Figure 3 are shown.

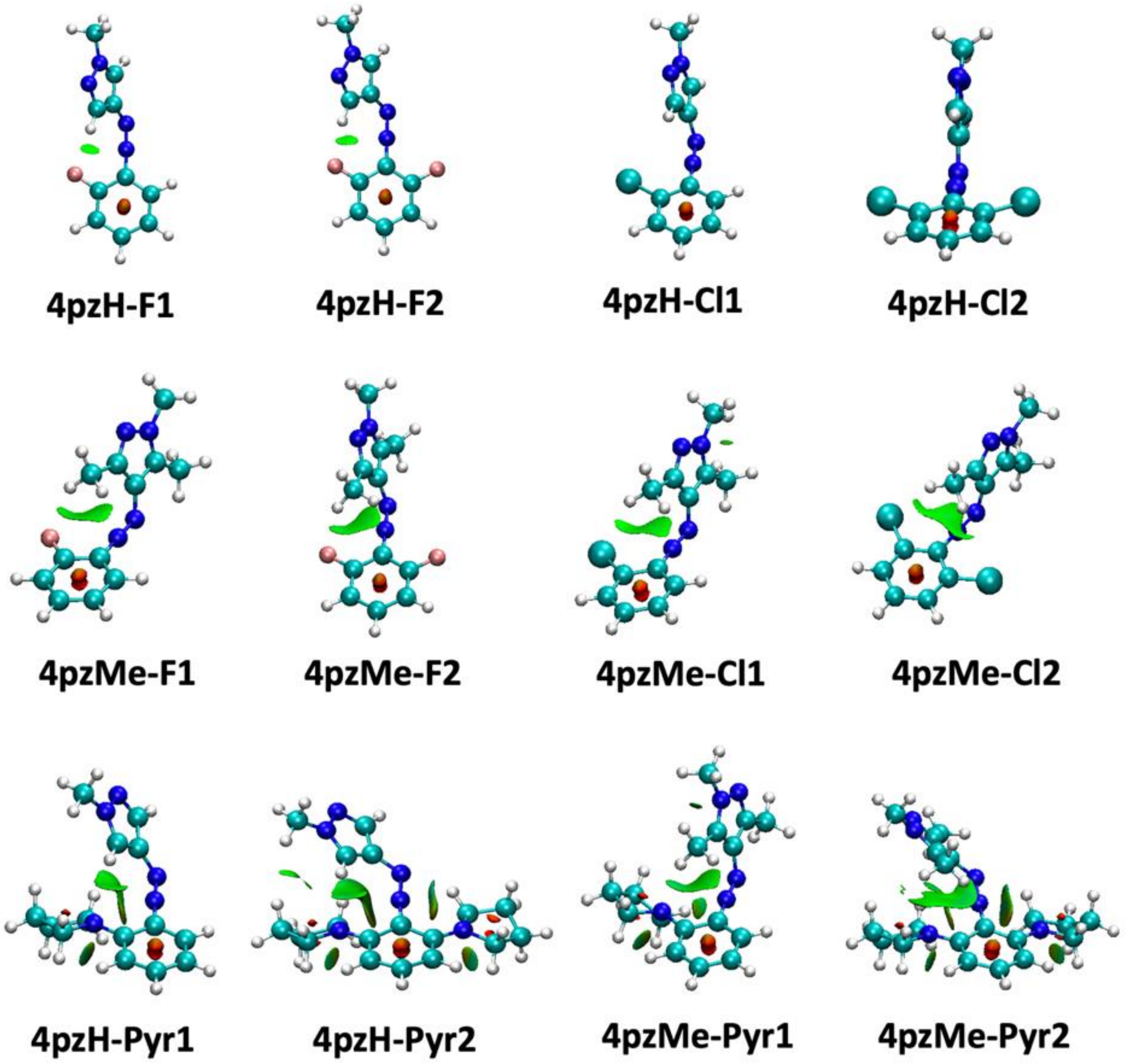

Figure S3. Non-covalent index (NCI) surfaces calculated for representative *ortho*-substituted arylazopyrazoles at the lowest-lying transition-state geometry.

Our previous studies reported a reasonably good correlation between the strength of the N=N azo bond and the half-life time in *Z*-arylazopyrazoles [1]. Thus, analysis of the Wiberg index of the N=N bond was demonstrated to be a diagnostic tool for predicting *Z*-isomer half-life in the heteroarylazo switches. The Wiberg index directly correlates with the azo bond strength and thus with the N=N distance (Figure S4).

However, a quick inspection of the relationship between the computed Wiberg indexes and the theoretical $t_{1/2}$ (Figure S5) for these *ortho*-substituted derivatives, suggests a weaker correlation for our new arylazopyrazoles. The pyrrolidine-substituted photoswitches were the largest outliers and so were removed from the comparison, resulting in an improved correlation between Wiberg index and $t_{1/2}$ (Figure S6). Nonetheless, for the halogen-substituted photoswitches, similar Wiberg indexes are computed (1.837, 1.838, 1.834 and 1.822 for **4pzH-F2**, **4pzH-Cl2**, **4pzMe-F2** and **4pzMe-Cl2**, respectively) whereas very large differences in half-life times are predicted (from 5 days in **4pzH-Cl2** to ca. 4000 days in **4pzH-F2**, or from 2 minutes in **4pzMe-Cl2** to 26 days in **4pzMe-F2**). Thus, it would seem that *ortho*-benzene substitution presents a limitation with respect to the use of the Wiberg index as a parameter to estimate the *Z*-isomer half-life.

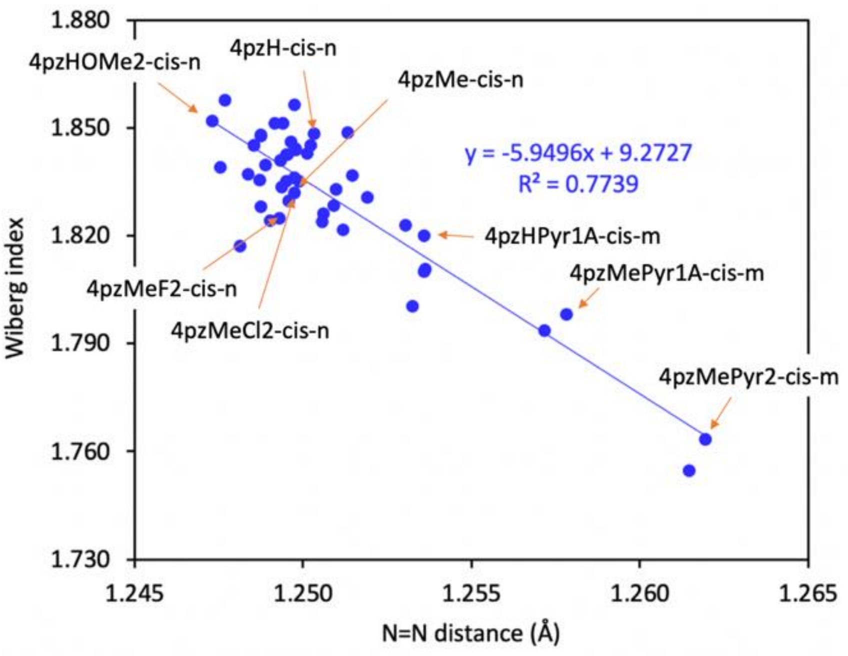

Figure S4: Relationship between the Wiberg index and the azo N=N distance for all the conformers studied.

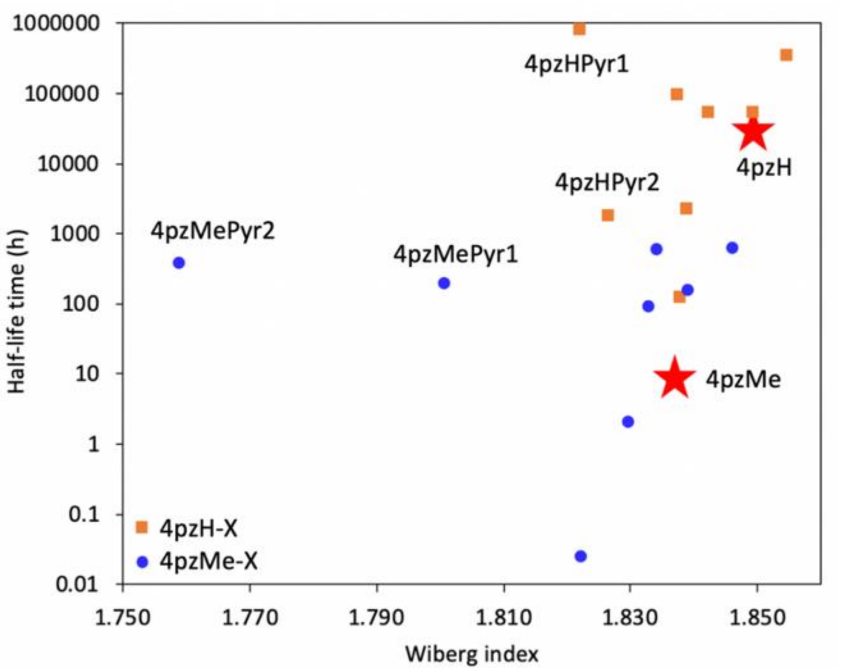

Figure S5: Relationship between the theoretical half-life time and the Wiberg index for the list of photoswitches under study in this paper.

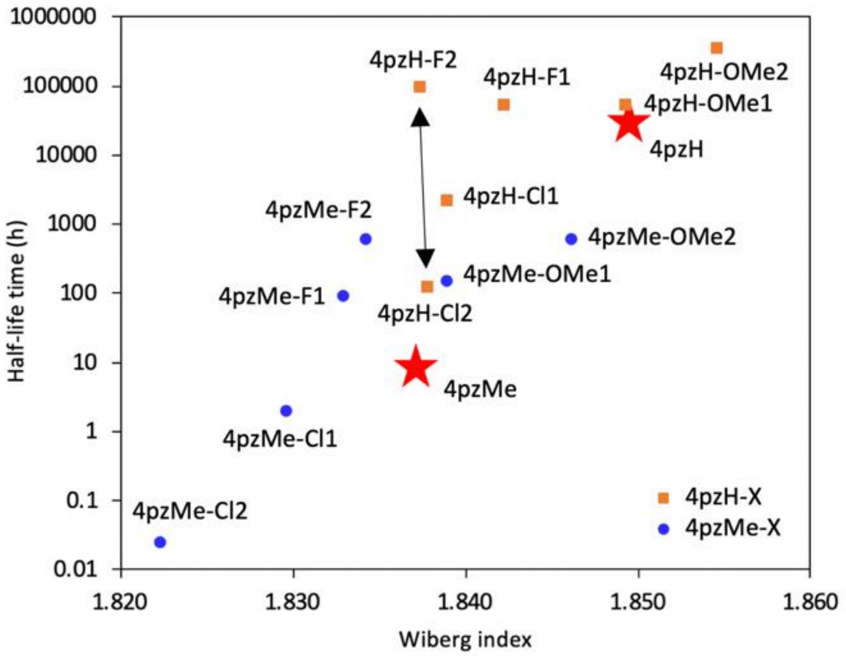

Figure S6: Relationship between the theoretical half-life time and the Wiberg index for the list of photoswitches under study in this paper excluding the pyrrolidine-based derivatives.

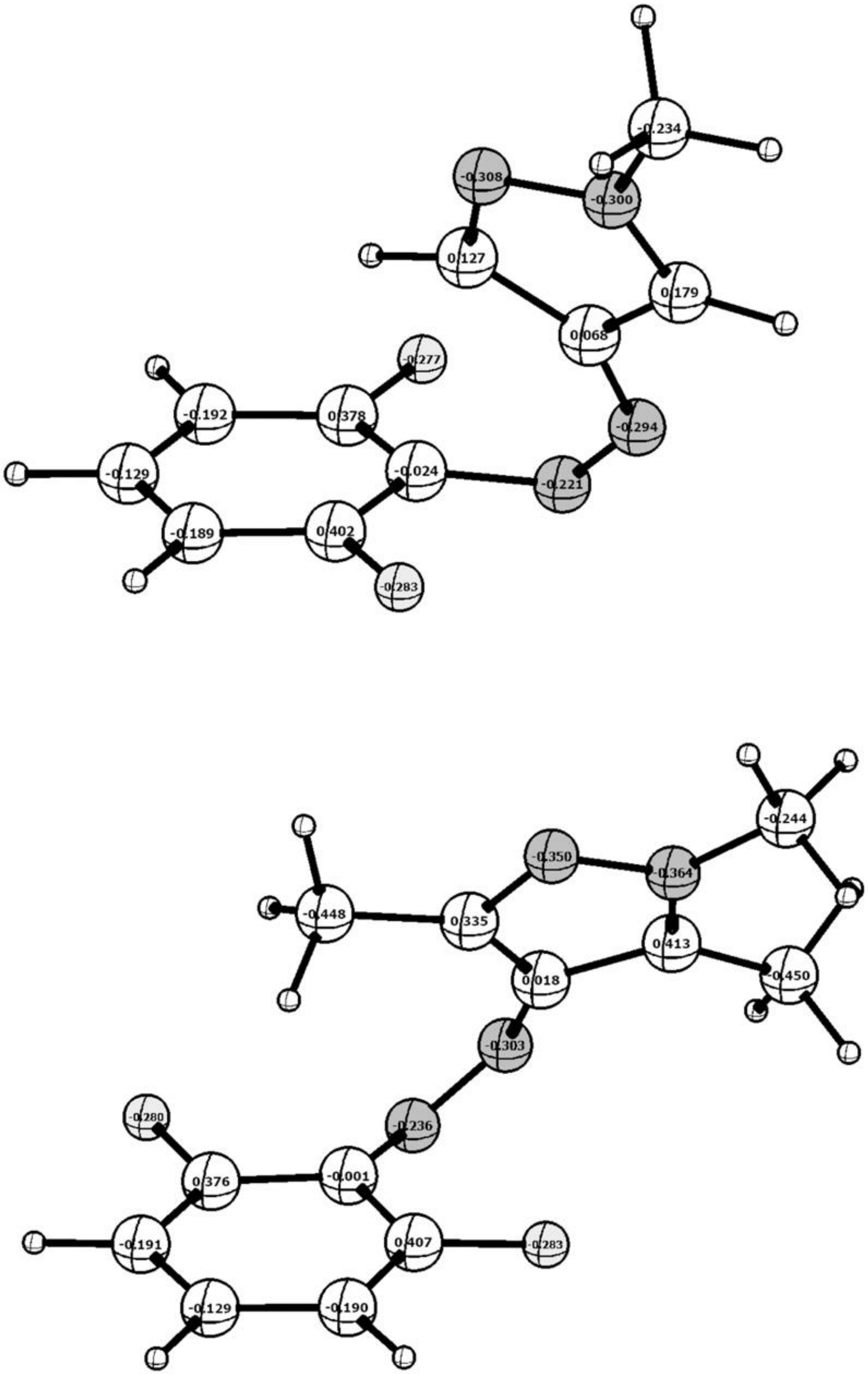

Figure S7: Atomic charges calculated for the *Z*-isomer minimum-energy geometry of **4pzH-F2** (top) and **4pzMe-F2** (bottom).

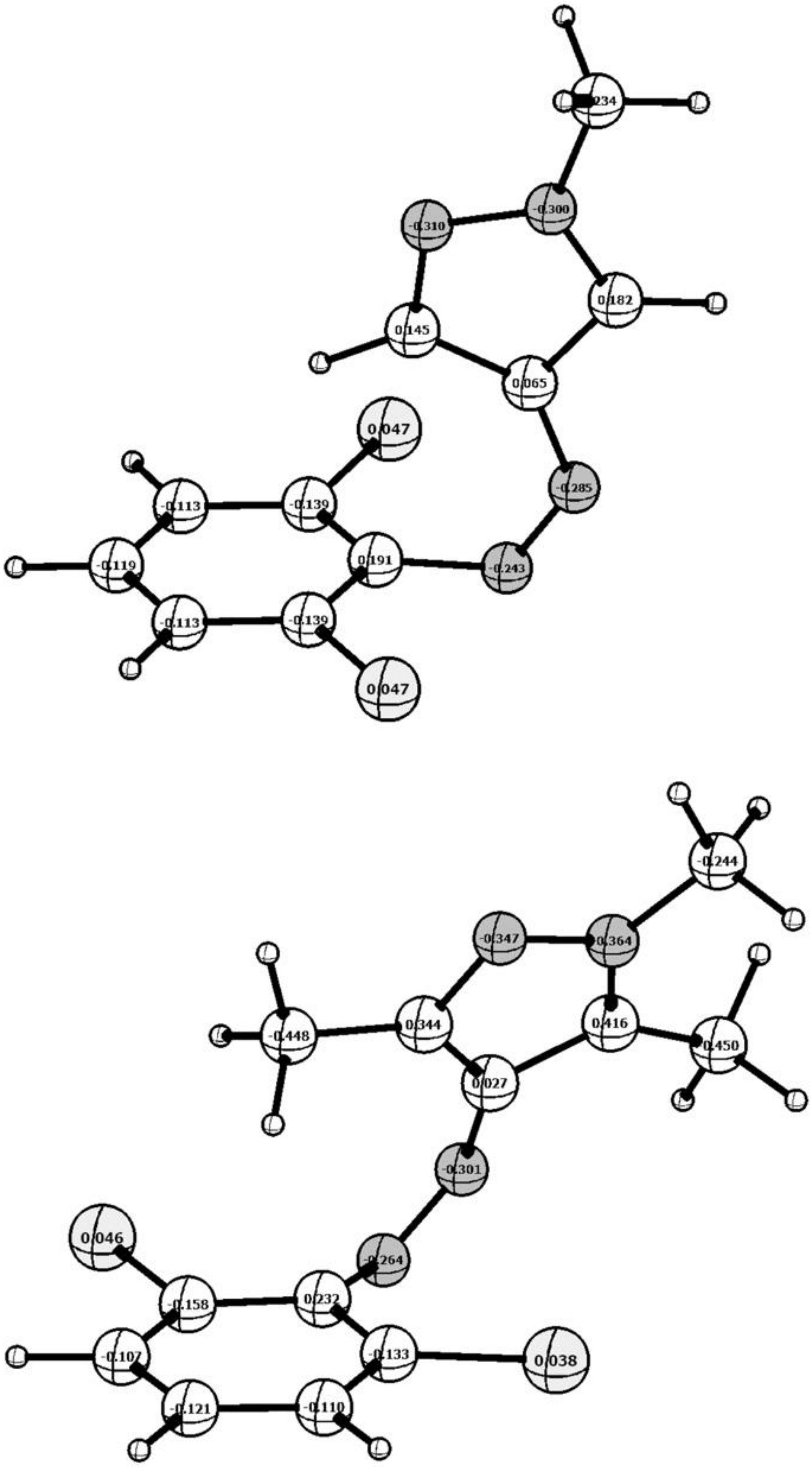

Figure S8: Atomic charges calculated for the *Z*-isomer minimum-energy geometry of **4pzH-Cl2** (top) and **4pzMe-Cl2** (bottom).

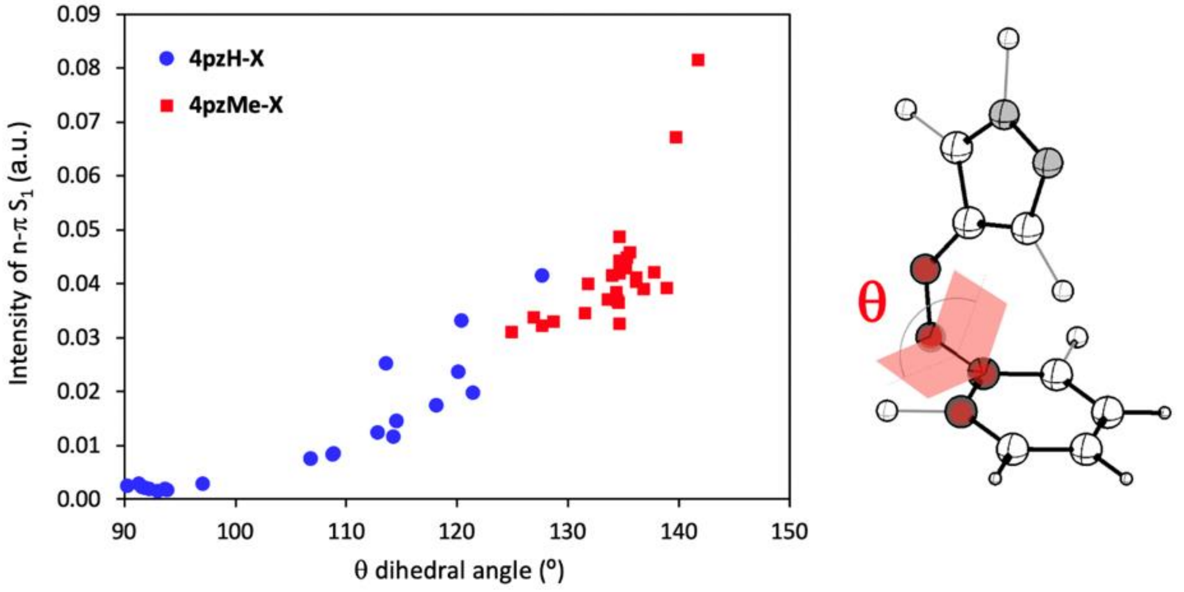

Figure S9: Intensity of the *n*–π* electronic excitation as a function of the CCNN dihedral angle θ between the aryl ring and the azo moiety.

<u>Conformer labelling</u>: "1" and "2" indicate the number of *ortho*-substitutions; "m" and "n" labels refer to the relative orientation of the heteroring with respect to the benzene moiety: N-methyl "m" or =N– atom "n" close to the benzene ring; and "X" and "Y" indicate the target *ortho* position in mono-substituted photoswitches (see Table 1).

Table S1: Excitation energies and oscillator strengths for the characteristic *n*–π* and π–π* transitions in *Z*-isomers.

	n-π* excitation			π-π* excitation[a]		
	E (eV)	E (nm)	f	E (eV)	E (nm)	f
4pzH-m	3.0264	410	0.0020	4.7832	259	0.13
4pzH-n	3.0332	409	0.0019	4.8637	255	0.12
4pzHCl1X-m	3.0311	409	0.0021	4.7692	260	0.15
4pzHCl1X-n	3.0400	408	0.0027	4.9402	251	0.25
4pzHCl1Y-m	3.0318	409	0.0021	4.7510	261	0.15
4pzHCl1Y-n	3.0401	408	0.0027	4.9402	251	0.25
4pzHCl2-m	3.0376	408	0.0018	4.7328	262	0.23
4pzHCl2-n	3.0570	406	0.0017	4.8438	256	0.27
4pzHF1X-m	2.9588	419	0.0084	4.9206	252	0.20
4pzHF1X-n	2.9123	426	0.0174	4.8819	254	0.30
4pzHF1Y-m	2.9595	419	0.0083	4.9206	252	0.20
4pzHF1Y-n	2.9123	426	0.0174	4.8819	254	0.30
4pzHF2-m	2.9441	421	0.0116	4.8062	258	0.23
4pzHF2-n	2.9229	424	0.0197	4.8438	256	0.28
4pzHOMe1X-m	2.9192	425	0.0075	4.8438	256	0.15
4pzHOMe1X-n	2.8829	430	0.0144	4.8819	254	0.30
4pzHOMe1Y-m	2.9194	425	0.0075	4.8438	256	0.15
4pzHOMe1Y-n	2.8827	430	0.0144	4.8819	254	0.30
4pzHOMe2-m	2.9413	422	0.0015	4.8249	257	0.20
4pzHOMe2-n	2.8918	429	0.0122	4.8819	254	0.25
4pzHPyr1X-m	2.6420	469	0.0252	4.5588	272	0.10
4pzHPyr1X-n	2.6557	467	0.0331	4.6269	268	0.13
4pzHPyr1Y-m	2.6267	472	0.0236	4.5091	275	0.10
4pzHPyr1Y-n	2.6334	471	0.0415	4.4765	277	0.12
4pzHPyr2-m	2.7493	451	0.0023	4.6269	268	0.15
4pzHPyr2-n	2.6266	472	0.0052	4.7328	262	0.15
4pzMe-m	2.6610	466	0.0389	4.1751	297	0.10
4pzMe-n	2.6960	460	0.0419	4.3206	287	0.18
4pzMeCl1X-m	2.7119	457	0.0401	4.2177	294	0.10
4pzMeCl1X-n	2.7424	452	0.0418	4.3972	282	0.20
4pzMeCl1Y-m	2.7696	448	0.0327	4.2321	293	0.10
4pzMeCl1Y-n	2.8108	441	0.0336	4.4928	276	0.18
4pzMeCl2-m	2.8094	441	0.0319	4.2177	294	0.10
4pzMeCl2-n	2.8513	435	0.0309	4.5588	272	0.24
4pzMeF1X-m	2.7523	450	0.0426	4.2321	293	0.07
4pzMeF1X-n	2.7552	450	0.0455	4.3972	282	0.19
4pzMeF1Y-m	2.7491	451	0.0388	4.1751	297	0.10
4pzMeF1Y-n	2.7645	448	0.0409	4.3509	285	0.20
4pzMeF2-m	2.8352	437	0.0414	4.2034	295	0.10
4pzMeF2-n	2.8241	439	0.0438	4.3972	282	0.18
4pzMeOMe1X-m	2.7513	451	0.0441	4.3056	288	0.14
4pzMeOMe1X-n	2.7557	450	0.0485	4.4444	279	0.18
4pzMeOMe1Y-m	2.7333	454	0.0324	4.1892	296	0.10
4pzMeOMe1Y-n	2.7409	452	0.0366	4.3816	283	0.19
4pzMeOMe2-m	2.8055	442	0.0344	4.3206	287	0.15
4pzMeOMe2-n	2.7956	443	0.0397	4.4604	278	0.23
4pzMePyr1X-m	2.5757	481	0.0362	4.2177	294	0.10
4pzMePyr1X-n	2.6059	476	0.0383	4.3206	287	0.14
4pzMePyr1Y-m	2.5816	480	0.0670	4.2177	294	0.12
4pzMePyr1Y-n	2.5907	479	0.0814	4.3056	288	0.18
4pzMePyr2-m	2.4752	501	0.0368	4.1892	296	0.10
4pzMePyr2-n	2.5041	495	0.0446	4.3056	288	0.15

[a] Excitation energies and intensities for the π-π* transition in Z isomers were obtained from the simulated absorption spectra through Gaussian convolution of all the computed electronic excitations with a full-width-at-half-maximum of 0.2 eV.

Table S2: Excitation energies and oscillator strengths for the characteristic *n*–π* and π–π* transitions in *E*-isomers.

	n-π* excitation			π-π* excitation		
	E (eV)	E (nm)	f	E (eV)	E (nm)	f
4pzH-m	2.7745	447	0.0000	3.8991	318	0.7770
4pzH-n	2.7727	447	0.0000	3.9699	312	0.8187
4pzH-Cl1X-m	2.6898	461	0.0000	3.7638	329	0.6834
4pzH-Cl1X-n	2.6846	462	0.0000	3.8218	324	0.7107
4pzH-Cl1Y-m	2.7023	459	0.0158	3.9933	310	0.5169
4pzH-Cl1Y-n	2.7101	458	0.0137	4.0524	306	0.5281
4pzH-Cl2-m	2.7320	454	0.0155	4.0759	304	0.5298
4pzH-Cl2-n	2.7443	452	0.0152	4.1463	299	0.5341
4pzH-F1X-m	2.7497	451	0.0000	3.8238	324	0.7652
4pzH-F1X-n	2.7455	452	0.0000	3.8881	319	0.8034
4pzH-F1Y-m	2.6976	460	0.0000	3.9325	315	0.7779
4pzH-F1Y-n	2.6987	459	0.0000	4.0052	310	0.8136
4pzH-F2-m	2.6560	467	0.0000	3.8992	318	0.8221
4pzH-F2-n	2.6529	467	0.0000	3.9732	312	0.8805
4pzH-OMe1X-m	2.7015	459	0.0000	3.5866	346	0.5749
4pzH-OMe1X-n	2.6997	459	0.0000	3.6305	342	0.5899
4pzH-OMe1Y-m	2.6560	467	0.0000	3.7510	331	0.5748
4pzH-OMe1Y-n	2.6613	466	0.0000	3.8121	325	0.5693
4pzH-OMe2-m	2.6218	473	0.0201	3.8302	324	0.6526
4pzH-OMe2-n	2.6415	469	0.0207	3.8951	318	0.6432
4pzH-Pyr1X-m	2.8232	439	0.0603	3.2570	381	0.2066
4pzH-Pyr1X-n	2.8281	438	0.0553	3.2930	377	0.1951
4pzH-Pyr1Y-m	2.7637	449	0.0003	3.0612	405	0.3504
4pzH-Pyr1Y-n	2.7711	447	0.0002	3.0817	402	0.3677
4pzH-Pyr2-m	2.7419	452	0.0594	2.9836	347	0.2708
4pzH-Pyr2-n	2.7484	451	0.0556	2.9991	346	0.2704
4pzMe-m	2.7358	453	0.0000	3.7937	327	0.7551
4pzMe-n	2.8094	441	0.0000	3.9022	318	0.8044
4pzMe-Cl1X-m	2.7086	458	0.0000	3.7014	335	0.6954
4pzMe-Cl1X-n	2.7225	455	0.0000	3.7739	329	0.7471
4pzMe-Cl1Y-m	2.7260	455	0.0193	3.9606	313	0.5769
4pzMe-Cl1Y-n	2.7599	449	0.0148	4.0252	308	0.6282
4pzMe-Cl2-m	2.8101	441	0.0140	4.0196	308	0.5588
4pzMe-Cl2-n	2.7868	445	0.0153	4.0778	304	0.6042
4pzMe-F1X-m	2.7062	458	0.0000	3.7168	334	0.7488
4pzMe-F1X-n	2.7794	446	0.0000	3.8205	325	0.7991
4pzMe-F1Y-m	2.7136	457	0.0031	3.8639	321	0.7665
4pzMe-F1Y-n	2.7365	453	0.0000	3.9432	314	0.8306
4pzMe-F2-m	2.6379	470	0.0017	3.7961	327	0.7816
4pzMe-F2-n	2.7073	458	0.0032	3.9164	317	0.8388
4pzMe-OMe1X-m	2.6593	466	0.0000	3.5322	351	0.6283
4pzMe-OMe1X-n	2.7323	454	0.0000	3.6168	343	0.6602
4pzMe-OMe1Y-m	2.6140	474	0.0000	3.6933	336	0.6396
4pzMe-OMe1Y-n	2.6890	461	0.0000	3.7930	327	0.6481
4pzMe-OMe2-m	2.5953	478	0.0095	3.7048	335	0.6652
4pzMe-OMe2-n	2.6616	466	0.0152	3.8167	325	0.7077
4pzMe-Pyr1X-m	2.7918	444	0.0547	3.2550	381	0.2322
4pzMe-Pyr1X-n	2.8417	436	0.0591	3.3370	372	0.2002
4pzMe-Pyr1Y-m	2.7371	453	0.0885	3.1391	395	0.2328
4pzMe-Pyr1Y-n	2.7794	446	0.0888	3.1711	391	0.2540
4pzMe-Pyr2-m	2.6756	463	0.0884	2.9741	352	0.3585
4pzMe-Pyr2-n	2.6898	461	0.0845	2.9983	349	0.3668

Table S3: Solvent effect on the theoretical half-life time (in days) calculated at the PBE0-D3/6-31G** level in a single-point calculation or including geometry reoptimization, under the PCM model (acetonitrile).[a]

	Gas phase	Single-point PCM	Optimized PCM
**4pzH**	1300	3200	4000
**4pzHF2**	4100	40000	46000
**4pzHCl2**	5.1	810	62
**4pzMe**	0.38	4.2	4.1
**4pzMeF2**	26	490	110
**4pzMeCl2**	0.001	0.10	0.01

[a] In all cases, the most stable transition state conformer is the one in which the azo N atom adjacent to the benzene moiety is inverted (B-type).

Table S4: Solvent effect (PCM, acetonitrile) on the theoretical energy separation (in nm) of the characteristic electronic excitations calculated at the PBE0-D3/6-31G** level.

	Gas phase	Single-point PCM	Gas phase	Single-point PCM
	n-π*(*E*–*Z*)	n-π*(*E*–*Z*)	π-π* (*E*–*Z*)	π–π* (*E*–*Z*)
**4pzH**	38	41	58	80
**4pzHF2**	44	46	58	67
**4pzHCl2**	46	41	43	50
**4pzMe**	-15	-10	30	48
**4pzMeF2**	26	23	33	41
**4pzMeCl2**	5	7	23	21

General synthetic methods

All reagents and solvents were purchased from commercial sources and used as supplied unless otherwise indicated. All reactions were monitored by thin-layer chromatography (TLC) using Merck silica gel 60 F254 plates (0.25 mm). TLC plates were visualised using UV light (254 nm) and/or by using the appropriate TLC stain. Silica column chromatography was performed using Merck Silica Gel 60 (230–400 mesh) treated with a solvent system specified in the individual procedures. Solvents were removed by rotary evaporator at 40 °C or below, in the dark, and the compounds further dried using high vacuum pumps. Infrared spectra were recorded neat on an Agilent Cary 630 FTIR. Reported absorptions are in wavenumbers (cm^{-1}). ^1H and ^{13}C NMR were recorded on a Bruker Avance 400 spectrometer at 400 MHz and 100 MHz, respectively. Chemical shifts (δ) are quoted in ppm (parts per million) downfield from tetramethylsilane, referenced to residual solvent signals: ^1H δ = 7.26 ($CHCl_3$), 1.94 (MeCN-d_3), ^{13}C δ = 77.16 ($CDCl_3$). The following abbreviations are used to designate multiplicity within ^1H NMR analysis; s = singlet, d = doublet, t = triplet, q = quartet, m = multiplet, br. = broad signal. High-resolution mass spectra (ESI, APCI) were recorded by the Imperial College London Department of Chemistry Mass Spectroscopy Service using a Micromass Autospec Premier and Micromass LCT Premier spectrometer. LCMS analysis of compounds were conducted using a reverse phase LCMS Waters 2767 system equipped with a photodiode array and an ESI mass spectrometer using an XBridge C18 (5 μm, 100 mm × 4.6 mm) column, equipped with an XBridge C18 guard column (5 μm, 4.6 mm × 20 mm). An eluent of MeCN and H_2O was used: 0–10 min 50–98% MeCN, 10–12 min 98% MeCN, 12–13 min 98 to 50% MeCN, 13–17 min 50% MeCN.

Synthetic route

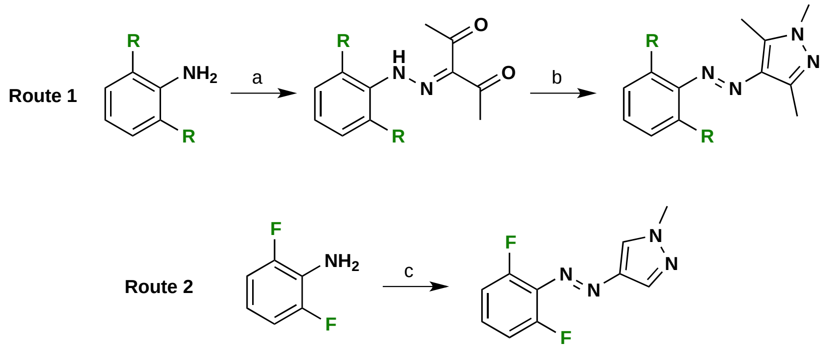

Scheme S1: (a) (i) $NaNO_2$, HCl or AcOH, 0°C (ii) Acetylacetone, NaOAc, H_2O. (b) (i) $MeNHNH_2$, EtOH or MeOH/CH_2Cl_2, reflux. (c) (i) oxone, CH_2Cl_2/H_2O (ii) 1-methyl-1H-pyrazol-4-amine, AcOH, $CHCl_3$.

3-(2-(2,6-Dimethoxyphenyl)hydrazono)pentane-2,4-dione

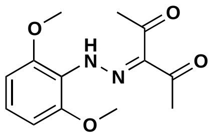

A solution of $NaNO_2$ (169 mg, 2.45 mmol) in water (0.2 mL) was added dropwise to 2,6-dimethoxyaniline (250 mg, 1.63 mmol) in 1 M HCl (5 mL) at 0 °C. The resulting solution was stirred for 0.5 h before being added dropwise to a solution of acetylacetone (0.2 mL, 2.0 mmol) and NaOAc (201 mg, 2.45 mmol) in water (2 mL) at 0 °C. Further NaOAc was added to adjust the pH to 7 resulting in a yellow-green precipitate. After 1 h, the precipitate was filtered, washed with water and dried to yield the desired product as yellow-green solid (375 mg, 1.42 mmol, 87% yield). ^{1}H NMR (400 MHz, $CDCl_3$) δ 14.67 (s, 1H), 7.11 (t, *J* = 8.4 Hz, 1H), 6.62 (d, *J* = 8.5 Hz, 2H), 3.88 (s, 6H), 2.56 (s, 3H), 2.39 (s, 3H); ^{13}C NMR (101 MHz, $CDCl_3$) δ 197.8, 196.8, 152.2, 133.6, 126.7, 119.5, 104.9, 56.4, 31.6, 26.5; HRMS (ES) *m/z* calc. for $C_{13}H_{17}N_2O_4$ $[M+H]^+$ 265.1188; found 265.1184; IR: 2964, 2832, 1653, 1517, 1354, 1326, 1255, 1180, 1105, 1095, 1025, 847.

(*E*)-4-((2,6-Dimethoxyphenyl)diazenyl)-1,3,5-trimethyl-1*H*-pyrazole

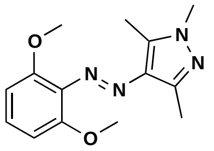

Methylhydrazine (0.10 mL, 2.0 mmol) was added to a solution of 3-(2-(2,6-dimethoxyphenyl)hydrazono)pentane-2,4-dione (350 mg, 1.32 mmol) in CH_2Cl_2 (3 mL) and MeOH (6 mL). The reaction mixture was heated at 50 °C for 1 h. Further methylhydrazine (0.10 mL, 2.0 mmol) was added and heating continued for another 0.75 h. The reaction mixture was allowed to cool to room temperature, diluted with CH_2Cl_2 (30 mL) and washed with 1 M HCl (50 mL). The organic layer was separated and the aqueous layer was extracted with CH_2Cl_2 (2 × 30 mL). The combined organic phases were washed with brine, dried over anhydrous Mg_2SO_4, concentrated in vacuo and subjected to purification by silica gel column chromatography (100% EtOAc). Recrystallization from diethyl ether produced pale orange crystals (329 mg, 1.20 mmol, 91% yield). LCMS (2.76 min); ^{1}H NMR (400 MHz, $CDCl_3$) δ 7.16 (t, J = 8.3 Hz, 1H), 6.66 (d, J = 8.4 Hz, 2H), 3.80 (s, 6H), 3.76 (s, 3H), 2.53 (s, 3H), 2.47 (s, 3H); ^{13}C NMR (101 MHz, $CDCl_3$) δ 152.3, 142.5, 137.9, 136.5, 134.8, 127.9, 105.8, 56.7, 36.0, 13.7, 10.2; HRMS (ES) *m/z* calc. for $C_{14}H_{19}N_4O_2$ $[M+H]^+$ 275.1508; found 275.1508; IR: 2990, 2935, 2836, 1587, 1476, 1433, 1409, 1366, 1251, 1108, 1031, 984, 892.

3-(2-(2,6-Difluorophenyl)hydrazono)pentane-2,4-dione

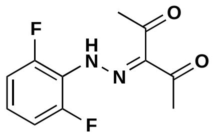

This compound was synthesized according to literature precedent [2]. ^{1}H NMR (400 MHz, $CDCl_3$) δ 14.44 (s, 1H), 7.17 – 7.04 (m, 1H), 7.03 – 6.93 (m, 2H) 2.59 (s, 3H), 2.40 (s, 3H); ^{13}C NMR (101 MHz, $CDCl_3$) δ 198.2, 197.5, 155.5, 155.4, 153.0, 152.9, 135.1, 125.5, 125.5, 125.5, 125.4, 119.8, 112.7, 112.7, 112.6, 112.5, 31.8, 26.6; ^{19}F NMR (377 MHz, $CDCl_3$) δ -124.4; HRMS (ES) *m/z* calc. for $C_{11}H_{11}N_2O_2F_2$ $[M+H]^+$ 241.0789; found 241.0796; IR: 3060, 3003, 1677, 1633, 1520.

(*E*)-4-((2,6-Difluorophenyl)diazenyl)-1,3,5-trimethyl-1*H*-pyrazole

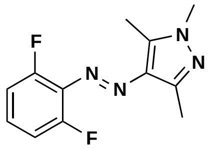

Methylhydrazine (70 µL, 1.2 mmol) was added to a solution of 3-(2-(2,6-difluorophenyl)hydrazono)pentane-2,4-dione (250 mg, 1.04 mmol) in EtOH (10 mL). The reaction mixture was refluxed for 1 h, cooled to room temperature and concentrated in vacuo. EtOAc (50 mL) and H_2O (25 mL) were added and the aqueous layer separated. The aqueous layer was extracted with EtOAc (2 × 10 mL). The combined organic phases were washed with 1 M HCl, saturated $NaHCO_3$, brine, dried over anhydrous Mg_2SO_4, concentrated in vacuo and subjected to purification by silica gel column chromatography (0–10% EtOAc/CH_2Cl_2). The title compound was obtained as orange solid (182 mg, 0.73 mmol, 70% yield). LCMS (5.83 min); ^{1}H NMR (400 MHz, $CDCl_3$) δ 7.23 – 7.15 (m, 1H), 7.01 – 6.93 (m, 2H), 3.78 (s, 3H), 2.55 (s, 3H), 2.46 (s, 3H); ^{13}C NMR (101 MHz, $CDCl_3$) δ 157.2, 157.1, 154.6, 154.6, 142.6, 140.0, 136.8, 132.3, 128.4, 128.3, 128.2, 112.5, 112.4, 112.3, 112.3, 36.2, 13.9, 10.0; HRMS (ES) *m/z* calc. for $C_{12}H_{13}N_4F_2$ $[M+H]^+$ 251.1108; found 251.1108; IR: 2985, 2920, 1610, 1506, 1422.

3-(2-(2,6-Dichlorophenyl)hydrazono)pentane-2,4-dione

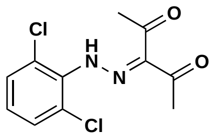

This compound was synthesized according to literature precedent [3]. ^{1}H NMR (400 MHz, $CDCl_3$) δ 14.54 (s, 1H), 7.38 (d, *J* = 8.1 Hz, 2H), 7.10 (dd, *J* = 8.4, 7.8 Hz, 1H), 2.62 (s, 3H), 2.44 (s, 3H); ^{13}C NMR (101 MHz, $CDCl_3$) δ 198.3, 197.6, 135.5, 134.6, 129.8, 129.2, 129.0, 127.6, 126.9, 31.8, 26.9; HRMS (ES) *m/z* calc. for $C_{11}H_{11}N_2O_2Cl_2$ $[M+H]^+$ 273.0198; found 273.0193; IR: 3076, 2999, 2919, 1668, 1631, 1506.

(*E*)-4-((2,6-Dichlorophenyl)diazenyl)-1,3,5-trimethyl-1*H*-pyrazole

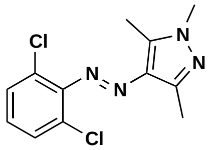

Methylhydrazine (93 µL, 1.8 mmol) was added to a solution of 3-(2-(2,6-dichlorophenyl)hydrazono)pentane-2,4-dione (400 mg, 1.47 mmol) in EtOH (10 mL). The reaction mixture was refluxed for 1 h, cooled to room temperature and concentrated in vacuo. EtOAc (50 mL) and H_2O (25 mL) were added and the aqueous layer separated. The aqueous layer was extracted with EtOAc (2 × 10 mL). The combined organic phases were washed with 1 M HCl, saturated $NaHCO_3$, brine, dried over anhydrous Mg_2SO_4, concentrated in vacuo and subjected to purification by silica gel column chromatography (0–10% EtOAc/CH_2Cl_2). The title compound was obtained as orange solid (261 mg, 0.93 mmol, 63% yield). LCMS (7.64 min); ^{1}H NMR (400 MHz, $CDCl_3$) δ 7.37 (d, *J* = 8.1 Hz, 2H), 7.11 (dd, *J* = 8.4, 7.8 Hz, 1H), 3.79 (s, 3H), 2.57 (s, 3H), 2.49 (s, 3H); ^{13}C NMR (101 MHz, $CDCl_3$) δ 148.9, 142.9, 140.3, 135.9, 129.2, 127.6, 127.4, 36.2, 13.9, 10.1; HRMS (ES) *m/z* calc. for $C_{12}H_{13}N_4Cl_2$ $[M+H]^+$ 283.0517; found 283.0518; IR: 2976, 2919, 1554, 1427, 1392, 1366.

(*E*)-4-((2,6-Difluorophenyl)diazenyl)-1-methyl-1*H*-pyrazole

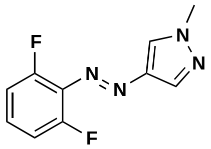

To a stirred biphasic mixture of 2,6-difluoroaniline (100 mg, 0.78 mmol) in CH_2Cl_2 (10 mL) and H_2O (10 mL) was added a solution of oxone (288 mg, 0.94 mmol) in H_2O (3 mL). The reaction mixture was stirred in the dark at room temperature for 18 h. CH_2Cl_2 (20 mL) and H_2O (5 mL) were added and the aqueous layer separated. The aqueous layer was extracted with CH_2Cl_2 (2 × 15 mL). The combined organic phases were washed with brine, dried over anhydrous Mg_2SO_4 and concentrated in vacuo to yield crude 1,3-difluoro-2-nitrosobenzene as tan solid. This was dissolved in AcOH acid/$CHCl_3$ 4:1 and added to a solution of 1-methyl-1*H*-pyrazol-4-amine (76 mg, 0.78 mmol) in AcOH (1 mL). The reaction mixture was stirred at room temperature for 18 h. Subsequently, the mixture was concentrated in vacuo, using PhMe to azeotrope off any AcOH, and subjected to purification by silica gel column chromatography (0–20% Et_2O/pentane). The title compound was obtained as orange solid (100 mg, 0.45 mmol, 58% yield). ^{1}H NMR (400 MHz, $CDCl_3$) δ 8.05 – 8.01 (m, 2H), 7.29 – 7.22 (m, 1H), 7.05 – 6.96 (m, 2H), 3.98 (s, 3H); ^{13}C NMR (101 MHz, $CDCl_3$) δ 157.1, 157.1, 154.6, 154.5, 142.8, 133.6, 129.6, 129.5, 129.4, 127.5, 112.7, 112.6, 112.5, 112.4, 39.8; HRMS (ES) *m/z* calc. for $C_{10}H_9N_4F_2$ $[M+H]^+$ 223.0795; found 223.0794; IR: 3111, 1534, 1472, 1450, 1386.

UV–vis, photostationary state (PSS) and thermal isomerization kinetics

All samples were irradiated using a Luzchem LZC-4V photoreactor fitted with 12 × 8W LZC-UV A (***E→Z***) or LZC-420 nm (***Z→E***) lamps. To measure UV–vis spectra, 25 µM solutions of each compound in MeCN were prepared. To determine the PSS, an ≈ 20 mM solution of the relevant compound in MeCN-d_3 was irradiated until no further change was observable by ^{1}H NMR. In order to determine the ***Z→E*** thermal isomerization kinetics an ≈ 20 mM solution of the relevant compound in MeCN-d_3 or DMSO-d_6 was irradiated using 12 × 8W LZC-UV A lamps until no further change was observable by ^{1}H NMR. The change in ***Z*** isomer percentage was then followed as a function of time by ^{1}H NMR, through integration of the relevant peaks. In between measurements, NMR samples were kept in the dark and immersed in an oil bath which was held at 25 °C. The natural log of the ***Z*** isomer concentration was plotted against time to yield a straight line, as would be expected from first order kinetics. The gradient of this line was used to calculate the rate constant for ***Z→E*** conversion from which the thermal half-life could be calculated:

$$t_{1/2} = \frac{\ln(2)}{k}$$

For the case of **4pzH-F2**, kinetic studies were instead performed at elevated temperatures (vide infra) so as to generate an Eyring plot which in turn allowed determination of the thermal half-life at 25 ºC via extrapolation.

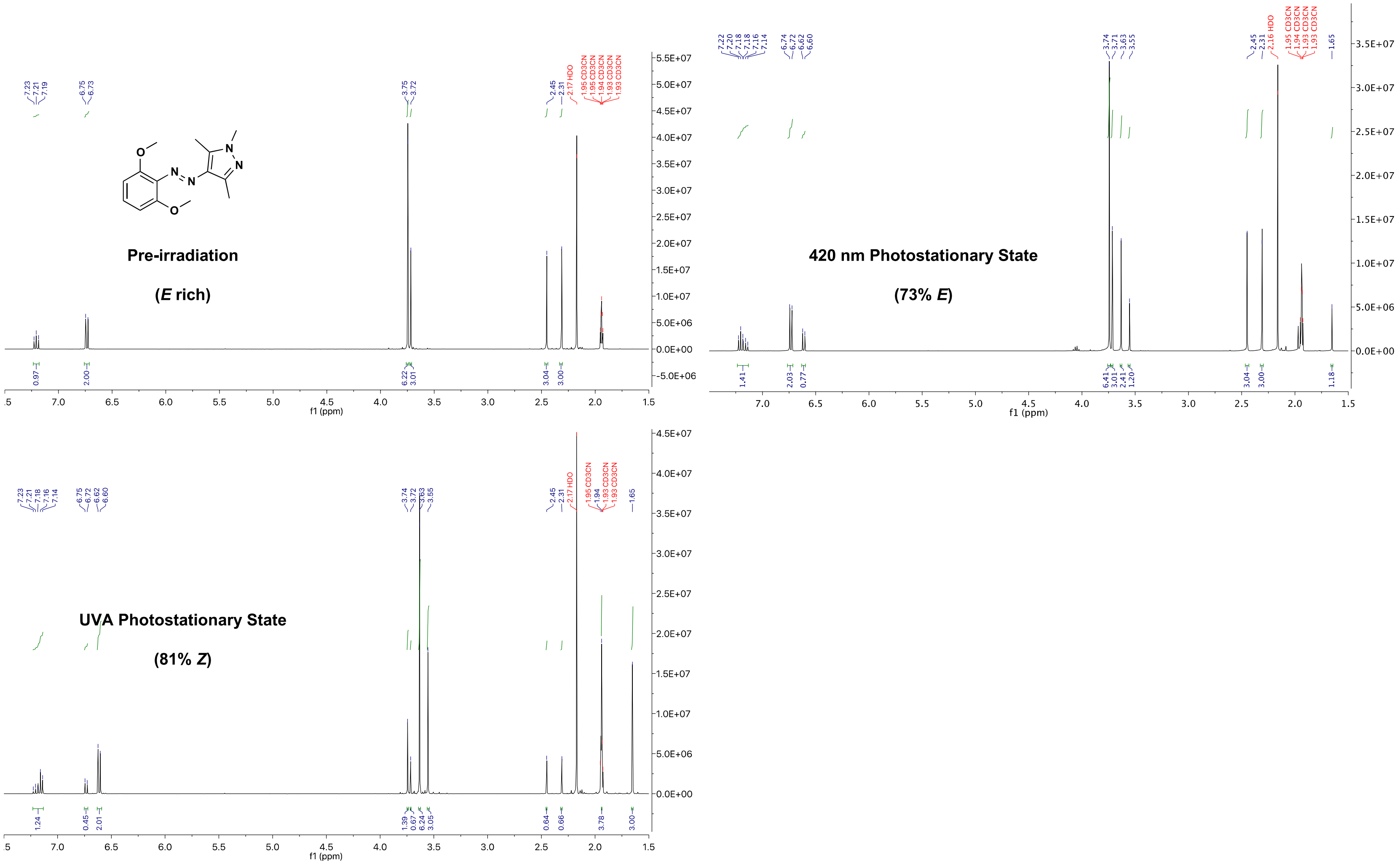

Figure S10. 4pzMe-OMe2 ^{1}H NMR spectra of the ***E***-rich state, UVA PSS and 420 nm PSS in MeCN-d$_3$ at ≈ 20 mM.

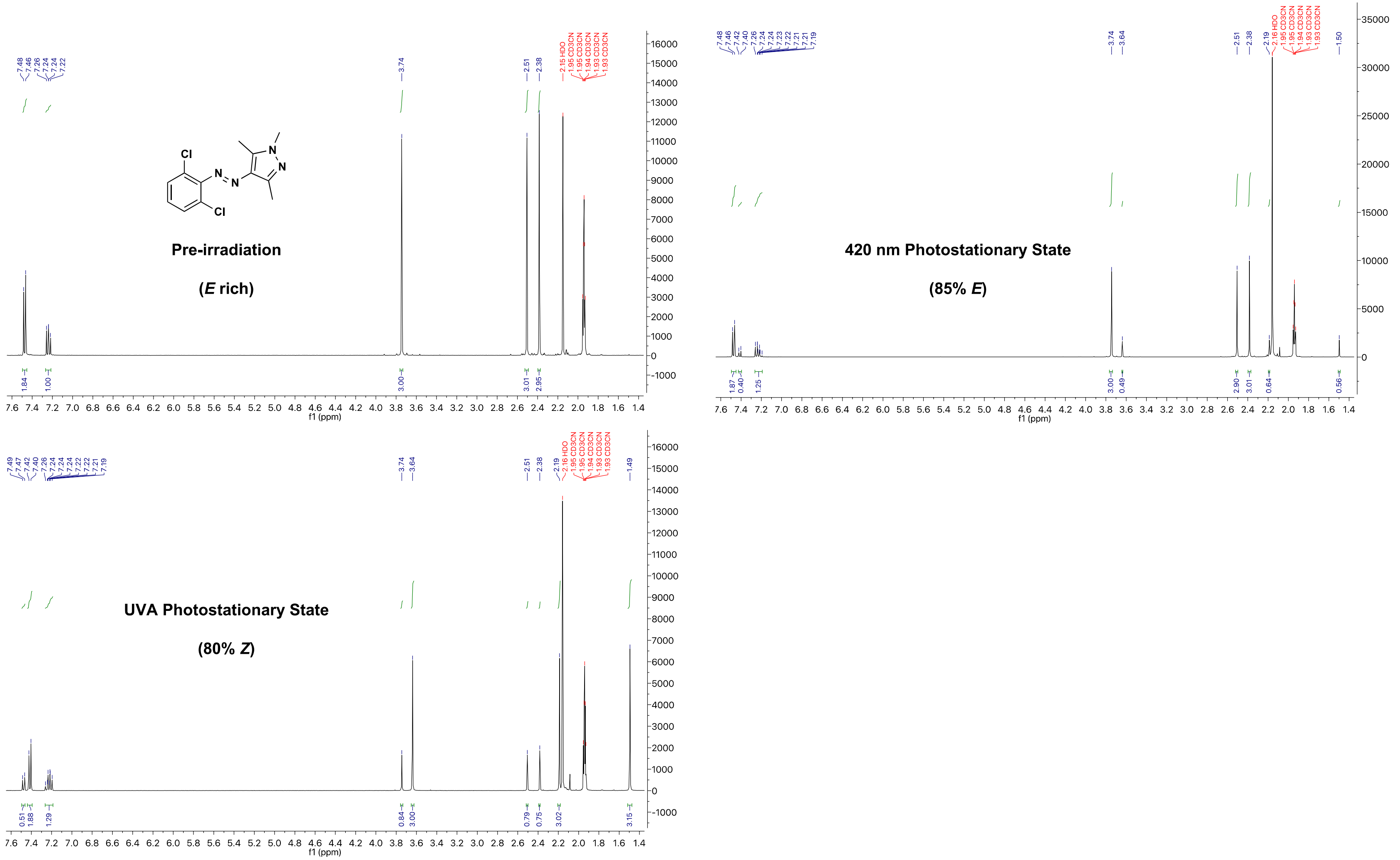

Figure S11. 4pzMe-Cl2 ^{1}H NMR spectra of the ***E***-rich state, UVA PSS and 420 nm PSS in MeCN-d_3 at ≈ 20 mM.

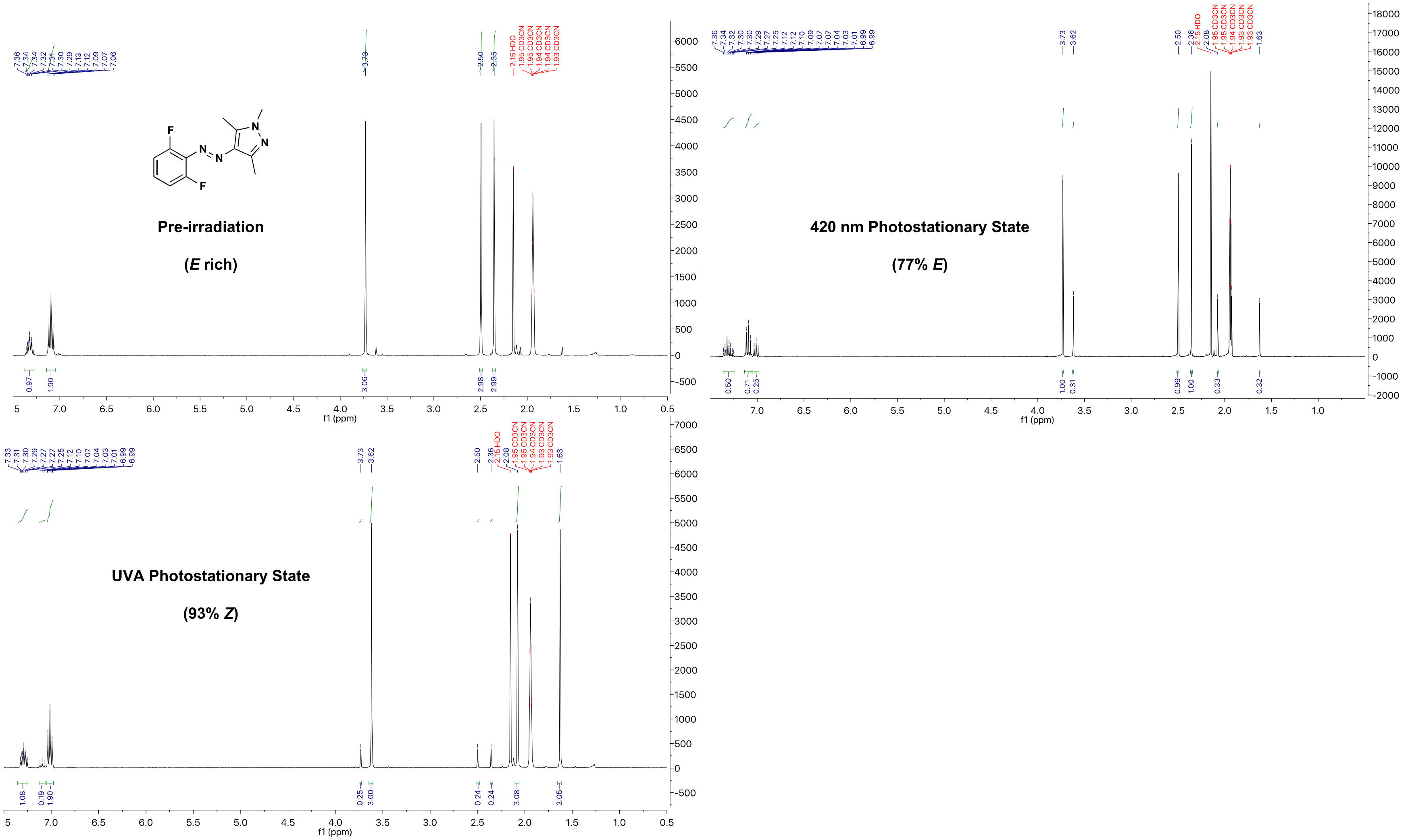

Figure S12. 4pzMe-F2 ^{1}H NMR spectra of the ***E***-rich state, UVA PSS and 420 nm PSS in MeCN-d_3 at ≈ 20 mM.

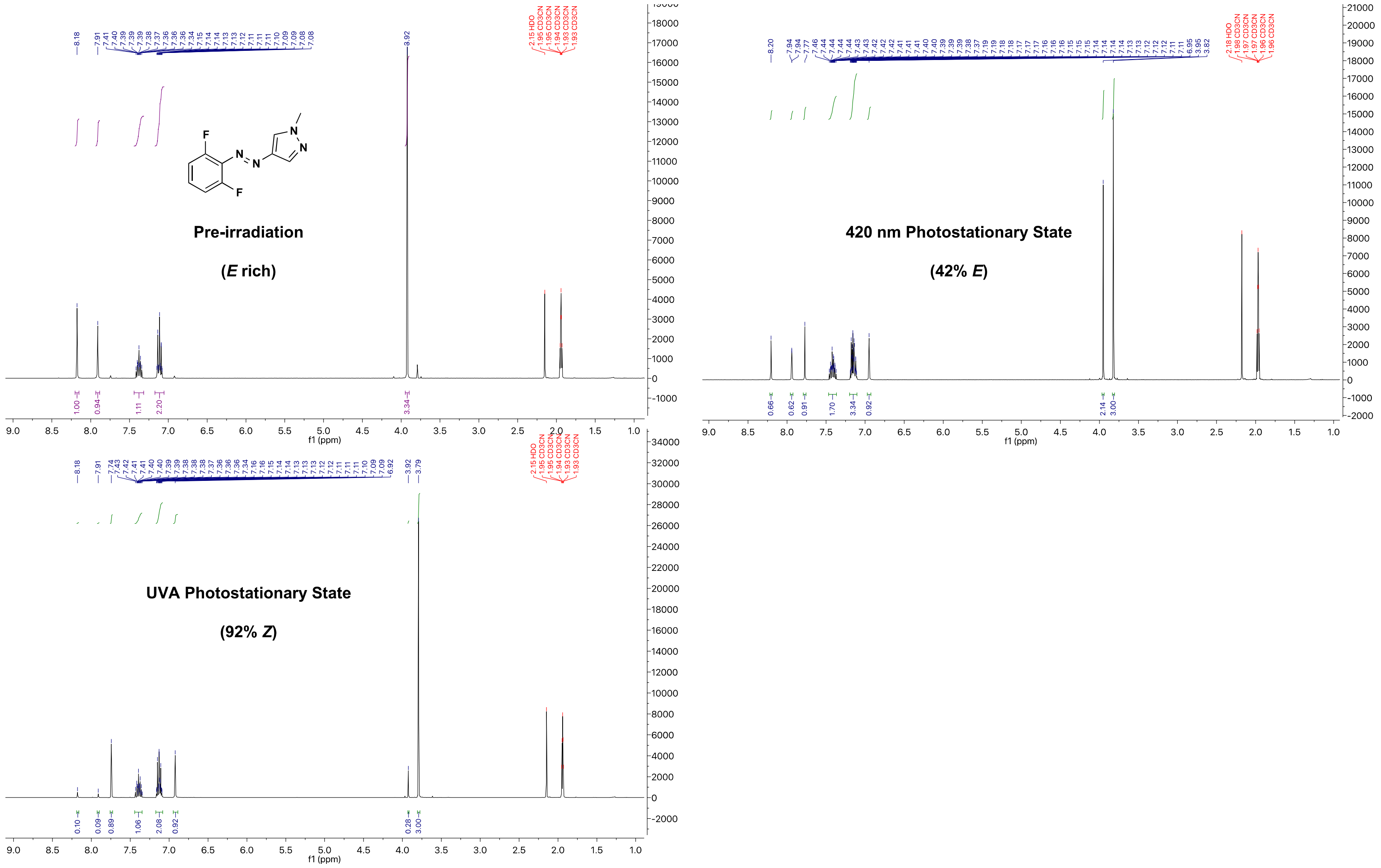

Figure S13. 4pzH-F2 ^{1}H NMR spectra of the ***E***-rich state, UVA PSS and 420 nm PSS in MeCN-d_3 at ≈ 20 mM.

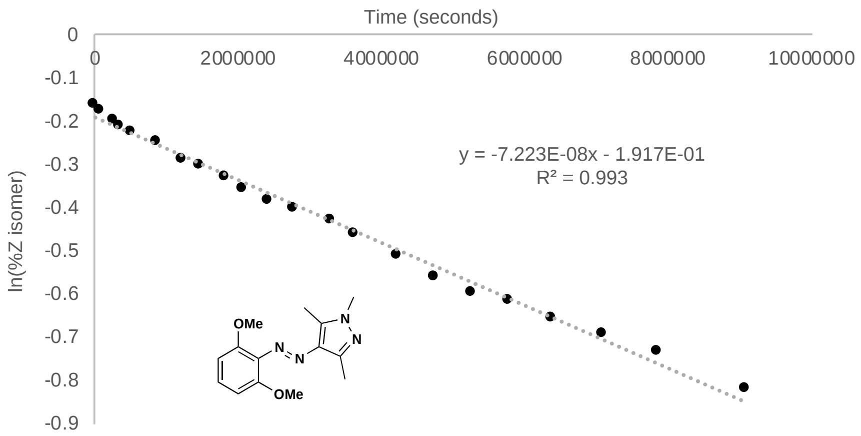

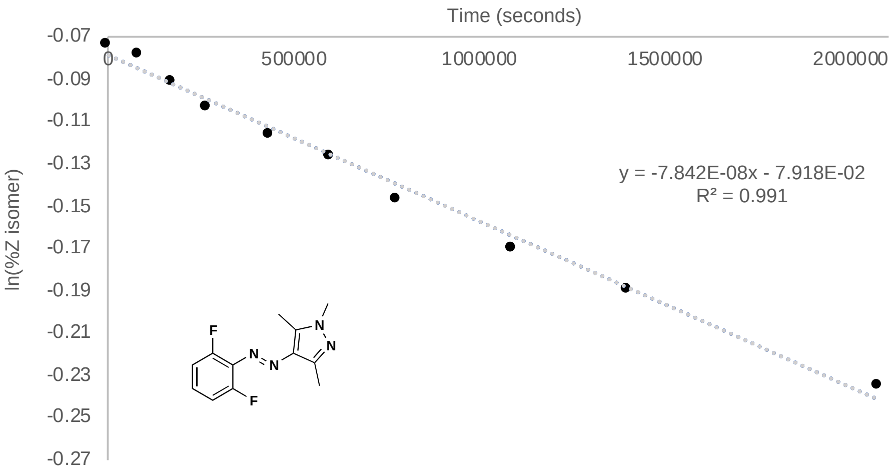

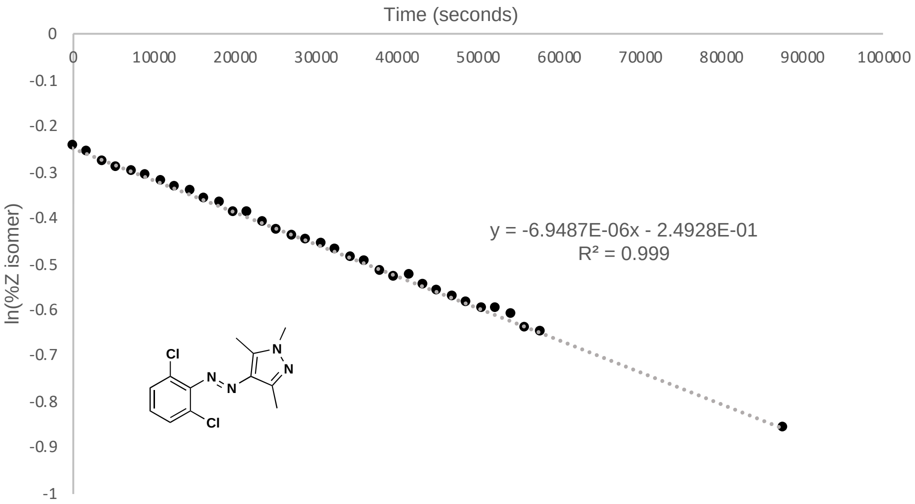

Figure S14. *Z*→*E* thermal isomerisation kinetics of **4pzMe-OMe2**, **4pzMe-F2** and **4pzMe-Cl2** in MeCN-d_3 at ≈ 20 mM.

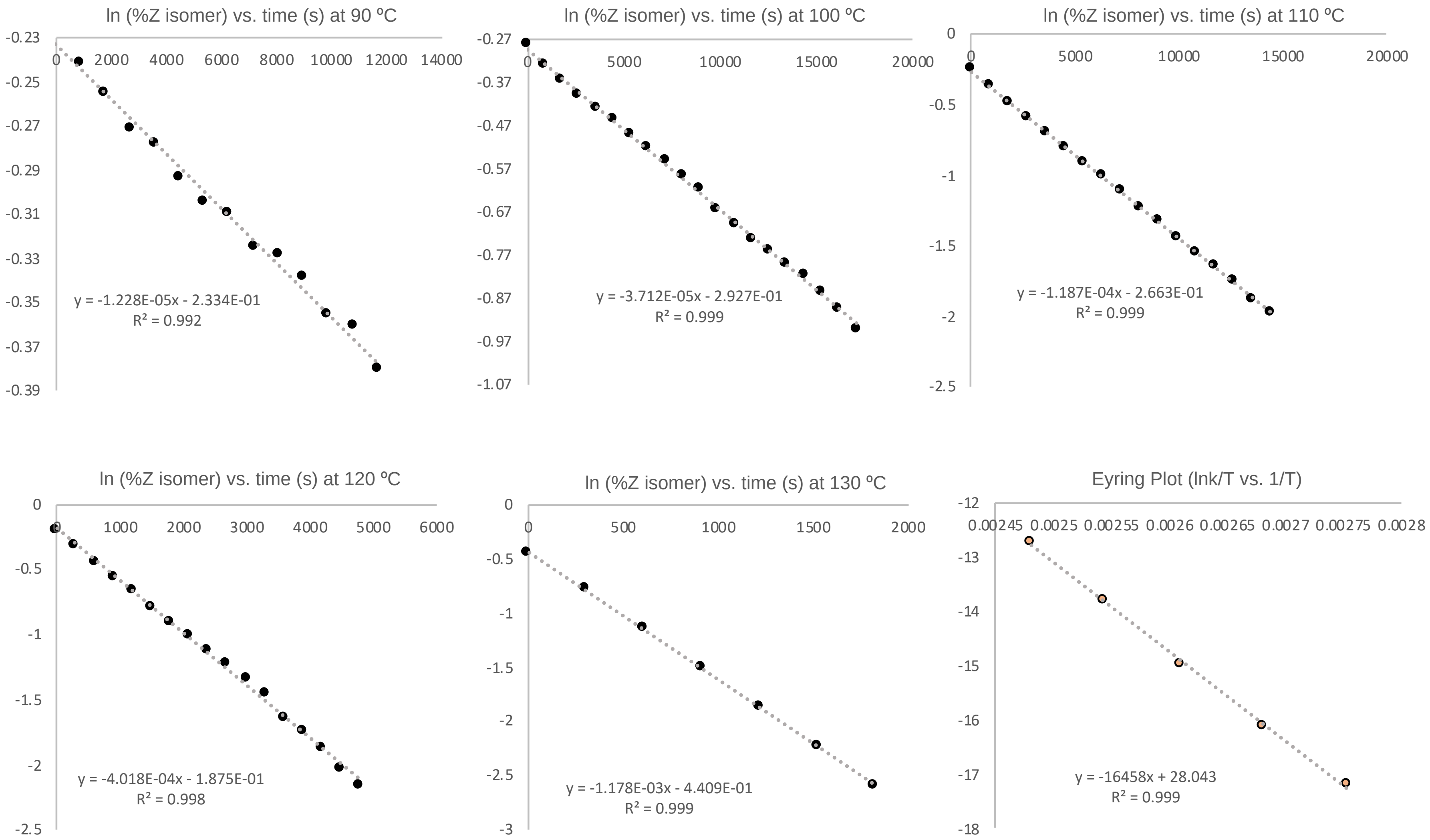

Figure S15. *Z*→*E* thermal isomerisation kinetics of **4pzH-F2** in DMSO-d_6 at ≈ 20 mM for a range of temperatures. An Eyring plot is also included for extrapolation to 25 ºC.

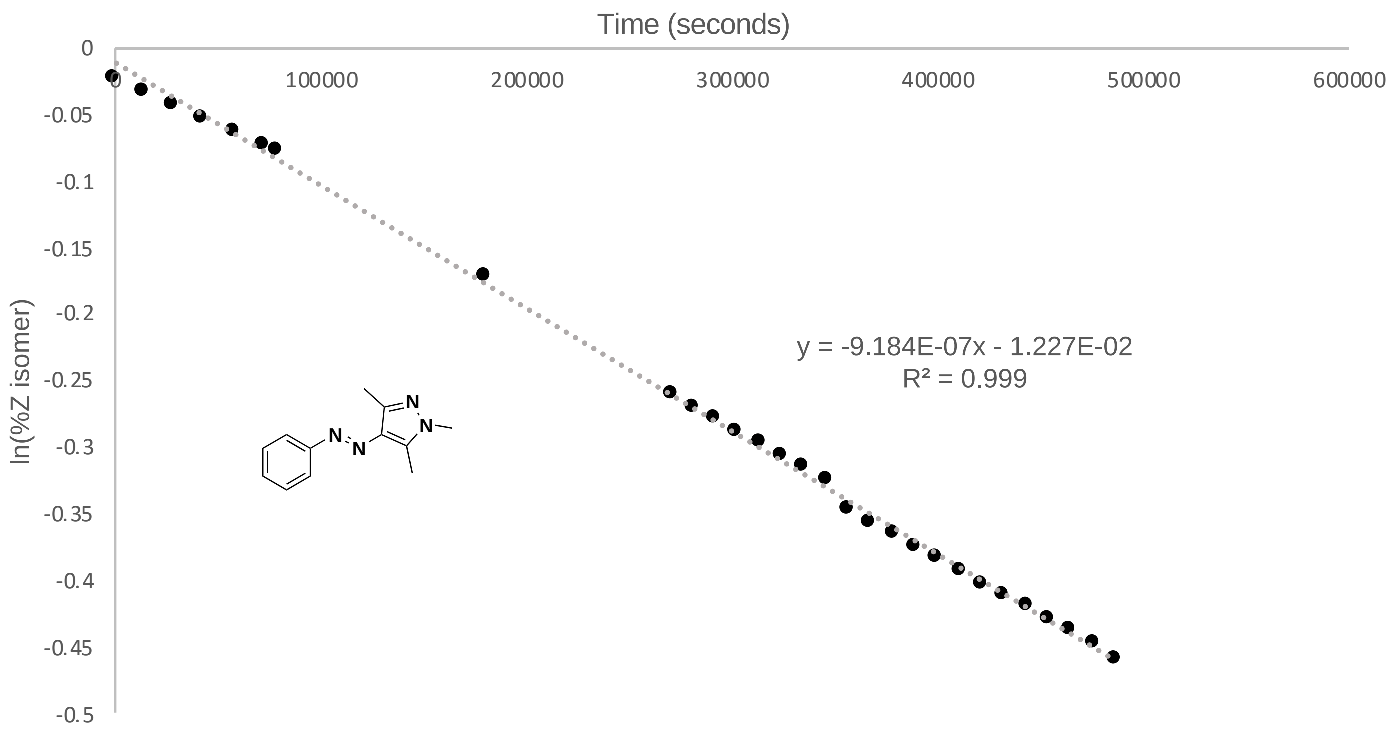

Figure S16 *Z*→*E* thermal isomerisation kinetics of **4pzMe** in DMSO-d_6 at ≈ 20 mM.

X-Ray crystallography

The X-ray crystal structure of 4pzH-F2

Crystal data for **4pzH-F2**: $C_{10}H_8F_2N_4$, M = 222.20, monoclinic, $P2_1/c$ (no. 14), a = 19.826(3), b = 3.9279(4), c = 13.2624(17) Å, β = 109.525(15)°, V = 973.4(2) Å^3, Z = 4, D_c = 1.516 g cm^{-3}, μ(Mo-Kα) = 0.124 mm^{-1}, T = 173 K, yellow tabular needles, Agilent Xcalibur 3 E diffractometer; 2044 independent measured reflections (R_{int} = 0.0207), F^2 refinement [4,5], R_1(obs) = 0.0403, wR_2(all) = 0.1004, 1675 independent observed absorption-corrected reflections [$|F_o| > 4\sigma(|F_o|)$, completeness to θ_{full}(25.2°) = 99.8%], 146 parameters. CCDC 1956314.

The X-ray crystal structure of 4pzMe-F2

Crystal data for **4pzMe-F2**: $C_{12}H_{12}F_2N_4$, M = 250.26, monoclinic, $P2_1/c$ (no. 14), a = 9.8817(7), b = 16.6285(11), c = 7.5309(5) Å, β = 103.152(7)°, V = 1205.00(15) Å^3, Z = 4, D_c = 1.379 g cm^{-3}, μ(Mo-Kα) = 0.109 mm^{-1}, T = 173 K, orange blocky needles, Agilent Xcalibur 3 E diffractometer; 2408 independent measured reflections (R_{int} = 0.0200), F^2 refinement [4,5], R_1(obs) = 0.0439, wR_2(all) = 0.1118, 1835 independent observed absorption-corrected reflections [$|F_o| > 4\sigma(|F_o|)$, completeness to θ_{full}(25.2°) = 99.0%], 167 parameters. CCDC 1941406.

The X-ray crystal structure of 4pzMe-OMe2

Crystal data for **4pzMe-OMe2**: $C_{14}H_{18}N_4O_2$, M = 274.32, monoclinic, $P2_1/c$ (no. 14), a = 7.0910(4), b = 20.3120(9), c = 10.3246(6) Å, β = 103.879(6)°, V = 1443.67(14) Å^3, Z = 4, D_c = 1.262 g cm^{-3}, μ(Mo-Kα) = 0.087 mm^{-1}, T = 173 K, yellow blocky needles, Agilent Xcalibur 3 E diffractometer; 2913 independent measured reflections (R_{int} =

0.0228), F^2 refinement [4,5], R_1(obs) = 0.0483, wR_2(all) = 0.1185, 2211 independent observed absorption-corrected reflections [$|F_o| > 4\sigma(|F_o|)$, completeness to θ_{full}(25.2°) = 99.0%], 186 parameters. CCDC 1941407.

The X-ray crystal structure of 4pzMe-OMe2_diethylether

Crystal data for **4pzMe-OMe2_diethylether**: $C_{14}H_{18}N_4O_2$, M = 274.32, monoclinic, $P2_1/c$ (no. 14), a = 7.0836(4), b = 20.2850(12), c = 10.3061(6) Å, β = 103.825(5)°, V = 1437.99(14) Å^3, Z = 4, D_c = 1.267 g cm^{-3}, μ(Mo-Kα) = 0.088 mm^{-1}, T = 173 K, yellow blocky needles, Agilent Xcalibur 3 E diffractometer; 2881 independent measured reflections (R_{int} = 0.0220), F^2 refinement [4,5], R_1(obs) = 0.0484, wR_2(all) = 0.1223, 2201 independent observed absorption-corrected reflections [$|F_o| > 4\sigma(|F_o|)$, completeness to θ_{full}(25.2°) = 98.7%], 187 parameters. CCDC 1941801.

Crystallization of **4pzMe-OMe2** from diethyl ether rather than ethyl acetate has led to an unchanged structure.

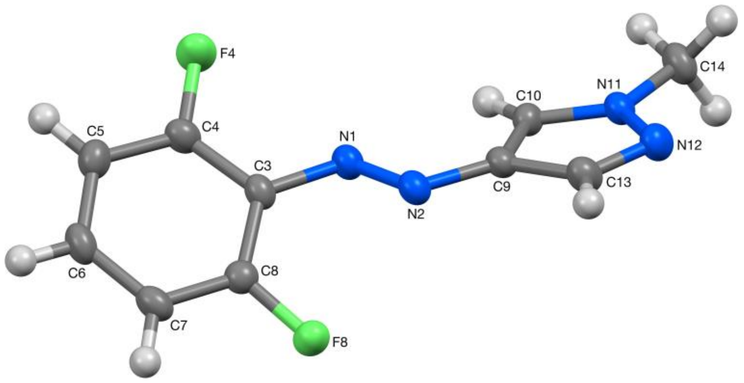

Figure S17. The crystal structure of **4pzH-F2** (50% probability ellipsoids).

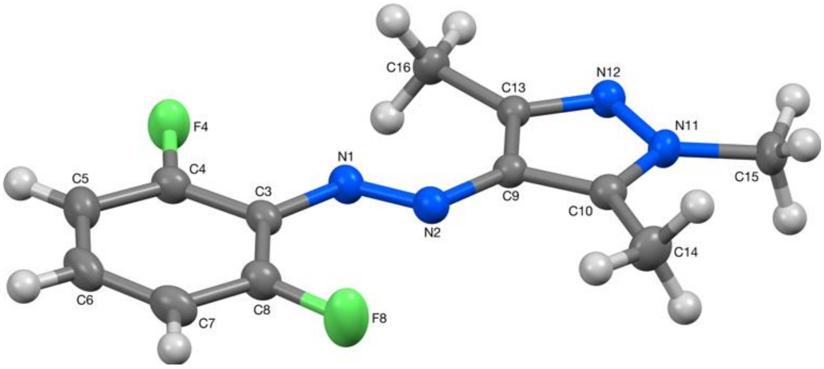

Figure S18. The crystal structure of **4pzMe-F2** (50% probability ellipsoids).

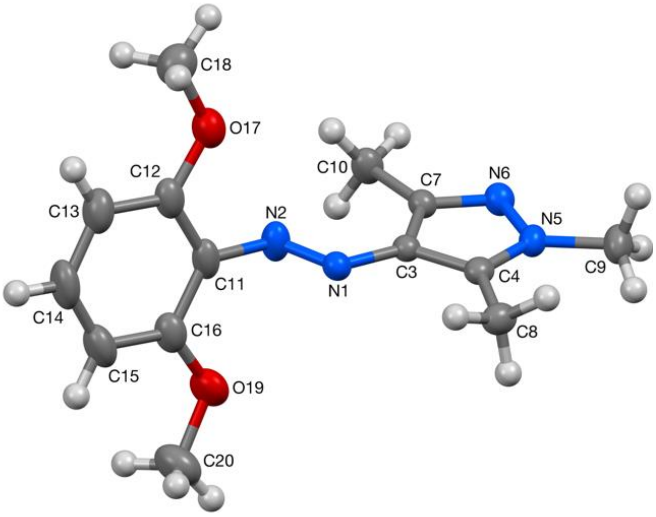

Figure S19. The crystal structure of **4pzMe-OMe2** (50% probability ellipsoids).

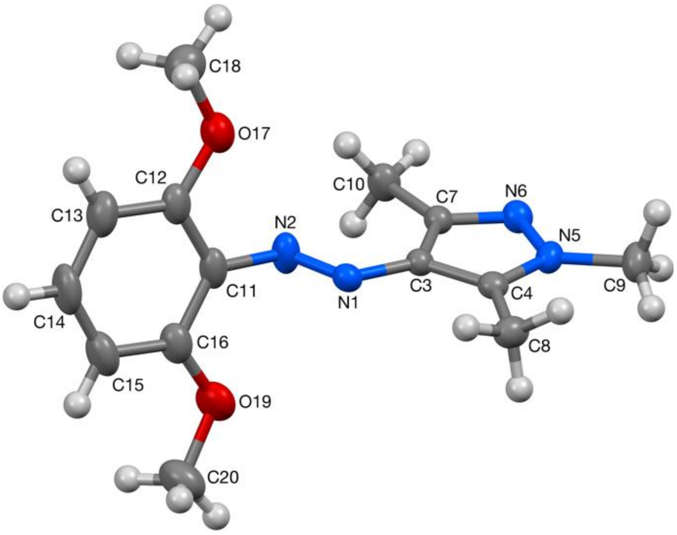

Figure S20. The crystal structure of **4pzMe-OMe2_diethylether** (50% probability ellipsoids).

Cartesian coordinates for all the optimized structures

```
24
4pzH-cis-m ; E = -605.348392323 Hartree
C       -1.882050       -0.345931       -1.210606
C       -2.609669        0.839330       -1.204569
C       -1.498487       -0.925379       -0.000308
H       -2.899926        1.294761       -2.146949
C       -2.967157        1.440902        0.000606
C       -1.881714       -0.346652        1.210447
H       -1.592351       -0.823717        2.141641
C       -2.609355        0.838598        1.205320
H       -2.899370        1.293449        2.148054
H       -1.592949       -0.822459       -2.142157
N       -0.778837       -2.153036       -0.000753
N        0.471489       -2.154533       -0.000570
C        1.258694       -0.999501       -0.000216
C        1.042663        0.386039       -0.000188
C        2.670409       -1.120861        0.000455
N        3.270877        0.054504        0.000818
N        2.260989        0.948617        0.000541
C        2.575873        2.356439       -0.000787
H        3.152414        2.605860       -0.894264
H        1.646795        2.926981        0.006830
H        3.165704        2.603615        0.884498
H        0.149380        0.989569       -0.000597
H        3.239996       -2.039995        0.000804
H       -3.536575        2.365166        0.000963
24
4pzH-cis-n ; E = -605.347923951 Hartree
C       -2.053256        0.312559        1.210337
C       -2.879167       -0.806213        1.204672
C       -1.622794        0.856583       -0.000050
H       -3.203642       -1.237259        2.147270
C       -3.288204       -1.374222        0.000134
C       -2.053027        0.312177       -1.210355
H       -1.724359        0.761891       -2.142096
C       -2.878923       -0.806600       -1.204511
H       -3.203251       -1.237943       -2.147023
H       -1.724838        0.762578        2.142016
N       -0.815698        2.029983       -0.000202
N        0.430766        1.948201       -0.000141
C        1.139001        0.740510       -0.000041
C        2.529644        0.802747        0.000010
C        0.861890       -0.661579       -0.000140
N        1.980662       -1.365193       -0.000154
N        2.978973       -0.457660       -0.000044
C        4.344546       -0.920264        0.000342
H        4.530393       -1.527250       -0.888416
H        5.011377       -0.057610       -0.000730
H        4.530696       -1.525282        0.890392
H        3.180207        1.664848        0.000034
H       -0.085740       -1.177307       -0.000247
H       -3.932010       -2.248185        0.000196
24
4pzH-trans-m ; E = -605.365368227 Hartree
C        2.652522       -1.347327        0.000392
C        4.039866       -1.260794        0.000279
```

C	1.875512	-0.187026	0.000065
H	4.639892	-2.165773	0.000613
C	4.655875	-0.012829	-0.000226
C	2.496247	1.069078	-0.000471
H	1.873372	1.956774	-0.000721
C	3.879851	1.148596	-0.000599
H	4.363580	2.121200	-0.000974
H	2.141296	-2.304989	0.000801
N	0.479149	-0.391081	0.000236
N	-0.198440	0.669437	0.000425
C	-1.567541	0.464908	0.000281
C	-2.316651	-0.709802	-0.000119
C	-2.533757	1.496361	0.000588
N	-3.764816	1.009933	0.000131
N	-3.604044	-0.328655	-0.000134
C	-4.775045	-1.170094	-0.000603
H	-5.375761	-0.968643	-0.890332
H	-4.461673	-2.214390	-0.000116
H	-5.376753	-0.968105	0.888324
H	-2.006553	-1.743034	-0.000374
H	-2.361118	2.563894	0.000949
H	5.739501	0.059208	-0.000308

24

4pzH-trans-n ; E = -605.365601144 Hartree

C	2.851801	1.236311	-0.000281
C	4.217150	0.975383	-0.000317
C	1.934828	0.183164	0.000006
H	4.926419	1.797532	-0.000619
C	4.671026	-0.340252	0.000012
C	2.392362	-1.141078	0.000356
H	1.662662	-1.943280	0.000578
C	3.754895	-1.394650	0.000348
H	4.112323	-2.420464	0.000589
H	2.464845	2.250522	-0.000550
N	0.575638	0.563024	0.000023
N	-0.231018	-0.402807	-0.000302
C	-1.561103	-0.019630	-0.000040
C	-2.618082	-0.920272	-0.000407
C	-2.184992	1.255032	0.000451
N	-3.501600	1.142243	0.000395
N	-3.743455	-0.185196	0.000005
C	-5.108284	-0.647651	-0.000164
H	-5.625721	-0.281276	-0.889768
H	-5.113355	-1.738105	0.000184
H	-5.626050	-0.280702	0.889004
H	-2.622829	-2.000031	-0.000832
H	-1.709813	2.225065	0.000789
H	5.736954	-0.548248	-0.000004

24

4pzHCl1A-cis-m ; E = -1064.78465959 Hartree

C	-1.784226	-0.314976	-0.487012
C	-2.470328	0.798685	-0.960625
C	-1.282505	-0.341888	0.816935
H	-2.844023	0.795608	-1.979070
C	-2.665124	1.896295	-0.128377
C	-1.506931	0.755408	1.650703
H	-1.130201	0.714532	2.667983
C	-2.186022	1.871835	1.179609

```
H       -2.345364        2.721427        1.836219
Cl       -1.512876       -1.666779       -1.539783
N       -0.647686       -1.498819        1.345802
N        0.588864       -1.652370        1.252987
C        1.447330       -0.751057        0.626101
C        1.325995        0.481720       -0.031707
C        2.834236       -1.033983        0.561686
N        3.504942       -0.086958       -0.066113
N        2.566675        0.817783       -0.413985
C        2.972311        2.000288       -1.134369
H        3.434367        1.714386       -2.081676
H        2.093987        2.616902       -1.326638
H        3.694736        2.564210       -0.540494
H        0.484260        1.119698       -0.246977
H        3.335447       -1.905510        0.959458
H       -3.202294        2.762576       -0.500981
24
4pzHCl1A-cis-n ; E = -1064.78460406 Hartree
C        1.875732       -0.557955        0.330287
C        2.695237        0.158929        1.195332
C        1.403068        0.022583       -0.849331
H        3.040351       -0.312651        2.109179
C        3.053825        1.465946        0.882998
C        1.792369        1.325088       -1.165234
H        1.436770        1.758021       -2.095016
C        2.603807        2.047545       -0.299828
H        2.887243        3.064705       -0.551279
Cl        1.407159       -2.178246        0.740004
N        0.637779       -0.721059       -1.790546
N       -0.605898       -0.787711       -1.702967
C       -1.356329       -0.164116       -0.704983
C       -2.732733       -0.373228       -0.692254
C       -1.134505        0.711437        0.402452
N       -2.269778        0.994764        1.015376
N       -3.224761        0.324560        0.337134
C       -4.593487        0.417901        0.781622
H       -4.904095        1.464538        0.796373
H       -5.228389       -0.142566        0.094979
H       -4.687413        0.003263        1.787831
H       -3.344224       -0.971970       -1.350928
H       -0.216655        1.144977        0.767767
H        3.691404        2.023201        1.561675
24
4pzHCl1A-trans-m ; E = -1064.80116010 Hartree
C        2.479229       -0.700889        0.000050
C        3.849626       -0.459842       -0.000050
C        1.561033        0.359315       -0.000013
H        4.535227       -1.299880        0.000005
C        4.318338        0.847653       -0.000252
C        2.057091        1.670430       -0.000224
H        1.331706        2.476892       -0.000285
C        3.419150        1.914870       -0.000346
H        3.786064        2.936567       -0.000531
Cl        1.927198       -2.345592        0.000266
N        0.192422        0.035431        0.000127
N       -0.574792        1.033313        0.000438
C       -1.917174        0.704675        0.000186
C       -2.548857       -0.538131       -0.000378
```

```
C       -2.978160        1.638845        0.000304
N       -4.155480        1.035104        0.000435
N       -3.865532       -0.282727       -0.000133
C       -4.950224       -1.233392       -0.000523
H       -5.567366       -1.089777       -0.890114
H       -4.537320       -2.242375       -0.000418
H       -5.567905       -1.089803        0.888691
H       -2.137274       -1.535441       -0.000770
H       -2.910378        2.717960        0.000616
H        5.388235        1.031854       -0.000317
24
4pzHCl1A-trans-n ; E = -1064.80090683 Hartree
C       -2.618272       -0.555208       -0.000023
C       -3.942204       -0.125984       -0.000052
C       -1.562412        0.368599        0.000027
H       -4.737408       -0.863161       -0.000095
C       -4.225827        1.233593       -0.000012
C       -1.872779        1.735626        0.000077
H       -1.043049        2.434253        0.000132
C       -3.187789        2.166390        0.000058
H       -3.410049        3.229026        0.000109
Cl       -2.299187       -2.259066       -0.000067
N       -0.251635       -0.141439        0.000044
N        0.644820        0.742080       -0.000023
C        1.927623        0.228484       -0.000008
C        3.071351        1.017302       -0.000090
C        2.417115       -1.104200        0.000178
N        3.737654       -1.125654        0.000160
N        4.114354        0.171097        0.000026
C        5.519912        0.490200       -0.000146
H        5.996052        0.071888       -0.889815
H        5.637699        1.574243        0.000320
H        5.996380        0.071084        0.888961
H        3.186500        2.090901       -0.000219
H        1.843397       -2.019393        0.000302
H       -5.260020        1.563877       -0.000044
24
4pzHCl1B-cis-m ; E = -1064.78465960 Hartree
C       -1.505810        0.755838       -1.650777
C       -2.184374        1.872647       -1.179748
C       -1.282422       -0.341686       -0.817049
H       -2.342960        2.722430       -1.836295
C       -2.663869        1.897273        0.128066
C       -1.784616       -0.314588        0.486687
Cl       -1.514481       -1.666823        1.539417
C       -2.470100        0.799422        0.960300
H       -2.844103        0.796478        1.978621
H       -1.128719        0.714868       -2.667918
N       -0.647926       -1.498928       -1.345772
N        0.588634       -1.652548       -1.252834
C        1.447085       -0.751280       -0.625837
C        1.326014        0.481373        0.032177
C        2.833990       -1.034430       -0.561582
N        3.504917       -0.087645        0.066304
N        2.566763        0.817168        0.414479
C        2.972754        1.999626        1.134715
H        3.693603        2.564555        0.539863
H        2.094269        2.615387        1.329000
```

H	3.436795	1.713520	2.080978
H	0.484351	1.119445	0.247658
H	3.334861	-1.906099	-0.959537
H	-3.200535	2.763903	0.500591

24

4pzHCl1B-cis-n ; E = -1064.78460407 Hartree

C	-1.792413	1.324891	-1.165414
C	-2.603676	2.047510	-0.299958
C	-1.403099	0.022434	-0.849363
H	-2.887093	3.064651	-0.551519
C	-3.053513	1.466120	0.883020
C	-1.875609	-0.557901	0.330432
Cl	-1.407045	-2.178300	0.740285
C	-2.694951	0.159116	1.195497
H	-3.039963	-0.312325	2.109452
H	-1.436967	1.757711	-2.095302
N	-0.637867	-0.721378	-1.790508
N	0.605808	-0.788048	-1.702879
C	1.356199	-0.164256	-0.705010
C	2.732570	-0.373581	-0.692018
C	1.134446	0.712025	0.401877
N	2.269674	0.995454	1.014800
N	3.224615	0.324569	0.337089
C	4.593272	0.417845	0.781774
H	4.686873	0.003893	1.788294
H	5.228183	-0.143272	0.095664
H	4.904151	1.464411	0.795849
H	3.343966	-0.972831	-1.350313
H	0.216631	1.145960	0.766798
H	-3.690970	2.023507	1.561703

24

4pzHCl1B-trans-m ; E = -1064.79441682 Hartree

C	-2.226575	1.839027	0.122927
C	-3.605701	1.934387	0.212002
C	-1.578960	0.603895	-0.006731
H	-4.077582	2.904109	0.333080
C	-4.376619	0.777991	0.130064
C	-2.372916	-0.553111	-0.109835
Cl	-1.705359	-2.126682	-0.406136
C	-3.760483	-0.455249	-0.044849
H	-4.350644	-1.360603	-0.137627
H	-1.593527	2.719745	0.166225
N	-0.175572	0.693309	-0.087876
N	0.449098	-0.286122	0.392843
C	1.824758	-0.186782	0.278805
C	2.628922	0.789275	-0.306628
C	2.738320	-1.142950	0.777194
N	3.989948	-0.794420	0.527124
N	3.894378	0.379658	-0.130579
C	5.104671	1.036563	-0.559173
H	5.655489	0.387741	-1.243701
H	4.843826	1.965802	-1.066287
H	5.732931	1.255554	0.306963
H	2.370295	1.704904	-0.815371
H	2.512240	-2.060346	1.302645
H	-5.459388	0.831444	0.185154

24

4pzHCl1B-trans-n ; E = -1064.79503623 Hartree

```
C         2.458437        1.738206       -0.093570
C         3.831529        1.671111       -0.264339
C         1.673523        0.585366        0.036154
H         4.410234        2.581512       -0.382447
C         4.459375        0.428562       -0.267113
C         2.325603       -0.661218        0.052608
Cl         1.490388       -2.155647        0.338622
C         3.708637       -0.728078       -0.094306
H         4.188577       -1.700427       -0.066591
H         1.935565        2.689086       -0.068319
N         0.298890        0.841255        0.205465
N        -0.467492       -0.039704       -0.260202
C        -1.810186        0.227811       -0.060611
C        -2.825238       -0.604862       -0.513531
C        -2.489047        1.293766        0.585040
N        -3.798342        1.126858        0.530121
N        -3.980546       -0.030813       -0.140627
C        -5.323401       -0.502309       -0.369008
H        -5.883166        0.235764       -0.947854
H        -5.280218       -1.441582       -0.921142
H        -5.828429       -0.663501        0.586046
H        -2.778673       -1.536737       -1.057033
H        -2.059166        2.154411        1.075994
H         5.535720        0.354162       -0.386477
24
4pzHCl2-cis-m ; E = -1524.21983515 Hartree
C        -1.564851       -1.198326       -0.079181
C        -2.272919       -1.206711       -1.275136
C        -1.188916        0.000054        0.537176
H        -2.542378       -2.155285       -1.725966
C        -2.623860       -0.000322       -1.871112
C        -1.564942        1.198229       -0.079488
Cl        -1.104570        2.700598        0.651619
C        -2.273013        1.206252       -1.275443
H        -2.542559        2.154671       -1.726546
Cl        -1.104349       -2.700460        0.652260
N        -0.578397        0.000190        1.819840
N         0.662931        0.000254        1.952250
C         1.564234        0.000171        0.894234
C         1.482408        0.000076       -0.506060
C         2.957323        0.000296        1.153154
N         3.666760        0.000285        0.041170
N         2.747980        0.000199       -0.947098
C         3.200142       -0.000673       -2.317476
H         3.801575       -0.892415       -2.506028
H         2.332411        0.003024       -2.977452
H         3.808070        0.886915       -2.504567
H         0.652405       -0.000076       -1.193761
H         3.434976        0.000487        2.123075
H        -3.181440       -0.000437       -2.801846
24
4pzHCl2-cis-n ; E = -1524.22011144 Hartree
C         1.703410       -1.198416        0.052264
C         2.531352       -1.207649        1.168087
C         1.265434       -0.000140       -0.520308
H         2.842707       -2.156281        1.590688
C         2.942768       -0.001602        1.724174
C         1.704996        1.197360        0.052630
```

```
Cl        1.173458        2.700341       -0.629038
C         2.532902        1.205160        1.168500
H         2.845464        2.153241        1.591445
Cl        1.170023       -2.700522       -0.629881
N         0.528327        0.000511       -1.735765
N        -0.719181        0.000821       -1.743558
C        -1.508724        0.000707       -0.597239
C        -2.893063        0.000746       -0.748187
C        -1.319362        0.000739        0.819156
N        -2.479283        0.000762        1.449751
N        -3.419133        0.000745        0.480642
C        -4.811251        0.000611        0.858118
H        -5.032874        0.889997        1.451734
H        -5.423160        0.001100       -0.044172
H        -5.033004       -0.889334        1.450856
H        -3.487181        0.000756       -1.649973
H        -0.407745        0.000768        1.396065
H         3.590732       -0.002148        2.594309
24
4pzHCl2-trans-m ; E = -1524.22919333 Hartree
C        -2.231681        1.223758        0.044216
C        -3.616174        1.156148        0.095017
C        -1.430266        0.075239       -0.054405
H        -4.191662        2.069467        0.192566
C        -4.236575       -0.084985        0.018452
C        -2.089189       -1.163679       -0.137343
Cl       -1.209849       -2.640081       -0.364977
C        -3.477832       -1.241160       -0.106729
H        -3.951428       -2.213173       -0.185248
Cl       -1.463253        2.776284        0.148704
N        -0.042431        0.267273       -0.179616
N         0.654075       -0.508563        0.521223
C         2.016782       -0.340496        0.357232
C         2.737666        0.543059       -0.444660
C         3.004637       -1.090292        1.034899
N         4.222617       -0.710684        0.686673
N         4.030776        0.280660       -0.209835
C         5.183890        0.922751       -0.792199
H         5.786473        0.185116       -1.326500
H         4.848105        1.693662       -1.486062
H         5.790264        1.376132       -0.005175
H         2.402382        1.305944       -1.130286
H         2.855012       -1.884386        1.753106
H        -5.319204       -0.151080        0.048247
24
4pzHCl2-trans-n ; E = -1524.22941741 Hartree
C         2.399324        1.082976       -0.075032
C         3.756514        0.850902       -0.243760
C         1.474461        0.035912        0.065344
H         4.429508        1.691430       -0.368140
C         4.224979       -0.457414       -0.247732
C         1.981724       -1.274848        0.063218
Cl        0.949402       -2.642584        0.329865
C         3.343262       -1.517854       -0.085888
H         3.699236       -2.541722       -0.069461
Cl        1.819868        2.717959       -0.079902
N         0.134739        0.386754        0.312763
N        -0.702504       -0.270756       -0.353954
```

```
C       -2.016681        0.049871       -0.068961
C       -3.098735       -0.556120       -0.694789
C       -2.600533        0.979843        0.830358
N       -3.918356        0.943815        0.759398
N       -4.199259        0.004811       -0.171059
C       -5.578134       -0.278201       -0.482680
H       -6.067874        0.625616       -0.851746
H       -5.616751       -1.052836       -1.249045
H       -6.097651       -0.624648        0.413457
H       -3.132081       -1.326072       -1.451120
H       -2.096932        1.655895        1.505667
H        5.285523       -0.651806       -0.369478
24
4pzHF1A-cis-m ; E = -704.489806843 Hartree
C       -1.618915       -0.268511       -0.929200
C       -2.366425        0.876757       -1.139204
C       -1.416494       -0.789089        0.349940
H       -2.489471        1.244522       -2.152377
C       -2.934712        1.526414       -0.044982
C       -2.018152       -0.147806        1.431201
H       -1.881154       -0.569205        2.422230
C       -2.760336        1.014165        1.237916
H       -3.211378        1.512079        2.090301
F       -1.043781       -0.881993       -1.970051
N       -0.717125       -2.015032        0.535597
N        0.529164       -2.059720        0.453752
C        1.343404       -0.946713        0.246856
C        1.178474        0.444781        0.270246
C        2.730417       -1.119289        0.016407
N        3.360082        0.034315       -0.105646
N        2.397918        0.964111        0.061007
C        2.758761        2.359636        0.000312
H        3.182879        2.588857       -0.979642
H        1.866031        2.964057        0.162867
H        3.499570        2.583188        0.770960
H        0.321648        1.078320        0.434399
H        3.260755       -2.057774       -0.067175
H       -3.521255        2.426110       -0.201019
24
4pzHF1A-cis-n ; E = -704.490084156 Hartree
C        1.627235        0.064769        0.967894
C        2.478239        1.155228        0.974319
C        1.546948       -0.799412       -0.125878
H        2.497174        1.800386        1.846132
C        3.277954        1.398182       -0.140113
C        2.381576       -0.564561       -1.217567
H        2.341204       -1.255897       -2.053514
C        3.227701        0.539765       -1.235196
H        3.856051        0.722326       -2.100982
F        0.839267       -0.162082        2.026846
N        0.756559       -1.982065       -0.077930
N       -0.491475       -1.931787       -0.050873
C       -1.229353       -0.752992       -0.173046
C       -2.595758       -0.783706        0.085714
C       -0.996108        0.583217       -0.616517
N       -2.115036        1.286011       -0.614268
N       -3.069941        0.441356       -0.174505
C       -4.415876        0.931344       -0.008826
```

```
H       -4.734213        1.428091       -0.926935
H       -5.077187        0.090008        0.200447
H       -4.460447        1.645070        0.817703
H       -3.214380       -1.596816        0.435461
H       -0.081688        1.045004       -0.956345
H        3.943982        2.254970       -0.145265
24
4pzHF1A-trans-m ; E = -704.507068932 Hartree
C       -2.559657        1.027647        0.000121
C       -3.939131        0.887783        0.000050
C       -1.703904       -0.079762       -0.000015
H       -4.556897        1.779082        0.000185
C       -4.487448       -0.389167       -0.000187
C       -2.280076       -1.357426       -0.000264
H       -1.609484       -2.209831       -0.000390
C       -3.656631       -1.511846       -0.000346
H       -4.089606       -2.507200       -0.000542
F       -2.038957        2.256660        0.000362
N       -0.323462        0.188307        0.000058
N        0.396270       -0.845320        0.000328
C        1.754069       -0.581756        0.000165
C        2.448656        0.626488       -0.000232
C        2.766215       -1.568420        0.000374
N        3.973371       -1.026083        0.000258
N        3.751381        0.304320       -0.000101
C        4.882932        1.198327       -0.000441
H        5.492127        1.024070       -0.890101
H        4.522125        2.227143       -0.000078
H        5.492863        1.023698        0.888634
H        2.090073        1.644158       -0.000537
H        2.642863       -2.642588        0.000688
H       -5.566517       -0.508247       -0.000257
24
4pzHF1A-trans-n ; E = -704.506971171 Hartree
C        2.730435        0.902208       -0.000064
C        4.079637        0.581733       -0.000051
C        1.736597       -0.083328        0.000001
H        4.809038        1.384222       -0.000101
C        4.455518       -0.756103        0.000024
C        2.140224       -1.425529        0.000077
H        1.363552       -2.182528        0.000129
C        3.484454       -1.759945        0.000084
H        3.782976       -2.803577        0.000141
F        2.376596        2.188162       -0.000118
N        0.403328        0.364086        0.000019
N       -0.444885       -0.566738       -0.000106
C       -1.754787       -0.123162        0.000002
C       -2.852799       -0.973850       -0.000047
C       -2.318186        1.179674        0.000193
N       -3.638326        1.128046        0.000177
N       -3.942106       -0.187033        0.000032
C       -5.327366       -0.584548       -0.000153
H       -5.826637       -0.194085       -0.889856
H       -5.384009       -1.673531        0.000420
H       -5.827056       -0.193127        0.888886
H       -2.907975       -2.052243       -0.000173
H       -1.797298        2.125883        0.000316
H        5.509606       -1.015902        0.000045
```

24
4pzHF1B-cis-m ; E = -704.489806906 Hartree

C	-2.016656	-0.145788	-1.431857
C	-2.758468	1.016276	-1.237756
C	-1.416383	-0.788732	-0.350806
H	-3.208505	1.515551	-2.089880
C	-2.933762	1.527039	0.045620
C	-1.619769	-0.269675	0.928800
F	-1.045932	-0.884794	1.969458
C	-2.366842	0.875755	1.139564
H	-2.490566	1.242308	2.153088
H	-1.878914	-0.565949	-2.423304
N	-0.717230	-2.014632	-0.537241
N	0.529037	-2.059444	-0.455420
C	1.343296	-0.946565	-0.247414
C	1.178400	0.444949	-0.269485
C	2.730210	-1.119425	-0.016711
N	3.359871	0.034088	0.106817
N	2.397766	0.964001	-0.059049
C	2.758950	2.359494	0.000819
H	3.491098	2.585639	-0.777398
H	1.864265	2.964045	-0.150050
H	3.193859	2.585895	0.976622
H	0.321591	1.078668	-0.432969
H	3.260592	-2.057923	0.066299
H	-3.519995	2.426867	0.202078

24
4pzHF1B-cis-n ; E = -704.490084127 Hartree

C	-2.381598	-0.564910	-1.217352
C	-3.227753	0.539390	-1.235232
C	-1.546925	-0.799471	-0.125629
H	-3.856114	0.721659	-2.101073
C	-3.278000	1.398080	-0.140364
C	-1.627122	0.065042	0.967896
F	-0.839103	-0.161465	2.026738
C	-2.478175	1.155462	0.974067
H	-2.497090	1.800870	1.845700
H	-2.341253	-1.256450	-2.053134
N	-0.756507	-1.982074	-0.077375
N	0.491532	-1.931806	-0.050525
C	1.229341	-0.753088	-0.173055
C	2.595736	-0.783741	0.085706
C	0.996089	0.583110	-0.616683
N	2.114958	1.285963	-0.614455
N	3.069868	0.441281	-0.174623
C	4.415758	0.931364	-0.008924
H	4.460391	1.644636	0.817995
H	5.077234	0.090005	0.199744
H	4.733790	1.428664	-0.926835
H	3.214332	-1.596872	0.435481
H	0.081634	1.044866	-0.956629
H	-3.944060	2.254839	-0.145625

24
4pzHF1B-trans-m ; E = -704.503169403 Hartree

C	-2.401749	1.575520	0.000529
C	-3.787166	1.627979	0.000763
C	-1.704932	0.359420	-0.000040
H	-4.296240	2.586141	0.001308

```
C       -4.516689        0.441479        0.000268
C       -2.469446       -0.821925       -0.000555
F       -1.887631       -2.019287       -0.001357
C       -3.854980       -0.782255       -0.000433
H       -4.394563       -1.723396       -0.000888
H       -1.799228        2.478319        0.000832
N       -0.307050        0.492331       -0.000261
N        0.351845       -0.582170        0.000631
C        1.722255       -0.387772        0.000497
C        2.484655        0.778832       -0.000575
C        2.677074       -1.430102        0.001499
N        3.913257       -0.957455        0.001031
N        3.767445        0.383333       -0.000202
C        4.948152        1.211038       -0.001099
H        5.546643        1.001827       -0.890549
H        4.647151        2.258981       -0.001410
H        5.547378        1.002559        0.888019
H        2.187188        1.815766       -0.001523
H        2.490845       -2.495052        0.002492
H       -5.602016        0.462864        0.000400
24
4pzHF1B-trans-n ; E = -704.503728670 Hartree
C        2.633390        1.465419       -0.000554
C        4.013659        1.335107       -0.000983
C        1.782848        0.351379        0.000086
H        4.644197        2.218084       -0.001591
C        4.581021        0.063102       -0.000594
C        2.385600       -0.920123        0.000525
F        1.651097       -2.031442        0.001462
C        3.764349       -1.063178        0.000218
H        4.175641       -2.067057        0.000627
H        2.154303        2.439315       -0.000740
N        0.414873        0.668198        0.000459
N       -0.379721       -0.310072       -0.000765
C       -1.710702        0.069179       -0.000172
C       -2.760602       -0.840038       -0.001291
C       -2.344972        1.338865        0.001483
N       -3.660624        1.215430        0.001362
N       -3.891430       -0.114282       -0.000275
C       -5.252527       -0.587835       -0.000887
H       -5.772788       -0.224737       -0.890172
H       -5.248468       -1.678255       -0.001576
H       -5.773108       -0.225857        0.888658
H       -2.754181       -1.919832       -0.002629
H       -1.879337        2.313253        0.002702
H        5.659794       -0.058106       -0.000893
24
4pzHF2-cis-m ; E = -803.630444827 Hartree
C       -2.070201       -0.356790       -0.951910
C       -2.815924        0.809126       -1.038839
C       -1.301632       -0.668086        0.168066
H       -3.380567        1.007470       -1.942724
C       -2.826116        1.682335        0.044510
C       -1.351065        0.227320        1.237947
F       -0.626558       -0.054127        2.325034
C       -2.093589        1.395096        1.193927
H       -2.090768        2.054825        2.054188
F       -2.047648       -1.203006       -1.984252
```

N	-0.656884	-1.933337	0.275398
N	0.588678	-2.024422	0.272440
C	1.459694	-0.956898	0.074229
C	1.344380	0.375940	-0.342852
C	2.855664	-1.144931	0.225126
N	3.535905	-0.048893	-0.053424
N	2.596518	0.853310	-0.402161
C	3.013665	2.178922	-0.790418
H	3.666530	2.119477	-1.663874
H	2.131393	2.772356	-1.031386
H	3.558332	2.648464	0.031351
H	0.499454	0.990494	-0.608280
H	3.356843	-2.051578	0.534529
H	-3.412158	2.593879	-0.005325

24

4pzHF2-cis-n ; E = -803.631039952 Hartree

C	2.331504	-0.799197	0.514605
C	3.179329	0.121998	1.108907
C	1.394749	-0.437295	-0.452698
H	3.870598	-0.214079	1.873210
C	3.122369	1.449908	0.697042
C	1.382114	0.902267	-0.847369
F	0.505825	1.272365	-1.787050
C	2.223379	1.848770	-0.288634
H	2.162748	2.875547	-0.630820
F	2.374989	-2.077615	0.897386
N	0.657525	-1.440314	-1.141921
N	-0.590341	-1.472935	-1.109365
C	-1.379976	-0.641241	-0.317686
C	-2.759338	-0.652206	-0.500662
C	-1.189835	0.240768	0.788088
N	-2.344181	0.716533	1.219080
N	-3.281349	0.170055	0.417413
C	-4.664077	0.531637	0.609996
H	-4.817202	1.584045	0.358852
H	-5.286673	-0.090997	-0.033046
H	-4.938601	0.369841	1.653882
H	-3.355193	-1.190330	-1.222805
H	-0.280111	0.525911	1.293553
H	3.784211	2.181478	1.148165

24

4pzHF2-trans-m ; E = -803.643917587 Hartree

C	-2.345366	1.279608	0.000231
C	-3.728974	1.287039	0.000384
C	-1.578415	0.100515	-0.000112
H	-4.251147	2.236591	0.000717
C	-4.401508	0.070181	0.000113
C	-2.308718	-1.104621	-0.000400
F	-1.673135	-2.271876	-0.000886
C	-3.694201	-1.127369	-0.000315
H	-4.194425	-2.089169	-0.000575
F	-1.703137	2.447695	0.000524
N	-0.192508	0.288123	-0.000398
N	0.505681	-0.762614	0.000382
C	1.865694	-0.510906	0.000337
C	2.574455	0.689543	-0.000421
C	2.866408	-1.509382	0.001245
N	4.079611	-0.981092	0.000858

N	3.873057	0.352261	-0.000173
C	5.015260	1.232588	-0.000943
H	5.622387	1.050462	-0.890455
H	4.666949	2.265711	-0.001099
H	5.623085	1.050966	0.888187
H	2.228183	1.711414	-0.001186
H	2.729094	-2.581817	0.002060
H	-5.486386	0.053547	0.000218

24

4pzHF2-trans-n ; E = -803.644114247 Hartree

C	2.553349	1.148672	-0.000091
C	3.924827	0.963566	-0.000388
C	1.630439	0.086976	0.000173
H	4.573531	1.831592	-0.000640
C	4.422115	-0.334607	-0.000372
C	2.186831	-1.207480	0.000199
F	1.395517	-2.276170	0.000524
C	3.555589	-1.422591	-0.000051
H	3.917588	-2.444488	-0.000008
F	2.080107	2.393547	-0.000168
N	0.283833	0.465590	0.000567
N	-0.552284	-0.479017	0.000073
C	-1.863207	-0.038675	0.000042
C	-2.955763	-0.896599	-0.000682
C	-2.435195	1.260778	0.000356
N	-3.754830	1.200512	0.000482
N	-4.049801	-0.117143	-0.000148
C	-5.432438	-0.523710	-0.000368
H	-5.934259	-0.136035	-0.889855
H	-5.481751	-1.613020	-0.000308
H	-5.934525	-0.135918	0.888911
H	-3.001916	-1.975290	-0.001235
H	-1.921226	2.210698	0.000807
H	5.494239	-0.501502	-0.000601

28

4pzHOMe1A-cis-m ; E = -719.750479204 Hartree

C	1.605746	-0.493443	0.336936
C	2.331574	0.434324	1.080237
C	1.287391	-0.206485	-1.004489
H	2.578414	0.233537	2.116536
C	2.741051	1.633839	0.494087
C	1.733310	0.972754	-1.585435
C	2.447538	1.906623	-0.835084
H	2.777399	2.833235	-1.293576
N	0.600106	-1.170208	-1.798330
N	-0.632672	-1.333331	-1.677334
C	-1.441852	-0.579717	-0.823952
C	-1.297941	0.588386	-0.064671
C	-2.797977	-0.940890	-0.634991
N	-3.429695	-0.100916	0.164879
N	-2.499531	0.819439	0.489079
C	-2.874365	1.920720	1.341476
H	-3.268867	1.537218	2.284745
H	-1.993997	2.534188	1.535277
H	-3.642606	2.525762	0.854274
H	-0.467800	1.256622	0.099721
H	-3.305433	-1.795301	-1.061146
H	3.303789	2.347729	1.087845

H	1.495230	1.154252	-2.629129
O	1.136669	-1.670789	0.809425
C	1.398098	-1.986591	2.155869
H	2.475359	-2.054885	2.353627
H	0.953819	-1.248716	2.836569
H	0.938450	-2.959329	2.331902

28

4pzHOMe1A-cis-n ; E = -719.750440189 Hartree

C	1.634812	-0.642014	-0.229605
C	2.499666	-0.888486	0.833860
C	1.459782	0.678647	-0.686302
H	2.636954	-1.897445	1.205411
C	3.187680	0.166173	1.435564
C	2.183365	1.711685	-0.104777
C	3.033939	1.465370	0.971213
H	3.576000	2.283930	1.433363
N	0.654760	0.955024	-1.829314
N	-0.589714	0.859580	-1.789964
C	-1.316588	0.559310	-0.634122
C	-2.680067	0.312685	-0.750066
C	-1.077293	0.492893	0.771289
N	-2.190933	0.223726	1.431195
N	-3.148884	0.109982	0.488827
C	-4.492679	-0.220334	0.891967
H	-4.824544	0.480485	1.660329
H	-5.150819	-0.150447	0.025335
H	-4.528737	-1.235263	1.296032
H	-3.301390	0.273431	-1.632450
H	-0.161209	0.648801	1.320080
H	3.852889	-0.042642	2.267786
H	2.052528	2.714541	-0.499781
O	0.907074	-1.586684	-0.870219
C	0.988669	-2.908449	-0.393799
H	2.007257	-3.307960	-0.479202
H	0.659974	-2.979775	0.650932
H	0.320197	-3.497239	-1.022613

28

4pzHOMe1A-trans-m ; E = -719.765917403 Hartree

C	2.417427	0.585195	0.000100
C	3.780820	0.277760	0.000098
C	1.469511	-0.463274	0.000064
H	4.521534	1.068359	0.000196
C	4.197040	-1.049353	0.000024
C	1.914935	-1.788408	0.000000
C	3.267967	-2.087514	-0.000032
H	3.599799	-3.120865	-0.000093
N	0.116032	-0.084966	0.000094
N	-0.691554	-1.051828	-0.000028
C	-2.021023	-0.663665	0.000007
C	-2.599249	0.603396	0.000225
C	-3.121131	-1.550468	-0.000229
N	-4.273375	-0.897582	-0.000166
N	-3.927981	0.405222	0.000123
C	-4.969701	1.401718	0.000014
H	-5.594892	1.285418	0.888204
H	-4.513083	2.391832	0.002205
H	-5.592373	1.288197	-0.890329
H	-2.145060	1.582041	0.000358

```
H        -3.099204       -2.631507       -0.000350
H         5.260685       -1.269149        0.000032
H         1.159062       -2.566408       -0.000028
O         1.930485        1.843948        0.000181
C         2.855320        2.905390       -0.000336
H         3.492124        2.890089        0.893459
H         3.492064        2.889262       -0.894153
H         2.262112        3.820543       -0.000730
28
4pzHOMe1A-trans-n ; E = -719.765974238 Hartree
C         2.545172        0.460967        0.000297
C         3.857314       -0.020944        0.000524
C         1.469288       -0.456102       -0.000539
H         4.694042        0.667218        0.001248
C         4.097956       -1.390721       -0.000183
C         1.739436       -1.827668       -0.001277
C         3.042239       -2.299892       -0.001102
H         3.237563       -3.367532       -0.001801
N         0.176840        0.095805       -0.001198
N        -0.749835       -0.757465        0.001320
C        -2.014769       -0.193898        0.000195
C        -3.188442       -0.935285        0.002529
C        -2.451675        1.156104       -0.003249
N        -3.771483        1.231861       -0.002980
N        -4.199121       -0.047430        0.000471
C        -5.615220       -0.312279        0.001516
H        -6.076826        0.120681       -0.889045
H        -5.774292       -1.391139        0.004859
H        -6.076465        0.126140        0.889583
H        -3.346910       -2.003378        0.005434
H        -1.840903        2.046859       -0.005757
H         5.124169       -1.746528       -0.000069
H         0.889183       -2.501273       -0.002075
O         2.225152        1.770843        0.001044
C         3.277803        2.705803        0.001483
H         3.906996        2.608607        0.895510
H         3.907363        2.609038       -0.892330
H         2.805451        3.688662        0.001630
28
4pzHOMe1B-cis-m ; E = -719.750479207 Hartree
C         1.733134       -0.973101       -1.585299
C         2.447375       -1.906856       -0.834835
C         1.287333        0.206281       -1.004535
H         2.777130       -2.833588       -1.293160
C         2.741003       -1.633851        0.494275
C         1.605798        0.493445        0.336810
C         2.331651       -0.434207        1.080235
H         2.578631       -0.233214        2.116460
N         0.600111        1.169932       -1.798470
N        -0.632658        1.333130       -1.677445
C        -1.441825        0.579677       -0.823918
C        -1.297968       -0.588349       -0.064484
C        -2.797927        0.940949       -0.634981
N        -3.429699        0.101094        0.164977
N        -2.499570       -0.819269        0.489265
C        -2.874501       -1.920601        1.341565
H        -3.642427       -2.525806        0.854070
H        -1.994088       -2.533889        1.535748
```

H	-3.269456	-1.537104	2.284642
H	-0.467815	-1.256504	0.100126
H	-3.305363	1.795324	-1.061208
H	3.303773	-2.347661	1.088098
O	1.136757	1.670888	0.809135
C	1.398377	1.986913	2.155488
H	0.954280	1.249103	2.836380
H	2.475662	2.055346	2.353060
H	0.938655	2.959631	2.331444
H	1.494982	-1.154755	-2.628945

28

4pzHOMe1B-cis-n ; E = -719.750440194 Hartree

C	2.183500	-1.711868	-0.104102
C	3.034350	-1.465087	0.971563
C	1.459795	-0.679081	-0.685925
H	3.576450	-2.283473	1.433979
C	3.188282	-0.165687	1.435268
C	1.635001	0.641785	-0.229845
C	2.500157	0.888729	0.833255
H	2.637636	1.897870	1.204245
N	0.654609	-0.955896	-1.828711
N	-0.589855	-0.860285	-1.789373
C	-1.316743	-0.559367	-0.633748
C	-2.680346	-0.313479	-0.749882
C	-1.077483	-0.491507	0.771622
N	-2.191224	-0.222237	1.431285
N	-3.149259	-0.109860	0.488806
C	-4.493164	0.220346	0.891672
H	-4.529809	1.235987	1.293902
H	-5.151479	0.148437	0.025339
H	-4.824337	-0.479305	1.661389
H	-3.301703	-0.275356	-1.632326
H	-0.161326	-0.646423	1.320580
H	3.853757	0.043495	2.267184
O	0.907115	1.586203	-0.870686
C	0.988868	2.908167	-0.394840
H	0.660570	2.979923	0.649986
H	2.007416	3.307660	-0.480802
H	0.320134	3.496684	-1.023632
H	2.052572	-2.714878	-0.498641

28

4pzHOMe1B-trans-m ; E = -719.761466839 Hartree

C	2.008300	-1.974804	-0.000590
C	3.375635	-2.202784	-0.001194
C	1.462714	-0.685889	0.000006
H	3.765678	-3.215048	-0.001836
C	4.233519	-1.109083	-0.000859
C	2.350395	0.424877	0.000420
C	3.728022	0.187592	0.000057
H	4.419311	1.021722	0.000499
N	0.061068	-0.685896	0.000608
N	-0.521082	0.432196	-0.001602
C	-1.904002	0.323121	-0.001074
C	-2.738266	-0.792068	0.001466
C	-2.792100	1.422090	-0.003676
N	-4.056976	1.028990	-0.002434
N	-3.995612	-0.317106	0.000473
C	-5.224579	-1.070411	0.002735

```
H       -5.811654       -0.826629       -0.885684
H       -4.988183       -2.134864        0.002727
H       -5.809038       -0.826111        0.892770
H       -2.506160       -1.845536        0.003818
H       -2.539840        2.473427       -0.006075
H        5.309716       -1.255398       -0.001195
O        1.819495        1.660244        0.001515
C        2.696016        2.762982        0.002216
H        3.331431        2.776065        0.896802
H        3.332568        2.776197       -0.891536
H        2.058268        3.647294        0.001899
H        1.296246       -2.794101       -0.000651
28
4pzHOMe1B-trans-n ; E = -719.761672894 Hartree
C       -2.298287       -1.872682        0.000155
C       -3.684143       -1.905411        0.000491
C       -1.576460       -0.673589       -0.000029
H       -4.212899       -2.852632        0.000693
C       -4.379212       -0.701845        0.000533
C       -2.298571        0.551046       -0.000065
C       -3.695813        0.510700        0.000231
H       -4.262933        1.433734        0.000206
N       -0.189052       -0.873556       -0.000339
N        0.546550        0.149925        0.000427
C        1.898070       -0.162249        0.000100
C        2.902415        0.795711        0.000827
C        2.594193       -1.398772       -0.001132
N        3.903364       -1.212554       -0.001012
N        4.069293        0.125781        0.000234
C        5.404766        0.665722        0.000724
H        5.943544        0.331282       -0.888989
H        5.346955        1.754746        0.001970
H        5.943690        0.329254        0.889581
H        2.844950        1.873923        0.001853
H        2.175332       -2.394179       -0.001982
H       -5.465292       -0.694860        0.000802
O       -1.598958        1.700667       -0.000481
C       -2.313712        2.914055       -0.000713
H       -2.942078        3.015796       -0.894659
H       -2.941852        3.016212        0.893347
H       -1.559867        3.701946       -0.000965
H       -1.708449       -2.783838        0.000082
32
4pzHOMe2-cis-m ; E = -834.151747414 Hartree
C        1.482879       -1.211640       -0.073012
C        2.184128       -1.213372        1.135860
C        1.118492        0.000143       -0.670210
H        2.465182       -2.143983        1.613550
C        2.523155        0.000547        1.724209
C        1.482280        1.212133       -0.073070
C        2.183531        1.214268        1.135804
H        2.464111        2.145044        1.613450
N        0.486155       -0.000059       -1.950325
N       -0.758288       -0.000256       -2.040137
C       -1.625116       -0.000311       -0.944580
C       -1.504879       -0.000275        0.451176
C       -3.024425       -0.000482       -1.162092
N       -3.705601       -0.000548       -0.030541
```

```
N       -2.760032       -0.000417        0.930161
C       -3.169658       -0.000488        2.312677
H       -3.768226       -0.889887        2.522135
H       -2.281398       -0.000210        2.945159
H       -3.768729        0.888590        2.522045
H       -0.655155       -0.000145        1.114375
H       -3.529010       -0.000550       -2.118407
H        3.069416        0.000704        2.662475
O        1.073053        2.321610       -0.731341
C        1.421278        3.571049       -0.184720
H        0.977146        3.716161        0.808717
H        2.509482        3.692131       -0.112335
H        1.021365        4.320441       -0.868287
O        1.074221       -2.321355       -0.731241
C        1.423039       -3.570596       -0.184544
H        2.511304       -3.691169       -0.112201
H        0.979019       -3.715845        0.808922
H        1.023440       -4.320218       -0.868041
32
4pzHOMe2-cis-n ; E = -834.151756590 Hartree
C        2.124501       -0.736719        0.002965
C        2.904145       -0.249226        1.055393
C        1.182238        0.091785       -0.617475
H        3.621726       -0.886942        1.556665
C        2.749081        1.072128        1.457304
C        1.051952        1.425903       -0.207754
C        1.835290        1.918014        0.837691
H        1.737143        2.944438        1.169012
N        0.497250       -0.385112       -1.774117
N       -0.727531       -0.620177       -1.753351
C       -1.525257       -0.531005       -0.608177
C       -2.897155       -0.711895       -0.741964
C       -1.342131       -0.351736        0.795824
N       -2.496112       -0.414818        1.438608
N       -3.426719       -0.628430        0.486417
C       -4.813294       -0.716809        0.868850
H       -5.161865        0.244582        1.254642
H       -5.405261       -0.994342       -0.003895
H       -4.930349       -1.475137        1.645527
H       -3.485529       -0.888637       -1.630109
H       -0.433048       -0.195975        1.355589
H        3.354730        1.452921        2.273869
O        0.122355        2.146695       -0.878295
C       -0.093095        3.476008       -0.469788
H       -0.428237        3.527220        0.573989
H        0.810004        4.088047       -0.588510
H       -0.877510        3.866683       -1.118743
O        2.172052       -2.004512       -0.467975
C        3.124979       -2.873920        0.095278
H        4.146227       -2.492839       -0.031556
H        2.935907       -3.044837        1.163009
H        3.025060       -3.818064       -0.441111
32
4pzHOMe2-trans-m ; E = -834.161134033 Hartree
C       -2.060221       -1.267658       -0.049874
C       -3.454425       -1.240424       -0.031859
C       -1.305850       -0.070441       -0.025938
H       -4.030295       -2.156136       -0.069322
```

C	-4.104108	-0.014558	0.032692
C	-1.999430	1.162747	0.046498
C	-3.397272	1.178594	0.080427
H	-3.934018	2.116897	0.138804
N	0.079315	-0.247891	0.036230
N	0.789319	0.701284	-0.391746
C	2.149518	0.460330	-0.249809
C	2.833368	-0.628193	0.284292
C	3.169831	1.346654	-0.660048
N	4.374480	0.856651	-0.406067
N	4.141363	-0.340989	0.165834
C	5.263274	-1.149769	0.571006
H	5.865676	-0.609766	1.305209
H	4.892012	-2.074548	1.013675
H	5.885925	-1.384170	-0.295579
H	2.464333	-1.544897	0.717241
H	3.054552	2.314906	-1.127179
H	-5.189642	0.011582	0.046542
O	-1.254880	2.281787	0.123587
C	-1.914033	3.504381	0.353871
H	-2.568875	3.775814	-0.483721
H	-2.504243	3.481337	1.278819
H	-1.126204	4.252625	0.446374
O	-1.338245	-2.407287	-0.114452
C	-2.038314	-3.628138	-0.109075
H	-2.644536	-3.746184	0.798195
H	-2.687058	-3.728321	-0.988671
H	-1.278002	-4.409672	-0.136328

32

4pzHOMe2-trans-n ; E = -834.161208341 Hartree

C	-2.277943	-1.099185	-0.056882
C	-3.651031	-0.860785	-0.112917
C	-1.352673	-0.029401	-0.012936
H	-4.356694	-1.679871	-0.165435
C	-4.108694	0.450369	-0.102858
C	-1.852484	1.295379	0.002240
C	-3.231397	1.523730	-0.037666
H	-3.621319	2.533534	-0.022751
N	-0.015377	-0.411643	0.131434
N	0.849089	0.398966	-0.296331
C	2.147128	-0.038540	-0.069607
C	3.273774	0.669060	-0.462704
C	2.664040	-1.204092	0.552204
N	3.986619	-1.208379	0.539509
N	4.336795	-0.063223	-0.080689
C	5.733850	0.238402	-0.258102
H	6.213352	-0.544450	-0.850626
H	5.827412	1.193592	-0.776058
H	6.229776	0.301853	0.713482
H	3.366946	1.615750	-0.973524
H	2.107627	-2.017302	0.994419
H	-5.176894	0.640935	-0.146484
O	-0.950214	2.291411	0.101741
C	-1.428245	3.601461	0.293393
H	-1.993140	3.958947	-0.576896
H	-2.059879	3.678388	1.187545
H	-0.542640	4.224740	0.422405
O	-1.734413	-2.334420	-0.065510

C	-2.608679	-3.437427	-0.062343
H	-3.265545	-3.433335	0.816832
H	-3.224929	-3.469702	-0.970034
H	-1.972807	-4.322879	-0.031120

36

4pzHPyr1A-cis-m ; E = -816.520560386 Hartree

C	-0.532170	-2.719224	1.025498
C	-0.803629	-3.513653	-0.082511
C	-0.670223	-1.334894	0.978861
H	-0.656942	-4.587621	-0.038724
C	-1.260913	-2.898877	-1.243180
C	-1.213674	-0.703389	-0.170299
C	-1.475372	-1.526181	-1.283155
H	-1.854204	-1.081804	-2.195966
N	-0.277862	-0.634056	2.160876
N	0.713497	0.133164	2.151481
C	1.547357	0.334585	1.049009
C	1.764424	-0.295547	-0.182402
C	2.540790	1.344338	1.090757
N	3.286809	1.354537	0.000937
N	2.800609	0.343815	-0.748300
C	3.422540	0.051503	-2.016025
H	4.457007	-0.264775	-1.862064
H	2.865399	-0.746707	-2.507365
H	3.413603	0.945065	-2.643631
H	1.285366	-1.130062	-0.669275
H	2.710830	2.053144	1.889673
H	-1.478635	-3.491344	-2.127238
C	-2.552585	2.627035	0.367227
C	-1.675492	1.509547	0.922158
N	-1.430104	0.652447	-0.237233
C	-1.900767	1.271883	-1.464762
C	-2.081955	2.733934	-1.080245
H	-3.607187	2.329896	0.395608
H	-2.151682	0.945009	1.729674
H	-1.178930	1.128653	-2.278215
H	-1.119762	3.255743	-1.127696
H	-2.860608	0.837593	-1.791623
H	-2.785793	3.253291	-1.735149
H	-2.447427	3.556677	0.931889
H	-0.735440	1.913088	1.321872
H	-0.156233	-3.151562	1.948306

36

4pzHPyr1A-cis-n ; E = -816.519600928 Hartree

C	-2.200692	-2.128670	0.736211
C	-2.982623	-2.405606	-0.376884
C	-1.432967	-0.967873	0.816302
H	-3.540833	-3.333645	-0.440152
C	-3.025151	-1.472225	-1.408107
C	-1.543400	0.032975	-0.182888
C	-2.332594	-0.272722	-1.308340
H	-2.404568	0.443256	-2.118414
N	-0.624736	-0.858637	1.986219
N	0.623124	-0.757582	1.922134
C	1.368330	-0.810335	0.739286
C	2.731110	-0.535189	0.807511
C	1.166525	-1.179400	-0.624946
N	2.298108	-1.125537	-1.306398

```
N        3.233086       -0.724042       -0.420292
C        4.587455       -0.528222       -0.871586
H        4.639803        0.318070       -1.561568
H        5.225295       -0.333724       -0.008698
H        4.932616       -1.427287       -1.385588
H        3.331561       -0.230175        1.651875
H        0.262602       -1.495755       -1.121983
H       -3.621659       -1.663559       -2.295403
C       -0.571235        3.379999        0.816617
C       -0.545782        1.894901        1.164665
N       -0.880299        1.235684       -0.096378
C       -0.909759        2.182875       -1.198454
C       -0.153786        3.385399       -0.651214
H       -1.588700        3.775393        0.915277
H       -1.259060        1.619429        1.948296
H       -0.447173        1.752015       -2.094182
H        0.925817        3.219976       -0.739130
H       -1.943160        2.468455       -1.458027
H       -0.402997        4.310442       -1.176848
H        0.084596        3.969752        1.462075
H        0.450498        1.595913        1.517352
H       -2.122194       -2.833213        1.559090
36
4pzHPyr1A-trans-m ; E = -816.530527678 Hartree
C        1.340975       -2.699847       -0.368021
C        2.601855       -3.210790       -0.124639
C        1.031003       -1.344775       -0.177097
H        2.805115       -4.269301       -0.246090
C        3.594181       -2.333264        0.313097
C        2.079964       -0.421366        0.125455
C        3.343315       -0.976598        0.429004
H        4.151759       -0.322459        0.731835
N       -0.347128       -1.106021       -0.245942
N       -0.792869       -0.156435        0.456744
C       -2.174223       -0.017515        0.373742
C       -3.120822       -0.715986       -0.369243
C       -2.929882        0.931467        1.097497
N       -4.224475        0.831804        0.830804
N       -4.313422       -0.175433       -0.057323
C       -5.604192       -0.515598       -0.601244
H       -5.934369        0.244311       -1.315269
H       -5.536545       -1.481075       -1.104057
H       -6.328439       -0.577128        0.212493
H       -3.007438       -1.531268       -1.066513
H       -2.567693        1.666192        1.803566
H        4.589351       -2.704133        0.542112
C        1.611341        2.950098       -1.094377
C        0.948308        1.618915       -0.754517
N        1.928010        0.938167        0.083790
C        3.027186        1.825124        0.433793
C        2.476895        3.212268        0.134179
H        2.245922        2.845757       -1.982133
H        0.714094        1.014636       -1.638910
H        3.318069        1.692528        1.482155
H        1.856928        3.559303        0.968000
H        3.915266        1.623598       -0.188278
H        3.268498        3.946799       -0.032812
H        0.880363        3.738147       -1.291754
```

```
H         0.009662        1.759328        -0.204735
H         0.513219       -3.342608        -0.651895
36
4pzHPyr1A-trans-n ; E = -816.530453350 Hartree
C        -1.717715       -2.621785         0.353916
C        -3.009560       -2.964589         0.000806
C        -1.219484       -1.319707         0.198862
H        -3.358494       -3.987055         0.097296
C        -3.838729       -1.966971        -0.511697
C        -2.109124       -0.268926        -0.184924
C        -3.404322       -0.654677        -0.595840
H        -4.092549        0.098407        -0.959778
N         0.167436       -1.261430         0.388658
N         0.791751       -0.391678        -0.280035
C         2.166521       -0.436822        -0.074220
C         3.071050        0.374875        -0.743322
C         2.972760       -1.226042         0.784837
N         4.254367       -0.923901         0.652139
N         4.292818        0.050741        -0.276714
C         5.569402        0.560495        -0.707615
H         6.059403       -0.140799        -1.389684
H         5.422042        1.515015        -1.215067
H         6.204073        0.707095         0.167438
H         2.915386        1.131284        -1.498257
H         2.650015       -1.981920         1.485325
H        -4.851097       -2.205613        -0.825598
C        -1.307113        3.011040         1.105001
C        -0.796448        1.605466         0.803343
N        -1.783075        1.059833        -0.121595
C        -2.728600        2.081612        -0.544146
C        -2.030685        3.385617        -0.184729
H        -2.019067        2.987988         1.938144
H        -0.716667        0.974697         1.696533
H        -2.951030        1.991070        -1.613624
H        -1.306836        3.651824        -0.962869
H        -3.681638        1.994554         0.004106
H        -2.732203        4.215786        -0.071650
H        -0.499304        3.697728         1.370289
H         0.195274        1.622951         0.335111
H        -1.008113       -3.365478         0.703478
36
4pzHPyr1B-cis-m ; E = -816.518559927 Hartree
C         1.463717        0.854665        -0.102699
C         1.789800        1.972823         0.691590
C         0.336586        0.976212        -0.951054
H         2.641777        1.918982         1.359140
C         1.048466        3.145319         0.639970
C        -0.394557        2.163208        -0.993810
C        -0.054228        3.252437        -0.202854
H        -0.626369        4.172505        -0.260501
N         0.048089       -0.017397        -1.934008
N        -1.027356       -0.656262        -1.920794
C        -1.952819       -0.612874        -0.871867
C        -2.002083       -0.059528         0.414898
C        -3.140479       -1.381268        -0.953153
N        -3.855826       -1.311786         0.154967
N        -3.140130       -0.509568         0.968314
C        -3.648276       -0.219383         2.286636
```

H	-3.757860	-1.146423	2.853812
H	-2.948820	0.442633	2.797778
H	-4.622768	0.267608	2.208669
H	-1.339538	0.602728	0.947974
H	-3.470718	-1.974929	-1.794484
H	1.342551	3.983269	1.265403
C	2.613629	-2.561488	0.468046
C	1.699222	-1.634629	-0.332328
N	2.215845	-0.294475	-0.041104
C	3.421863	-0.353676	0.764815
C	3.919417	-1.775676	0.548309
H	2.210089	-2.712184	1.475894
H	1.746578	-1.849890	-1.404253
H	4.142470	0.407707	0.444037
H	4.585174	-2.112935	1.346658
H	0.651883	-1.724291	-0.020554
H	2.721167	-3.542983	-0.000156
H	4.461774	-1.840347	-0.401218
H	3.208945	-0.180094	1.834538
H	-1.232229	2.218208	-1.683362

36
4pzHPyr1B-cis-n ; E = -816.518113843 Hartree

C	1.701090	0.760023	-0.074592
C	2.226548	1.836344	0.667067
C	0.491210	0.987119	-0.774591
H	3.150054	1.702550	1.217939
C	1.589229	3.068531	0.709903
C	-0.136830	2.232076	-0.717815
C	0.397716	3.277868	0.021119
H	-0.096529	4.243434	0.041630
N	0.034997	0.042971	-1.738530
N	-1.119286	-0.437298	-1.707158
C	-2.000254	-0.285483	-0.627065
C	-3.286614	-0.803686	-0.737437
C	-1.928653	0.156370	0.728789
N	-3.064720	-0.073045	1.365623
N	-3.876618	-0.648635	0.456424
C	-5.227531	-0.980477	0.833949
H	-5.811116	-0.070882	0.998332
H	-5.685582	-1.567176	0.036966
H	-5.212191	-1.564761	1.755722
H	-3.776812	-1.261107	-1.584093
H	-1.108482	0.621545	1.253137
H	2.033240	3.872125	1.290254
C	2.547231	-2.748653	0.408505
C	1.656701	-1.736015	-0.310429
N	2.341571	-0.456340	-0.105588
C	3.618094	-0.640866	0.561713
C	3.931280	-2.111143	0.323737
H	2.245328	-2.839259	1.458179
H	1.565108	-1.966637	-1.376269
H	4.376200	0.033643	0.146712
H	4.642949	-2.510583	1.050622
H	0.644990	-1.705577	0.111363
H	2.494318	-3.742327	-0.043391
H	4.352044	-2.246225	-0.678672
H	3.546062	-0.434891	1.644064
H	-1.046480	2.370520	-1.295007

36
4pzHPyr1B-trans-m ; E = -816.536534834 Hartree

C	-2.166021	-0.360742	-0.014315
C	-3.444689	-0.970205	-0.050077
C	-1.038664	-1.233987	0.032704
H	-4.329961	-0.347770	-0.084423
C	-3.599087	-2.341849	-0.017213
C	-1.230714	-2.625017	0.077235
C	-2.488004	-3.188806	0.053123
H	-2.612656	-4.266302	0.083539
N	0.236190	-0.662257	0.002785
N	1.200014	-1.477726	0.016883
C	2.443385	-0.866867	-0.011301
C	2.809228	0.477164	-0.015422
C	3.674634	-1.560804	-0.028938
N	4.706053	-0.729242	-0.044878
N	4.155443	0.498601	-0.031430
C	5.017166	1.651686	-0.100037
H	5.394801	1.793546	-1.116938
H	4.457055	2.536566	0.205423
H	5.861148	1.502253	0.574785
H	2.212032	1.375599	-0.003590
H	3.828205	-2.631019	-0.025402
H	-4.602325	-2.758442	-0.038322
C	-1.500969	3.201679	0.536560
C	-0.915681	1.818194	0.270716
N	-2.086119	1.004333	-0.041222
C	-3.269260	1.826314	-0.252932
C	-2.714970	3.238992	-0.384593
H	-1.820934	3.279137	1.581988
H	-0.211925	1.830210	-0.572285
H	-3.814334	1.504206	-1.148019
H	-3.450072	4.001249	-0.115003
H	-0.372289	1.407234	1.125397
H	-0.781413	4.001811	0.344115
H	-2.399289	3.421229	-1.417852
H	-3.962345	1.755280	0.600985
H	-0.334904	-3.235265	0.117568

36
4pzHPyr1B-trans-n ; E = -816.537343706 Hartree

C	-2.181474	-0.511461	-0.011215
C	-3.362935	-1.293960	-0.043785
C	-0.943342	-1.219826	0.037650
H	-4.326182	-0.800817	-0.079239
C	-3.324868	-2.673395	-0.005820
C	-0.940368	-2.623856	0.087229
C	-2.106715	-3.357605	0.066491
H	-2.080406	-4.441884	0.100968
N	0.238960	-0.476046	0.003899
N	1.307517	-1.148554	0.020247
C	2.449104	-0.363901	-0.011838
C	3.736628	-0.882087	-0.032257
C	2.640680	1.042336	-0.034337
N	3.926731	1.353065	-0.067216
N	4.574221	0.172336	-0.071415
C	6.014397	0.160642	-0.041244
H	6.385484	0.390228	0.962125
H	6.371241	-0.825497	-0.341868

```
H        6.388184        0.911405        -0.738880
H        4.083149       -1.904687        -0.026836
H        1.888401        1.817399        -0.032851
H       -4.260463       -3.225560        -0.024404
C       -2.015644        3.110949         0.521474
C       -1.244355        1.821148         0.261094
N       -2.292307        0.850060        -0.041970
C       -3.578017        1.499318        -0.256846
C       -3.224566        2.974459        -0.396951
H       -2.341479        3.149052         1.567302
H       -0.551982        1.925724        -0.584075
H       -4.073826        1.099599        -1.149344
H       -4.057994        3.628908        -0.129986
H       -0.646830        1.495129         1.115833
H       -1.413502        4.001230         0.323104
H       -2.938569        3.192905        -1.431816
H       -4.253907        1.337668         0.598524
H        0.031683       -3.103378         0.128761
48
4pzHPyr2-cis-m ; E = -1027.68676191 Hartree
C       -1.265612       -0.936145         0.794309
C       -1.245377       -0.864406         2.197880
C       -0.030751       -0.920673         0.112287
H       -2.175441       -0.841736         2.752413
C       -0.035835       -0.824364         2.879305
C        1.196174       -0.963407         0.803576
C        1.176255       -0.903108         2.205535
H        2.105539       -0.918246         2.762305
N       -0.015824       -0.802908        -1.313132
N        0.030676        0.339139        -1.826632
C        0.061319        1.529007        -1.100803
C        0.041265        1.909744         0.248247
C        0.123287        2.761330        -1.798366
N        0.141011        3.797722        -0.980963
N        0.089579        3.250921         0.252185
C        0.090491        4.119770         1.402698
H       -0.777962        4.781575         1.369213
H        0.051172        3.509047         2.305199
H        0.999278        4.725781         1.408969
H       -0.003567        1.353283         1.171328
H        0.155247        2.895624        -2.870937
H       -0.039439       -0.768707         3.964285
C        4.044099       -1.619909        -1.453147
C        2.629256       -1.943547        -0.954431
N        2.389266       -0.947016         0.087678
C        3.646424       -0.764077         0.782463
C        4.658291       -0.718323        -0.360564
H        4.620883       -2.536594        -1.601650
H        2.588845       -2.959194        -0.526019
H        3.627426        0.154207         1.377785
H        4.752055        0.308047        -0.725210
C       -4.027740       -1.218664        -1.579753
C       -2.718101       -1.776366        -1.044544
N       -2.457656       -0.893927         0.086430
C       -3.713115       -0.695953         0.787423
C       -4.791088       -0.805958        -0.309005
H       -3.814426       -0.342751        -2.199501
H       -2.838895       -2.820892        -0.706865
```

H	-3.879116	-1.468318	1.558218
H	-5.326989	0.136393	-0.446520
H	-1.896840	-1.745144	-1.762995
H	-4.575806	-1.941138	-2.189802
H	-5.532259	-1.561026	-0.032310
H	-3.709645	0.275301	1.294676
H	3.869826	-1.605590	1.464979
H	5.649724	-1.049640	-0.041018
H	4.010835	-1.097908	-2.412616
H	1.864742	-1.879604	-1.731552

48

4pzHPyr2-cis-n ; E = -1027.68684943 Hartree

C	-0.652704	-1.427841	0.659667
C	-0.539653	-1.729926	2.021788
C	0.452815	-0.858655	-0.004859
H	-1.382040	-2.148005	2.559203
C	0.656095	-1.479070	2.685137
C	1.698820	-0.701791	0.636978
C	1.767207	-0.993359	2.014167
H	2.694487	-0.842264	2.552912
N	0.287251	-0.406901	-1.354402
N	-0.278762	0.689635	-1.565312
C	-0.724724	1.554876	-0.561923
C	-1.422398	2.693108	-0.954699
C	-0.644540	1.679252	0.859670
N	-1.240723	2.784622	1.272762
N	-1.710669	3.381682	0.157094
C	-2.444884	4.615130	0.279780
H	-1.840231	5.350849	0.814173
H	-2.678769	4.990253	-0.717143
H	-3.372371	4.448224	0.833224
H	-1.711269	3.017310	-1.943576
H	-0.176571	1.029631	1.583666
H	0.732918	-1.697857	3.746377
C	4.572893	-0.321700	-1.568930
C	3.052685	-0.434158	-1.450863
N	2.792524	-0.191300	-0.029204
C	4.013102	0.133306	0.685105
C	4.944681	0.610743	-0.419337
H	5.036472	-1.302461	-1.412202
H	2.678625	-1.416370	-1.756722
H	3.824447	0.894816	1.450921
H	4.712866	1.649349	-0.679955
C	-3.356038	-2.104704	-1.721159
C	-1.911774	-2.315589	-1.278415
N	-1.857286	-1.567528	-0.028035
C	-3.066764	-1.899652	0.696538
C	-4.142958	-1.961624	-0.399287
H	-3.425261	-1.183639	-2.306520
H	-1.705410	-3.386073	-1.099868
H	-2.986526	-2.879357	1.202360
H	-4.748532	-1.051814	-0.403255
H	-1.174814	-1.941215	-1.992042
H	-3.718656	-2.925514	-2.345325
H	-4.821233	-2.802097	-0.229316
H	-3.268970	-1.145739	1.463855
H	4.443362	-0.748737	1.191546
H	5.998184	0.555646	-0.133507

H 4.887518 0.044471 -2.549551
H 2.544269 0.309040 -2.075735
48
4pzHPyr2-trans-m ; E = -1027.69725120 Hartree
C 0.787205 -2.106889 0.207035
C 1.908693 -2.865438 0.598821
C 0.917315 -0.675177 0.179464
H 1.826847 -3.936889 0.717603
C 3.105461 -2.244251 0.909021
C 2.219110 -0.078016 0.319839
C 3.284793 -0.879183 0.763863
H 4.258926 -0.446383 0.947295
N -0.279068 0.013946 0.105944
N -0.331390 1.174700 0.614274
C -1.595604 1.745254 0.512236
C -2.745793 1.324616 -0.149824
C -1.973268 2.965398 1.117175
N -3.238467 3.270899 0.861559
N -3.683643 2.264590 0.090948
C -5.045438 2.291408 -0.378205
H -5.218546 3.190648 -0.974287
H -5.226105 1.408494 -0.992402
H -5.734279 2.290665 0.470089
H -2.933532 0.460902 -0.768352
H -1.363131 3.613618 1.731178
H 3.941109 -2.853158 1.243574
C 2.721734 2.914345 -1.637992
C 1.699621 1.969048 -1.016212
N 2.461816 1.225669 -0.022316
C 3.782731 1.807536 0.164317
C 3.664219 3.186010 -0.469909
H 3.265203 2.414168 -2.448257
H 1.241784 1.283268 -1.738703
H 4.049202 1.839901 1.226923
H 3.202121 3.887272 0.233585
C -2.609624 -3.469322 -0.314503
C -1.708530 -2.237999 -0.313616
N -0.375148 -2.781317 -0.074222
C -0.365946 -4.233895 -0.197675
C -1.698753 -4.562215 -0.856618
H -2.913651 -3.708811 0.710921
H -1.750445 -1.706121 -1.274752
H 0.487056 -4.566144 -0.800268
H -2.038629 -5.575475 -0.627725
H -1.981607 -1.512748 0.455399
H -3.516052 -3.319104 -0.907155
H -1.607534 -4.472429 -1.944967
H -0.282118 -4.718466 0.788474
H 4.552842 1.213934 -0.355548
H 4.634264 3.590603 -0.769269
H 2.257541 3.815779 -2.046084
H 0.882479 2.519713 -0.533883
48
4pzHPyr2-trans-n ; E = -1027.69767461 Hartree
C -2.309146 0.081621 0.272291
C -3.381079 -0.688260 0.769410
C -1.014945 -0.540424 0.190171
H -4.348056 -0.232868 0.931953

C	-3.192988	-2.011601	1.124835
C	-0.897241	-1.961531	0.383172
C	-1.986271	-2.662103	0.927620
H	-1.906576	-3.717794	1.149270
N	0.039475	0.336414	0.013894
N	1.175663	0.000424	0.466271
C	2.136961	0.985288	0.264259
C	3.418330	0.930581	0.793770
C	2.117005	2.201387	-0.466872
N	3.279353	2.833295	-0.387188
N	4.054612	2.046329	0.379157
C	5.376466	2.492450	0.736298
H	5.345795	3.172180	1.593841
H	5.996045	1.628547	0.983315
H	5.806362	3.016835	-0.117719
H	3.889195	0.189999	1.422970
H	1.310951	2.609275	-1.058968
H	-4.030124	-2.567558	1.538597
C	1.591997	-3.554282	-1.689167
C	1.105666	-2.242447	-1.082793
N	0.218690	-2.656540	-0.003783
C	0.315177	-4.089708	0.228966
C	1.601105	-4.486530	-0.481952
H	0.878649	-3.919581	-2.437272
H	0.570212	-1.603976	-1.795057
H	0.325440	-4.312165	1.302159
H	2.466726	-4.272965	0.154912
C	-2.445839	3.709980	-0.419206
C	-1.603835	2.438078	-0.408793
N	-2.557848	1.390700	-0.052427
C	-3.929287	1.881965	-0.113346
C	-3.818730	3.211021	-0.848401
H	-2.498435	4.130157	0.591726
H	-1.153986	2.242773	-1.391924
H	-4.573271	1.169043	-0.640758
H	-4.635523	3.893533	-0.599969
H	-0.781015	2.480409	0.306609
H	-2.029533	4.475159	-1.079492
H	-3.836812	3.039706	-1.930616
H	-4.348733	2.021747	0.896057
H	-0.543453	-4.621228	-0.213844
H	1.621585	-5.547638	-0.742688
H	2.567006	-3.451443	-2.172402
H	1.937864	-1.649417	-0.683753

30

4pzMe-cis-m ; E = -683.903377388 Hartree

C	-2.809900	-0.470705	-0.926639
C	-3.724585	0.389703	-0.331275
C	-1.671354	-0.876998	-0.227442
H	-4.596557	0.718532	-0.888861
C	-3.535335	0.810719	0.982826
C	-1.492301	-0.475748	1.101229
H	-0.628203	-0.824482	1.656750
C	-2.425978	0.361653	1.698581
H	-2.287446	0.665353	2.732197
H	-2.954561	-0.834477	-1.939410
N	-0.831386	-1.838067	-0.856698
N	0.416798	-1.774790	-0.827334

C	1.141242	-0.671497	-0.360565
C	0.988719	0.706016	-0.544756
C	2.397978	-0.827715	0.282761
N	2.952076	0.350182	0.520989
N	2.094568	1.256724	0.003908
C	2.466206	2.649447	0.016027
H	2.719253	3.003533	-0.988929
H	1.657665	3.263999	0.420231
H	3.342953	2.735857	0.656017
C	-0.043753	1.510267	-1.246766
H	0.385994	2.418021	-1.679219
H	-0.486803	0.927554	-2.056169
H	-0.859242	1.796535	-0.572234
C	3.035079	-2.103823	0.703373
H	2.577081	-2.499816	1.616284
H	2.908325	-2.860331	-0.076100
H	4.098209	-1.946944	0.894819
H	-4.257168	1.470412	1.454447

30

4pzMe-cis-n ; E = -683.904412835 Hartree

C	-2.967972	0.633401	0.779835
C	-3.907787	-0.276279	0.311824
C	-1.793260	0.857625	0.058894
H	-4.807546	-0.466419	0.889403
C	-3.706751	-0.928252	-0.903024
C	-1.600247	0.223394	-1.173088
H	-0.703962	0.429877	-1.748406
C	-2.559168	-0.662378	-1.648389
H	-2.408597	-1.151118	-2.606547
H	-3.118017	1.171728	1.710544
N	-0.918439	1.872618	0.527589
N	0.323115	1.734792	0.564909
C	1.006599	0.529145	0.364652
C	2.312662	0.572240	-0.124086
C	0.790896	-0.838685	0.729741
N	1.871635	-1.553768	0.453154
N	2.764312	-0.694920	-0.084775
C	4.046239	-1.186941	-0.519218
H	4.179769	-1.039711	-1.595377
H	4.859574	-0.684109	0.012573
H	4.071452	-2.251861	-0.292954
C	3.092906	1.732129	-0.617941
H	3.175726	1.738751	-1.711050
H	2.579533	2.645858	-0.310575
H	4.107778	1.739525	-0.207298
C	-0.353406	-1.492506	1.423000
H	-0.718408	-0.875793	2.248727
H	-1.196660	-1.662558	0.746984
H	-0.021522	-2.453846	1.820277
H	-4.447749	-1.628733	-1.275634

30

4pzMe-trans-m ; E = -683.923838401 Hartree

C	-3.022466	-1.360107	0.000263
C	-4.403766	-1.204314	0.000210
C	-2.187387	-0.240737	0.000040
H	-5.047736	-2.078666	0.000386
C	-4.957807	0.072396	-0.000070
C	-2.746015	1.044124	-0.000243

```
H       -2.079593        1.899629       -0.000416
C       -4.124068        1.192837       -0.000295
H       -4.558007        2.188763       -0.000516
H       -2.562439       -2.343575        0.000482
N       -0.801748       -0.510983        0.000121
N       -0.077809        0.520132       -0.000011
C        1.285234        0.314287        0.000020
C        2.079003       -0.841955       -0.000042
C        2.204833        1.399839        0.000084
N        3.450883        0.959829        0.000075
N        3.348370       -0.392462       -0.000004
C        4.550224       -1.186329       -0.000135
H        5.389375       -0.492509        0.000430
H        4.600633       -1.820920        0.890006
H        4.601091       -1.819994       -0.890917
C        1.743178       -2.288602       -0.000156
H        2.152471       -2.791366        0.884120
H        0.660019       -2.399060       -0.000747
H        2.153562       -2.791497       -0.883845
C        1.890801        2.853632        0.000193
H        1.300576        3.126683       -0.879585
H        1.301351        3.126739        0.880476
H        2.816740        3.431556       -0.000218
H       -6.036494        0.198176       -0.000116
30
4pzMe-trans-n ; E = -683.925382155 Hartree
C       -3.180892        1.157125       -0.000430
C       -4.535487        0.845674       -0.000463
C       -2.223575        0.139981        0.000030
H       -5.274455        1.641373       -0.000843
C       -4.941345       -0.485701        0.000006
C       -2.633511       -1.199611        0.000518
H       -1.876181       -1.975663        0.000899
C       -3.985930       -1.504137        0.000503
H       -4.304158       -2.542904        0.000897
H       -2.832807        2.185455       -0.000770
N       -0.879937        0.572200        0.000020
N       -0.035756       -0.364221       -0.000120
C        1.281443        0.033150       -0.000018
C        2.324807       -0.894446       -0.000292
C        1.913422        1.315294        0.000153
N        3.227447        1.171075       -0.000005
N        3.455553       -0.165360       -0.000340
C        4.819097       -0.626622        0.000272
H        5.335699       -0.263740        0.892320
H        4.831742       -1.716698       -0.006098
H        5.339175       -0.253230       -0.885300
C        2.258024       -2.376725       -0.000372
H        2.736238       -2.808015        0.885737
H        1.206577       -2.668965       -0.000464
H        2.736308       -2.807950       -0.886486
C        1.295148        2.666369        0.000397
H        0.651785        2.799191       -0.873988
H        0.652000        2.798972        0.874974
H        2.078520        3.427340        0.000412
H       -5.998789       -0.733139        0.000005
52
4pzMeAmid2-cis-m ; E = -1478.16512866 Hartree
```

C	2.035439	1.335751	-0.337922
C	2.148596	2.711376	-0.142908
C	0.767755	0.749886	-0.580195
H	3.117955	3.129477	0.090298
C	1.013829	3.507545	-0.263346
C	-0.347039	1.586171	-0.758175
C	-0.219331	2.966425	-0.594383
H	-1.094798	3.590823	-0.717230
N	0.808593	-0.645143	-0.826174
N	0.009082	-1.505130	-0.373996
C	-1.014627	-1.295845	0.532813
C	-1.280424	-0.399380	1.586136
C	-2.127398	-2.188712	0.516897
N	-3.023639	-1.841133	1.420100
N	-2.483844	-0.774685	2.058825
C	-3.258606	-0.097133	3.069495
H	-2.728102	-0.079011	4.025844
H	-3.471715	0.926820	2.748179
H	-4.187197	-0.654518	3.180074
C	-0.518300	0.701240	2.228536
H	-0.615067	0.649480	3.317476
H	0.539273	0.647463	1.975954
H	-0.899360	1.673370	1.894690
C	-2.337257	-3.340752	-0.398618
H	-2.480430	-3.003540	-1.429547
H	-1.462985	-3.997416	-0.394525
H	-3.219905	-3.902404	-0.087396
H	1.103568	4.579765	-0.120089
N	3.106737	0.450181	-0.277544
H	2.870499	-0.496047	-0.562771
N	-1.583643	1.031266	-1.121819
H	-1.568038	0.179055	-1.664554
C	4.410870	0.725924	0.025516
O	4.850279	1.818432	0.357052
C	-2.762342	1.323415	-0.485672
O	-2.896579	2.195957	0.364138
C	6.638545	-0.556531	0.159342
C	6.966877	-1.920482	-0.082467
C	5.802936	-2.531828	-0.437503
O	4.781354	-1.647969	-0.432424
C	5.298889	-0.441771	-0.066841
H	7.289908	0.249760	0.460796
H	7.937596	-2.387709	-0.004407
H	5.548663	-3.544249	-0.711106
C	-5.185447	0.471127	-0.447777
C	-5.828273	-0.595717	-1.135164
C	-4.881134	-1.150617	-1.940381
O	-3.702000	-0.504136	-1.803920
C	-3.894878	0.484999	-0.886570
H	-5.602637	1.148150	0.282121
H	-6.856379	-0.913288	-1.043432
H	-4.890359	-1.972275	-2.639764

52

4pzMeAmid2-cis-n ; E = -1478.16360202 Hartree

C	2.023987	1.370093	-0.136164
C	2.158129	2.685677	0.307646
C	0.746345	0.853027	-0.438948
H	3.137903	3.051646	0.580829

C	1.023920	3.486672	0.391169
C	-0.371418	1.704422	-0.417057
C	-0.230366	3.023871	0.014619
H	-1.104353	3.659505	0.059222
N	0.716250	-0.471540	-0.945063
N	-0.084758	-1.354669	-0.538616
C	-0.927175	-1.222850	0.555741
C	-2.112925	-1.965723	0.581914
C	-0.863727	-0.574320	1.836722
N	-1.938412	-0.882568	2.542790
N	-2.689232	-1.692952	1.762654
C	-3.998434	-2.091288	2.211991
H	-4.775685	-1.552818	1.659503
H	-4.140033	-3.166779	2.078083
H	-4.065163	-1.842148	3.269972
C	-2.686467	-2.878754	-0.435345
H	-3.641788	-2.512284	-0.825110
H	-1.980372	-2.966921	-1.262727
H	-2.857566	-3.875851	-0.014693
C	0.219707	0.220652	2.478348
H	1.205125	-0.189922	2.242733
H	0.208631	1.263108	2.148448
H	0.076251	0.198200	3.560377
H	1.126820	4.509789	0.738977
N	3.082527	0.475431	-0.266397
H	2.831306	-0.404422	-0.705774
N	-1.597834	1.209930	-0.876112
H	-1.560243	0.425003	-1.510893
C	4.399221	0.678930	0.042823
O	4.865188	1.693203	0.542111
C	-2.824276	1.504474	-0.344494
O	-3.036754	2.323443	0.533939
C	6.606830	-0.636204	-0.105211
C	6.905019	-1.943835	-0.582689
C	5.722076	-2.471415	-1.002905
O	4.716325	-1.587912	-0.820255
C	5.264199	-0.467501	-0.271110
H	7.279542	0.098948	0.309829
H	7.869121	-2.430004	-0.611568
H	5.443438	-3.419746	-1.435942
C	-5.242885	0.648692	-0.573481
C	-5.847535	-0.271885	-1.478407
C	-4.857747	-0.681807	-2.319218
O	-3.685412	-0.088083	-1.995256
C	-3.929041	0.725201	-0.929354
H	-5.700186	1.199255	0.234837
H	-6.882347	-0.580420	-1.509762
H	-4.829584	-1.351542	-3.164988

52

4pzMeAmid2-trans-m ; E = -1478.18685783 Hartree

C	1.349149	-2.178837	-0.029898
C	1.365650	-3.568806	0.035038
C	0.115115	-1.467658	-0.079163
H	2.313096	-4.087036	0.069428
C	0.151882	-4.245914	0.051867
C	-1.108631	-2.195212	-0.061077
C	-1.071319	-3.591624	0.007379
H	-2.004237	-4.135852	0.026719

N	0.279183	-0.090523	-0.089314
N	-0.726867	0.650943	-0.298299
C	-0.490624	2.006034	-0.196399
C	0.503999	2.701756	0.501211
C	-1.318539	3.004310	-0.777067
N	-0.869356	4.211024	-0.473883
N	0.222072	4.005329	0.299828
C	0.898410	5.147472	0.861788
H	0.707904	5.235006	1.936505
H	1.976187	5.079620	0.693190
H	0.505952	6.028380	0.356044
C	1.613917	2.227675	1.365140
H	1.833027	2.939769	2.166042
H	1.346620	1.266303	1.809415
H	2.532790	2.060924	0.792676
C	-2.488910	2.809134	-1.674033
H	-2.165404	2.587826	-2.696921
H	-3.098906	1.967888	-1.338840
H	-3.094785	3.717279	-1.700798
H	0.159305	-5.330917	0.099120
N	2.500094	-1.401570	-0.065052
H	2.308060	-0.407833	-0.138681
N	-2.295155	-1.477218	-0.079093
H	-2.163026	-0.466850	-0.184590
C	3.802126	-1.811194	-0.008803
O	4.188445	-2.965825	0.102147
C	-3.567678	-1.965313	0.070520
O	-3.884294	-3.143848	0.149807
C	6.138802	-0.741435	-0.066966
C	6.572614	0.607001	-0.212495
C	5.442794	1.356993	-0.324544
O	4.342229	0.572311	-0.257512
C	4.776710	-0.711573	-0.099806
H	6.736990	-1.632947	0.045249
H	7.588729	0.972694	-0.234329
H	5.262053	2.412825	-0.455964
C	-5.957256	-1.041565	0.258898
C	-6.469768	0.285301	0.312844
C	-5.390363	1.110602	0.227091
O	-4.250089	0.391440	0.123356
C	-4.603595	-0.924319	0.141960
H	-6.497944	-1.974862	0.302128
H	-7.502220	0.589018	0.404932
H	-5.271246	2.183297	0.226064

52

4pzMeAmid2-trans-n ; E = -1478.18769106 Hartree

C	1.606692	-2.060472	-0.063518
C	1.731104	-3.447063	-0.094121
C	0.320409	-1.445857	-0.067752
H	2.716565	-3.889736	-0.094239
C	0.576022	-4.217804	-0.126097
C	-0.840744	-2.269835	-0.102790
C	-0.694752	-3.659866	-0.128747
H	-1.581981	-4.275588	-0.148250
N	0.373424	-0.060936	0.008001
N	-0.689414	0.605730	-0.166452
C	-0.563354	1.970989	-0.010082
C	-1.509531	2.873656	-0.497193

```
C        0.412842        2.769159        0.660337
N        0.081280        4.047960        0.584156
N       -1.068989        4.091256       -0.126743
C       -1.669535        5.367222       -0.418714
H       -1.669016        5.566519       -1.494997
H       -2.698483        5.408853       -0.049257
H       -1.072496        6.123039        0.089249
C       -2.729447        2.651648       -1.310637
H       -2.600816        3.017981       -2.335764
H       -2.950326        1.585052       -1.353963
H       -3.596883        3.168419       -0.885353
C        1.621636        2.347619        1.416554
H        1.401654        1.490630        2.059672
H        2.428772        2.040995        0.745860
H        1.974154        3.179571        2.030256
H        0.669373       -5.299527       -0.151994
N        2.696944       -1.200634       -0.045958
H        2.434617       -0.220550       -0.033308
N       -2.083212       -1.650905       -0.077852
H       -2.031543       -0.628574       -0.106155
C        4.026465       -1.520976       -0.050581
O        4.492028       -2.651671       -0.041620
C       -3.311174       -2.250448        0.015845
O       -3.532710       -3.453221        0.010457
C        6.284279       -0.291897       -0.052502
C        6.621171        1.090792       -0.096504
C        5.440384        1.766412       -0.141286
O        4.399550        0.902946       -0.128628
C        4.922732       -0.354824       -0.072331
H        6.944350       -1.145047       -0.011625
H        7.608959        1.527752       -0.095656
H        5.182673        2.813565       -0.183288
C       -5.771872       -1.550235        0.231001
C       -6.400856       -0.279179        0.354841
C       -5.398562        0.641259        0.327109
O       -4.197655        0.033828        0.194293
C       -4.433612       -1.307127        0.135955
H       -6.227139       -2.528821        0.215551
H       -7.456616       -0.073894        0.454717
H       -5.377528        1.717948        0.393744
30
4pzMeCl1A-cis-m ; E = -1143.34071795 Hartree
C       -2.484252       -0.156319       -0.115080
C       -3.238630        0.889535        0.404490
C       -1.302573       -0.575209        0.504786
H       -4.141719        1.198905       -0.110471
C       -2.826363        1.518746        1.573523
C       -0.906599        0.064122        1.685743
H       -0.002482       -0.273007        2.182009
C       -1.661332        1.100659        2.215150
H       -1.342336        1.581141        3.134809
Cl       -2.980206       -0.915692       -1.593693
N       -0.660712       -1.758318        0.055273
N        0.575101       -1.834499       -0.112681
C        1.434216       -0.734452       -0.112750
C        1.325486        0.550346       -0.657414
C        2.778005       -0.841009        0.337377
N        3.428805        0.292431        0.136198
```

```
N        2.541558       1.108892       -0.474919
C        2.988065       2.403570       -0.926078
H        3.085292       2.433625       -2.016374
H        2.295855       3.185897       -0.604897
H        3.964638       2.574647       -0.475837
C        0.233789       1.230357       -1.399138
H        0.633512       1.933254       -2.135074
H       -0.374711       0.496747       -1.932830
H       -0.433878       1.777891       -0.723788
C        3.410244      -2.013358        0.998203
H        3.107167      -2.094294        2.047713
H        3.100774      -2.936298        0.500055
H        4.497345      -1.922325        0.962545
H       -3.419543       2.329098        1.984961
30
4pzMeCl1A-cis-n ; E = -1143.34158459 Hartree
C        2.617707      -0.232942        0.006436
C        3.427454       0.894665        0.067394
C        1.406370      -0.214660       -0.692132
H        4.350682       0.854617        0.635239
C        3.042868       2.054190       -0.597329
C        1.038825       0.956431       -1.364118
H        0.110101       0.964101       -1.925216
C        1.849867       2.081374       -1.317165
H        1.551203       2.981738       -1.844833
Cl        3.078495      -1.666860        0.865236
N        0.690451      -1.420757       -0.884228
N       -0.542164      -1.510411       -0.703557
C       -1.336075      -0.545360       -0.081052
C       -2.697308      -0.504012       -0.391653
C       -1.173069       0.376392        1.004516
N       -2.337404       0.937412        1.293616
N       -3.234455       0.416381        0.427250
C       -4.602764       0.864772        0.472755
H       -4.878777       1.381073       -0.451858
H       -5.283633       0.022937        0.628113
H       -4.683556       1.554703        1.311362
C       -3.451843      -1.272221       -1.410470
H       -3.718109      -0.655898       -2.276956
H       -2.821117      -2.093534       -1.757138
H       -4.378302      -1.684902       -0.998235
C        0.007539       0.681048        1.859183
H        0.553922      -0.229938        2.119728
H        0.710229       1.353894        1.358499
H       -0.338433       1.158911        2.777845
H        3.678955       2.932481       -0.555317
30
4pzMeCl1A-trans-m ; E = -1143.36111639 Hartree
C       -2.819880      -0.634036        0.000209
C       -4.173094      -0.311512        0.000291
C       -1.836812       0.366461       -0.000315
H       -4.908281      -1.108636        0.000732
C       -4.561785       1.022137       -0.000205
C       -2.253249       1.705165       -0.000819
H       -1.480154       2.466103       -0.001255
C       -3.598188       2.031636       -0.000764
H       -3.901943       3.073999       -0.001189
Cl       -2.364770      -2.310294        0.000845
```

```
N       -0.493406       -0.043430       -0.000489
N        0.337694        0.907652        0.000204
C        1.654758        0.522053        0.000039
C        2.240045       -0.754548       -0.000440
C        2.740785        1.438906        0.000500
N        3.892786        0.792689        0.000332
N        3.565111       -0.526583       -0.000250
C        4.632346       -1.497432       -0.000367
H        5.564796       -0.935365       -0.000682
H        4.589761       -2.128989        0.891530
H        4.589270       -2.129196       -0.892081
C        1.622767       -2.102473       -0.000863
H        0.969988       -2.221084        0.868768
H        0.970770       -2.220955       -0.871096
H        2.374700       -2.895193       -0.000571
C        2.678640        2.924643        0.001020
H        2.145266        3.294150       -0.879919
H        2.142765        3.293404        0.880734
H        3.689192        3.336918        0.002549
H       -5.618339        1.271538       -0.000178
30
4pzMeCl1A-trans-n ; E = -1143.36175490 Hartree
C        2.939574       -0.442611        0.000708
C        4.240014        0.052216        0.000725
C        1.836128        0.424011       -0.000501
H        5.071326       -0.644083        0.001725
C        4.454904        1.424519       -0.000557
C        2.078279        1.804750       -0.001817
H        1.215352        2.461765       -0.002836
C        3.369924        2.302001       -0.001842
H        3.537198        3.374793       -0.002923
Cl        2.704950       -2.161781        0.002331
N        0.556664       -0.157037       -0.000642
N       -0.390947        0.676220        0.000172
C       -1.646587        0.121495       -0.000197
C       -2.800085        0.909768        0.000541
C       -2.108855       -1.232466       -0.001533
N       -3.429842       -1.255752       -0.001637
N       -3.826719        0.042259       -0.000577
C       -5.238466        0.324214        0.001639
H       -5.701854       -0.099108        0.896228
H       -5.391833        1.403454       -0.009006
H       -5.706738       -0.117448       -0.881290
C       -2.923621        2.388310        0.002280
H       -3.453330        2.753076        0.888903
H       -1.918978        2.814366        0.002498
H       -3.453727        2.755144       -0.883271
C       -1.318368       -2.489681       -0.002877
H       -0.659899       -2.534236       -0.875069
H       -0.659740       -2.535972        0.869095
H       -1.993884       -3.347709       -0.003703
H        5.470810        1.807246       -0.000599
30
4pzMeCl1B-cis-m ; E = -1143.33848826 Hartree
C       -2.607504        0.240785       -1.348653
C       -3.561580        0.904634       -0.593562
C       -1.590769       -0.507738       -0.743907
H       -4.325387        1.496962       -1.087416
```

C	-3.544796	0.792933	0.795358
C	-1.615532	-0.650221	0.650944
Cl	-0.490446	-1.685155	1.471308
C	-2.582139	0.003069	1.411983
H	-2.573766	-0.125076	2.489179
H	-2.613372	0.292245	-2.433260
N	-0.730316	-1.231294	-1.613491
N	0.516599	-1.162587	-1.561818
C	1.234183	-0.281652	-0.758200
C	1.065873	1.058471	-0.387281
C	2.496751	-0.663403	-0.226537
N	3.040530	0.332377	0.449807
N	2.171525	1.360737	0.327561
C	2.535051	2.640897	0.882249
H	2.786156	3.360474	0.095763
H	1.723762	3.043885	1.493666
H	3.411530	2.474478	1.506509
C	0.044739	2.083445	-0.727220
H	0.486141	3.083429	-0.754010
H	-0.382944	1.879217	-1.709501
H	-0.782205	2.089711	-0.008151
C	3.133007	-2.002750	-0.319842
H	2.646097	-2.715585	0.354112
H	3.032736	-2.398387	-1.334200
H	4.188703	-1.936881	-0.050609
H	-4.291947	1.297724	1.399284

30

4pzMeCl1B-cis-n ; E = -1143.34017951 Hartree

C	-2.760390	-0.465524	1.251107
C	-3.748291	-0.910934	0.387205
C	-1.706331	0.334674	0.796429
H	-4.541097	-1.552806	0.758065
C	-3.726094	-0.521395	-0.950722
C	-1.721634	0.754869	-0.540692
Cl	-0.532140	1.866253	-1.146245
C	-2.722046	0.323418	-1.407792
H	-2.704688	0.661697	-2.438262
H	-2.766396	-0.738434	2.301824
N	-0.804491	0.830792	1.772124
N	0.433805	0.681863	1.699941
C	1.107531	-0.095861	0.762730
C	2.419110	0.266659	0.444660
C	0.880056	-1.324693	0.059530
N	1.961923	-1.649584	-0.631769
N	2.864479	-0.668791	-0.409184
C	4.129481	-0.707713	-1.097066
H	4.176006	0.054666	-1.881238
H	4.953719	-0.550693	-0.396558
H	4.218559	-1.695150	-1.547749
C	3.193344	1.450679	0.885235
H	3.256446	2.209413	0.096867
H	2.681756	1.895008	1.741377
H	4.214052	1.182417	1.175940
C	-0.259425	-2.284238	0.081973
H	-0.620186	-2.449762	1.100223
H	-1.106841	-1.930635	-0.511976
H	0.081801	-3.236025	-0.330047
H	-4.498643	-0.855610	-1.635677

```
30
4pzMeCl1B-trans-m ; E = -1143.35362662 Hartree
C       -2.571403       -1.752212        0.546774
C       -3.952292       -1.769805        0.657787
C       -1.878952       -0.611985        0.120120
H       -4.458096       -2.662628        1.011271
C       -4.681589       -0.638932        0.300601
C       -2.632174        0.514278       -0.257907
Cl       -1.909189        1.942772       -0.931438
C       -4.021548        0.490522       -0.168186
H       -4.578306        1.369137       -0.475935
H       -1.971967       -2.620317        0.802900
N       -0.480747       -0.766121        0.052441
N        0.175741        0.278182        0.314895
C        1.541072        0.156093        0.216153
C        2.354319       -0.911820       -0.191727
C        2.433664        1.216358        0.529347
N        3.685620        0.841038        0.336663
N        3.612060       -0.444958       -0.096824
C        4.840271       -1.132734       -0.412260
H        5.651250       -0.445922       -0.175542
H        4.946911       -2.041031        0.187245
H        4.885821       -1.393445       -1.473803
C        2.005527       -2.278124       -0.651801
H        1.557897       -2.855136        0.163359
H        1.245438       -2.225128       -1.435348
H        2.878441       -2.816513       -1.028870
C        2.086436        2.578494        1.013912
H        1.344080        3.043514        0.359632
H        1.648538        2.537238        2.016066
H        2.982364        3.200935        1.046858
H       -5.764859       -0.635602        0.369527
30
4pzMeCl1B-trans-n ; E = -1143.35582843 Hartree
C       -2.780296        1.618789        0.469964
C       -4.147984        1.473054        0.635340
C       -1.962952        0.547578        0.086607
H       -4.750364        2.318438        0.952126
C       -4.740445        0.239606        0.378761
C       -2.581184       -0.685615       -0.189517
Cl       -1.709314       -2.059611       -0.801034
C       -3.958699       -0.828199       -0.045150
H       -4.409935       -1.787386       -0.275471
H       -2.286309        2.569311        0.645678
N       -0.599482        0.875053       -0.042694
N        0.195074       -0.064889        0.227142
C        1.528979        0.230528        0.084686
C        2.510265       -0.725059        0.356871
C        2.239082        1.403196       -0.319717
N        3.539940        1.173374       -0.291425
N        3.682684       -0.113095        0.117622
C        5.013732       -0.648003        0.239363
H        5.583649       -0.067376        0.968885
H        4.956559       -1.685803        0.568087
H        5.523463       -0.600687       -0.725976
C        2.341382       -2.128534        0.807156
H        2.814938       -2.305549        1.778837
H        1.272122       -2.328052        0.899914
```

H   2.764564   -2.839638   0.089612
C   1.704733   2.727424   -0.729503
H   1.034600   2.626466   -1.587943
H   1.108363   3.170873   0.073075
H   2.531431   3.394081   -0.983797
H   -5.811933   0.107009   0.490879
30
4pzMeCl2-cis-m ; E = -1602.77490089 Hartree
C   2.468649   -0.367725   -0.280356
C   3.289523   -0.272139   0.833776
C   1.326722   0.431277   -0.440258
H   4.149636   -0.926525   0.919501
C   2.992098   0.663696   1.818075
C   1.072399   1.386680   0.557044
Cl   -0.244245   2.502176   0.393683
C   1.889308   1.496838   1.677488
H   1.656635   2.246757   2.425225
Cl   2.824184   -1.553451   -1.495137
N   0.648683   0.401961   -1.685457
N   -0.576347   0.187008   -1.796952
C   -1.408899   -0.215577   -0.761426
C   -1.258733   -1.084627   0.328483
C   -2.774444   0.183104   -0.741278
N   -3.401145   -0.351032   0.290782
N   -2.476579   -1.120266   0.909798
C   -2.890923   -1.928838   2.029759
H   -2.940862   -2.988850   1.759571
H   -2.207031   -1.803172   2.872710
H   -3.884502   -1.585753   2.313762
C   -0.142684   -1.952786   0.784226
H   -0.522236   -2.857292   1.266954
H   0.468616   -2.261959   -0.065807
H   0.514251   -1.435885   1.492772
C   -3.441076   1.125465   -1.676563
H   -3.150221   2.159763   -1.464531
H   -3.141544   0.910930   -2.705923
H   -4.525320   1.044166   -1.581744
H   3.629697   0.753610   2.691205
30
4pzMeCl2-cis-n ; E = -1602.77645797 Hartree
C   2.574859   -0.517274   -0.103579
C   3.461171   0.075271   0.783426
C   1.428398   0.144834   -0.564737
H   4.321740   -0.485031   1.130959
C   3.225471   1.376820   1.212912
C   1.234084   1.465828   -0.134142
Cl   -0.101367   2.396533   -0.735389
C   2.116973   2.075306   0.750936
H   1.928829   3.096199   1.063730
Cl   2.846606   -2.151225   -0.614551
N   0.667289   -0.447469   -1.600579
N   -0.554034   -0.686479   -1.505580
C   -1.320294   -0.572369   -0.353578
C   -2.693219   -0.358142   -0.514354
C   -1.129751   -0.725977   1.060784
N   -2.291175   -0.593680   1.681825
N   -3.211883   -0.347645   0.722443
C   -4.575180   -0.085074   1.107733

H	-4.823122	0.973743	0.983099
H	-5.265546	-0.686882	0.511367
H	-4.669289	-0.357016	2.158066
C	-3.464747	-0.135410	-1.759473
H	-3.748509	0.916602	-1.876765
H	-2.835469	-0.413525	-2.607053
H	-4.380646	-0.734614	-1.779979
C	0.062870	-1.106823	1.867988
H	0.614228	-1.927433	1.401099
H	0.756363	-0.270891	1.996584
H	-0.276895	-1.428762	2.854294
H	3.914057	1.852355	1.903315

30

4pzMeCl2-trans-m ; E = -1602.78838145 Hartree

C	-2.546210	-1.155109	0.253382
C	-3.923756	-1.013403	0.331489
C	-1.697215	-0.080622	-0.057761
H	-4.533922	-1.869480	0.595569
C	-4.493305	0.226446	0.067156
C	-2.307900	1.156706	-0.329580
Cl	-1.376852	2.532604	-0.832726
C	-3.689460	1.306869	-0.271769
H	-4.122596	2.274406	-0.499079
Cl	-1.847371	-2.709113	0.590381
N	-0.327582	-0.356310	-0.180944
N	0.408322	0.501182	0.350529
C	1.750147	0.275098	0.214740
C	2.459952	-0.782787	-0.378865
C	2.735547	1.167022	0.715694
N	3.945506	0.711077	0.452885
N	3.754978	-0.465312	-0.203531
C	4.938205	-1.180818	-0.612140
H	5.541490	-1.420742	0.266256
H	4.661526	-2.100618	-1.124389
H	5.527609	-0.554971	-1.286015
C	1.945345	-2.001440	-1.050063
H	1.206160	-2.489502	-0.409872
H	1.412271	-1.731422	-1.966881
H	2.736215	-2.712381	-1.296424
C	2.515958	2.447881	1.437346
H	1.861940	3.109278	0.862553
H	2.027077	2.272409	2.400481
H	3.471848	2.944996	1.610751
H	-5.570243	0.349159	0.116569

30

4pzMeCl2-trans-n ; E = -1602.79095332 Hartree

C	-2.706732	0.966382	0.294741
C	-4.049104	0.651167	0.446229
C	-1.744124	0.002651	-0.048509
H	-4.749932	1.427444	0.731173
C	-4.467852	-0.655605	0.226987
C	-2.204113	-1.307312	-0.270607
Cl	-1.130742	-2.564576	-0.801225
C	-3.550381	-1.631459	-0.139149
H	-3.866889	-2.650411	-0.331443
Cl	-2.194970	2.600836	0.577733
N	-0.427025	0.449559	-0.250390
N	0.452774	-0.297581	0.254155

C	1.749562	0.094302	0.037288
C	2.822339	-0.655632	0.525535
C	2.335523	1.211643	-0.635506
N	3.651846	1.140375	-0.558098
N	3.924751	0.008476	0.142536
C	5.303678	-0.330303	0.381135
H	5.789598	0.466859	0.948904
H	5.354073	-1.260657	0.947002
H	5.825438	-0.455223	-0.570683
C	2.797720	-1.922116	1.297621
H	3.269407	-1.809885	2.279675
H	1.755166	-2.210400	1.443888
H	3.308943	-2.732051	0.766501
C	1.664358	2.336034	-1.336253
H	1.054972	1.966503	-2.166120
H	0.976747	2.856223	-0.663536
H	2.414420	3.034025	-1.713732
H	-5.516517	-0.913158	0.333244

30
4pzMeF1A-cis-m ; E = -783.046043499 Hartree

C	-2.598813	-0.215390	-0.571423
C	-3.467670	0.613072	0.117358
C	-1.475726	-0.782233	0.031219
H	-4.314355	1.041235	-0.408326
C	-3.228643	0.867993	1.464920
C	-1.253837	-0.518219	1.386395
H	-0.396435	-0.971360	1.873251
C	-2.123530	0.300161	2.096666
H	-1.941714	0.489101	3.149860
F	-2.795763	-0.436377	-1.877610
N	-0.740314	-1.765684	-0.686112
N	0.508242	-1.755578	-0.740716
C	1.289505	-0.673936	-0.326286
C	1.149336	0.701813	-0.529629
C	2.575906	-0.841969	0.251588
N	3.160013	0.330705	0.436739
N	2.291720	1.242989	-0.052342
C	2.687576	2.628337	-0.094793
H	2.915122	2.946711	-1.117678
H	1.901159	3.267936	0.313851
H	3.584840	2.720024	0.515363
C	0.100252	1.504934	-1.207210
H	0.535577	2.351242	-1.746426
H	-0.441441	0.888794	-1.927952
H	-0.635478	1.891974	-0.492248
C	3.213124	-2.120424	0.664854
H	2.790942	-2.494788	1.603803
H	3.042788	-2.888011	-0.095304
H	4.285699	-1.975717	0.806327
H	-3.907337	1.508396	2.019033

30
4pzMeF1A-cis-n ; E = -783.046721669 Hartree

C	-2.772236	0.472286	0.383319
C	-3.672640	-0.534991	0.084504
C	-1.591707	0.649349	-0.339387
H	-4.562680	-0.642828	0.694951
C	-3.408506	-1.379488	-0.990692
C	-1.347739	-0.200771	-1.422084

```
H       -0.445687       -0.054859       -2.007184
C       -2.249077       -1.208202       -1.744457
H       -2.047830       -1.858630       -2.589644
F       -3.001363        1.269986        1.432162
N       -0.804881        1.801848       -0.086796
N        0.437758        1.748556        0.033301
C        1.175288        0.570193        0.175964
C        2.508523        0.552093       -0.236261
C        0.978968       -0.672165        0.860108
N        2.097204       -1.381812        0.831961
N        2.995532       -0.642212        0.145510
C        4.316235       -1.169253       -0.084739
H        4.500360       -1.317227       -1.153344
H        5.079944       -0.498133        0.318959
H        4.369827       -2.128100        0.428619
C        3.281846        1.587928       -0.962111
H        3.440500        1.322791       -2.013855
H        2.718972        2.523210       -0.927959
H        4.264778        1.748819       -0.507637
C       -0.188082       -1.182013        1.631857
H       -0.665478       -0.381023        2.203254
H       -0.948843       -1.615121        0.974825
H        0.154633       -1.954147        2.323620
H       -4.110756       -2.169077       -1.238014
30
4pzMeF1A-trans-m ; E = -783.066407739 Hartree
C       -2.914792       -1.002776       -0.000028
C       -4.285877       -0.801279       -0.000015
C       -2.008510        0.063549       -0.000001
H       -4.944064       -1.663195       -0.000020
C       -4.775156        0.500133        0.000010
C       -2.524670        1.366091        0.000019
H       -1.815310        2.186675        0.000035
C       -3.893344        1.582919        0.000017
H       -4.280315        2.597146        0.000033
F       -2.446955       -2.255317       -0.000010
N       -0.645966       -0.274672        0.000009
N        0.132012        0.717616        0.000004
C        1.476896        0.419147        0.000003
C        2.178613       -0.796191        0.000004
C        2.476391        1.431285        0.000001
N        3.684510        0.896153       -0.000007
N        3.477999       -0.444815       -0.000010
C        4.615577       -1.328585        0.000019
H        5.505599       -0.701396        0.000073
H        4.616692       -1.964729        0.890390
H        4.616770       -1.964684       -0.890384
C        1.731634       -2.212968        0.000002
H        2.104236       -2.744613        0.883673
H        0.642929       -2.245398       -0.000060
H        2.104354       -2.744650       -0.883595
C        2.275996        2.905030       -0.000023
H        1.709432        3.223243       -0.880224
H        1.709365        3.223256        0.880130
H        3.244014        3.409264        0.000007
H       -5.847552        0.668834        0.000017
30
4pzMeF1A-trans-n ; E = -783.067189005 Hartree
```

C	3.052979	-0.807998	0.000375
C	4.389003	-0.438302	0.000472
C	2.021638	0.138552	-0.000107
H	5.147648	-1.213289	0.000877
C	4.715571	0.912893	0.000041
C	2.375895	1.494244	-0.000555
H	1.572872	2.223153	-0.000975
C	3.707276	1.878726	-0.000482
H	3.966140	2.933009	-0.000852
F	2.745474	-2.107350	0.000795
N	0.709667	-0.364935	-0.000221
N	-0.182496	0.527672	-0.000044
C	-1.472156	0.052214	-0.000109
C	-2.573392	0.910661	-0.000110
C	-2.018607	-1.269294	-0.000369
N	-3.338877	-1.210323	-0.000519
N	-3.653538	0.109253	-0.000331
C	-5.044461	0.479972	0.000924
H	-5.535211	0.084938	0.893900
H	-5.129009	1.566831	-0.007013
H	-5.539231	0.071543	-0.883641
C	-2.603414	2.394134	0.000342
H	-3.109076	2.792848	0.886444
H	-1.573540	2.755100	0.000591
H	-3.108741	2.793419	-0.885712
C	-1.309358	-2.574605	-0.000600
H	-0.655748	-2.661942	-0.873084
H	-0.655644	-2.662208	0.871776
H	-2.038046	-3.388017	-0.000686
H	5.759179	1.211916	0.000097

30

4pzMeF1B-cis-m ; E = -783.046231322 Hartree

C	-2.722297	-0.149107	-1.111189
C	-3.626462	0.608964	-0.378490
C	-1.605889	-0.730481	-0.503564
H	-4.473743	1.069163	-0.876528
C	-3.452948	0.759338	0.995643
C	-1.478039	-0.597423	0.883434
F	-0.471700	-1.211630	1.516695
C	-2.378317	0.143271	1.630868
H	-2.226728	0.216560	2.702577
H	-2.849765	-0.299627	-2.178956
N	-0.786264	-1.584278	-1.292343
N	0.463690	-1.536237	-1.254552
C	1.176500	-0.533647	-0.599333
C	1.022375	0.854442	-0.573367
C	2.386580	-0.794122	0.095833
N	2.914373	0.330296	0.549515
N	2.087241	1.310012	0.124488
C	2.453583	2.682725	0.365136
H	2.800986	3.170940	-0.551837
H	1.608106	3.243192	0.772117
H	3.264614	2.670769	1.091747
C	0.040986	1.761744	-1.222013
H	0.514839	2.693905	-1.543081
H	-0.386874	1.280127	-2.102879
H	-0.791156	2.008631	-0.552343
C	2.983975	-2.125101	0.378176

```
H        2.413673       -2.653106        1.149683
H        2.965710       -2.747532       -0.521023
H        4.012615       -2.010015        0.724477
H       -4.160543        1.339492        1.579225
30
4pzMeF1B-cis-n ; E = -783.047470797 Hartree
C       -2.900640        0.420999        0.999766
C       -3.838196       -0.413584        0.406496
C       -1.731185        0.785968        0.326949
H       -4.726073       -0.706364        0.957494
C       -3.644100       -0.859386       -0.899497
C       -1.579650        0.355978       -0.995492
F       -0.512601        0.753317       -1.700954
C       -2.514099       -0.462018       -1.608252
H       -2.342013       -0.771416       -2.633639
H       -3.039806        0.797718        2.008361
N       -0.869788        1.728173        0.945403
N        0.373012        1.586473        0.974856
C        1.049109        0.429595        0.594681
C        2.337319        0.537021        0.071557
C        0.821263       -0.975435        0.744512
N        1.881972       -1.648707        0.323108
N        2.769491       -0.724510       -0.103104
C        4.015650       -1.157130       -0.681408
H        4.056620       -0.923975       -1.750089
H        4.862466       -0.680714       -0.179628
H        4.073298       -2.235866       -0.543863
C        3.106899        1.753694       -0.280401
H        3.121222        1.928138       -1.362360
H        2.627136        2.612779        0.193402
H        4.144357        1.690414        0.063161
C       -0.304640       -1.720111        1.375245
H       -0.656373       -1.213017        2.277641
H       -1.161776       -1.817629        0.702162
H        0.041844       -2.720005        1.644314
H       -4.376694       -1.503478       -1.375125
30
4pzMeF1B-trans-m ; E = -783.061798528 Hartree
C       -2.760766       -1.559253        0.190117
C       -4.147296       -1.547412        0.201220
C       -2.008400       -0.387434        0.031639
H       -4.698570       -2.472673        0.333184
C       -4.823481       -0.340835        0.037777
C       -2.720119        0.814510       -0.137449
F       -2.085512        1.972071       -0.323260
C       -4.106056        0.838327       -0.135985
H       -4.601800        1.793413       -0.274142
H       -2.201471       -2.482003        0.309214
N       -0.617304       -0.578589        0.037222
N        0.078835        0.476055        0.064667
C        1.438513        0.273730        0.048449
C        2.209741       -0.894537       -0.045081
C        2.374238        1.341063        0.114502
N        3.611596        0.879453        0.066878
N        3.486635       -0.469785       -0.028342
C        4.687474       -1.264324       -0.112549
H        5.524399       -0.571420       -0.042488
H        4.742099       -1.983356        0.709709
```

```
H        4.740982       -1.800647       -1.064636
C        1.809939       -2.319220       -0.152158
H        1.283653       -2.637973        0.752676
H        1.101294       -2.448960       -0.974175
H        2.670982       -2.973101       -0.310863
C        2.079970        2.793794        0.233313
H        1.317725        3.093389       -0.490563
H        1.688406        3.033045        1.227276
H        2.989674        3.373821        0.067839
H       -5.908631       -0.312572        0.040556
30
4pzMeF1B-trans-n ; E = -783.064215244 Hartree
C       -2.980525        1.394155       -0.001483
C       -4.353627        1.200900       -0.001937
C       -2.077918        0.321821       -0.000056
H       -5.023342        2.054640       -0.003132
C       -4.863201       -0.095140       -0.000777
C       -2.623877       -0.975269        0.001158
F       -1.841919       -2.057487        0.002903
C       -3.994332       -1.181721        0.000826
H       -4.358180       -2.203835        0.001858
H       -2.547669        2.389605       -0.002269
N       -0.725314        0.700449        0.000290
N        0.105273       -0.250609       -0.000534
C        1.426110        0.126120       -0.000165
C        2.442132       -0.831642       -0.001065
C        2.094114        1.389749        0.000716
N        3.403283        1.207482        0.000409
N        3.592861       -0.136216       -0.000809
C        4.942229       -0.637305        0.000022
H        5.467976       -0.294001        0.894497
H        4.921658       -1.727242       -0.011288
H        5.474438       -0.275535       -0.883121
C        2.318782       -2.310112       -0.001876
H        2.780668       -2.759382        0.883948
H        1.255349       -2.557911       -0.002098
H        2.780637       -2.758449       -0.888214
C        1.513661        2.757529        0.001801
H        0.874928        2.908820       -0.873091
H        0.874785        2.907355        0.876847
H        2.317207        3.497116        0.002521
H       -5.935188       -0.266049       -0.001065
30
4pzMeF2-cis-m ; E = -882.188736999 Hartree
C       -2.541735        0.170533       -0.675012
C       -3.394266        0.683829        0.287075
C       -1.432871       -0.618154       -0.364758
H       -4.224331        1.309305       -0.020743
C       -3.157247        0.371554        1.622772
C       -1.242345       -0.917471        0.988059
F       -0.223382       -1.711756        1.330354
C       -2.078155       -0.431735        1.979774
H       -1.877332       -0.699427        3.010892
F       -2.733768        0.484966       -1.961194
N       -0.724080       -1.271244       -1.412679
N        0.524606       -1.245289       -1.468682
C        1.295662       -0.400590       -0.673561
C        1.158042        0.960155       -0.392515
```

```
C        2.538464       -0.784657       -0.105773
N        3.101418        0.240398        0.511782
N        2.264342        1.282346        0.314510
C        2.658811        2.591472        0.769091
H        2.983133        3.223303       -0.064875
H        1.833684        3.082342        1.291271
H        3.492243        2.452057        1.456047
C        0.155126        1.967725       -0.822108
H        0.635269        2.911226       -1.099251
H       -0.399712        1.604140       -1.689207
H       -0.575547        2.174825       -0.031140
C        3.133925       -2.146059       -0.096310
H        2.581491       -2.807664        0.579039
H        3.086485       -2.589329       -1.095228
H        4.172996       -2.099436        0.234192
H       -3.818507        0.756678        2.391633
30
4pzMeF2-cis-n ; E = -882.189401241 Hartree
C       -2.732279       -0.488626       -0.467928
C       -3.622972        0.549669       -0.260390
C       -1.549512       -0.630506        0.261359
H       -4.509285        0.617948       -0.880596
C       -3.347605        1.476176        0.741809
C       -1.323906        0.312012        1.269169
F       -0.232728        0.185977        2.032526
C       -2.195520        1.360608        1.513536
H       -1.963037        2.061108        2.307501
F       -2.963567       -1.368338       -1.446176
N       -0.785707       -1.817576        0.117034
N        0.456243       -1.789125       -0.026703
C        1.187672       -0.634762       -0.286118
C        2.504684       -0.535709        0.161752
C        0.978749        0.521580       -1.101833
N        2.077750        1.261324       -1.119521
N        2.972029        0.624081       -0.332922
C        4.259500        1.226752       -0.099532
H        4.347177        1.584034        0.931417
H        5.062390        0.512124       -0.299287
H        4.345999        2.070027       -0.783150
C        3.269297       -1.457110        1.035181
H        3.351085       -1.074213        2.058821
H        2.741949       -2.412650        1.073489
H        4.282847       -1.625548        0.657621
C       -0.175438        0.905912       -1.961875
H       -0.655491        0.025663       -2.398107
H       -0.938848        1.449574       -1.395976
H        0.179807        1.551627       -2.767578
H       -4.036158        2.294648        0.922703
30
4pzMeF2-trans-m ; E = -882.203494870 Hartree
C       -2.709970       -1.251482       -0.081650
C       -4.091564       -1.189299       -0.105679
C       -1.885841       -0.116776        0.013539
H       -4.660442       -2.107794       -0.190045
C       -4.702973        0.056973       -0.018554
C       -2.553970        1.119006        0.103809
F       -1.857830        2.246210        0.227987
C       -3.936628        1.212580        0.090185
```

H	-4.388637	2.194930	0.168100
F	-2.122339	-2.447869	-0.163865
N	-0.513182	-0.375120	0.049836
N	0.240897	0.631123	-0.069813
C	1.589065	0.354421	-0.032598
C	2.312762	-0.845318	0.053948
C	2.569844	1.382864	-0.100267
N	3.787207	0.871673	-0.056131
N	3.605057	-0.470450	0.036325
C	4.759881	-1.330682	0.078887
H	5.634627	-0.685952	0.147649
H	4.720202	-1.989670	0.950824
H	4.829920	-1.942403	-0.826346
C	1.890669	-2.267148	0.139126
H	2.121822	-2.687170	1.125852
H	0.814140	-2.333129	-0.013149
H	2.406609	-2.880159	-0.608341
C	2.337485	2.848268	-0.198797
H	1.739569	3.088909	-1.082518
H	1.783455	3.215531	0.670142
H	3.293285	3.371789	-0.258984
H	-5.785448	0.129024	-0.033492

30

4pzMeF2-trans-n ; E = -882.205206512 Hartree

C	-2.892762	1.048922	-0.125147
C	-4.252092	0.791910	-0.154822
C	-1.919081	0.043902	0.008620
H	-4.942630	1.619103	-0.270268
C	-4.684636	-0.524075	-0.033007
C	-2.409770	-1.269727	0.132732
F	-1.565305	-2.287536	0.297215
C	-3.765199	-1.557214	0.115098
H	-4.075879	-2.590466	0.221355
F	-2.478545	2.310997	-0.240644
N	-0.593766	0.489986	0.046516
N	0.283732	-0.412952	-0.061471
C	1.578495	0.041567	-0.010062
C	2.657614	-0.838023	-0.110594
C	2.156919	1.342648	0.123053
N	3.475279	1.252587	0.102983
N	3.757050	-0.068580	-0.042618
C	5.132275	-0.494734	-0.045597
H	5.423641	-0.907217	0.926264
H	5.293524	-1.251515	-0.817226
H	5.743353	0.381038	-0.260359
C	2.656516	-2.313150	-0.258881
H	3.171723	-2.805373	0.573261
H	1.619766	-2.654413	-0.278397
H	3.145911	-2.628320	-1.187328
C	1.478896	2.657460	0.261768
H	0.790586	2.831047	-0.569956
H	0.866544	2.684271	1.167922
H	2.225250	3.453780	0.300158
H	-5.746256	-0.747279	-0.051406

34

4pzMeOMe1A-cis-m ; E = -798.306503177 Hartree

C	-2.391399	-0.208933	-0.124109
C	-3.153075	0.534140	0.775891

C	-1.192972	-0.811408	0.301792
H	-4.072972	1.009562	0.455588
C	-2.730537	0.668783	2.097731
C	-0.786829	-0.665710	1.626866
C	-1.550411	0.071922	2.525797
H	-1.227563	0.169105	3.557312
N	-0.554009	-1.729436	-0.575761
N	0.676228	-1.702555	-0.793403
C	1.504076	-0.642028	-0.408140
C	1.335981	0.742008	-0.492353
C	2.855963	-0.835156	-0.020190
N	3.459523	0.328056	0.166598
N	2.531686	1.262266	-0.134825
C	2.913787	2.651487	-0.127857
H	2.991693	3.049547	-1.145330
H	2.192804	3.248765	0.436386
H	3.889052	2.709042	0.353234
C	0.199629	1.576513	-0.956900
H	0.551647	2.471890	-1.477804
H	-0.429053	1.006576	-1.645119
H	-0.437390	1.888710	-0.120632
C	3.544816	-2.132460	0.212693
H	3.254938	-2.575001	1.172033
H	3.272494	-2.847601	-0.568647
H	4.626717	-1.987292	0.216715
H	-3.335143	1.244690	2.791675
H	0.129224	-1.152164	1.946469
O	-2.679618	-0.361598	-1.438101
C	-3.900987	0.162681	-1.905264
H	-4.755411	-0.266958	-1.367929
H	-3.932838	1.256211	-1.814512
H	-3.963498	-0.111649	-2.958632

34

4pzMeOMe1A-cis-n ; E = -798.307030260 Hartree

C	2.538774	-0.227939	-0.116983
C	3.335706	0.902945	0.047987
C	1.305241	-0.121279	-0.786947
H	4.280795	0.836928	0.574399
C	2.914741	2.131119	-0.461257
C	0.903263	1.112680	-1.293387
C	1.702530	2.240152	-1.131958
H	1.381041	3.194784	-1.535615
N	0.611350	-1.314471	-1.111717
N	-0.614662	-1.451546	-0.914265
C	-1.396517	-0.580717	-0.145781
C	-2.760125	-0.471494	-0.416201
C	-1.198271	0.144560	1.071355
N	-2.347146	0.665154	1.478409
N	-3.268433	0.305884	0.558575
C	-4.625920	0.763910	0.702375
H	-4.895412	1.461126	-0.097444
H	-5.324187	-0.077947	0.690577
H	-4.689985	1.273413	1.662756
C	-3.545050	-1.049538	-1.533453
H	-3.813276	-0.296319	-2.283450
H	-2.934713	-1.812947	-2.020917
H	-4.472594	-1.511017	-1.179103
C	0.013782	0.289155	1.923655

H	0.587389	-0.641662	1.954060
H	0.679952	1.070886	1.544759
H	-0.293472	0.552824	2.937886
H	3.546317	3.004186	-0.328443
H	-0.040294	1.173131	-1.826121
O	2.826041	-1.454000	0.376326
C	4.068718	-1.626090	1.016551
H	4.904457	-1.387362	0.346869
H	4.145839	-1.009063	1.921076
H	4.120812	-2.678885	1.295359

34

4pzMeOMe1A-trans-m ; E = -798.325364831 Hartree

C	2.779610	0.516603	0.000171
C	4.120126	0.124404	0.000148
C	1.766350	-0.468439	-0.000102
H	4.909762	0.866234	0.000372
C	4.451048	-1.227224	-0.000155
C	2.125348	-1.818876	-0.000419
C	3.457328	-2.203203	-0.000441
H	3.722582	-3.255708	-0.000697
N	0.443719	0.001839	-0.000129
N	-0.433580	-0.903733	0.000075
C	-1.740397	-0.460655	0.000042
C	-2.305972	0.822402	-0.000199
C	-2.843500	-1.357684	0.000262
N	-3.987686	-0.695217	0.000164
N	-3.637353	0.614640	-0.000109
C	-4.671448	1.616668	-0.000165
H	-5.624584	1.090164	-0.000590
H	-4.604341	2.249435	0.890298
H	-4.603789	2.249910	-0.890242
C	-1.706642	2.181925	-0.000489
H	-2.019085	2.751844	0.882874
H	-0.620624	2.091922	-0.000032
H	-2.018336	2.751133	-0.884585
C	-2.803532	-2.844622	0.000529
H	-2.274547	-3.222516	-0.879521
H	-2.274104	-3.222213	0.880433
H	-3.820338	-3.241660	0.000815
H	5.498415	-1.514602	-0.000176
H	1.320993	-2.546813	-0.000653
O	2.370938	1.805193	0.000434
C	3.360033	2.806849	0.000354
H	3.994669	2.751173	0.894094
H	3.994597	2.751114	-0.893431
H	2.825425	3.757446	0.000347

34

4pzMeOMe1A-trans-n ; E = -798.326208305 Hartree

C	-2.865808	0.346541	0.000134
C	-4.155442	-0.190649	0.000157
C	-1.750038	-0.521608	-0.000203
H	-5.021240	0.460739	0.000406
C	-4.336719	-1.570227	-0.000170
C	-1.960344	-1.902931	-0.000549
C	-3.242143	-2.431462	-0.000531
H	-3.390471	-3.506747	-0.000825
N	-0.488289	0.094162	-0.000292
N	0.486515	-0.707294	0.000020

C	1.721142	-0.097852	-0.000052
C	2.908418	-0.830234	0.000077
C	2.120091	1.274623	-0.000524
N	3.439711	1.361365	-0.000676
N	3.896273	0.084853	-0.000464
C	5.318617	-0.133433	0.000903
H	5.764808	0.310515	0.894470
H	5.519749	-1.204940	-0.008293
H	5.767655	0.326419	-0.882995
C	3.099631	-2.301783	0.000829
H	3.645051	-2.644091	0.887088
H	2.114462	-2.771465	0.000953
H	3.645134	-2.645029	-0.885037
C	1.270652	2.493651	-0.000944
H	0.609510	2.506185	-0.872059
H	0.609329	2.506618	0.870020
H	1.905014	3.382915	-0.001118
H	-5.346352	-1.970570	-0.000160
H	-1.082577	-2.540225	-0.000840
O	-2.600236	1.670407	0.000417
C	-3.691707	2.559129	0.001072
H	-4.316921	2.435787	-0.892640
H	-4.316600	2.434855	0.894877
H	-3.262226	3.561578	0.001512

34

4pzMeOMe1B-cis-m ; E = -798.307178815 Hartree

C	-2.715505	0.322438	-1.236962
C	-3.600422	0.812581	-0.282965
C	-1.598196	-0.424795	-0.870970
H	-4.449080	1.417241	-0.585548
C	-3.389995	0.508044	1.056592
C	-1.426263	-0.785998	0.483150
C	-2.316856	-0.295608	1.438217
H	-2.184577	-0.548630	2.483930
N	-0.783886	-0.922766	-1.928585
N	0.464867	-0.845044	-1.909571
C	1.169424	-0.133532	-0.935001
C	0.993948	1.145565	-0.410494
C	2.363443	-0.624609	-0.348304
N	2.863783	0.257629	0.502645
N	2.033209	1.321104	0.440967
C	2.378132	2.518260	1.163641
H	2.756974	3.297425	0.493027
H	1.512105	2.905100	1.707110
H	3.160392	2.247932	1.871660
C	0.004795	2.214989	-0.702023
H	0.470687	3.204842	-0.680048
H	-0.423000	2.064847	-1.694415
H	-0.826814	2.205609	0.012270
C	2.971188	-1.965279	-0.553226
H	2.340917	-2.750907	-0.122668
H	3.064005	-2.182906	-1.621330
H	3.956648	-2.007699	-0.085677
H	-4.072355	0.876263	1.816693
O	-0.399102	-1.616980	0.761479
C	-0.069280	-1.838742	2.113270
H	0.133769	-0.894799	2.633420
H	-0.862841	-2.383104	2.640169

H 0.838202 -2.442702 2.105508
H -2.854504 0.530972 -2.293675
34
4pzMeOMe1B-cis-n ; E = -798.307416181 Hartree
C 2.881749 -1.113645 -0.636718
C 3.823360 -0.709401 0.302175
C 1.725134 -0.371292 -0.865541
H 4.701550 -1.318001 0.491190
C 3.630694 0.485493 0.985733
C 1.563522 0.869174 -0.212166
C 2.514920 1.278104 0.722436
H 2.392893 2.218289 1.247761
N 0.854763 -0.834235 -1.887634
N -0.385030 -0.912343 -1.740596
C -1.056719 -0.719148 -0.533144
C -2.342906 -0.185029 -0.554302
C -0.817466 -1.065217 0.833929
N -1.871349 -0.740374 1.571273
N -2.764863 -0.185539 0.724535
C -4.023549 0.290599 1.236019
H -4.152392 1.357794 1.030337
H -4.861456 -0.259055 0.795991
H -4.011926 0.127077 2.312651
C -3.122559 0.335081 -1.702554
H -3.143334 1.431155 -1.721459
H -2.644805 -0.007277 -2.623013
H -4.158484 -0.018806 -1.684101
C 0.315684 -1.791800 1.473283
H 0.660402 -2.615246 0.842454
H 1.175501 -1.139260 1.651760
H -0.020448 -2.196690 2.430217
H 4.357124 0.821061 1.719555
O 0.480891 1.599722 -0.562191
C 0.194648 2.756475 0.186769
H 0.064623 2.520821 1.250375
H 0.976843 3.518075 0.075749
H -0.741695 3.148268 -0.212442
H 3.004751 -2.034912 -1.198059
34
4pzMeOMe1B-trans-m ; E = -798.319731027 Hartree
C 2.407785 -2.004643 0.018154
C 3.785313 -2.161708 0.018812
C 1.794672 -0.746725 0.006732
H 4.226327 -3.152869 0.028386
C 4.586290 -1.026181 0.006648
C 2.625140 0.407981 -0.006015
C 4.013069 0.242026 -0.005951
H 4.659891 1.111071 -0.015704
N 0.393480 -0.815231 0.007689
N -0.232122 0.280286 0.009113
C -1.609384 0.184731 0.007073
C -2.506210 -0.892137 -0.006585
C -2.425222 1.349344 0.017065
N -3.707634 1.028253 0.010392
N -3.730380 -0.327330 -0.003491
C -4.999602 -1.006968 -0.018970
H -5.771464 -0.239282 0.005908
H -5.113036 -1.608188 -0.926650

H	-5.105761	-1.659428	0.853270
C	-2.306446	-2.363876	-0.023605
H	-2.775710	-2.819945	-0.903430
H	-1.237964	-2.572432	-0.042227
H	-2.744425	-2.835592	0.864253
C	-1.969249	2.764905	0.034875
H	-1.380912	2.972923	0.933694
H	-1.323862	2.975332	-0.822977
H	-2.831312	3.434114	0.009495
H	5.668575	-1.116566	0.006588
O	2.033507	1.617212	-0.018681
C	2.854407	2.761191	-0.034535
H	3.490969	2.817793	0.857661
H	3.487638	2.795554	-0.930242
H	2.173448	3.612946	-0.043736
H	1.742750	-2.862622	0.027056

34

4pzMeOMe1B-trans-n ; E = -798.321884117 Hartree

C	2.653475	-1.818791	0.003945
C	4.039814	-1.799378	0.005304
C	1.885220	-0.649021	0.001397
H	4.603373	-2.726412	0.007275
C	4.689525	-0.571172	0.004075
C	2.561616	0.601616	0.000142
C	3.959179	0.613874	0.001529
H	4.491021	1.557698	0.000618
N	0.505390	-0.902737	0.000353
N	-0.261926	0.099119	-0.001873
C	-1.605492	-0.206656	-0.001999
C	-2.568215	0.801769	-0.005230
C	-2.341254	-1.431725	-0.003110
N	-3.640120	-1.180171	-0.006553
N	-3.756966	0.171448	-0.011770
C	-5.067566	0.764952	0.021356
H	-5.277722	1.213050	0.998421
H	-5.161793	1.534338	-0.750070
H	-5.785706	-0.031254	-0.170101
C	-2.385072	2.272995	-0.002285
H	-2.834265	2.738143	0.882431
H	-1.312753	2.478754	-0.000833
H	-2.831416	2.740988	-0.887139
C	-1.838377	-2.830539	-0.005044
H	-1.208474	-3.016320	-0.879662
H	-1.210417	-3.019228	0.870307
H	-2.682818	-3.523417	-0.007196
H	5.774495	-0.522543	0.005082
O	1.821775	1.729155	-0.002356
C	2.496686	2.964879	-0.003921
H	3.120448	3.089706	0.890386
H	3.122207	3.086472	-0.897442
H	1.718107	3.728733	-0.006069
H	2.099637	-2.752384	0.004801

38

4pzMeOMe2-cis-m ; E = -912.710258974 Hartree

C	2.437917	-0.111961	-0.239164
C	3.134341	0.312706	0.893008
C	1.203535	0.461165	-0.581779
H	4.076662	-0.143076	1.170705

C	2.597979	1.333232	1.669903
C	0.695202	1.510275	0.204786
C	1.389553	1.934750	1.340181
H	1.000614	2.736771	1.955281
N	0.636442	0.113888	-1.842272
N	-0.555901	-0.243830	-1.952850
C	-1.361257	-0.522741	-0.845348
C	-1.121037	-1.293025	0.290143
C	-2.721126	-0.131252	-0.759639
N	-3.268419	-0.585134	0.357371
N	-2.293834	-1.292894	0.968622
C	-2.608311	-2.027739	2.165976
H	-2.698357	-3.101494	1.966962
H	-1.840834	-1.869688	2.928323
H	-3.564107	-1.652302	2.529163
C	0.060814	-2.080687	0.723291
H	-0.237060	-3.049612	1.136820
H	0.722856	-2.259837	-0.126584
H	0.646045	-1.546590	1.481546
C	-3.465280	0.728241	-1.717069
H	-3.067625	1.748591	-1.718791
H	-3.359311	0.345279	-2.736520
H	-4.523087	0.761007	-1.449580
H	3.135021	1.669065	2.551649
O	-0.459772	2.064369	-0.224945
C	-1.072373	3.035816	0.590221
H	-1.295365	2.638418	1.587803
H	-0.450359	3.934399	0.686816
H	-2.007113	3.297599	0.093834
O	2.833132	-1.127301	-1.043988
C	4.093701	-1.705374	-0.796964
H	4.896355	-0.959611	-0.848698
H	4.126560	-2.201166	0.181870
H	4.242256	-2.448945	-1.580505

38

4pzMeOMe2-cis-n ; E = -912.709808065 Hartree

C	2.582856	-0.277796	-0.189697
C	3.346970	0.480296	0.697520
C	1.332573	0.181349	-0.634608
H	4.299983	0.117529	1.061980
C	2.861749	1.711666	1.123636
C	0.877347	1.442280	-0.212169
C	1.639148	2.200647	0.680293
H	1.290429	3.168023	1.019928
N	0.689101	-0.548557	-1.669062
N	-0.511036	-0.889546	-1.596828
C	-1.285731	-0.781155	-0.440757
C	-2.652181	-0.538036	-0.547353
C	-1.053162	-1.014924	0.949182
N	-2.190698	-0.901150	1.622672
N	-3.134498	-0.587307	0.709673
C	-4.488325	-0.359427	1.141965
H	-4.796208	0.673664	0.950530
H	-5.181537	-1.035812	0.632908
H	-4.517016	-0.552879	2.213338
C	-3.459163	-0.238505	-1.754114
H	-3.718655	0.824728	-1.821086
H	-2.865936	-0.495737	-2.634239

```
H        -4.391958       -0.811624       -1.773069
C         0.181492       -1.444052        1.663524
H         0.783233       -2.111939        1.041035
H         0.812479       -0.588989        1.926967
H        -0.098421       -1.965175        2.581788
H         3.450319        2.304224        1.817229
O        -0.298806        1.852887       -0.739901
C        -0.835325        3.071530       -0.285998
H        -1.015765        3.053020        0.796096
H        -0.182567        3.919430       -0.529220
H        -1.785883        3.193205       -0.807000
O         2.923325       -1.510010       -0.632316
C         4.186516       -2.011471       -0.263266
H         4.996787       -1.351664       -0.597582
H         4.263448       -2.153296        0.822405
H         4.282445       -2.978219       -0.758407
38
4pzMeOMe2-trans-m ; E = -912.720681427 Hartree
C        -2.410458       -1.223497       -0.099919
C        -3.801750       -1.138858       -0.129733
C        -1.607427       -0.061987       -0.003211
H        -4.412961       -2.027521       -0.220938
C        -4.402844        0.110314       -0.042085
C        -2.252732        1.195806        0.090094
C        -3.649028        1.269558        0.074349
H        -4.148470        2.227242        0.148225
N        -0.236330       -0.310276        0.090047
N         0.538334        0.634314       -0.225057
C         1.882536        0.333207       -0.108516
C         2.566553       -0.859654        0.158894
C         2.893948        1.311765       -0.301107
N         4.096954        0.779870       -0.160797
N         3.873332       -0.528601        0.115016
C         5.000090       -1.408830        0.283959
H         5.891001       -0.783337        0.316331
H         4.914464       -1.973043        1.216928
H         5.083562       -2.113244       -0.550553
C         2.091896       -2.242742        0.420830
H         2.195523       -2.500325        1.482431
H         1.033259       -2.310016        0.168252
H         2.663953       -2.977912       -0.155963
C         2.703696        2.756468       -0.599598
H         2.030139        2.889237       -1.450682
H         2.249004        3.275048        0.250675
H         3.666099        3.221807       -0.821088
H        -5.486131        0.182015       -0.064607
O        -1.465924        2.280100        0.229873
C        -2.082801        3.523952        0.461284
H        -2.694741        3.838955       -0.393440
H        -2.707298        3.508274        1.363659
H        -1.268864        4.236894        0.597868
O        -1.734542       -2.392810       -0.181280
C        -2.485020       -3.581788       -0.243353
H        -3.127651       -3.703758        0.637854
H        -3.105345       -3.623891       -1.147746
H        -1.757802       -4.394181       -0.271736
38
4pzMeOMe2-trans-n ; E = -912.722044178 Hartree
```

```
C        2.606426       -0.984225       -0.152405
C        3.967134       -0.684085       -0.210333
C        1.636707        0.037749       -0.014087
H        4.705380       -1.466106       -0.333373
C        4.370294        0.641002       -0.109044
C        2.082382        1.377685        0.092745
C        3.449498        1.667396        0.048669
H        3.797331        2.689204        0.132255
N        0.322349       -0.420905        0.106502
N       -0.591480        0.394013       -0.198658
C       -1.865330       -0.110217       -0.035874
C       -2.992164        0.664902       -0.300078
C       -2.366822       -1.387458        0.361333
N       -3.689970       -1.381894        0.337397
N       -4.048329       -0.136869       -0.065735
C       -5.444331        0.201903       -0.144181
H       -5.749682        0.838155        0.693784
H       -5.661057        0.721331       -1.081595
H       -6.005310       -0.731146       -0.107713
C       -3.077709        2.077368       -0.743276
H       -3.611704        2.701328       -0.017625
H       -2.060621        2.459447       -0.851619
H       -3.592379        2.172824       -1.706222
C       -1.611237       -2.607637        0.748172
H       -0.889629       -2.879215       -0.026978
H       -1.021335       -2.428077        1.651982
H       -2.307241       -3.431524        0.922461
H        5.428622        0.880121       -0.153455
O        1.141681        2.327512        0.275972
C        1.571265        3.639072        0.552091
H        2.214664        3.679512        1.440238
H        2.106705        4.079561       -0.298670
H        0.664302        4.215904        0.737898
O        2.116285       -2.240035       -0.242793
C        3.037436       -3.299943       -0.334634
H        3.637174       -3.239271       -1.251939
H        3.710396       -3.330341        0.531793
H        2.441383       -4.213005       -0.357788
42
4pzMePyr1A-cis-m ; E = -895.077124832 Hartree
C       -3.023194       -0.385740        1.187924
C       -3.919369       -0.493480        0.135698
C       -1.737604        0.132412        1.006605
H       -4.898228       -0.935581        0.289059
C       -3.531895       -0.032954       -1.121839
C       -1.389359        0.728769       -0.235037
C       -2.297321        0.575171       -1.301570
H       -2.038811        0.969438       -2.277823
N       -0.911583        0.063921        2.162313
N        0.282526       -0.329870        2.128181
C        0.901743       -0.865002        0.995377
C        0.477225       -1.785454        0.032260
C        2.276445       -0.638715        0.714218
N        2.653719       -1.309567       -0.363874
N        1.559526       -1.999952       -0.749686
C        1.675433       -2.928570       -1.845365
H        1.676129       -3.965579       -1.492600
H        0.855795       -2.793482       -2.555750
```

H	2.623648	-2.719603	-2.338531
C	-0.788477	-2.544318	-0.139160
H	-0.596152	-3.548516	-0.528489
H	-1.295491	-2.644996	0.820980
H	-1.480801	-2.033198	-0.817264
C	3.221954	0.232714	1.464003
H	3.214250	1.264085	1.093037
H	2.941210	0.260430	2.520190
H	4.240658	-0.148410	1.365260
H	-4.208677	-0.115564	-1.967439
C	1.196255	3.292262	-0.130537
C	0.349041	2.281182	0.642019
N	-0.219626	1.429950	-0.400580
C	0.255188	1.812440	-1.717779
C	1.555429	2.539123	-1.410156
H	0.602017	4.179380	-0.376993
H	-0.447823	2.746742	1.234493
H	0.391823	0.926125	-2.347585
H	2.346286	1.805685	-1.219294
H	-0.449654	2.489210	-2.230940
H	1.876014	3.192649	-2.225208
H	2.067813	3.622063	0.441009
H	0.966597	1.706406	1.340526
H	-3.267493	-0.757706	2.178908

42

4pzMePyr1A-cis-n ; E = -895.077080682 Hartree

C	-3.143966	-0.778193	0.977660
C	-4.026806	-0.703564	-0.088083
C	-1.876122	-0.192423	0.919176
H	-4.989674	-1.201330	-0.041278
C	-3.646313	0.010667	-1.223421
C	-1.536343	0.643902	-0.176574
C	-2.431840	0.680801	-1.262348
H	-2.177390	1.262664	-2.140801
N	-1.056954	-0.446161	2.050472
N	0.148846	-0.788325	1.953538
C	0.790441	-1.093393	0.749791
C	2.143724	-0.796684	0.595934
C	0.435497	-1.831096	-0.423413
N	1.484803	-1.932252	-1.228052
N	2.494126	-1.280538	-0.612576
C	3.769894	-1.171826	-1.269710
H	3.994071	-0.132481	-1.532639
H	4.572206	-1.557571	-0.633938
H	3.711385	-1.769139	-2.178527
C	3.068795	-0.093813	1.518122
H	3.270326	0.937567	1.203504
H	2.608953	-0.062363	2.508658
H	4.031080	-0.610815	1.593462
C	-0.817603	-2.559548	-0.766057
H	-1.226888	-3.064647	0.112713
H	-1.593148	-1.889223	-1.146004
H	-0.594184	-3.306399	-1.530902
H	-4.312637	0.074086	-2.078779
C	0.940297	3.236516	0.444507
C	0.137658	2.062164	1.003618
N	-0.382268	1.394757	-0.186592
C	0.099824	2.020431	-1.403881

C	1.356077	2.742215	-0.939553
H	0.302054	4.121412	0.342890
H	-0.682668	2.373503	1.661182
H	0.288895	1.262868	-2.172721
H	2.183323	2.027591	-0.858723
H	-0.622970	2.747917	-1.811857
H	1.658163	3.543702	-1.618231
H	1.783993	3.504791	1.085926
H	0.778500	1.395554	1.592289
H	-3.382557	-1.346979	1.871638

42

4pzMePyr1A-trans-m ; E = -895.088860267 Hartree

C	1.693101	-2.763648	-0.034912
C	2.962535	-3.198004	0.299470
C	1.342997	-1.405847	-0.045482
H	3.195698	-4.256861	0.333026
C	3.921986	-2.239648	0.624320
C	2.359712	-0.419156	0.143734
C	3.630916	-0.888101	0.542632
H	4.414912	-0.173763	0.762555
N	-0.040105	-1.213335	-0.178984
N	-0.514923	-0.185905	0.381077
C	-1.889029	-0.032966	0.289503
C	-2.902342	-0.786908	-0.314384
C	-2.552793	1.069394	0.892659
N	-3.858156	1.005934	0.685855
N	-4.044125	-0.119641	-0.042522
C	-5.378200	-0.484585	-0.442419
H	-5.477814	-0.483152	-1.532647
H	-5.642762	-1.476916	-0.064440
H	-6.051786	0.257695	-0.016865
C	-2.882746	-2.046945	-1.101129
H	-3.375876	-1.920249	-2.072018
H	-1.847826	-2.346848	-1.258849
H	-3.402094	-2.855087	-0.571670
C	-1.935045	2.185983	1.657886
H	-1.246629	1.803738	2.416112
H	-1.354331	2.842204	0.999557
H	-2.712504	2.781957	2.139720
H	4.921973	-2.544715	0.919724
C	1.838040	2.744692	-1.531826
C	1.203212	1.448582	-1.037702
N	2.170953	0.918948	-0.084979
C	3.233286	1.878812	0.171907
C	2.650894	3.195332	-0.322153
H	2.506957	2.545975	-2.377253
H	1.015845	0.724724	-1.839615
H	3.497397	1.894444	1.235580
H	1.990568	3.623829	0.440005
H	4.145482	1.628771	-0.395705
H	3.423867	3.930633	-0.558655
H	1.091439	3.474396	-1.855359
H	0.242117	1.632196	-0.541895
H	0.890710	-3.466896	-0.237062

42

4pzMePyr1A-trans-n ; E = -895.090304196 Hartree

C	-2.035689	-2.621733	-0.038764
C	-3.329323	-2.878979	-0.456222

```
C        -1.510843       -1.323495        0.014034
H        -3.699011       -3.896763       -0.520826
C        -4.131063       -1.798613       -0.820532
C        -2.372258       -0.208547       -0.219803
C        -3.668172       -0.498150       -0.698331
H        -4.335617        0.316549       -0.951754
N        -0.124924       -1.316838        0.240616
N         0.515525       -0.370470       -0.296933
C         1.884049       -0.414909       -0.105124
C         2.723175        0.541961       -0.671986
C         2.746402       -1.295214        0.615849
N         4.003964       -0.899460        0.493023
N         3.969789        0.210161       -0.283370
C         5.203416        0.860088       -0.639062
H         5.452393        0.694318       -1.692750
H         5.140198        1.936174       -0.454214
H         5.984799        0.429686       -0.014125
C         2.385122        1.703949       -1.529688
H         2.888524        1.654223       -2.501654
H         1.306279        1.702492       -1.698716
H         2.664783        2.654167       -1.059816
C         2.403170       -2.496368        1.421873
H         1.690231       -2.245640        2.212485
H         1.917153       -3.255572        0.802598
H         3.311337       -2.911923        1.864270
H        -5.143024       -1.964488       -1.179345
C        -1.548395        2.821143        1.569979
C        -1.054767        1.465296        1.073913
N        -2.015200        1.091451        0.040772
C        -2.934410        2.183727       -0.237228
C        -2.222051        3.402567        0.330926
H        -2.288343        2.690068        2.367992
H        -1.020096        0.704128        1.862202
H        -3.136330        2.261715       -1.311809
H        -1.468098        3.762736       -0.378065
H        -3.900760        2.035934        0.274141
H        -2.909161        4.224432        0.546742
H        -0.737059        3.440619        1.960679
H        -0.045009        1.533014        0.651436
H        -1.347446       -3.426130        0.202757
42
4pzMePyr1B-cis-m ; E = -895.075168267 Hartree
C         1.805844        0.762185       -0.350033
C         2.143958        2.101401       -0.050625
C         0.532047        0.540136       -0.941075
H         3.090343        2.315701        0.430144
C         1.299730        3.156373       -0.357170
C        -0.295729        1.622880       -1.253513
C         0.071594        2.929069       -0.974663
H        -0.580527        3.752910       -1.244642
N         0.183710       -0.755040       -1.405012
N        -0.958888       -1.246283       -1.247633
C        -1.937042       -0.711119       -0.399656
C        -1.877315       -0.157188        0.883466
C        -3.321209       -0.874095       -0.671681
N        -4.055871       -0.422823        0.332843
N        -3.167659       -0.007676        1.261304
C        -3.661102        0.470725        2.527846
```

H	-3.432208	-0.231073	3.336948
H	-3.233969	1.447784	2.769246
H	-4.741776	0.561673	2.429528
C	-0.739569	0.173953	1.779167
H	-1.030310	0.109769	2.831431
H	0.090128	-0.517671	1.617620
H	-0.355668	1.181757	1.581096
C	-3.928505	-1.426261	-1.911813
H	-3.906182	-0.696908	-2.728981
H	-3.365859	-2.302424	-2.246139
H	-4.968560	-1.703541	-1.729764
H	1.613442	4.168247	-0.117174
C	3.561686	-2.286820	0.687188
C	2.486431	-1.677832	-0.203171
N	2.673036	-0.242409	-0.008334
C	3.996298	0.057245	0.521271
C	4.709129	-1.292503	0.545392
H	3.213595	-2.318923	1.726117
H	2.625424	-1.964790	-1.252922
H	4.521873	0.786303	-0.108982
H	5.449698	-1.356441	1.346405
H	1.477476	-1.985703	0.077016
H	3.825835	-3.303303	0.385289
H	5.227585	-1.456502	-0.405604
H	3.921983	0.490834	1.530723
H	-1.240095	1.413023	-1.745119

42

4pzMePyr1B-cis-n ; E = -895.076317642 Hartree

C	1.964644	0.765167	-0.273478
C	2.409394	2.045058	0.124657
C	0.642571	0.675486	-0.788136
H	3.401056	2.156454	0.545546
C	1.611503	3.170197	-0.009407
C	-0.138743	1.827312	-0.923437
C	0.328495	3.075055	-0.544113
H	-0.289744	3.956816	-0.674666
N	0.202064	-0.534368	-1.377213
N	-0.959905	-0.979314	-1.227225
C	-1.875744	-0.514828	-0.273166
C	-3.240293	-0.675050	-0.509375
C	-1.784809	-0.071756	1.085828
N	-2.998966	0.042949	1.605794
N	-3.861403	-0.297493	0.625181
C	-5.277514	-0.252918	0.880132
H	-5.778336	0.437260	0.194224
H	-5.728072	-1.245165	0.777557
H	-5.405557	0.096562	1.903588
C	-3.931991	-1.143825	-1.734246
H	-4.411312	-0.321985	-2.279130
H	-3.188748	-1.599686	-2.392158
H	-4.704315	-1.885222	-1.504175
C	-0.600104	0.176631	1.953586
H	0.149807	-0.612159	1.842504
H	-0.110267	1.123701	1.706873
H	-0.925821	0.206141	2.995588
H	2.003674	4.133573	0.303650
C	3.514039	-2.451244	0.517237
C	2.426915	-1.725166	-0.267042

N	2.787962	-0.317280	-0.116090
C	4.151707	-0.161186	0.365494
C	4.735611	-1.567591	0.286873
H	3.259210	-2.470603	1.583185
H	2.422643	-2.027592	-1.320449
H	4.709235	0.556972	-0.248926
H	5.537250	-1.725557	1.012571
H	1.424825	-1.915840	0.124102
H	3.650999	-3.482332	0.181910
H	5.144378	-1.747021	-0.713668
H	4.162641	0.214156	1.401647
H	-1.125897	1.722022	-1.361329

42

4pzMePyr1B-trans-m ; E = -895.094123380 Hartree

C	-2.448178	-0.489670	-0.150136
C	-3.631466	-1.237765	-0.348815
C	-1.219858	-1.211088	-0.153862
H	-4.586919	-0.728841	-0.373973
C	-3.606538	-2.612989	-0.495283
C	-1.227762	-2.606880	-0.288807
C	-2.400851	-3.316519	-0.453608
H	-2.382306	-4.394610	-0.575846
N	-0.021073	-0.484053	-0.115939
N	1.004705	-1.167598	0.162928
C	2.207576	-0.495944	0.085082
C	2.570297	0.714457	-0.517247
C	3.413166	-1.021757	0.623968
N	4.427559	-0.205281	0.390820
N	3.897520	0.832064	-0.299300
C	4.772545	1.875358	-0.768209
H	4.906936	1.826447	-1.854208
H	4.379222	2.860209	-0.501775
H	5.735337	1.724465	-0.282099
C	1.786409	1.699681	-1.302212
H	2.369944	2.112824	-2.131123
H	0.889920	1.214852	-1.691387
H	1.451858	2.537579	-0.677558
C	3.586320	-2.290693	1.380241
H	3.123885	-2.227551	2.370671
H	3.105688	-3.120659	0.854899
H	4.648809	-2.507489	1.505931
H	-4.543213	-3.142947	-0.644495
C	-2.315688	2.868381	1.245994
C	-1.515057	1.707741	0.663798
N	-2.531616	0.865957	0.035025
C	-3.813978	1.551097	-0.036366
C	-3.468909	3.004939	0.257614
H	-2.701852	2.601970	2.236576
H	-0.785404	2.061855	-0.072564
H	-4.279206	1.414356	-1.019930
H	-4.322681	3.563485	0.649128
H	-0.957259	1.144692	1.417017
H	-1.716871	3.777053	1.349691
H	-3.126552	3.502768	-0.656606
H	-4.515931	1.161377	0.719249
H	-0.261240	-3.099558	-0.289245

42

4pzMePyr1B-trans-n ; E = -895.095381617 Hartree

C	-2.467063	0.634752	0.085312
C	-3.556728	1.533275	0.172619
C	-1.159145	1.199394	0.134088
H	-4.567773	1.146389	0.151487
C	-3.370958	2.899954	0.268276
C	-1.005048	2.591019	0.220101
C	-2.086476	3.447143	0.283838
H	-1.939206	4.518708	0.371628
N	-0.048310	0.340790	0.192268
N	1.050936	0.896193	-0.088882
C	2.181290	0.122334	0.068377
C	3.425583	0.578289	-0.368634
C	2.440183	-1.145429	0.673881
N	3.731721	-1.429377	0.603935
N	4.306731	-0.383139	-0.034215
C	5.724603	-0.398524	-0.280828
H	5.938694	-0.412115	-1.354375
H	6.209226	0.474655	0.166610
H	6.117864	-1.304885	0.177343
C	3.781134	1.838227	-1.064567
H	4.174185	1.652406	-2.070723
H	2.879880	2.447246	-1.154033
H	4.537157	2.408460	-0.513086
C	1.502275	-2.082590	1.345456
H	0.816919	-1.542027	2.003209
H	0.885025	-2.614157	0.613784
H	2.068709	-2.818195	1.920870
H	-4.241905	3.546187	0.334153
C	-2.663297	-2.817283	-1.068106
C	-1.771106	-1.718494	-0.500923
N	-2.718516	-0.703858	-0.049615
C	-4.079059	-1.220315	-0.025408
C	-3.903430	-2.725278	-0.185463
H	-2.925506	-2.591804	-2.108224
H	-1.167015	-2.089761	0.333808
H	-4.586794	-0.951223	0.908781
H	-4.787732	-3.202998	-0.614465
H	-1.075996	-1.301316	-1.234412
H	-2.181166	-3.798047	-1.045176
H	-3.707186	-3.185986	0.789138
H	-4.674574	-0.807671	-0.856271
H	0.012712	2.963040	0.267394

54

4pzMePyr2-cis-m ; E = -1106.24555520 Hartree

C	2.145084	0.570763	0.535724
C	2.312241	0.927915	1.891271
C	0.828141	0.642084	-0.011082
H	3.276466	0.814842	2.367362
C	1.242004	1.382498	2.643279
C	-0.222836	1.248798	0.732837
C	-0.014543	1.559038	2.087225
H	-0.814995	1.979231	2.681990
N	0.650669	0.159407	-1.329546
N	-0.296955	-0.605306	-1.660779
C	-1.189326	-1.182243	-0.754828
C	-1.003516	-1.783546	0.493590
C	-2.547427	-1.431629	-1.089479
N	-3.164428	-2.077902	-0.110967

N	-2.214370	-2.287838	0.825053
C	-2.548051	-3.064690	1.991250
H	-2.090867	-4.059513	1.954464
H	-2.222490	-2.555685	2.902316
H	-3.631864	-3.171034	2.000707
C	0.209702	-2.012536	1.319699
H	0.134738	-2.949738	1.879388
H	1.093219	-2.070934	0.680856
H	0.378975	-1.193703	2.027332
C	-3.256115	-1.049153	-2.341341
H	-3.648876	-0.026974	-2.299542
H	-2.565300	-1.098573	-3.187547
H	-4.097219	-1.723769	-2.514986
H	1.401917	1.634932	3.687965
C	-2.842822	2.837161	-1.225488
C	-1.572210	1.990062	-1.239305
N	-1.433266	1.538759	0.142531
C	-2.573686	1.942871	0.946698
C	-3.652487	2.216759	-0.089138
H	-2.605809	3.879935	-0.985116
H	-0.679425	2.543711	-1.552874
H	-2.842822	1.152780	1.656626
H	-4.106352	1.272122	-0.407114
C	4.682171	-0.978991	-1.646630
C	3.313166	-0.319854	-1.550470
N	3.232980	0.077941	-0.145602
C	4.560868	0.144289	0.456401
C	5.523244	-0.140252	-0.693217
H	4.622884	-2.014086	-1.290705
H	3.238773	0.548616	-2.218945
H	4.744750	1.129820	0.902509
H	6.437491	-0.635383	-0.356153
H	2.497390	-0.989886	-1.818602
H	5.064789	-0.993698	-2.670317
H	5.809932	0.799157	-1.178783
H	4.660892	-0.601538	1.259832
H	-2.362111	2.859672	1.522747
H	-4.443133	2.868400	0.291178
H	-3.358751	2.823296	-2.189305
H	-1.676226	1.146511	-1.932077

54

4pzMePyr2-cis-n ; E = -1106.24541809 Hartree

C	2.293676	0.670493	0.301270
C	2.543017	1.523255	1.396950
C	0.951486	0.579833	-0.177960
H	3.529759	1.565293	1.837600
C	1.525322	2.279488	1.953970
C	-0.045382	1.465576	0.317131
C	0.243817	2.273633	1.428091
H	-0.515166	2.921418	1.846687
N	0.706796	-0.343833	-1.217809
N	-0.281772	-1.127389	-1.224193
C	-1.159174	-1.298483	-0.151704
C	-2.491916	-1.625192	-0.394791
C	-1.005369	-1.399141	1.267427
N	-2.162881	-1.726062	1.826454
N	-3.051204	-1.831190	0.815504
C	-4.426937	-2.129660	1.112105

H	-5.079472	-1.285288	0.865049
H	-4.765122	-3.011650	0.559951
H	-4.485648	-2.329620	2.181151
C	-3.213809	-1.750300	-1.684666
H	-3.858456	-0.887601	-1.892523
H	-2.473738	-1.820089	-2.485381
H	-3.842801	-2.646435	-1.708287
C	0.217101	-1.318543	2.114289
H	1.062740	-1.814930	1.629515
H	0.515003	-0.283832	2.304645
H	0.020110	-1.810436	3.069511
H	1.746909	2.912603	2.808673
C	-2.745566	2.316618	-1.966180
C	-1.478015	1.496749	-1.733623
N	-1.283942	1.549428	-0.287985
C	-2.384815	2.231641	0.368385
C	-3.507059	2.142343	-0.654249
H	-2.497209	3.374401	-2.109668
H	-0.599505	1.894115	-2.255224
H	-2.619061	1.748207	1.323439
H	-3.972697	1.151122	-0.609873
C	4.648570	-1.815681	-1.054982
C	3.295057	-1.131309	-1.205982
N	3.333099	-0.080454	-0.189427
C	4.692641	0.154036	0.278846
C	5.565009	-0.661927	-0.667908
H	4.607797	-2.552668	-0.244492
H	3.168392	-0.712724	-2.211783
H	4.939903	1.222518	0.251503
H	6.501859	-0.977430	-0.201642
H	2.458162	-1.810258	-1.039904
H	4.955596	-2.332516	-1.967854
H	5.812024	-0.066488	-1.554009
H	4.814885	-0.184051	1.320413
H	-2.152234	3.290903	0.571736
H	-4.283670	2.894548	-0.494232
H	-3.301752	1.980090	-2.845326
H	-1.613196	0.466740	-2.085998

54

4pzMePyr2-trans-m ; E = -1106.25444011 Hartree

C	2.513760	-0.279460	0.176685
C	3.715586	0.381490	0.501299
C	1.312598	0.501078	0.102326
H	4.628718	-0.181722	0.633212
C	3.730638	1.746464	0.724200
C	1.382774	1.931781	0.186716
C	2.599370	2.526909	0.564868
H	2.670763	3.598905	0.688391
N	0.132073	-0.230809	-0.002706
N	-0.877947	0.236396	0.602891
C	-2.036464	-0.512186	0.458684
C	-2.432664	-1.464293	-0.484894
C	-3.151299	-0.359749	1.326068
N	-4.143180	-1.158836	0.964588
N	-3.690480	-1.813517	-0.128206
C	-4.573905	-2.716040	-0.818516
H	-4.923702	-2.290200	-1.765623
H	-4.074097	-3.667472	-1.020697

H	-5.429625	-2.886268	-0.166537
C	-1.764620	-1.992599	-1.700739
H	-2.452330	-2.036583	-2.552252
H	-0.918732	-1.350069	-1.948099
H	-1.366456	-3.002476	-1.542358
C	-3.250443	0.531047	2.513446
H	-2.656378	0.145858	3.348897
H	-2.861871	1.526061	2.279114
H	-4.290282	0.611008	2.836511
H	4.665897	2.223023	1.005495
C	-1.125249	3.703388	-1.733698
C	-0.732406	2.382486	-1.080898
N	0.328953	2.745390	-0.153203
C	0.441309	4.190506	-0.029914
C	-0.865225	4.706728	-0.615219
H	-0.472107	3.918374	-2.587641
H	-0.378842	1.632583	-1.798649
H	0.591984	4.484078	1.015424
H	-1.662779	4.652564	0.134138
C	2.111343	-3.944291	0.126026
C	1.454065	-2.588860	-0.108405
N	2.560189	-1.643306	0.008388
C	3.844814	-2.330735	-0.030551
C	3.500790	-3.749142	-0.465797
H	2.184833	-4.143800	1.201279
H	0.995662	-2.542230	-1.104723
H	4.532337	-1.836551	-0.727393
H	4.236740	-4.479830	-0.120696
H	0.664128	-2.366349	0.610636
H	1.549043	-4.762739	-0.331729
H	3.455244	-3.804292	-1.559399
H	4.325637	-2.330612	0.961094
H	1.296706	4.574879	-0.610475
H	-0.787502	5.741435	-0.958255
H	-2.158917	3.698954	-2.089069
H	-1.579963	1.939106	-0.542981

54

4pzMePyr2-trans-n ; E = -1106.25528133 Hartree

C	2.565660	0.089490	0.316870
C	3.577814	0.938185	0.812192
C	1.250884	0.635427	0.149341
H	4.560772	0.542728	1.026786
C	3.311010	2.264717	1.094624
C	1.042262	2.047586	0.295482
C	2.076406	2.833184	0.832875
H	1.929799	3.890702	1.005385
N	0.231322	-0.286729	-0.102177
N	-0.879564	-0.030275	0.447241
C	-1.895299	-0.935076	0.183599
C	-3.074961	-0.912388	0.925954
C	-2.091150	-1.952930	-0.797385
N	-3.288638	-2.504011	-0.651774
N	-3.861995	-1.870102	0.395493
C	-5.167088	-2.281006	0.840458
H	-5.113204	-2.818978	1.793044
H	-5.824746	-1.415292	0.959481
H	-5.573115	-2.943035	0.076675
C	-3.447762	-0.045060	2.069740

H	-3.562923	-0.615429	2.998623
H	-2.650884	0.687777	2.212492
H	-4.388341	0.487665	1.888924
C	-1.199779	-2.384989	-1.907506
H	-0.697564	-1.525062	-2.357860
H	-0.414392	-3.059174	-1.552395
H	-1.786296	-2.910327	-2.664740
H	4.103090	2.885417	1.504861
C	-1.514492	3.424796	-1.861177
C	-0.969174	2.167343	-1.192875
N	-0.105685	2.672090	-0.134089
C	-0.268689	4.107970	0.029562
C	-1.569843	4.411099	-0.699480
H	-0.815437	3.786339	-2.624368
H	-0.405663	1.522520	-1.877282
H	-0.294876	4.382213	1.090792
H	-2.426474	4.187433	-0.053682
C	2.888440	-3.563309	-0.087821
C	2.013189	-2.329332	-0.272263
N	2.894196	-1.224428	0.087095
C	4.286064	-1.653907	0.138501
C	4.272420	-3.052639	-0.465966
H	2.876299	-3.876571	0.962484
H	1.666966	-2.238228	-1.310192
H	4.929218	-0.966897	-0.424483
H	5.091162	-3.674209	-0.094447
H	1.120263	-2.341153	0.354619
H	2.549661	-4.406544	-0.695673
H	4.363927	-2.988510	-1.556111
H	4.656851	-1.675087	1.175929
H	0.565443	4.659970	-0.435447
H	-1.639285	5.456798	-1.009509
H	-2.481797	3.254024	-2.340768
H	-1.781386	1.560137	-0.774019

24

4pzH-ts-B-m ; E = -605.300223945 Hartree

C	2.390899	-0.143536	1.220656
C	3.618130	0.498183	1.196611
C	1.750942	-0.476895	-0.000158
H	4.089995	0.740516	2.145318
C	4.250194	0.831889	0.000228
C	2.390527	-0.142103	-1.220777
H	1.909954	-0.400013	-2.157877
C	3.617788	0.499549	-1.196352
H	4.089381	0.742963	-2.144918
H	1.910608	-0.402538	2.157599
N	0.575894	-1.086562	-0.000342
N	-0.498176	-1.674443	-0.000559
C	-1.649061	-0.862675	-0.000260
C	-1.782827	0.522263	0.000099
C	-2.971933	-1.352168	-0.000275
N	-3.849337	-0.361358	0.000029
N	-3.102836	0.761409	0.000237
C	-3.770329	2.041267	0.000603
H	-4.397111	2.129634	0.890389
H	-3.020447	2.832522	0.000422
H	-4.397716	2.129793	-0.888739
H	-1.042115	1.308389	0.000142

H	-3.298792	-2.382696	-0.000525
H	5.211445	1.333457	0.000376

24
4pzH-ts-B-n ; E = -605.300668029 Hartree

C	-2.516767	0.155681	1.220780
C	-3.799091	-0.367249	1.195836
C	-1.849111	0.430174	0.000692
H	-4.290691	-0.568202	2.144162
C	-4.460287	-0.635912	-0.001059
C	-2.516889	0.159990	-1.220290
H	-2.012746	0.370094	-2.156874
C	-3.799195	-0.363061	-1.197065
H	-4.290876	-0.560696	-2.146046
H	-2.012535	0.362486	2.158051
N	-0.623589	0.931895	0.001518
N	0.499207	1.419897	0.002314
C	1.569863	0.505144	0.000858
C	2.899920	0.897383	0.001412
C	1.610302	-0.910189	-0.001304
N	2.858129	-1.345945	-0.002013
N	3.622651	-0.234249	-0.000331
C	5.058583	-0.373910	-0.000825
H	5.377404	-0.918781	-0.891939
H	5.511028	0.618058	0.001867
H	5.377407	-0.923614	0.887301
H	3.344921	1.881304	0.002891
H	0.781565	-1.604705	-0.002301
H	-5.464226	-1.045436	-0.001732

24
4pzH-ts-H-m ; E = -605.295863534 Hartree

C	-1.735526	0.776478	0.307693
C	-2.815404	1.646350	0.362547
C	-1.940202	-0.563044	-0.029499
H	-2.661089	2.687383	0.632954
C	-4.101847	1.186964	0.075818
C	-3.227512	-1.026727	-0.296718
H	-3.350180	-2.078172	-0.539509
C	-4.305991	-0.150908	-0.254078
H	-5.307107	-0.510448	-0.473145
H	-0.729072	1.110461	0.541461
N	-0.902438	-1.558031	-0.092092
N	0.260899	-1.128732	-0.009742
C	1.500285	-0.690266	0.044029
C	1.987462	0.615574	-0.194905
C	2.700215	-1.428581	0.327613
N	3.771579	-0.667225	0.276258
N	3.316533	0.561439	-0.075747
C	4.280007	1.604179	-0.329024
H	4.810876	1.413218	-1.265743
H	3.763549	2.562688	-0.390443
H	5.000394	1.626851	0.490062
H	1.466139	1.516674	-0.484362
H	2.775197	-2.472836	0.600526
H	-4.943635	1.871907	0.116599

24
4pzH-ts-H-n ; E = -605.296293711 Hartree

C	-1.904227	0.807698	0.214257
C	-3.077175	1.547823	0.258873

C	-1.962837	-0.571796	0.007569
H	-3.035435	2.620657	0.425631
C	-4.311771	0.917907	0.093466
C	-3.196545	-1.204219	-0.140218
H	-3.203134	-2.280610	-0.284988
C	-4.369592	-0.459404	-0.106404
H	-5.329760	-0.951253	-0.231740
H	-0.935110	1.277464	0.351877
N	-0.818990	-1.444049	-0.034718
N	0.290231	-0.882596	-0.042646
C	1.470908	-0.302289	-0.085302
C	2.726437	-0.900081	0.164768
C	1.825088	1.057181	-0.393994
N	3.125440	1.251663	-0.344350
N	3.647538	0.055123	0.026451
C	5.067695	-0.030289	0.263016
H	5.597014	0.403570	-0.586852
H	5.334998	0.520587	1.168723
H	5.349164	-1.077696	0.375368
H	2.968303	-1.911868	0.455966
H	1.163421	1.861129	-0.689638
H	-5.226874	1.501846	0.126308

52

4pzHAmid2-ts-B-m ; E = -1478.12028087 Hartree

C	2.121389	1.655123	0.009619
C	2.352425	3.013553	0.212479
C	0.781794	1.166699	-0.127203
H	3.366387	3.363133	0.348452
C	1.269998	3.888679	0.205075
C	-0.299713	2.109606	-0.128905
C	-0.045267	3.469082	0.020113
H	-0.869750	4.168466	0.002771
N	0.541107	-0.131374	-0.248895
N	0.344139	-1.358220	-0.419171
C	-0.887749	-1.837137	0.064147
C	-1.616309	-1.462921	1.196153
C	-1.623393	-2.892471	-0.531823
N	-2.734230	-3.126000	0.153263
N	-2.696741	-2.274376	1.202663
C	-3.798388	-2.255055	2.130008
H	-3.432286	-2.175989	3.156228
H	-4.473229	-1.416881	1.922938
H	-4.340842	-3.192148	2.010621
C	-1.332269	-0.488012	2.281867
H	-1.371976	-0.968516	3.265607
H	-0.338336	-0.059706	2.151253
H	-2.049863	0.339442	2.284358
C	-1.294617	-3.639046	-1.775590
H	-1.428936	-3.010233	-2.661989
H	-0.249966	-3.962013	-1.765211
H	-1.942397	-4.512672	-1.869267
H	1.461143	4.949255	0.339302
N	3.105351	0.686976	-0.044781
H	2.760260	-0.241518	-0.266016
N	-1.554233	1.559947	-0.326280
H	-1.559771	0.548928	-0.416676
C	4.456258	0.841672	0.128004
O	5.018435	1.890814	0.404718

C	-2.771418	2.191388	-0.358904
O	-2.951012	3.393756	-0.240548
C	6.558667	-0.640894	0.045468
C	6.732189	-2.028963	-0.217475
C	5.486396	-2.528536	-0.448564
O	4.557355	-1.553262	-0.347864
C	5.219370	-0.401103	-0.045801
H	7.309668	0.100256	0.273517
H	7.658110	-2.584877	-0.233923
H	5.112571	-3.512315	-0.687114
C	-5.246285	1.484859	-0.571797
C	-5.851466	0.210578	-0.770009
C	-4.830181	-0.688413	-0.856254
O	-3.641882	-0.059147	-0.723303
C	-3.899779	1.267188	-0.548595
H	-5.724880	2.445913	-0.458163
H	-6.906248	-0.010789	-0.842829
H	-4.770499	-1.758956	-0.987559

52

4pzHAmid2-ts-B-n ; E = -1478.11684741 Hartree

C	-2.104529	-1.713990	0.005353
C	-2.359900	-3.070608	0.174920
C	-0.757504	-1.241081	-0.131842
H	-3.379882	-3.402138	0.311346
C	-1.296734	-3.967700	0.119665
C	0.305261	-2.200997	-0.157457
C	0.019968	-3.561016	-0.068222
H	0.830190	-4.273980	-0.135805
N	-0.530477	0.057242	-0.281020
N	-0.286594	1.262931	-0.503547
C	0.886813	1.798395	0.046152
C	1.512238	2.909942	-0.514471
C	1.602617	1.520531	1.247012
N	2.600296	2.384321	1.387435
N	2.536370	3.201030	0.310467
C	3.547446	4.211519	0.135195
H	4.304598	3.896138	-0.591166
H	3.096189	5.145603	-0.207205
H	4.019601	4.371102	1.104055
C	1.188468	3.644960	-1.761357
H	1.940820	3.481054	-2.541020
H	0.227343	3.285270	-2.133671
H	1.115201	4.724029	-1.589013
C	1.307006	0.518872	2.309498
H	1.811663	0.812322	3.232054
H	0.231006	0.458472	2.496322
H	1.647396	-0.485161	2.040742
H	-1.504767	-5.028833	0.217730
N	-3.076734	-0.729736	-0.024306
H	-2.722570	0.194338	-0.247943
N	1.584692	-1.687671	-0.302396
H	1.653170	-0.679193	-0.221083
C	-4.426541	-0.866474	0.158191
O	-5.004344	-1.908186	0.432492
C	2.760733	-2.376477	-0.467270
O	2.855810	-3.575170	-0.682888
C	-6.509186	0.644791	0.106975
C	-6.668059	2.037399	-0.142220

C	-5.418631	2.523518	-0.380692
O	-4.500523	1.535709	-0.297340
C	-5.174167	0.389060	0.001001
H	-7.266857	-0.089192	0.335984
H	-7.587165	2.604794	-0.144762
H	-5.035110	3.504842	-0.613839
C	5.280209	-1.801982	-0.509085
C	5.975830	-0.603025	-0.180711
C	5.022890	0.306832	0.167715
O	3.793981	-0.241002	0.068558
C	3.954306	-1.529108	-0.341487
H	5.687880	-2.751403	-0.822002
H	7.043720	-0.440585	-0.190407
H	5.044113	1.324726	0.525489

52

4pzHAmid2-ts-H-m ; E = -1478.11838419 Hartree

C	0.111351	-1.954017	-0.168188
C	-0.132107	-3.312097	0.053369
C	-0.963961	-1.037001	-0.132526
H	0.698627	-4.003579	0.039375
C	-1.433574	-3.742904	0.277383
C	-2.284646	-1.517903	0.027968
C	-2.518062	-2.874568	0.254565
H	-3.531171	-3.217231	0.410604
N	-0.879995	0.368494	-0.352176
N	0.155484	0.956245	0.021250
C	1.263350	1.587897	0.328491
C	2.251356	1.192288	1.272432
C	1.802199	2.785568	-0.267436
N	2.989109	3.060543	0.230278
N	3.212421	2.117255	1.192442
C	4.450717	2.162466	1.928384
H	4.272151	1.923534	2.979029
H	5.173138	1.452438	1.512063
H	4.846372	3.173931	1.843319
C	2.240586	0.073523	2.241812
H	2.160305	0.432665	3.274545
H	1.378228	-0.565161	2.039409
H	3.148922	-0.533394	2.166046
C	1.187915	3.575726	-1.362963
H	1.290827	3.062888	-2.325657
H	0.116956	3.701960	-1.179915
H	1.665179	4.554111	-1.440880
H	-1.611058	-4.799366	0.454867
N	1.384709	-1.478455	-0.497393
H	1.434282	-0.517889	-0.801008
N	-3.288902	-0.557479	-0.027381
H	-2.946325	0.371546	-0.256331
C	2.589932	-2.109207	-0.355315
O	2.766919	-3.238922	0.076579
C	-4.633231	-0.733103	0.137371
O	-5.191621	-1.789280	0.400822
C	5.078228	-1.472461	-0.567146
C	5.722374	-0.282717	-1.016801
C	4.728544	0.556764	-1.422574
O	3.522302	-0.029482	-1.258944
C	3.740112	-1.266932	-0.730824
H	5.525375	-2.369899	-0.166873

H	6.782853	-0.078365	-1.042417
H	4.711008	1.561894	-1.814976
C	-6.759133	0.715190	0.065361
C	-6.959576	2.102398	-0.184579
C	-5.723541	2.627763	-0.408702
O	-4.775598	1.669402	-0.315703
C	-5.415685	0.501888	-0.025632
H	-7.495269	-0.043065	0.285227
H	-7.896038	2.640574	-0.197135
H	-5.369049	3.621082	-0.637179

52

4pzHAmid2-ts-H-n ; E = -1478.11386239 Hartree

C	0.108413	-2.035276	-0.112141
C	-0.173008	-3.389137	0.089609
C	-0.946051	-1.094237	-0.100881
H	0.642563	-4.098589	0.097454
C	-1.490322	-3.791574	0.268158
C	-2.281942	-1.545681	0.013316
C	-2.553656	-2.898389	0.218516
H	-3.578721	-3.218981	0.339010
N	-0.824189	0.311334	-0.295388
N	0.215289	0.870633	0.110830
C	1.297690	1.492237	0.514659
C	1.812278	2.714574	0.009755
C	2.266714	1.081357	1.508980
N	3.246591	1.956412	1.584377
N	2.971474	2.907704	0.646369
C	3.968680	3.917432	0.402525
H	3.508529	4.788262	-0.066052
H	4.407815	4.202813	1.358833
H	4.760545	3.530303	-0.247503
C	1.250794	3.570223	-1.058595
H	0.242074	3.213690	-1.281591
H	1.190621	4.618795	-0.749124
H	1.838276	3.525951	-1.982870
C	2.170109	-0.082925	2.428270
H	1.961502	0.245446	3.451325
H	1.361860	-0.745957	2.113454
H	3.107382	-0.645123	2.442899
H	-1.697316	-4.844927	0.431662
N	1.398190	-1.586407	-0.404190
H	1.477477	-0.620623	-0.683357
N	-3.262483	-0.561646	-0.062505
H	-2.891124	0.362494	-0.263811
C	2.584748	-2.250007	-0.255031
O	2.726877	-3.386699	0.170613
C	-4.614851	-0.708235	0.053341
O	-5.207163	-1.754451	0.279844
C	5.088371	-1.697681	-0.503185
C	5.767035	-0.551177	-1.006896
C	4.796928	0.313025	-1.412475
O	3.571114	-0.213697	-1.198194
C	3.756121	-1.443158	-0.638537
H	5.509923	-2.600466	-0.087851
H	6.833605	-0.389696	-1.061262
H	4.816071	1.291797	-1.866581
C	-6.704217	0.788735	-0.065152
C	-6.866571	2.183845	-0.298631

C -5.612941 2.685250 -0.473766
O -4.689118 1.704825 -0.365893
C -5.363530 0.547224 -0.115886
H -7.463125 0.043438 0.118557
H -7.790369 2.742622 -0.332447
H -5.230134 3.674164 -0.673874
24
4pzHCl1A-ts-B-m ; E = -1064.73982498 Hartree
C 2.077083 0.652267 0.249680
C 3.315610 0.968978 -0.279365
C 1.561451 -0.669734 0.182132
H 3.673643 1.989007 -0.183294
C 4.069370 0.004097 -0.942047
C 2.352669 -1.644473 -0.479406
H 1.975957 -2.659184 -0.538808
C 3.565342 -1.292339 -1.044221
H 4.139933 -2.058345 -1.557021
Cl 1.103963 1.864869 1.029464
N 0.389677 -0.966612 0.706228
N -0.691045 -1.260044 1.206871
C -1.829279 -0.758283 0.560916
C -1.949391 0.119926 -0.513104
C -3.157983 -1.057639 0.932122
N -4.024447 -0.428380 0.156216
N -3.265645 0.280199 -0.706560
C -3.920850 1.100600 -1.697114
H -4.511442 0.472115 -2.367273
H -4.581535 1.814206 -1.201127
H -3.164209 1.637580 -2.269343
H -1.200605 0.628560 -1.101403
H -3.495128 -1.706252 1.728295
H 5.032105 0.263006 -1.367754
24
4pzHCl1A-ts-B-n ; E = -1064.73984731 Hartree
C -2.244315 -0.671891 0.055940
C -3.532503 -0.725637 -0.445874
C -1.620116 0.569513 0.348132
H -3.971104 -1.699721 -0.637243
C -4.236555 0.443775 -0.719764
C -2.357861 1.751077 0.080500
H -1.896889 2.707542 0.298935
C -3.628454 1.671520 -0.461310
H -4.161481 2.595097 -0.668228
Cl -1.343402 -2.128437 0.357781
N -0.399638 0.612562 0.844336
N 0.726692 0.661575 1.324990
C 1.784656 0.381233 0.448873
C 3.117862 0.444964 0.829045
C 1.814223 -0.015163 -0.911086
N 3.057475 -0.177677 -1.326100
N 3.830661 0.108733 -0.256383
C 5.264702 0.023586 -0.389556
H 5.724825 0.269706 0.567713
H 5.548066 -0.989523 -0.682178
H 5.608577 0.725482 -1.152330
H 3.569278 0.701531 1.775836
H 0.980450 -0.193844 -1.575534
H -5.240046 0.392950 -1.126547

```
24
4pzHCl1A-ts-H-m ; E = -1064.72983880 Hartree
C       -1.891053       -0.614856       -0.271214
C       -3.035343       -1.329496        0.073311
C       -1.822624        0.768114       -0.070010
H       -3.063530       -2.398923       -0.108450
C       -4.126485       -0.671598        0.627686
C       -2.950488        1.413443        0.445890
H       -2.885321        2.492320        0.554810
C       -4.085597        0.708953        0.813781
H       -4.939246        1.231542        1.233842
Cl       -0.590654       -1.497186       -1.024440
N       -0.746037        1.668232       -0.401537
N        0.409914        1.229926       -0.298388
C        1.645562        0.813550       -0.143325
C        2.103079       -0.348880        0.521330
C        2.866270        1.431966       -0.582681
N        3.923855        0.734997       -0.234217
N        3.433242       -0.328901        0.458617
C        4.371532       -1.256305        1.041290
H        3.823054       -2.092409        1.475779
H        5.046343       -1.622837        0.265464
H        4.958017       -0.757848        1.817081
H        1.551157       -1.122882        1.033286
H        2.967868        2.342102       -1.158935
H       -5.013153       -1.238028        0.894922
24
4pzHCl1A-ts-H-n ; E = -1064.72919826 Hartree
C       -2.010750       -0.612014       -0.191691
C       -3.242156       -1.157988        0.161228
C       -1.820011        0.774524       -0.194871
H       -3.364924       -2.235927        0.141482
C       -4.296658       -0.326963        0.519713
C       -2.909001        1.591010        0.122865
H       -2.744347        2.663602        0.072729
C       -4.131030        1.056460        0.499087
H       -4.954360        1.711970        0.765002
Cl       -0.759407       -1.710815       -0.693487
N       -0.638376        1.508029       -0.573826
N        0.456426        0.990925       -0.303706
C        1.630349        0.499004        0.021383
C        2.896639        0.920625       -0.443300
C        1.960624       -0.573375        0.921676
N        3.259015       -0.764288        1.000011
N        3.802719        0.136525        0.138136
C        5.231219        0.118512       -0.059001
H        5.521176        0.984706       -0.654354
H        5.726995        0.156685        0.912488
H        5.528459       -0.798139       -0.574878
H        3.155148        1.687710       -1.158565
H        1.279048       -1.160088        1.521824
H       -5.251091       -0.763774        0.796709
24
4pzHCl1B-ts-B-m ; E = -1064.73982867 Hartree
C       -2.356218       -1.641744       -0.485171
C       -3.569369       -1.285477       -1.046344
C       -1.562365       -0.670948        0.179012
H       -4.146058       -2.048540       -1.561173
```

C	-4.071213	0.011330	-0.938156
C	-2.075510	0.651741	0.251834
Cl	-1.098255	1.860533	1.034212
C	-3.314615	0.972501	-0.273316
H	-3.670707	1.992784	-0.172841
H	-1.981272	-2.656826	-0.549182
N	-0.390395	-0.971750	0.700366
N	0.690220	-1.267917	1.199667
C	1.828575	-0.763372	0.556086
C	1.948750	0.123361	-0.510903
C	3.157225	-1.069591	0.921733
N	4.023739	-0.436522	0.148969
N	3.265020	0.280860	-0.706572
C	3.920264	1.118455	-1.682581
H	3.163192	1.603804	-2.298668
H	4.521582	1.875771	-1.175162
H	4.570711	0.507768	-2.311904
H	1.199862	0.639724	-1.092824
H	3.494123	-1.727146	1.711037
H	-5.034329	0.273379	-1.361061

24

4pzHCl1B-ts-B-n ; E = -1064.73984738 Hartree

C	2.359092	1.750926	0.078785
C	3.629879	1.669944	-0.462351
C	1.620481	0.570147	0.347471
H	4.163620	2.592940	-0.670014
C	4.237276	0.441514	-0.719202
C	2.243907	-0.671993	0.056725
Cl	1.341607	-2.127560	0.358893
C	3.532266	-0.727130	-0.444512
H	3.970240	-1.701709	-0.634797
H	1.898648	2.707932	0.295957
N	0.399853	0.614491	0.843196
N	-0.726435	0.663931	1.323916
C	-1.784450	0.382133	0.448346
C	-3.117617	0.446577	0.828550
C	-1.814187	-0.016324	-0.911011
N	-3.057499	-0.179407	-1.325628
N	-3.830551	0.108658	-0.256260
C	-5.264598	0.023081	-0.389103
H	-5.607668	0.719584	-1.157120
H	-5.548621	-0.991971	-0.674367
H	-5.724874	0.276553	0.566174
H	-3.568889	0.704676	1.774960
H	-0.980589	-0.196028	-1.575338
H	5.240948	0.389577	-1.125401

24

4pzHCl1B-ts-H-m ; E = -1064.73075532 Hartree

C	1.029105	1.336958	-0.406494
C	1.920393	2.396555	-0.342166
C	1.450899	0.024445	-0.186160
H	1.578525	3.410245	-0.528835
C	3.260870	2.152808	-0.045326
C	2.801806	-0.205323	0.093121
Cl	3.378911	-1.817677	0.370923
C	3.701885	0.852752	0.174339
H	4.741027	0.647687	0.408104
H	-0.017385	1.492748	-0.653321

N	0.580366	-1.115573	-0.302692
N	-0.634564	-0.860223	-0.265196
C	-1.924405	-0.615138	-0.181770
C	-2.575287	0.564634	0.250486
C	-3.023325	-1.493374	-0.476274
N	-4.185152	-0.919461	-0.257605
N	-3.884953	0.322560	0.206194
C	-4.970581	1.183496	0.607821
H	-4.572005	2.165922	0.862186
H	-5.681162	1.276338	-0.215558
H	-5.482931	0.758950	1.474601
H	-2.167159	1.499790	0.606552
H	-2.966970	-2.503372	-0.860139
H	3.968544	2.974202	0.010344

24

4pzHCl1B-ts-H-n ; E = -1064.73140827 Hartree

C	1.237515	1.403490	0.275485
C	2.258989	2.338255	0.215536
C	1.500631	0.038361	0.149418
H	2.037882	3.395713	0.325050
C	3.572677	1.913052	0.020647
C	2.825194	-0.373181	-0.029812
Cl	3.208265	-2.058511	-0.188870
C	3.856576	0.557830	-0.103893
H	4.872981	0.211870	-0.257910
H	0.206649	1.703289	0.440827
N	0.488746	-0.978618	0.260513
N	-0.682854	-0.577936	0.158426
C	-1.927295	-0.177181	0.008939
C	-3.099849	-0.925019	0.262769
C	-2.449438	1.083034	-0.447938
N	-3.763750	1.092577	-0.471296
N	-4.133984	-0.133738	-0.015691
C	-5.542026	-0.413785	0.127360
H	-5.670165	-1.456134	0.419899
H	-5.976229	0.238252	0.889005
H	-6.044998	-0.233924	-0.824601
H	-3.212817	-1.933111	0.634556
H	-1.890078	1.947921	-0.780756
H	4.381299	2.635686	-0.030010

24

4pzHCl2-ts-B-m ; E = -1524.17686965 Hartree

C	2.245300	-1.029455	0.194695
C	3.442381	-0.693576	0.799670
C	1.360763	-0.034458	-0.302741
H	4.075067	-1.493972	1.168322
C	3.831048	0.638471	0.917700
C	1.771618	1.315889	-0.133102
Cl	0.686173	2.557176	-0.682134
C	2.986608	1.638151	0.443031
H	3.255816	2.685501	0.529584
Cl	1.757154	-2.687988	0.046076
N	0.221609	-0.338628	-0.879988
N	-0.830743	-0.583503	-1.457251
C	-2.004110	-0.402913	-0.721534
C	-2.203771	0.091650	0.565893
C	-3.299859	-0.694875	-1.201424
N	-4.218802	-0.411331	-0.295206

```
N       -3.527347        0.065038        0.763246
C       -4.256510        0.485918        1.936139
H       -3.548007        0.826426        2.691282
H       -4.935271        1.300207        1.674464
H       -4.836518       -0.352557        2.326620
H       -1.504867        0.454031        1.304963
H       -3.575009       -1.099667       -2.165322
H        4.778394        0.893994        1.377493
24
4pzHCl2-ts-B-n ; E = -1524.17720122 Hartree
C        2.121249       -1.204834        0.007557
C        3.398293       -1.191845        0.537721
C        1.428846       -0.000794       -0.289102
H        3.879525       -2.142192        0.742965
C        4.045886        0.011713        0.802011
C        2.111370        1.209869        0.003642
Cl        1.278958        2.700365       -0.315862
C        3.388827        1.209065        0.532994
H        3.862535        2.163986        0.734493
Cl        1.301039       -2.703274       -0.307122
N        0.220887       -0.006440       -0.805623
N       -0.895189       -0.010129       -1.306677
C       -1.976562       -0.005269       -0.422396
C       -3.297688       -0.009762       -0.850509
C       -2.053299        0.004781        0.992900
N       -3.310613        0.006302        1.393447
N       -4.047195       -0.002690        0.259808
C       -5.486522       -0.003194        0.364638
H       -5.815673       -0.888660        0.912233
H       -5.817594        0.890839        0.896970
H       -5.914925       -0.012265       -0.637790
H       -3.714815       -0.017402       -1.846502
H       -1.244130        0.010982        1.709725
H        5.047506        0.016477        1.215241
24
4pzHCl2-ts-H-m ; E = -1524.16381404 Hartree
C        1.360627        1.294392       -0.271748
C        2.369061        2.129535        0.198354
C        1.493856       -0.098047       -0.263990
H        2.230452        3.204414        0.158183
C        3.538758        1.574881        0.700359
C        2.696984       -0.619729        0.228840
Cl        2.919413       -2.341098        0.265973
C        3.707103        0.194677        0.721254
H        4.611831       -0.255776        1.113797
Cl       -0.063385        2.051283       -0.933628
N        0.560869       -1.041281       -0.836513
N       -0.635452       -0.851804       -0.579013
C       -1.909529       -0.708231       -0.296397
C       -2.507282        0.223033        0.585654
C       -3.035767       -1.452155       -0.789563
N       -4.166559       -1.029363       -0.273755
N       -3.814869       -0.015029        0.566527
C       -4.860141        0.650756        1.305162
H       -4.418021        1.435952        1.918711
H       -5.578697        1.087929        0.608752
H       -5.375690       -0.070140        1.943374
H       -2.058236        0.995799        1.192063
```

```
H       -3.021761       -2.265479       -1.503010
H        4.327687        2.221813        1.070011
24
4pzHCl2-ts-H-n ; E = -1524.16349670 Hartree
C        1.547114        1.274034       -0.193188
C        2.678267        1.957106        0.241694
C        1.515931       -0.122729       -0.266405
H        2.665530        3.041145        0.269460
C        3.805667        1.242528        0.624626
C        2.679071       -0.808087        0.104980
Cl        2.696645       -2.543189        0.034700
C        3.810730       -0.146478        0.560960
H        4.680471       -0.720471        0.860261
Cl        0.181048        2.225258       -0.705829
N        0.437895       -0.911973       -0.815640
N       -0.703605       -0.606578       -0.444968
C       -1.927026       -0.340400       -0.047915
C       -3.129147       -0.919702       -0.514806
C       -2.384270        0.587010        0.951485
N       -3.691365        0.565670        1.077203
N       -4.120135       -0.352863        0.166181
C       -5.538975       -0.587033        0.050824
H       -5.713241       -1.359549       -0.698375
H       -5.934856       -0.912402        1.015038
H       -6.041126        0.335881       -0.247449
H       -3.290273       -1.669816       -1.275564
H       -1.779890        1.241373        1.565014
H        4.688891        1.771382        0.967861
24
4pzHF1A-ts-B-m ; E = -704.442254488 Hartree
C        1.965486        0.713724        0.545500
C        3.179529        1.320925        0.325947
C        1.666938       -0.590126        0.075339
H        3.346598        2.311584        0.736463
C        4.142437        0.671658       -0.450596
C        2.666238       -1.236603       -0.691706
H        2.458578       -2.229501       -1.074297
C        3.862178       -0.590628       -0.969239
H        4.601507       -1.100286       -1.579871
F        0.997796        1.345961        1.239007
N        0.490305       -1.127305        0.337869
N       -0.588415       -1.640119        0.636188
C       -1.729592       -0.913896        0.255815
C       -1.849761        0.398486       -0.190802
C       -3.053152       -1.401781        0.299144
N       -3.916542       -0.480443       -0.097420
N       -3.162069        0.601024       -0.381544
C       -3.817484        1.816765       -0.799299
H       -4.436606        2.206686        0.012066
H       -3.060311        2.553489       -1.068497
H       -4.450957        1.609017       -1.663646
H       -1.104633        1.167739       -0.326446
H       -3.389596       -2.386847        0.591273
H        5.095396        1.151235       -0.644383
24
4pzHF1A-ts-B-n ; E = -704.442262177 Hartree
C        2.166784        0.802933        0.375208
C        3.438577        1.223664        0.064565
```

```
C        1.740406       -0.535499        0.183665
H        3.703208        2.258149        0.258413
C        4.335052        0.330957       -0.528259
C        2.672569       -1.428758       -0.396189
H        2.366745       -2.455306       -0.563532
C        3.930866       -0.979544       -0.770886
H        4.617542       -1.679870       -1.237055
F        1.268459        1.657367        0.897548
N        0.513008       -0.884724        0.522452
N       -0.613694       -1.201946        0.901138
C       -1.672515       -0.497179        0.304483
C       -3.008125       -0.824752        0.486066
C       -1.691604        0.643267       -0.535295
N       -2.931293        0.982606       -0.842570
N       -3.712408        0.076248       -0.218374
C       -5.143175        0.156855       -0.381321
H       -5.415866       -0.041252       -1.420746
H       -5.616299       -0.581491        0.266676
H       -5.485069        1.156574       -0.107016
H       -3.469183       -1.620471        1.051884
H       -0.852197        1.228685       -0.882577
H        5.332676        0.661742       -0.794721
24
4pzHF1A-ts-H-m ; E = -704.438089288 Hartree
C        1.808935       -0.719833       -0.258335
C        2.927251       -1.531346       -0.163935
C        1.872305        0.658711       -0.026086
H        2.819842       -2.592634       -0.363276
C        4.155405       -0.968212        0.171003
C        3.122940        1.199126        0.283487
H        3.159923        2.273539        0.439492
C        4.254452        0.403245        0.395029
H        5.210980        0.848317        0.649633
F        0.647928       -1.300701       -0.612583
N        0.810183        1.626320       -0.108280
N       -0.351413        1.183520       -0.092022
C       -1.595765        0.767118       -0.054021
C       -2.071020       -0.542479        0.195321
C       -2.809575        1.517662       -0.230499
N       -3.878039        0.763193       -0.108089
N       -3.400179       -0.482350        0.165729
C       -4.351138       -1.541857        0.395660
H       -4.955300       -1.314043        1.277032
H       -5.008481       -1.636474       -0.471079
H       -3.812007       -2.476570        0.551505
H       -1.525593       -1.452703        0.391873
H       -2.900076        2.572273       -0.455348
H        5.032753       -1.602998        0.246309
24
4pzHF1A-ts-H-n ; E = -704.437401756 Hartree
C        1.973090       -0.713538        0.211449
C        3.184811       -1.377297        0.111185
C        1.877717        0.675285        0.064434
H        3.200472       -2.454550        0.240621
C        4.344992       -0.651187       -0.142146
C        3.062005        1.380580       -0.163307
H        2.973434        2.459589       -0.252567
C        4.284518        0.733994       -0.279204
```

H	5.187546	1.305007	-0.469876
F	0.882100	-1.446511	0.485220
N	0.704111	1.499728	0.166996
N	-0.394506	0.923808	0.079943
C	-1.577950	0.365803	-0.033292
C	-2.839825	0.965780	0.184535
C	-1.926291	-0.977925	-0.412826
N	-3.227578	-1.162154	-0.428576
N	-3.759116	0.030027	-0.042858
C	-5.192035	0.129486	0.084092
H	-5.458017	1.157012	0.333442
H	-5.661108	-0.153112	-0.860475
H	-5.543593	-0.542509	0.870691
H	-3.088155	1.969375	0.498381
H	-1.250719	-1.777079	-0.682629
H	5.294671	-1.170873	-0.222621

24

4pzHF1B-ts-B-m ; E = -704.442254493 Hartree

C	-2.666346	-1.236625	-0.691575
C	-3.862252	-0.590583	-0.969095
C	-1.666953	-0.590160	0.075356
H	-4.601651	-1.100231	-1.579651
C	-4.142389	0.671763	-0.450536
C	-1.965385	0.713747	0.545441
F	-0.997647	1.345940	1.238813
C	-3.179399	1.321016	0.325919
H	-3.346375	2.311710	0.736386
H	-2.458786	-2.229575	-1.074084
N	-0.490343	-1.127410	0.337845
N	0.588405	-1.640184	0.636132
C	1.729563	-0.913952	0.255712
C	1.849727	0.398483	-0.190750
C	3.053127	-1.401836	0.298998
N	3.916512	-0.480451	-0.097463
N	3.162036	0.601049	-0.381458
C	3.817435	1.816822	-0.799146
H	4.451826	1.608809	-1.662747
H	3.060274	2.553140	-1.069490
H	4.435625	2.207411	0.012615
H	1.104613	1.167725	-0.326379
H	3.389583	-2.386924	0.591001
H	-5.095322	1.151399	-0.644313

24

4pzHF1B-ts-B-n ; E = -704.442262211 Hartree

C	-2.673023	-1.428719	-0.395950
C	-3.931219	-0.979209	-0.770633
C	-1.740495	-0.535616	0.183557
H	-4.618152	-1.679398	-1.236627
C	-4.334997	0.331442	-0.528118
C	-2.166548	0.802917	0.375179
F	-1.268081	1.657105	0.897772
C	-3.438273	1.223944	0.064636
H	-3.702667	2.258484	0.258511
H	-2.367503	-2.455386	-0.563122
N	-0.513067	-0.885058	0.522031
N	0.613698	-1.202572	0.900354
C	1.672474	-0.497474	0.304047
C	3.008086	-0.825014	0.485695

```
C        1.691594        0.643338       -0.535236
N        2.931305        0.982922       -0.842159
N        3.712407        0.076385       -0.218195
C        5.143187        0.157048       -0.381017
H        5.484850        1.156995       -0.107273
H        5.616361       -0.580822        0.267494
H        5.416045       -0.041640       -1.420290
H        3.469065       -1.621026        1.051255
H        0.852160        1.228689       -0.882527
H       -5.332546        0.662484       -0.794546
24
4pzHF1B-ts-H-m ; E = -704.436550574 Hartree
C        1.396485        1.051566       -0.361079
C        2.389513        2.020321       -0.368594
C        1.694861       -0.282927       -0.079183
H        2.143649        3.052253       -0.599951
C        3.708372        1.662754       -0.086382
C        3.025742       -0.617498        0.182817
F        3.343632       -1.886035        0.446866
C        4.030271        0.338571        0.192075
H        5.045916        0.030016        0.415219
H        0.364885        1.298494       -0.594975
N        0.738956       -1.357562       -0.099898
N       -0.451471       -1.000991       -0.124241
C       -1.717959       -0.645321       -0.114403
C       -2.283891        0.603926        0.232597
C       -2.874185       -1.442177       -0.420885
N       -3.991486       -0.762486       -0.285394
N       -3.608102        0.468113        0.141530
C       -4.631910        1.426577        0.478431
H       -4.169690        2.399607        0.647403
H       -5.342297        1.495303       -0.347096
H       -5.162889        1.110933        1.380336
H       -1.814640        1.516654        0.570978
H       -2.887557       -2.468468       -0.763101
H        4.490862        2.415254       -0.086961
24
4pzHF1B-ts-H-n ; E = -704.437144932 Hartree
C       -1.581293        1.098071        0.240951
C       -2.684409        1.939119        0.242372
C       -1.728076       -0.280428        0.076504
H       -2.554428        3.008274        0.379176
C       -3.962898        1.405990        0.074290
C       -3.019984       -0.790592       -0.074139
F       -3.193370       -2.105340       -0.225106
C       -4.134113        0.035018       -0.085890
H       -5.114751       -0.409015       -0.218990
H       -0.576662        1.484905        0.384338
N       -0.649200       -1.231141        0.107247
N        0.490899       -0.737920        0.051557
C        1.703703       -0.235906       -0.042599
C        2.924412       -0.898644        0.218746
C        2.136572        1.079605       -0.432027
N        3.446726        1.193216       -0.414208
N        3.900153       -0.015019        0.009463
C        5.318925       -0.182940        0.208658
H        5.656463        0.431641        1.047062
H        5.528459       -1.232602        0.415985
```

```
H        5.847099        0.126238       -0.694959
H        3.107405       -1.907997        0.557625
H        1.520641        1.908967       -0.755300
H       -4.830976        2.057939        0.071402
24
4pzHF2-ts-B-m ; E = -803.582855184 Hartree
C       -2.610755        0.943882        0.357243
C       -3.788500        0.301340        0.677541
C       -1.505973        0.266089       -0.220757
H       -4.582973        0.877433        1.139123
C       -3.947297       -1.049113        0.366007
C       -1.705363       -1.116540       -0.463235
F       -0.664446       -1.790089       -0.983781
C       -2.900629       -1.759034       -0.221812
H       -2.988258       -2.811994       -0.466337
F       -2.448305        2.238896        0.640213
N       -0.372672        0.866293       -0.510919
N        0.672943        1.446981       -0.814345
C        1.855875        0.838672       -0.368160
C        2.070672       -0.414851        0.198170
C        3.139055        1.423003       -0.442901
N        4.064831        0.611572        0.042063
N        3.391445       -0.495701        0.417436
C        4.131693       -1.610663        0.956645
H        4.715971       -1.278084        1.816770
H        3.430161       -2.385080        1.267561
H        4.808540       -2.011611        0.198616
H        1.385437       -1.223904        0.400675
H        3.401970        2.401338       -0.820456
H       -4.884609       -1.548093        0.584070
24
4pzHF2-ts-B-n ; E = -803.583037222 Hartree
C       -2.611874       -1.096190       -0.202830
C       -3.861115       -0.640600       -0.569404
C       -1.582427       -0.224985        0.236930
H       -4.591608       -1.360495       -0.921910
C       -4.167863        0.714081       -0.441627
C       -1.933680        1.147216        0.297036
F       -0.971541        1.995416        0.691608
C       -3.199650        1.610819        0.009350
H       -3.402833        2.671380        0.109612
F       -2.306164       -2.392815       -0.309768
N       -0.382755       -0.650263        0.568536
N        0.727049       -1.059863        0.917185
C        1.823101       -0.408319        0.331595
C        3.134272       -0.835037        0.487919
C        1.915938        0.749045       -0.480759
N        3.173533        1.006629       -0.794341
N        3.894100        0.030929       -0.202076
C        5.324652        0.010552       -0.383885
H        5.740666        0.979461       -0.101709
H        5.751797       -0.768503        0.248202
H        5.569311       -0.192474       -1.429388
H        3.542006       -1.677353        1.026683
H        1.117602        1.402262       -0.802378
H       -5.159214        1.070815       -0.696742
24
4pzHF2-ts-H-m ; E = -803.577850517 Hartree
```

```
C        1.508363        1.013490        0.313157
C        2.546685        1.918377        0.165897
C        1.667046       -0.364268        0.123769
H        2.355163        2.971444        0.339789
C        3.806946        1.446147       -0.186767
C        2.959693       -0.788217       -0.209297
F        3.168951       -2.091788       -0.391700
C        4.021407        0.085378       -0.378855
H        4.991649       -0.311652       -0.654474
F        0.310077        1.495692        0.683310
N        0.684819       -1.399089        0.319238
N       -0.501894       -1.041633        0.223794
C       -1.770522       -0.723692        0.118429
C       -2.327633        0.526382       -0.243655
C       -2.934104       -1.544067        0.321154
N       -4.047446       -0.880351        0.109846
N       -3.648940        0.375469       -0.238313
C       -4.665474        1.350347       -0.549149
H       -5.324793        1.479859        0.311758
H       -4.186498        2.299281       -0.791343
H       -5.256530        1.007815       -1.401470
H       -1.841087        1.458738       -0.486701
H       -2.957916       -2.584938        0.616231
H        4.628544        2.144284       -0.308329
24
4pzHF2-ts-H-n ; E = -803.577403869 Hartree
C        1.700008        1.020389       -0.248435
C        2.851501        1.772585       -0.082370
C        1.684559       -0.375674       -0.143709
H        2.794171        2.850308       -0.187125
C        4.047430        1.121199        0.202027
C        2.918181       -0.982751        0.121195
F        2.959458       -2.312019        0.219749
C        4.088320       -0.265261        0.307445
H        5.005152       -0.799576        0.528551
F        0.567520        1.670660       -0.552687
N        0.571065       -1.257840       -0.372500
N       -0.555742       -0.760386       -0.203099
C       -1.768853       -0.292293       -0.022926
C       -2.996114       -0.956271       -0.254878
C       -2.190563        0.994703        0.463611
N       -3.499363        1.093066        0.522004
N       -3.964996       -0.105606        0.072197
C       -5.392428       -0.300624        0.005330
H       -5.826999       -0.169651        0.998618
H       -5.832895        0.431623       -0.675012
H       -5.598303       -1.308419       -0.355990
H       -3.188689       -1.951753       -0.628261
H       -1.559594        1.819721        0.762909
H        4.955353        1.699321        0.338792
28
4pzHOMe1A-ts-B-m ; E = -719.702897244 Hartree
C        1.912059       -0.545617       -0.248393
C        3.152519       -0.982275        0.181542
C        1.553983        0.833529       -0.135408
H        3.424408       -2.027313        0.078309
C        4.045122       -0.092719        0.790984
C        2.483028        1.718799        0.452137
```

C	3.693493	1.242987	0.939944
H	4.379847	1.937775	1.414627
N	0.363054	1.221627	-0.554874
N	-0.725798	1.572946	-1.007679
C	-1.848158	0.929647	-0.455181
C	-1.928392	-0.162195	0.402452
C	-3.189665	1.286739	-0.708155
N	-4.028857	0.493216	-0.060761
N	-3.238710	-0.377005	0.600686
C	-3.854884	-1.416706	1.387908
H	-4.540599	-0.969294	2.110041
H	-4.413347	-2.097471	0.740821
H	-3.077492	-1.969331	1.916186
H	-1.152351	-0.781551	0.825808
H	-3.557054	2.093037	-1.327745
H	5.006537	-0.454017	1.139905
H	2.210866	2.764685	0.538942
O	0.942800	-1.322252	-0.807201
C	1.253299	-2.676585	-1.029586
H	2.110616	-2.784513	-1.705776
H	1.473026	-3.203480	-0.091278
H	0.370919	-3.119612	-1.493301

28

4pzHOMe1A-ts-B-n ; E = -719.703154772 Hartree

C	2.070680	0.551147	0.163915
C	3.361526	0.825374	-0.252485
C	1.611751	-0.800148	0.241383
H	3.709032	1.851897	-0.297059
C	4.209830	-0.210468	-0.661272
C	2.496317	-1.830925	-0.140143
C	3.763313	-1.525176	-0.621391
H	4.415465	-2.332724	-0.940266
N	0.372569	-1.031335	0.638160
N	-0.762024	-1.221581	1.074306
C	-1.797316	-0.592560	0.358642
C	-3.142719	-0.793124	0.627293
C	-1.778420	0.340371	-0.706477
N	-3.006278	0.682244	-1.058942
N	-3.817651	-0.018769	-0.239904
C	-5.245530	0.105081	-0.395863
H	-5.523037	1.160533	-0.373275
H	-5.562462	-0.326646	-1.348701
H	-5.739214	-0.419301	0.422960
H	-3.630647	-1.420897	1.357998
H	-0.916465	0.778074	-1.188836
H	5.211959	0.022907	-1.004809
H	2.146394	-2.855476	-0.081512
O	1.141658	1.469370	0.541216
C	1.522771	2.823521	0.506139
H	2.353750	3.024093	1.194385
H	1.814463	3.136294	-0.505125
H	0.647982	3.393816	0.820712

28

4pzHOMe1A-ts-H-m ; E = -719.695229731 Hartree

C	2.737636	-0.215014	0.064599
C	3.624139	0.865362	0.087282
C	1.365787	0.022866	-0.164122
H	4.679529	0.704997	0.273388

```
C        3.157359        2.159044       -0.129065
C        0.925005        1.325743       -0.381458
C        1.807114        2.397486       -0.368576
H        1.450875        3.406777       -0.550075
N        0.506893       -1.127128       -0.223635
N       -0.710807       -0.878388       -0.212969
C       -2.004646       -0.644397       -0.169009
C       -2.677748        0.493910        0.330717
C       -3.086281       -1.495119       -0.583479
N       -4.260138       -0.939629       -0.370305
N       -3.987742        0.250860        0.217908
C       -5.087824        1.047618        0.701242
H       -4.741432        2.065087        0.886908
H       -5.871274        1.059318       -0.057653
H       -5.492502        0.624757        1.625398
H       -2.289153        1.392052        0.788842
H       -3.009987       -2.461985       -1.062834
H        3.862872        2.984668       -0.112856
H       -0.133175        1.468920       -0.582105
O        3.105201       -1.497066        0.265015
C        4.470683       -1.764071        0.476943
H        5.083829       -1.448347       -0.377004
H        4.844132       -1.275024        1.386040
H        4.549882       -2.845279        0.594392
28
4pzHOMe1A-ts-H-n ; E = -719.695612043 Hartree
C        2.758146       -0.339807        0.001912
C        3.757695        0.636965        0.013290
C        1.409125        0.054559       -0.122673
H        4.798696        0.355632        0.119962
C        3.422947        1.982585       -0.111611
C        1.101167        1.406395       -0.250145
C        2.094670        2.375663       -0.247598
H        1.839909        3.425274       -0.356154
N        0.428863       -0.994308       -0.172740
N       -0.753584       -0.620791       -0.080580
C       -2.009453       -0.251229        0.054358
C       -3.162922       -0.984109       -0.302550
C       -2.561448        0.962007        0.595114
N       -3.877731        0.957157        0.575398
N       -4.217704       -0.220829       -0.003896
C       -5.610317       -0.476403       -0.276581
H       -5.742824       -1.534075       -0.506995
H       -6.195341       -0.216809        0.606862
H       -5.952541        0.127948       -1.121459
H       -3.253318       -1.953671       -0.770122
H       -2.022681        1.793104        1.031743
H        4.214354        2.726453       -0.105395
H        0.054637        1.672784       -0.368480
O        2.995217       -1.663009        0.118632
C        4.333563       -2.082783        0.231164
H        4.926399       -1.791251       -0.645525
H        4.810863       -1.682078        1.134898
H        4.302151       -3.170911        0.296240
28
4pzHOMe1B-ts-B-m ; E = -719.702897244 Hartree
C        2.482993       -1.718833        0.452121
C        3.693476       -1.243083        0.939937
```

```
C        1.553977       -0.833516       -0.135407
H        4.379801       -1.937908        1.414608
C        4.045162        0.092611        0.790996
C        1.912111        0.545619       -0.248367
C        3.152595        0.982211        0.181567
H        3.424534        2.027236        0.078342
N        0.363041       -1.221571       -0.554884
N       -0.725811       -1.572851       -1.007716
C       -1.848189       -0.929583       -0.455211
C       -1.928470        0.162240        0.402437
C       -3.189682       -1.286726       -0.708198
N       -4.028908       -0.493244       -0.060799
N       -3.238799        0.376997        0.600668
C       -3.855007        1.416654        1.387925
H       -3.077672        1.969191        1.916386
H       -4.413338        2.097509        0.740823
H       -4.540849        0.969189        2.109904
H       -1.152447        0.781608        0.825803
H       -3.557027       -2.093022       -1.327815
H        5.006594        0.453864        1.139917
O        0.942903        1.322316       -0.807173
C        1.253469        2.676642       -1.029513
H        1.473240        3.203490       -0.091190
H        2.110779        2.784550       -1.705715
H        0.371104        3.119732       -1.493197
H        2.210788       -2.764709        0.538908
28
4pzHOMe1B-ts-B-n ; E = -719.703154772 Hartree
C        2.496242       -1.830944        0.140081
C        3.763239       -1.525271        0.621376
C        1.611727       -0.800112       -0.241418
H        4.415342       -2.332862        0.940244
C        4.209816       -0.210586        0.661315
C        2.070723        0.551157       -0.163907
C        3.361571        0.825307        0.252536
H        3.709124        1.851813        0.297150
N        0.372555       -1.031261       -0.638253
N       -0.762053       -1.221470       -1.074373
C       -1.797338       -0.592494       -0.358660
C       -3.142735       -0.792986       -0.627393
C       -1.778465        0.340235        0.706634
N       -3.006330        0.682072        1.059114
N       -3.817685       -0.018769        0.239911
C       -5.245567        0.105135        0.395797
H       -5.562494       -0.326283        1.348773
H       -5.523109        1.160573        0.372853
H       -5.739227       -0.419535       -0.422856
H       -3.630623       -1.420628       -1.358242
H       -0.916516        0.777815        1.189109
H        5.211944        0.022733        1.004895
O        1.141765        1.469436       -0.541224
C        1.522950        2.823565       -0.506107
H        1.814611        3.136306        0.505176
H        2.353973        3.024103       -1.194311
H        0.648207        3.393913       -0.820713
H        2.146274       -2.855477        0.081406
28
4pzHOMe1B-ts-H-m ; E = -719.696239438 Hartree
```

C	3.044218	-1.489500	0.281477
C	4.218738	-0.767574	0.440543
C	1.824996	-0.865213	0.020941
H	5.153166	-1.275540	0.655520
C	4.177821	0.616089	0.311442
C	1.795501	0.541827	-0.122485
C	2.979710	1.266736	0.025844
H	2.976779	2.345333	-0.081409
N	0.736681	-1.787081	-0.129571
N	-0.412390	-1.320156	-0.122208
C	-1.652755	-0.896466	-0.108113
C	-2.152851	0.281842	0.489026
C	-2.832870	-1.499478	-0.664618
N	-3.907706	-0.772601	-0.440923
N	-3.477674	0.280405	0.296078
C	-4.458327	1.182045	0.845545
H	-5.226384	1.359722	0.091853
H	-4.926007	0.752358	1.736798
H	-3.976417	2.125213	1.107629
H	-1.639979	1.039626	1.061163
H	-2.890907	-2.403694	-1.255809
H	5.083748	1.204475	0.423689
O	0.606310	1.118373	-0.420375
C	0.600783	2.486626	-0.754996
H	0.877689	3.117993	0.099324
H	1.277771	2.700059	-1.591292
H	-0.423746	2.714824	-1.052503
H	3.021653	-2.572928	0.356778

28

4pzHOMe1B-ts-H-n ; E = -719.696350169 Hartree

C	2.982965	-1.647358	0.057911
C	4.232623	-1.047704	0.126214
C	1.810190	-0.898844	-0.034286
H	5.130323	-1.651472	0.209663
C	4.313034	0.339482	0.079450
C	1.902522	0.512203	-0.094801
C	3.161275	1.114347	-0.035902
H	3.252170	2.193418	-0.080424
N	0.629391	-1.708565	-0.109904
N	-0.466216	-1.139645	0.008958
C	-1.653822	-0.600622	0.144925
C	-2.886534	-1.059539	-0.366717
C	-2.023462	0.604453	0.836868
N	-3.318630	0.836238	0.761857
N	-3.813021	-0.160238	-0.009485
C	-5.196026	-0.103760	-0.410198
H	-5.784759	0.256714	0.434014
H	-5.329503	0.578034	-1.255935
H	-5.533301	-1.102340	-0.692038
H	-3.110093	-1.914854	-0.987154
H	-1.377972	1.242331	1.423939
H	5.278732	0.834395	0.125126
O	0.752021	1.210020	-0.226454
C	0.835676	2.604743	-0.408503
H	1.268413	3.104130	0.467683
H	1.427360	2.860817	-1.296192
H	-0.191069	2.946454	-0.544963
H	2.862212	-2.726734	0.078160

32
4pzHOMe2-ts-B-m ; E = -834.104218701 Hartree

C	2.459090	-0.733016	0.127710
C	3.568621	-0.018317	0.563238
C	1.270513	-0.052180	-0.270493
H	4.463810	-0.539314	0.881869
C	3.547982	1.375200	0.551185
C	1.270740	1.371988	-0.220342
C	2.411815	2.073382	0.148460
H	2.415351	3.157180	0.152754
N	0.206996	-0.713830	-0.676045
N	-0.770610	-1.348672	-1.081453
C	-2.006848	-0.987923	-0.512836
C	-2.339528	0.039536	0.363052
C	-3.223054	-1.665368	-0.745561
N	-4.221664	-1.111791	-0.074144
N	-3.659115	-0.080444	0.587513
C	-4.497917	0.768606	1.396131
H	-3.868065	1.463799	1.952131
H	-5.192154	1.329077	0.764556
H	-5.069883	0.155036	2.094795
H	-1.731830	0.826205	0.783469
H	-3.389628	-2.532794	-1.369188
H	4.430710	1.924750	0.860814
O	0.093590	1.935832	-0.601243
C	0.044496	3.340519	-0.677961
H	0.231813	3.809408	0.297150
H	0.770822	3.726732	-1.403670
H	-0.963854	3.591063	-1.010333
O	2.363490	-2.080638	0.084137
C	3.495362	-2.822868	0.468257
H	4.364655	-2.590517	-0.160545
H	3.757039	-2.644437	1.519298
H	3.227693	-3.871932	0.337599

32
4pzHOMe2-ts-B-n ; E = -834.104481023 Hartree

C	1.515693	-1.336015	-0.138122
C	2.753201	-1.872702	0.195324
C	1.335822	0.073452	-0.248405
H	2.891580	-2.946262	0.248523
C	3.812427	-1.020815	0.500159
C	2.449771	0.913871	0.045101
C	3.661199	0.363519	0.447396
H	4.499987	1.005005	0.690732
N	0.176008	0.577406	-0.616268
N	-0.895542	1.057316	-0.996329
C	-2.043494	0.566003	-0.345091
C	-3.318485	1.055145	-0.584703
C	-2.226585	-0.443282	0.631676
N	-3.503110	-0.564340	0.958568
N	-4.147644	0.353740	0.209589
C	-5.570849	0.513908	0.369007
H	-6.051007	-0.464865	0.318498
H	-5.800276	0.973738	1.334294
H	-5.950175	1.147492	-0.433651
H	-3.661381	1.832506	-1.251198
H	-1.474577	-1.084696	1.067242
H	4.771098	-1.441970	0.784467

O	2.185805	2.236502	-0.052901
C	3.234475	3.128326	0.236515
H	3.567944	3.034657	1.278172
H	4.094033	2.973097	-0.428525
H	2.833143	4.129710	0.076448
O	0.402548	-2.054660	-0.430020
C	0.502802	-3.457892	-0.376562
H	1.225028	-3.835877	-1.111018
H	0.793756	-3.806391	0.622893
H	-0.489834	-3.840830	-0.616202

32

4pzHOMe2-ts-H-m ; E = -834.094683432 Hartree

C	2.769949	-0.535318	0.118666
C	3.749239	0.434845	0.337763
C	1.427206	-0.167790	-0.123758
H	4.775263	0.154147	0.539070
C	3.394158	1.777451	0.299676
C	1.101455	1.205634	-0.155867
C	2.087338	2.174406	0.052054
H	1.842340	3.228901	0.023507
N	0.549882	-1.252917	-0.435312
N	-0.667486	-1.042752	-0.314924
C	-1.965594	-0.895120	-0.217105
C	-2.664807	0.023681	0.596766
C	-3.025487	-1.604938	-0.880130
N	-4.213179	-1.166859	-0.517432
N	-3.970307	-0.206346	0.408038
C	-5.086842	0.360407	1.120704
H	-4.794760	1.321490	1.546960
H	-5.908179	0.505457	0.417901
H	-5.417589	-0.307332	1.922389
H	-2.289729	0.751815	1.299861
H	-2.927760	-2.374425	-1.634423
H	4.155574	2.533463	0.466888
O	-0.188886	1.520748	-0.420183
C	-0.502523	2.868436	-0.681645
H	-0.372045	3.498397	0.207821
H	0.106021	3.274100	-1.499146
H	-1.553580	2.875547	-0.973780
O	3.017635	-1.861069	0.140168
C	4.347306	-2.279040	0.332325
H	5.012914	-1.890749	-0.449253
H	4.732022	-1.973949	1.314173
H	4.328765	-3.368104	0.278866

32

4pzHOMe2-ts-H-n ; E = -834.094714688 Hartree

C	2.765649	-0.668167	0.015691
C	3.840820	0.215278	0.125221
C	1.441168	-0.187912	-0.095508
H	4.854444	-0.151256	0.225951
C	3.598344	1.582954	0.108764
C	1.230963	1.209136	-0.111100
C	2.311835	2.089893	-0.012035
H	2.155697	3.161299	-0.027100
N	0.450238	-1.198657	-0.293779
N	-0.731106	-0.880036	-0.084094
C	-1.998382	-0.612445	0.115567
C	-3.129005	-1.228893	-0.462992

C	-2.582307	0.394402	0.960279
N	-3.899136	0.378751	0.908173
N	-4.205515	-0.586081	0.008283
C	-5.581448	-0.751172	-0.385899
H	-5.721939	-1.749162	-0.803847
H	-6.209942	-0.632538	0.497453
H	-5.866984	-0.001269	-1.130347
H	-3.194702	-2.021971	-1.193278
H	-2.061320	1.067713	1.626792
H	4.433900	2.271634	0.191828
O	-0.045888	1.631123	-0.250480
C	-0.279927	3.009942	-0.417168
H	0.011429	3.582231	0.472655
H	0.252190	3.408180	-1.290073
H	-1.355465	3.109104	-0.568673
O	2.902377	-2.010381	0.026952
C	4.204285	-2.538289	0.101670
H	4.824446	-2.214195	-0.743999
H	4.703460	-2.259636	1.038812
H	4.089548	-3.622271	0.067021

36

4pzHPyr1A-ts-B-m ; E = -816.469106927 Hartree

C	-2.012417	0.740450	0.043542
C	-2.882837	1.836244	-0.096311
C	-0.609531	1.015670	0.253267
H	-3.944992	1.660531	-0.222657
C	-2.420083	3.143636	-0.036770
C	-0.171249	2.365715	0.213098
C	-1.061181	3.419836	0.124924
H	-0.706266	4.444668	0.166396
N	0.236348	0.059582	0.602798
N	1.029178	-0.816968	0.993231
C	2.359998	-0.671608	0.553570
C	2.942045	0.250641	-0.309200
C	3.416700	-1.541218	0.900692
N	4.552067	-1.187993	0.314482
N	4.237265	-0.104221	-0.417835
C	5.278198	0.572407	-1.150386
H	5.897191	1.174242	-0.478500
H	5.906136	-0.173147	-1.639999
H	4.824879	1.219613	-1.902510
H	2.521358	1.090950	-0.841084
H	3.376556	-2.404040	1.551089
H	-3.133060	3.957840	-0.128759
C	-2.660224	-2.751795	-0.776665
C	-1.656680	-1.726003	-0.254064
N	-2.475485	-0.538834	-0.016314
C	-3.895813	-0.828523	-0.128040
C	-3.956352	-2.349909	-0.079117
H	-2.774474	-2.649987	-1.861975
H	-1.155625	-2.075374	0.657563
H	-4.456522	-0.353152	0.685627
H	-4.854633	-2.746870	-0.558305
H	-0.870537	-1.502688	-0.982764
H	-2.350790	-3.778154	-0.564855
H	-3.946664	-2.690093	0.962198
H	-4.303208	-0.448391	-1.079030
H	0.891594	2.539839	0.351803

36
4pzHPyr1A-ts-B-n ; E = -816.469678615 Hartree

C	-2.140690	0.623652	0.051621
C	-3.140871	1.607563	-0.048365
C	-0.768603	1.064058	0.154976
H	-4.181536	1.307943	-0.095989
C	-2.829763	2.960141	-0.050324
C	-0.493861	2.453425	0.056253
C	-1.504290	3.395168	0.007705
H	-1.269002	4.454610	-0.000372
N	0.204893	0.220986	0.463098
N	1.118235	-0.545033	0.822235
C	2.396594	-0.239231	0.312776
C	3.540371	-0.955232	0.630991
C	2.841917	0.741179	-0.607604
N	4.144143	0.638726	-0.828720
N	4.547322	-0.397425	-0.070466
C	5.947945	-0.733627	-0.027944
H	6.061722	-1.750517	0.350604
H	6.354138	-0.672989	-1.038417
H	6.496209	-0.042341	0.619112
H	3.684218	-1.796749	1.292210
H	2.256281	1.492287	-1.119217
H	-3.637186	3.684357	-0.109153
C	-2.430982	-2.939809	-0.655577
C	-1.521295	-1.792148	-0.221805
N	-2.455157	-0.701493	0.050722
C	-3.836328	-1.154881	0.040683
C	-3.716563	-2.671409	0.121146
H	-2.627266	-2.880039	-1.732227
H	-0.923412	-2.057574	0.659325
H	-4.394444	-0.726256	0.881667
H	-4.592137	-3.180972	-0.288584
H	-0.816692	-1.498613	-1.006720
H	-1.991324	-3.918237	-0.446808
H	-3.599351	-2.981273	1.165425
H	-4.346311	-0.848687	-0.887230
H	0.548558	2.751625	0.111108

36
4pzHPyr1A-ts-H-m ; E = -816.463134887 Hartree

C	3.003385	-1.885121	-0.247781
C	4.050747	-1.490135	0.570338
C	1.852264	-1.109967	-0.389538
H	4.925032	-2.120809	0.694434
C	3.938403	-0.283265	1.256056
C	1.790338	0.181631	0.200113
C	2.835384	0.540622	1.070035
H	2.803011	1.493143	1.586384
N	0.789369	-1.816213	-1.055784
N	-0.358017	-1.500331	-0.691248
C	-1.596636	-1.252086	-0.328983
C	-2.011867	-0.421494	0.736422
C	-2.841684	-1.707672	-0.884064
N	-3.876866	-1.210948	-0.241137
N	-3.346875	-0.448730	0.751363
C	-4.244329	0.163524	1.698792
H	-5.047549	0.664929	1.156230
H	-4.678867	-0.593079	2.358014

H	-3.692752	0.892013	2.294317
H	-1.417237	0.128649	1.450298
H	-2.983264	-2.352665	-1.741368
H	4.730372	0.040544	1.925544
C	-0.083666	2.682452	-1.607706
C	0.279824	1.203381	-1.471024
N	0.762743	1.067484	-0.087791
C	0.709856	2.356086	0.586189
C	-0.380814	3.097425	-0.169298
H	0.771410	3.254608	-1.985009
H	1.055306	0.888023	-2.178761
H	0.501100	2.227310	1.654732
H	-1.367718	2.731715	0.136736
H	1.661452	2.908363	0.498053
H	-0.345656	4.177596	-0.007135
H	-0.921310	2.841234	-2.291349
H	-0.596804	0.572520	-1.639726
H	3.003765	-2.845221	-0.756525

36

4pzHPyr1A-ts-H-n ; E = -816.462052174 Hartree

C	-3.087697	-1.928716	0.259092
C	-4.292935	-1.409103	-0.186231
C	-1.917131	-1.167501	0.276034
H	-5.185062	-2.026256	-0.214901
C	-4.325840	-0.086762	-0.619074
C	-1.966186	0.210738	-0.070497
C	-3.187514	0.708034	-0.552719
H	-3.256668	1.746186	-0.856817
N	-0.767180	-1.972688	0.575494
N	0.336585	-1.518685	0.231543
C	1.530228	-1.113684	-0.132627
C	2.787960	-1.469980	0.400493
C	1.871093	-0.160818	-1.153302
N	3.173899	0.029765	-1.231394
N	3.703472	-0.739547	-0.251901
C	5.113686	-0.632469	0.025251
H	5.644928	-0.540587	-0.922621
H	5.324580	0.248491	0.639631
H	5.449947	-1.529020	0.548115
H	3.041075	-2.132004	1.215445
H	1.191397	0.319904	-1.842214
H	-5.251176	0.345262	-0.989605
C	0.858258	2.218509	1.189205
C	-0.229361	1.155369	1.379762
N	-0.856631	1.044425	0.062712
C	-0.915279	2.400407	-0.454679
C	0.459175	2.964722	-0.101163
H	0.905923	2.884066	2.055250
H	-0.976634	1.486279	2.120184
H	-1.120708	2.394083	-1.529635
H	1.173054	2.742942	-0.898443
H	-1.708151	2.993240	0.037873
H	0.428095	4.049822	0.025836
H	1.839668	1.752844	1.073198
H	0.166046	0.196589	1.714295
H	-2.988080	-2.967212	0.561973

36

4pzHPyr1B-ts-B-m ; E = -816.470599862 Hartree

C	-2.423743	-2.274327	-0.191243
C	-3.570452	-2.114826	0.570824
C	-1.510280	-1.204952	-0.351824
H	-4.226751	-2.963497	0.737988
C	-3.885549	-0.865845	1.098491
C	-1.858295	0.091316	0.153005
C	-3.048218	0.225813	0.868487
H	-3.314742	1.190597	1.285692
N	-0.314796	-1.421659	-0.879893
N	0.794847	-1.707261	-1.348134
C	1.885783	-1.234866	-0.589523
C	1.901072	-0.459203	0.566383
C	3.247018	-1.466829	-0.882757
N	4.037687	-0.886845	0.008083
N	3.197878	-0.279070	0.870025
C	3.748750	0.434203	1.995614
H	4.203007	-0.264033	2.703473
H	4.511792	1.131211	1.644384
H	2.948900	0.985231	2.491531
H	1.085556	-0.023350	1.125953
H	3.663208	-2.030368	-1.706335
H	-4.791914	-0.728464	1.679732
C	-0.237252	2.893082	-1.489949
C	-0.366241	1.372240	-1.381046
N	-0.971195	1.143710	-0.053432
C	-1.229767	2.422038	0.594000
C	-0.195315	3.342758	-0.031629
H	0.640561	3.193577	-2.067380
H	0.617895	0.898202	-1.445814
H	-2.242145	2.800416	0.370743
H	-0.432173	4.400446	0.107741
H	-1.143826	2.326067	1.682079
H	0.792970	3.149617	0.401328
H	-1.121448	3.317659	-1.978205
H	-0.995825	0.947292	-2.170992
H	-2.151094	-3.236335	-0.611457

36

4pzHPyr1B-ts-B-n ; E = -816.470394752 Hartree

C	2.277891	-2.414936	0.373558
C	3.474585	-2.429051	-0.325418
C	1.473659	-1.250455	0.408377
H	4.044248	-3.350264	-0.400284
C	3.947171	-1.257177	-0.909660
C	1.988139	-0.030788	-0.145047
C	3.222643	-0.071393	-0.796726
H	3.613173	0.829965	-1.255649
N	0.231189	-1.320719	0.865307
N	-0.932021	-1.474055	1.260674
C	-1.916971	-0.934161	0.405524
C	-3.280246	-1.032025	0.639234
C	-1.813590	-0.219550	-0.814368
N	-3.010619	0.098772	-1.282007
N	-3.884400	-0.402946	-0.386351
C	-5.297712	-0.279505	-0.641438
H	-5.516887	0.745562	-0.944188
H	-5.606879	-0.960668	-1.439269
H	-5.847346	-0.516068	0.270517
H	-3.827196	-1.495347	1.446870

H -0.910388 0.082617 -1.327008
H 4.891711 -1.253572 -1.444738
C 0.567276 3.002382 1.243715
C 0.512876 1.475016 1.192897
N 1.227725 1.123659 -0.046190
C 1.665351 2.330622 -0.732023
C 0.697258 3.388439 -0.227408
H -0.309961 3.431643 1.734326
H -0.526058 1.131647 1.157239
H 2.697037 2.607475 -0.454641
H 1.066786 4.405086 -0.382840
H 1.638934 2.187071 -1.817814
H -0.267842 3.288245 -0.736353
H 1.456319 3.334786 1.791493
H 0.991516 1.006784 2.060502
H 1.879723 -3.312458 0.834502
36
4pzHPyr1B-ts-H-m ; E = -816.466937787 Hartree
C 2.042122 0.742056 -0.051898
C 2.640288 2.025790 -0.015252
C 0.620730 0.701785 -0.136771
H 3.717882 2.108705 0.054133
C 1.886275 3.183969 -0.045767
C -0.114372 1.891483 -0.158252
C 0.491554 3.131600 -0.114159
H -0.098372 4.042022 -0.145526
N -0.007717 -0.570350 -0.291373
N -1.250750 -0.582407 -0.268222
C -2.564972 -0.627536 -0.236049
C -3.459659 0.247303 0.419378
C -3.446295 -1.594093 -0.831267
N -4.711852 -1.330819 -0.576093
N -4.694385 -0.235123 0.215678
C -5.930625 0.213763 0.804689
H -6.727984 0.074111 0.074180
H -6.166351 -0.361322 1.705724
H -5.849023 1.271536 1.060137
H -3.267653 1.109214 1.041835
H -3.171990 -2.422608 -1.470517
H 2.395211 4.143344 -0.011944
C 3.709075 -2.425821 0.704462
C 2.417107 -1.734926 0.278964
N 2.841204 -0.365676 -0.010114
C 4.291148 -0.252300 -0.057210
C 4.768385 -1.697780 -0.115864
H 3.888648 -2.265321 1.773890
H 1.974024 -2.204377 -0.606110
H 4.610902 0.333014 -0.927748
H 5.784238 -1.816317 0.269320
H 1.646665 -1.744915 1.053959
H 3.681445 -3.503545 0.524896
H 4.753525 -2.053925 -1.151920
H 4.681808 0.251818 0.842121
H -1.193046 1.797428 -0.245584
36
4pzHPyr1B-ts-H-n ; E = -816.466985713 Hartree
C 2.110047 0.678006 -0.084349
C 2.823574 1.901304 -0.111129

C	0.688588	0.767348	-0.068852
H	3.906427	1.885837	-0.121134
C	2.177281	3.123345	-0.104242
C	0.064429	2.018749	-0.053934
C	0.782360	3.198149	-0.070249
H	0.276308	4.158164	-0.069994
N	-0.060246	-0.444144	-0.163005
N	-1.295568	-0.345192	-0.061275
C	-2.602609	-0.268412	0.069757
C	-3.576580	-1.145421	-0.453512
C	-3.396686	0.702656	0.773188
N	-4.685564	0.437192	0.693243
N	-4.772490	-0.663068	-0.087062
C	-6.076357	-1.118924	-0.498278
H	-6.433942	-0.551739	-1.363408
H	-6.027333	-2.178608	-0.753850
H	-6.766905	-0.974092	0.333016
H	-3.464949	-2.015820	-1.083057
H	-3.043469	1.532270	1.371791
H	2.772113	4.032443	-0.121668
C	3.533081	-2.614645	0.629962
C	2.282044	-1.816904	0.274296
N	2.806012	-0.498778	-0.080303
C	4.253811	-0.518693	-0.223303
C	4.594443	-2.002398	-0.277703
H	3.799700	-2.450222	1.680449
H	1.737642	-2.262517	-0.565475
H	4.566553	0.019525	-1.126190
H	5.619659	-2.205772	0.041625
H	1.570099	-1.739881	1.099972
H	3.396730	-3.688764	0.480614
H	4.476385	-2.375405	-1.301173
H	4.747167	-0.036555	0.636804
H	-1.021746	2.024106	-0.059411

48

4pzHPyr2-ts-B-m ; E = -1027.64402182 Hartree

C	0.018016	1.708155	0.246514
C	0.472456	2.822309	0.950309
C	0.875322	0.554440	0.080208
H	-0.170515	3.676593	1.124471
C	1.782491	2.829122	1.425175
C	2.283638	0.678900	0.413052
C	2.678986	1.807496	1.162749
H	3.690269	1.887673	1.540309
N	0.369671	-0.613993	-0.247295
N	-0.086001	-1.773806	-0.395378
C	-1.430972	-1.950656	-0.013360
C	-2.263786	-1.151158	0.763717
C	-2.206642	-3.101404	-0.283264
N	-3.415538	-3.015171	0.254925
N	-3.427930	-1.824119	0.881093
C	-4.578842	-1.454727	1.664390
H	-5.475830	-1.793276	1.144481
H	-4.546532	-1.916050	2.656605
H	-4.607313	-0.368888	1.773454
H	-2.092151	-0.180084	1.202800
H	-1.923633	-3.969337	-0.862971
H	2.128904	3.689445	1.991947

C	5.317409	-1.172559	-0.405431
C	4.593368	-0.141076	0.457836
N	3.183141	-0.295650	0.115887
C	2.981042	-1.393315	-0.836118
C	4.246560	-2.223677	-0.671083
H	5.628599	-0.711505	-1.349420
H	4.943731	0.876115	0.242632
H	2.076359	-1.961780	-0.627572
H	4.140173	-2.886637	0.194961
C	-2.653651	1.828858	-2.192626
C	-1.481359	1.032713	-1.616396
N	-1.243885	1.644357	-0.294524
C	-2.200188	2.713672	-0.050664
C	-3.371202	2.342163	-0.946651
H	-2.289063	2.677792	-2.782007
H	-1.737978	-0.024560	-1.518990
H	-2.454734	2.767518	1.013787
H	-4.038159	3.186022	-1.139866
H	-0.576873	1.104706	-2.230459
H	-3.287485	1.217912	-2.839951
H	-3.955953	1.536982	-0.486931
H	-1.794943	3.695899	-0.346699
H	2.897918	-0.999360	-1.861285
H	4.452965	-2.843464	-1.547229
H	6.211779	-1.566767	0.083591
H	4.748371	-0.321804	1.531251

48

4pzHPyr2-ts-B-n ; E = -1027.64570221 Hartree

C	-2.388032	0.534910	0.365765
C	-2.968947	1.605319	1.071082
C	-0.963671	0.580266	0.092335
H	-4.012152	1.567669	1.361439
C	-2.216660	2.731492	1.363547
C	-0.264436	1.832292	0.280721
C	-0.891959	2.880231	0.956622
H	-0.367951	3.807596	1.154800
N	-0.321587	-0.535912	-0.174845
N	0.265391	-1.640693	-0.269657
C	1.613651	-1.646326	0.154018
C	2.490274	-2.699390	-0.060972
C	2.353987	-0.737728	0.953613
N	3.580494	-1.186071	1.189189
N	3.641578	-2.376807	0.567061
C	4.881732	-3.108593	0.566452
H	5.557956	-2.741303	-0.212384
H	5.358725	-2.982423	1.539026
H	4.677486	-4.167032	0.395721
H	2.363816	-3.623234	-0.605707
H	2.019141	0.207687	1.356004
H	-2.693909	3.552597	1.892286
C	2.525310	2.230244	-1.995418
C	1.416192	1.314529	-1.475373
N	1.013283	1.932282	-0.201091
C	1.833125	3.097652	0.090963
C	3.095808	2.840040	-0.717102
H	3.265898	1.685000	-2.585802
H	1.790479	0.299669	-1.322558
H	1.346706	4.029066	-0.244800

```
H        3.679444        3.748331       -0.886542
C       -4.081733       -2.175196       -1.365137
C       -2.751781       -1.501506       -1.026344
N       -3.111908       -0.566720        0.045936
C       -4.482292       -0.746246        0.488860
C       -4.845898       -2.125856       -0.044757
H       -4.618738       -1.595521       -2.125046
H       -1.996976       -2.220194       -0.692997
H       -4.548832       -0.676366        1.580627
H       -5.924717       -2.260688       -0.156319
H       -2.328127       -0.960867       -1.879476
H       -3.945047       -3.188619       -1.750741
H       -4.473190       -2.897787        0.637480
H       -5.147549        0.024955        0.065002
H        2.009541        3.182485        1.168790
H        3.727357        2.110498       -0.198144
H        2.105470        3.021241       -2.627436
H        0.556397        1.257867       -2.152619
48
4pzHPyr2-ts-H-m ; E = -1027.62970419 Hartree
C       -2.414245        0.478540       -0.466572
C       -2.796661        1.597562       -1.241128
C       -1.030468        0.365777       -0.112861
H       -3.805290        1.670529       -1.625085
C       -1.880366        2.577237       -1.574941
C       -0.157273        1.483032       -0.301714
C       -0.576690        2.555769       -1.097739
H        0.086886        3.384433       -1.308959
N       -0.598401       -0.939683        0.238016
N        0.610337       -1.196802        0.077661
C        1.857210       -1.565482       -0.100381
C        2.835718       -0.892330       -0.865038
C        2.580999       -2.692767        0.421699
N        3.838678       -2.696433        0.027415
N        3.962924       -1.614425       -0.777539
C        5.197330       -1.425719       -1.495558
H        6.020177       -1.736400       -0.850797
H        5.215373       -2.025696       -2.411083
H        5.311150       -0.370990       -1.752247
H        2.746078       -0.005354       -1.473726
H        2.215468       -3.452012        1.100658
H       -2.204216        3.409057       -2.194749
C        2.291451        1.964399        2.301818
C        1.234281        1.040911        1.697237
N        1.081283        1.511519        0.314434
C        1.994994        2.614060        0.056922
C        3.097241        2.400920        1.081302
H        1.820029        2.839646        2.763402
H        0.271934        1.086685        2.220261
H        2.344081        2.594987       -0.982112
H        3.762006        1.592373        0.756794
C       -4.583289       -2.317276        0.598152
C       -3.201407       -1.674099        0.628717
N       -3.372476       -0.447155       -0.148462
C       -4.783472       -0.152241       -0.372122
C       -5.523006       -1.120573        0.543697
H       -4.691247       -2.921857       -0.309755
H       -2.881044       -1.441468        1.655019
```

H	-5.007207	0.891559	-0.123726
H	-6.524013	-1.359953	0.175553
H	-2.424171	-2.302865	0.198574
H	-4.754577	-2.969969	1.458012
H	-5.626652	-0.678775	1.541160
H	-5.059870	-0.306606	-1.426968
H	1.514057	3.592572	0.226984
H	3.695831	3.300089	1.247901
H	2.890938	1.462656	3.065511
H	1.573766	0.001906	1.719754

48

4pzHPyr2-ts-H-n ; E = -1027.62897678 Hartree

C	2.486232	0.398153	0.397684
C	3.007886	1.530468	1.062935
C	1.075779	0.380219	0.137061
H	4.041752	1.543711	1.380643
C	2.196654	2.605702	1.376693
C	0.313595	1.583777	0.284202
C	0.868573	2.666331	0.977191
H	0.288629	3.560950	1.164502
N	0.518244	-0.909231	-0.033635
N	-0.706806	-1.045226	0.141520
C	-1.971197	-1.282277	0.407523
C	-2.797223	-2.332523	-0.044079
C	-2.869484	-0.488555	1.201168
N	-4.085937	-1.002996	1.233485
N	-4.028129	-2.095357	0.443231
C	-5.248015	-2.796930	0.137124
H	-5.746660	-2.361531	-0.735390
H	-5.028188	-3.847983	-0.059755
H	-5.908332	-2.717818	1.000978
H	-2.581375	-3.161501	-0.701754
H	-2.620058	0.389590	1.780590
H	2.626504	3.446388	1.914738
C	-2.289458	2.074914	-2.169937
C	-1.233803	1.132093	-1.592441
N	-0.949720	1.674844	-0.260283
C	-1.785645	2.836046	0.003556
C	-2.973795	2.623389	-0.920349
H	-1.813293	2.894375	-2.720673
H	-0.314672	1.095005	-2.189266
H	-2.053918	2.885698	1.064622
H	-3.648325	1.873067	-0.493684
C	4.341690	-2.622532	-0.644212
C	3.016833	-1.868895	-0.610204
N	3.341168	-0.625105	0.087280
C	4.783897	-0.440523	0.201603
C	5.376419	-1.507742	-0.711388
H	4.465806	-3.195518	0.281902
H	2.640695	-1.655977	-1.622385
H	5.073182	0.567839	-0.115106
H	6.377981	-1.814261	-0.398743
H	2.227215	-2.410324	-0.093348
H	4.397709	-3.322909	-1.481535
H	5.445662	-1.120610	-1.734227
H	5.119852	-0.568832	1.242334
H	-1.271584	3.777094	-0.257113
H	-3.537704	3.542780	-1.097110

H -2.971817 1.562485 -2.852962
H -1.624433 0.113084 -1.523226
30
4pzMe-ts-B-m ; E = -683.860614377 Hartree
C 2.672850 -0.314893 1.220558
C 3.954308 0.210544 1.196392
C 2.002065 -0.590815 0.000003
H 4.446029 0.410590 2.145001
C 4.615327 0.483596 -0.000010
C 2.672881 -0.315002 -1.220559
H 2.171426 -0.530261 -2.157817
C 3.954329 0.210456 -1.196406
H 4.446067 0.410434 -2.145020
H 2.171377 -0.530084 2.157822
N 0.771054 -1.067709 0.000007
N -0.365194 -1.532473 -0.000019
C -1.427397 -0.625285 0.000031
C -1.436927 0.775155 0.000033
C -2.788025 -1.024691 0.000018
N -3.575635 0.037093 0.000004
N -2.739538 1.107395 0.000023
C -3.318809 2.429867 -0.000105
H -4.399219 2.295844 -0.000507
H -3.022908 2.988372 0.892224
H -3.022262 2.988493 -0.892138
C -0.322276 1.756128 0.000043
H 0.321357 1.617087 0.874547
H 0.321122 1.617383 -0.874678
H -0.690305 2.784312 0.000278
C -3.338767 -2.406129 0.000023
H -3.001941 -2.961785 -0.879876
H -3.002005 -2.961755 0.879965
H -4.429404 -2.367288 -0.000013
H 5.618486 0.894927 -0.000017
30
4pzMe-ts-B-n ; E = -683.862607625 Hartree
C 2.788975 0.321153 -1.219554
C 4.106642 -0.105153 -1.194326
C 2.099504 0.545905 0.000084
H 4.612325 -0.269190 -2.142469
C 4.785632 -0.325464 0.002737
C 2.786817 0.321941 1.221089
H 2.268016 0.494025 2.157692
C 4.104526 -0.104396 1.198455
H 4.608533 -0.267827 2.147594
H 2.271827 0.492641 -2.157181
N 0.838072 0.938277 -0.001138
N -0.325123 1.326916 -0.001704
C -1.326175 0.352198 -0.001515
C -2.675187 0.699685 -0.003147
C -1.289533 -1.073187 -0.002008
N -2.522237 -1.550265 -0.003647
N -3.341466 -0.467441 -0.006407
C -4.768938 -0.664399 0.011443
H -5.202177 -0.322641 0.956647
H -5.243994 -0.127679 -0.814474
H -4.944696 -1.733145 -0.100710
C -3.316405 2.036588 -0.001330

H	-3.947377	2.179936	0.882606
H	-2.535831	2.799054	0.000800
H	-3.944834	2.183368	-0.886641
C	-0.111818	-1.981124	-0.003121
H	0.522406	-1.811691	-0.879139
H	0.522488	-1.813562	0.873192
H	-0.452634	-3.018203	-0.004233
H	5.816989	-0.659973	0.003755

30

4pzMe-ts-H-m ; E = -683.857597679 Hartree

C	-2.116417	-0.470672	-0.755591
C	-3.253437	-1.240696	-0.968823
C	-2.173989	0.632458	0.099757
H	-3.213980	-2.088850	-1.646883
C	-4.447886	-0.919513	-0.325277
C	-3.379683	0.979213	0.709039
H	-3.400580	1.862874	1.340114
C	-4.508115	0.192312	0.513635
H	-5.440156	0.449057	1.008736
H	-1.183891	-0.688775	-1.268535
N	-1.068699	1.519729	0.325970
N	0.064098	1.027337	0.177041
C	1.264786	0.507899	0.060772
C	1.662788	-0.850196	0.189820
C	2.500770	1.210925	-0.188162
N	3.518684	0.378797	-0.222084
N	2.993106	-0.852630	0.044311
C	3.892235	-1.967922	0.195193
H	3.605829	-2.791605	-0.464917
H	4.886019	-1.614123	-0.075950
H	3.902700	-2.326660	1.229474
C	0.863540	-2.046591	0.536003
H	1.048940	-2.375719	1.565425
H	-0.198659	-1.805662	0.451942
H	1.090516	-2.889276	-0.125887
C	2.641936	2.665753	-0.443406
H	3.665309	2.991398	-0.248470
H	2.395494	2.911280	-1.482149
H	1.946939	3.220760	0.193178
H	-5.333967	-1.525152	-0.490502

30

4pzMe-ts-H-n ; E = -683.857916569 Hartree

C	-2.222214	0.454656	0.754332
C	-3.426176	1.108382	0.985660
C	-2.191963	-0.676977	-0.063388
H	-3.453101	1.980703	1.632866
C	-4.601257	0.640485	0.398897
C	-3.374044	-1.170748	-0.615032
H	-3.322624	-2.072610	-1.218029
C	-4.572327	-0.500683	-0.401409
H	-5.488385	-0.871284	-0.852238
H	-1.300237	0.786800	1.222706
N	-1.006819	-1.448565	-0.304354
N	0.069786	-0.830843	-0.211155
C	1.214237	-0.188034	-0.172587
C	2.485718	-0.769287	0.067589
C	1.502424	1.217585	-0.355931
N	2.792938	1.447951	-0.232179

N	3.359722	0.242070	0.060734
C	4.764238	0.200076	0.377330
H	5.246532	-0.636914	-0.133658
H	5.200182	1.137519	0.033996
H	4.923245	0.100377	1.456171
C	2.809684	-2.184676	0.346537
H	3.091461	-2.349370	1.393070
H	1.919506	-2.782640	0.135143
H	3.633576	-2.541626	-0.280418
C	0.555307	2.290567	-0.750739
H	0.313677	2.223289	-1.817066
H	-0.390066	2.196967	-0.209210
H	0.995494	3.271158	-0.560762
H	-5.540414	1.155256	0.578547

52

4pzMeAmid2-ts-B-m ; E = -1478.12028087 Hartree

C	2.121389	1.655123	0.009619
C	2.352425	3.013553	0.212479
C	0.781794	1.166699	-0.127203
H	3.366387	3.363133	0.348452
C	1.269998	3.888679	0.205075
C	-0.299713	2.109606	-0.128905
C	-0.045267	3.469082	0.020113
H	-0.869750	4.168466	0.002771
N	0.541107	-0.131374	-0.248895
N	0.344139	-1.358220	-0.419171
C	-0.887749	-1.837137	0.064147
C	-1.616309	-1.462921	1.196153
C	-1.623393	-2.892471	-0.531823
N	-2.734230	-3.126000	0.153263
N	-2.696741	-2.274376	1.202663
C	-3.798388	-2.255055	2.130008
H	-3.432286	-2.175989	3.156228
H	-4.473229	-1.416881	1.922938
H	-4.340842	-3.192148	2.010621
C	-1.332269	-0.488012	2.281867
H	-1.371976	-0.968516	3.265607
H	-0.338336	-0.059706	2.151253
H	-2.049863	0.339442	2.284358
C	-1.294617	-3.639046	-1.775590
H	-1.428936	-3.010233	-2.661989
H	-0.249966	-3.962013	-1.765211
H	-1.942397	-4.512672	-1.869267
H	1.461143	4.949255	0.339302
N	3.105351	0.686976	-0.044781
H	2.760260	-0.241518	-0.266016
N	-1.554233	1.559947	-0.326280
H	-1.559771	0.548928	-0.416676
C	4.456258	0.841672	0.128004
O	5.018435	1.890814	0.404718
C	-2.771418	2.191388	-0.358904
O	-2.951012	3.393756	-0.240548
C	6.558667	-0.640894	0.045468
C	6.732189	-2.028963	-0.217475
C	5.486396	-2.528536	-0.448564
O	4.557355	-1.553262	-0.347864
C	5.219370	-0.401103	-0.045801
H	7.309668	0.100256	0.273517

```
H        7.658110       -2.584877       -0.233923
H        5.112571       -3.512315       -0.687114
C       -5.246285        1.484859       -0.571797
C       -5.851466        0.210578       -0.770009
C       -4.830181       -0.688413       -0.856254
O       -3.641882       -0.059147       -0.723303
C       -3.899779        1.267188       -0.548595
H       -5.724880        2.445913       -0.458163
H       -6.906248       -0.010789       -0.842829
H       -4.770499       -1.758956       -0.987559
52
4pzMeAmid2-ts-B-n ; E = -1478.11684741 Hartree
C       -2.104529       -1.713990        0.005353
C       -2.359900       -3.070608        0.174920
C       -0.757504       -1.241081       -0.131842
H       -3.379882       -3.402138        0.311346
C       -1.296734       -3.967700        0.119665
C        0.305261       -2.200997       -0.157457
C        0.019968       -3.561016       -0.068222
H        0.830190       -4.273980       -0.135805
N       -0.530477        0.057242       -0.281020
N       -0.286594        1.262931       -0.503547
C        0.886813        1.798395        0.046152
C        1.512238        2.909942       -0.514471
C        1.602617        1.520531        1.247012
N        2.600296        2.384321        1.387435
N        2.536370        3.201030        0.310467
C        3.547446        4.211519        0.135195
H        4.304598        3.896138       -0.591166
H        3.096189        5.145603       -0.207205
H        4.019601        4.371102        1.104055
C        1.188468        3.644960       -1.761357
H        1.940820        3.481054       -2.541020
H        0.227343        3.285270       -2.133671
H        1.115201        4.724029       -1.589013
C        1.307006        0.518872        2.309498
H        1.811663        0.812322        3.232054
H        0.231006        0.458472        2.496322
H        1.647396       -0.485161        2.040742
H       -1.504767       -5.028833        0.217730
N       -3.076734       -0.729736       -0.024306
H       -2.722570        0.194338       -0.247943
N        1.584692       -1.687671       -0.302396
H        1.653170       -0.679193       -0.221083
C       -4.426541       -0.866474        0.158191
O       -5.004344       -1.908186        0.432492
C        2.760733       -2.376477       -0.467270
O        2.855810       -3.575170       -0.682888
C       -6.509186        0.644791        0.106975
C       -6.668059        2.037399       -0.142220
C       -5.418631        2.523518       -0.380692
O       -4.500523        1.535709       -0.297340
C       -5.174167        0.389060        0.001001
H       -7.266857       -0.089192        0.335984
H       -7.587165        2.604794       -0.144762
H       -5.035110        3.504842       -0.613839
C        5.280209       -1.801982       -0.509085
C        5.975830       -0.603025       -0.180711
```

C	5.022890	0.306832	0.167715
O	3.793981	-0.241002	0.068558
C	3.954306	-1.529108	-0.341487
H	5.687880	-2.751403	-0.822002
H	7.043720	-0.440585	-0.190407
H	5.044113	1.324726	0.525489

52

4pzMeAmid2-ts-H-m ; E = -1478.11838419 Hartree

C	0.111351	-1.954017	-0.168188
C	-0.132107	-3.312097	0.053369
C	-0.963961	-1.037001	-0.132526
H	0.698627	-4.003579	0.039375
C	-1.433574	-3.742904	0.277383
C	-2.284646	-1.517903	0.027968
C	-2.518062	-2.874568	0.254565
H	-3.531171	-3.217231	0.410604
N	-0.879995	0.368494	-0.352176
N	0.155484	0.956245	0.021250
C	1.263350	1.587897	0.328491
C	2.251356	1.192288	1.272432
C	1.802199	2.785568	-0.267436
N	2.989109	3.060543	0.230278
N	3.212421	2.117255	1.192442
C	4.450717	2.162466	1.928384
H	4.272151	1.923534	2.979029
H	5.173138	1.452438	1.512063
H	4.846372	3.173931	1.843319
C	2.240586	0.073523	2.241812
H	2.160305	0.432665	3.274545
H	1.378228	-0.565161	2.039409
H	3.148922	-0.533394	2.166046
C	1.187915	3.575726	-1.362963
H	1.290827	3.062888	-2.325657
H	0.116956	3.701960	-1.179915
H	1.665179	4.554111	-1.440880
H	-1.611058	-4.799366	0.454867
N	1.384709	-1.478455	-0.497393
H	1.434282	-0.517889	-0.801008
N	-3.288902	-0.557479	-0.027381
H	-2.946325	0.371546	-0.256331
C	2.589932	-2.109207	-0.355315
O	2.766919	-3.238922	0.076579
C	-4.633231	-0.733103	0.137371
O	-5.191621	-1.789280	0.400822
C	5.078228	-1.472461	-0.567146
C	5.722374	-0.282717	-1.016801
C	4.728544	0.556764	-1.422574
O	3.522302	-0.029482	-1.258944
C	3.740112	-1.266932	-0.730824
H	5.525375	-2.369899	-0.166873
H	6.782853	-0.078365	-1.042417
H	4.711008	1.561894	-1.814976
C	-6.759133	0.715190	0.065361
C	-6.959576	2.102398	-0.184579
C	-5.723541	2.627763	-0.408702
O	-4.775598	1.669402	-0.315703
C	-5.415685	0.501888	-0.025632
H	-7.495269	-0.043065	0.285227

H	-7.896038	2.640574	-0.197135
H	-5.369049	3.621082	-0.637179

52

4pzMeAmid2-ts-H-n ; E = -1478.11386239 Hartree

C	0.108413	-2.035276	-0.112141
C	-0.173008	-3.389137	0.089609
C	-0.946051	-1.094237	-0.100881
H	0.642563	-4.098589	0.097454
C	-1.490322	-3.791574	0.268158
C	-2.281942	-1.545681	0.013316
C	-2.553656	-2.898389	0.218516
H	-3.578721	-3.218981	0.339010
N	-0.824189	0.311334	-0.295388
N	0.215289	0.870633	0.110830
C	1.297690	1.492237	0.514659
C	1.812278	2.714574	0.009755
C	2.266714	1.081357	1.508980
N	3.246591	1.956412	1.584377
N	2.971474	2.907704	0.646369
C	3.968680	3.917432	0.402525
H	3.508529	4.788262	-0.066052
H	4.407815	4.202813	1.358833
H	4.760545	3.530303	-0.247503
C	1.250794	3.570223	-1.058595
H	0.242074	3.213690	-1.281591
H	1.190621	4.618795	-0.749124
H	1.838276	3.525951	-1.982870
C	2.170109	-0.082925	2.428270
H	1.961502	0.245446	3.451325
H	1.361860	-0.745957	2.113454
H	3.107382	-0.645123	2.442899
H	-1.697316	-4.844927	0.431662
N	1.398190	-1.586407	-0.404190
H	1.477477	-0.620623	-0.683357
N	-3.262483	-0.561646	-0.062505
H	-2.891124	0.362494	-0.263811
C	2.584748	-2.250007	-0.255031
O	2.726877	-3.386699	0.170613
C	-4.614851	-0.708235	0.053341
O	-5.207163	-1.754451	0.279844
C	5.088371	-1.697681	-0.503185
C	5.767035	-0.551177	-1.006896
C	4.796928	0.313025	-1.412475
O	3.571114	-0.213697	-1.198194
C	3.756121	-1.443158	-0.638537
H	5.509923	-2.600466	-0.087851
H	6.833605	-0.389696	-1.061262
H	4.816071	1.291797	-1.866581
C	-6.704217	0.788735	-0.065152
C	-6.866571	2.183845	-0.298631
C	-5.612941	2.685250	-0.473766
O	-4.689118	1.704825	-0.365893
C	-5.363530	0.547224	-0.115886
H	-7.463125	0.043438	0.118557
H	-7.790369	2.742622	-0.332447
H	-5.230134	3.674164	-0.673874

30

4pzMeCl1A-ts-B-m ; E = -1143.30051289 Hartree

C	-2.526970	-0.089443	-0.648197
C	-3.797384	0.425153	-0.462605
C	-1.742581	-0.537425	0.447543
H	-4.355732	0.745415	-1.336528
C	-4.339646	0.538265	0.814883
C	-2.309782	-0.413168	1.744361
H	-1.728308	-0.749458	2.595449
C	-3.577606	0.118036	1.904165
H	-3.979622	0.198316	2.910310
Cl	-1.835371	-0.201767	-2.243638
N	-0.534542	-1.024791	0.267597
N	0.598767	-1.494430	0.214264
C	1.666600	-0.605121	0.172861
C	1.698994	0.796715	0.149872
C	3.020981	-1.027669	0.127683
N	3.823980	0.020079	0.085565
N	3.004612	1.105225	0.098538
C	3.605634	2.417093	0.040521
H	3.312918	2.942778	-0.872561
H	3.323163	3.015646	0.910613
H	4.683637	2.265293	0.040216
C	0.601811	1.796546	0.152644
H	-0.054613	1.644000	-0.710159
H	-0.023597	1.690886	1.044241
H	0.987382	2.817576	0.118598
C	3.547107	-2.418249	0.129149
H	4.634638	-2.400386	0.040994
H	3.274366	-2.939631	1.051399
H	3.128961	-2.993152	-0.701843
H	-5.334557	0.945770	0.952080

30

4pzMeCl1A-ts-B-n ; E = -1143.30224341 Hartree

C	-2.620841	-0.672404	-0.024855
C	-3.929345	-0.427053	-0.400267
C	-1.827235	0.333750	0.586138
H	-4.492970	-1.233827	-0.857916
C	-4.502942	0.826516	-0.204894
C	-2.426376	1.606757	0.777853
H	-1.836671	2.389723	1.241143
C	-3.733099	1.830267	0.380883
H	-4.158874	2.816948	0.540567
Cl	-1.894246	-2.232179	-0.294794
N	-0.583393	0.096199	0.942484
N	0.576911	-0.055799	1.309933
C	1.581715	0.111598	0.363591
C	2.925786	-0.043286	0.704960
C	1.564631	0.433081	-1.026571
N	2.801419	0.472662	-1.487431
N	3.604857	0.183476	-0.431020
C	5.029843	0.163452	-0.643281
H	5.528281	-0.132876	0.279681
H	5.272892	-0.550083	-1.433520
H	5.377401	1.155548	-0.941711
C	3.526215	-0.384764	2.018130
H	4.185091	0.410275	2.382722
H	2.718886	-0.522466	2.739400
H	4.107393	-1.311465	1.968550
C	0.401001	0.686834	-1.916025

H -0.260341 -0.184739 -1.952659
H -0.202184 1.526150 -1.556202
H 0.756180 0.908214 -2.924140
H -5.527573 1.011379 -0.506150
30
4pzMeCl1A-ts-H-m ; E = -1143.29239648 Hartree
C 2.197202 -0.299728 0.598727
C 3.371216 -1.045137 0.524926
C 1.998588 0.808848 -0.231926
H 3.503715 -1.890084 1.192704
C 4.359903 -0.692932 -0.386027
C 3.026491 1.174442 -1.103433
H 2.869344 2.063128 -1.708045
C 4.188337 0.423547 -1.202369
H 4.963162 0.711208 -1.906268
Cl 1.023026 -0.742043 1.809828
N 0.860991 1.692105 -0.202819
N -0.251290 1.138254 -0.173391
C -1.438921 0.586814 -0.167914
C -1.773473 -0.762168 -0.468087
C -2.708394 1.204342 0.134059
N -3.688698 0.335121 0.036990
N -3.099914 -0.839145 -0.351494
C -3.947683 -1.966595 -0.645100
H -4.036003 -2.122469 -1.725314
H -3.550386 -2.873984 -0.183677
H -4.930872 -1.745881 -0.231420
C -0.904154 -1.870898 -0.919173
H 0.134905 -1.537011 -0.892464
H -1.002176 -2.748395 -0.271630
H -1.140963 -2.180143 -1.943681
C -2.922136 2.609548 0.559364
H -2.652319 2.743238 1.612369
H -2.281896 3.277498 -0.023611
H -3.968096 2.893753 0.432023
H 5.270674 -1.280963 -0.441333
30
4pzMeCl1A-ts-H-n ; E = -1143.29188391 Hartree
C 2.282253 -0.316769 0.601080
C 3.522443 -0.939973 0.492330
C 2.009424 0.861538 -0.101449
H 3.710455 -1.845929 1.059054
C 4.503606 -0.392868 -0.326058
C 3.026033 1.421917 -0.876502
H 2.806991 2.359223 -1.379979
C 4.256050 0.794939 -1.011291
H 5.023545 1.233685 -1.641401
Cl 1.114825 -1.002693 1.700199
N 0.790550 1.624145 -0.014854
N -0.258426 0.966199 -0.123737
C -1.389922 0.323384 -0.271192
C -2.680851 0.789230 0.090866
C -1.630304 -0.995096 -0.812762
N -2.912610 -1.284282 -0.780084
N -3.522022 -0.203287 -0.203013
C -4.931157 -0.287935 0.084052
H -5.103931 -0.609353 1.116445
H -5.406986 0.682137 -0.074221

```
H       -5.360533       -1.022805       -0.596250
C       -3.046888        2.073184        0.726305
H       -2.161260        2.713369        0.729335
H       -3.848818        2.581394        0.180484
H       -3.378112        1.941130        1.762487
C       -0.631773       -1.902795       -1.430782
H       -0.384493       -1.574140       -2.446048
H        0.295007       -1.898898       -0.851864
H       -1.026184       -2.918919       -1.486349
H        5.466367       -0.887076       -0.410843
30
4pzMeCl1B-ts-B-m ; E = -1143.30051303 Hartree
C        2.306295       -0.414338        1.744410
C        3.574124        0.115934        1.907305
C        1.741938       -0.537531        0.446250
H        3.974078        0.195119        2.914359
C        4.338553        0.536908        0.820001
C        2.528697       -0.088869       -0.647507
Cl        1.840409       -0.199074       -2.243903
C        3.798808        0.425346       -0.458690
H        4.359040        0.746361       -1.331133
H        1.722826       -0.750959        2.594000
N        0.534171       -1.024536        0.263391
N       -0.599006       -1.494446        0.209543
C       -1.667104       -0.605334        0.170937
C       -1.699689        0.796515        0.148857
C       -3.021476       -1.028027        0.126845
N       -3.824646        0.019656        0.086340
N       -3.005390        1.104893        0.098969
C       -3.606629        2.416745        0.042790
H       -4.684577        2.264616        0.038872
H       -3.326939        3.013070        0.915333
H       -3.311347        2.944949       -0.867986
C       -0.602593        1.796409        0.150512
H       -0.988287        2.817429        0.117496
H        0.024144        1.690425        1.041134
H        0.052588        1.644244       -0.713318
C       -3.547411       -2.418663        0.128481
H       -3.123809       -2.995582       -0.698296
H       -3.280607       -2.937640        1.053858
H       -4.634381       -2.401215        0.033557
H        5.333337        0.943895        0.959627
30
4pzMeCl1B-ts-B-n ; E = -1143.30233100 Hartree
C        2.441702       -1.613524       -0.756322
C        3.749323       -1.820289       -0.353369
C        1.831195       -0.343342       -0.583286
H        4.183797       -2.805488       -0.497987
C        4.509498       -0.801556        0.219047
C        2.614945        0.678448        0.014385
Cl        1.874901        2.235792        0.260190
C        3.925032        0.449832        0.394962
H        4.481047        1.268031        0.841500
H        1.859642       -2.407975       -1.209593
N        0.585892       -0.121756       -0.945009
N       -0.574817        0.023109       -1.313577
C       -1.578435       -0.126884       -0.362190
C       -2.922660        0.019668       -0.704128
```

C -1.559629 -0.427578 1.032796
N -2.796047 -0.462498 1.495563
N -3.601464 -0.196293 0.433616
C -5.025644 -0.115065 0.639299
H -5.358632 0.926380 0.694312
H -5.555196 -0.618699 -0.172800
H -5.245731 -0.612501 1.582824
C -3.541153 0.344834 -2.011751
H -2.748732 0.472649 -2.750884
H -4.209129 -0.454181 -2.351735
H -4.124773 1.270188 -1.961617
C -0.395550 -0.672883 1.924400
H 0.207584 -1.515707 1.572878
H 0.265657 0.199048 1.953325
H -0.750645 -0.884826 2.934602
H 5.535101 -0.973271 0.524742
30
4pzMeCl1B-ts-H-m ; E = -1143.29336567 Hartree
C 1.485018 -1.149017 0.951699
C 2.463617 -2.126387 1.071157
C 1.753437 0.065136 0.314805
H 2.242950 -3.057848 1.583939
C 3.732987 -1.900168 0.543552
C 3.039940 0.286178 -0.185827
Cl 3.417376 1.782849 -0.979889
C 4.021156 -0.694084 -0.088053
H 5.003250 -0.504888 -0.508082
H 0.494651 -1.287831 1.377194
N 0.801239 1.136736 0.274240
N -0.392925 0.795213 0.223308
C -1.654449 0.446948 0.124597
C -2.204121 -0.780094 -0.339059
C -2.811838 1.255766 0.427414
N -3.922960 0.595356 0.190199
N -3.529492 -0.619405 -0.299761
C -4.541982 -1.538776 -0.753970
H -4.436666 -2.508766 -0.259928
H -5.506083 -1.102420 -0.496541
H -4.483555 -1.681179 -1.837621
C -1.526815 -1.983139 -0.870792
H -0.458149 -1.918982 -0.653470
H -1.925980 -2.901180 -0.426515
H -1.641425 -2.064470 -1.958089
C -2.795821 2.630081 0.985907
H -2.625027 2.613120 2.067717
H -1.977269 3.201183 0.538980
H -3.746269 3.131939 0.796538
H 4.506361 -2.657069 0.629501
30
4pzMeCl1B-ts-H-n ; E = -1143.29395053 Hartree
C -1.624649 1.206748 0.852069
C -2.696446 2.082053 0.958113
C -1.789574 -0.071625 0.314630
H -2.553411 3.067586 1.391028
C -3.957629 1.685235 0.518928
C -3.066985 -0.463660 -0.096224
Cl -3.317368 -2.047498 -0.763752
C -4.143446 0.411969 -0.011052

```
H        -5.118256         0.089850        -0.361297
H        -0.635627         1.478435         1.210649
N        -0.728907        -1.035789         0.289894
N         0.415968        -0.568176         0.156648
C         1.628556        -0.106340        -0.038347
C         2.836180        -0.787246         0.268560
C         2.069756         1.152051        -0.599607
N         3.383459         1.217093        -0.618898
N         3.822307         0.048355        -0.062132
C         5.236816        -0.116458         0.156923
H         5.753220         0.560940        -0.522377
H         5.507380         0.131610         1.188511
H         5.534167        -1.145934        -0.054026
C         3.003171        -2.121561         0.882906
H         3.685960        -2.750920         0.302476
H         3.391926        -2.061006         1.905748
H         2.022409        -2.602587         0.916251
C         1.228639         2.223171        -1.190029
H         0.826299         1.909864        -2.159265
H         0.368617         2.443639        -0.551466
H         1.819998         3.128397        -1.337913
H        -4.803576         2.361189         0.595659
30
4pzMeCl2-ts-B-m ; E = -1602.73869229 Hartree
C        -2.291672         1.206109        -0.094275
C        -3.567756         1.202224         0.437661
C        -1.605234        -0.001300        -0.401671
H        -4.042379         2.155729         0.644143
C        -4.222397         0.002979         0.706169
C        -2.292744        -1.206329        -0.087474
Cl        -1.458158        -2.703585        -0.380894
C        -3.568805        -1.198345         0.444421
H        -4.044258        -2.150256         0.656277
Cl        -1.455880         2.700819        -0.396067
N        -0.400834        -0.003298        -0.915574
N         0.722051        -0.004560        -1.411185
C         1.810514        -0.002207        -0.555422
C         1.880873         0.003440         0.846240
C         3.154729        -0.004588        -1.014524
N         3.984794        -0.000895         0.011630
N         3.193634         0.003849         1.119330
C         3.831541         0.009434         2.415281
H         3.558718         0.903964         2.981494
H         3.560309        -0.881142         2.988465
H         4.904806         0.009684         2.233274
C         0.808978         0.007800         1.872243
H         0.165810         0.885388         1.752645
H         0.168331        -0.872988         1.762781
H         1.219270         0.014229         2.884060
C         3.642351        -0.010883        -2.418610
H         3.283092        -0.895687        -2.951838
H         3.275852         0.864519        -2.962242
H         4.733449        -0.006679        -2.429398
H        -5.224122         0.004579         1.119051
30
4pzMeCl2-ts-B-n ; E = -1602.74057169 Hartree
C        -2.373117         1.214048        -0.126854
C        -3.685897         1.219032         0.306855
```

C	-1.674505	0.001646	-0.378864
H	-4.167771	2.175840	0.478075
C	-4.367709	0.024479	0.522661
C	-2.393792	-1.198646	-0.127531
Cl	-1.555257	-2.701471	-0.370961
C	-3.706384	-1.181476	0.306295
H	-4.204522	-2.129989	0.477115
Cl	-1.509199	2.703032	-0.369418
N	-0.433279	-0.008982	-0.797940
N	0.721674	-0.009470	-1.209471
C	1.743025	-0.009834	-0.273781
C	3.082237	-0.012772	-0.668440
C	1.754452	-0.013081	1.154051
N	3.001136	-0.017318	1.586325
N	3.784457	-0.022354	0.473948
C	5.216521	0.025943	0.631050
H	5.448864	-0.345442	1.628274
H	5.697986	-0.606925	-0.117405
H	5.590235	1.050072	0.531800
C	3.670213	-0.005427	-2.029255
H	4.292389	0.880542	-2.193995
H	4.292608	-0.889953	-2.202809
H	2.859992	-0.003116	-2.760017
C	0.607511	-0.018167	2.099081
H	-0.025902	0.862721	1.954049
H	-0.025695	-0.897703	1.945037
H	0.981567	-0.023385	3.124461
H	-5.397248	0.033203	0.860235

30

4pzMeCl2-ts-H-m ; E = -1602.72751287 Hartree

C	1.745773	-1.145446	0.661189
C	2.818605	-1.973711	0.346842
C	1.729282	0.206013	0.299789
H	2.798516	-3.012215	0.658700
C	3.901373	-1.454015	-0.350962
C	2.846064	0.699126	-0.382484
Cl	2.871643	2.370215	-0.855058
C	3.919119	-0.113815	-0.722296
H	4.754118	0.306127	-1.271990
Cl	0.428387	-1.826730	1.577433
N	0.711024	1.142817	0.710205
N	-0.454034	0.824315	0.427086
C	-1.693733	0.526368	0.131381
C	-2.155163	-0.514971	-0.720473
C	-2.902871	1.170180	0.587740
N	-3.963950	0.587481	0.079546
N	-3.483412	-0.420752	-0.717275
C	-4.428104	-1.190227	-1.487106
H	-5.402885	-1.075136	-1.014529
H	-4.482568	-0.827802	-2.518813
H	-4.142715	-2.244425	-1.490918
C	-1.376564	-1.487140	-1.518169
H	-0.314395	-1.334057	-1.316993
H	-1.631579	-2.519361	-1.256017
H	-1.547992	-1.358809	-2.592578
C	-2.991386	2.308942	1.534345
H	-2.755308	1.983841	2.552968
H	-2.261772	3.078476	1.267173

H -3.996135 2.734448 1.527652
H 4.740452 -2.095189 -0.600726
30
4pzMeCl2-ts-H-n ; E = -1602.72737419 Hartree
C -1.901144 -1.168221 -0.542617
C -3.068908 -1.830365 -0.179288
C -1.749152 0.208584 -0.355638
H -3.151921 -2.897797 -0.351745
C -4.112115 -1.111592 0.391146
C -2.827145 0.903271 0.198763
Cl -2.683006 2.616747 0.450373
C -3.995863 0.260397 0.585252
H -4.798835 0.835042 1.033297
Cl -0.633504 -2.095184 -1.300058
N -0.610911 0.962956 -0.826949
N 0.487159 0.579059 -0.394494
C 1.669343 0.234536 0.050250
C 2.923269 0.709769 -0.418329
C 2.013921 -0.687331 1.108489
N 3.316884 -0.756479 1.258581
N 3.844184 0.086770 0.313859
C 5.277968 0.143082 0.182464
H 5.711451 -0.033107 1.166845
H 5.637321 -0.625282 -0.509474
H 5.579173 1.126409 -0.182537
C 3.185281 1.669537 -1.512165
H 2.223588 2.051454 -1.863149
H 3.795946 2.513452 -1.174121
H 3.700055 1.197996 -2.356234
C 1.077273 -1.438181 1.981034
H 0.473064 -0.750215 2.580873
H 0.385994 -2.032253 1.376702
H 1.632069 -2.096650 2.651241
H -5.024799 -1.622683 0.679823
30
4pzMeF1A-ts-B-m ; E = -783.003123547 Hartree
C 2.518209 0.196039 0.877011
C 3.793923 0.676288 0.703130
C 1.874555 -0.625054 -0.080112
H 4.219029 1.294560 1.487499
C 4.498445 0.377701 -0.465927
C 2.604042 -0.920975 -1.257798
H 2.137992 -1.544102 -2.012554
C 3.882716 -0.409767 -1.434288
H 4.410861 -0.649920 -2.352647
F 1.807078 0.511872 1.983369
N 0.651503 -1.067023 0.138857
N -0.489148 -1.502347 0.277909
C -1.550432 -0.626898 0.059997
C -1.573666 0.755337 -0.169487
C -2.907320 -1.042540 0.071758
N -3.703131 -0.009915 -0.142753
N -2.877118 1.060408 -0.281115
C -3.469449 2.360438 -0.490320
H -3.288286 3.018297 0.364650
H -3.075476 2.827015 -1.396778
H -4.540644 2.200489 -0.600824
C -0.471836 1.748048 -0.233139

H	0.169683	1.649065	0.647008
H	0.163415	1.579007	-1.108267
H	-0.852546	2.770786	-0.274534
C	-3.441932	-2.415911	0.269248
H	-3.033566	-2.863909	1.179128
H	-4.530333	-2.381260	0.340930
H	-3.165212	-3.068843	-0.564194
H	5.503110	0.757857	-0.610463

30

4pzMeF1A-ts-B-n ; E = -783.004922900 Hartree

C	-2.608376	-0.276088	0.854214
C	-3.919580	-0.658509	0.701972
C	-1.964776	0.608898	-0.044528
H	-4.343998	-1.333706	1.438257
C	-4.659193	-0.196480	-0.389706
C	-2.730970	1.074662	-1.140021
H	-2.263977	1.748861	-1.848923
C	-4.044569	0.655424	-1.302381
H	-4.600527	1.021482	-2.160752
F	-1.868303	-0.745308	1.881462
N	-0.706751	0.952975	0.153335
N	0.460266	1.287846	0.336668
C	1.456562	0.360753	0.037518
C	2.805867	0.694611	0.147305
C	1.424751	-1.002812	-0.380785
N	2.658282	-1.456061	-0.515182
N	3.474874	-0.419078	-0.193231
C	4.901576	-0.594077	-0.294851
H	5.094353	-1.665464	-0.328265
H	5.294541	-0.128006	-1.204282
H	5.400398	-0.159526	0.574731
C	3.438993	1.975897	0.542277
H	4.047092	2.393206	-0.267638
H	2.653049	2.690744	0.791196
H	4.086917	1.851668	1.416766
C	0.249341	-1.883087	-0.612520
H	-0.376998	-1.931701	0.283003
H	-0.381195	-1.504163	-1.422681
H	0.591502	-2.887724	-0.867845
H	-5.690893	-0.502822	-0.518826

30

4pzMeF1A-ts-H-m ; E = -782.998860638 Hartree

C	2.142306	-0.435787	0.641703
C	3.296198	-1.196140	0.742203
C	2.084148	0.720483	-0.143211
H	3.288574	-2.077875	1.374712
C	4.434115	-0.802452	0.043916
C	3.251218	1.116848	-0.798516
H	3.206081	2.037855	-1.372703
C	4.411784	0.357587	-0.728281
H	5.300687	0.672660	-1.265586
F	1.066267	-0.819169	1.350855
N	0.960404	1.608668	-0.240147
N	-0.163146	1.075837	-0.179034
C	-1.362131	0.554694	-0.111175
C	-1.740555	-0.803330	-0.297965
C	-2.609729	1.230069	0.159525
N	-3.615724	0.384687	0.151000

```
N       -3.067602       -0.831449       -0.154428
C       -3.949787       -1.954690       -0.343166
H       -3.597243       -2.820408        0.223483
H       -4.931759       -1.655054        0.020639
H       -4.022463       -2.225085       -1.401790
C       -0.922766       -1.977103       -0.677688
H        0.116395       -1.666461       -0.786901
H       -0.968879       -2.761319        0.085694
H       -1.255689       -2.412747       -1.626429
C       -2.775407        2.670878        0.472658
H       -2.504656        2.879244        1.513345
H       -2.110510        3.267298       -0.158359
H       -3.810505        2.980438        0.317918
H        5.339683       -1.396576        0.116537
30
4pzMeF1A-ts-H-n ; E = -782.999191446 Hartree
C        2.284007       -0.401298        0.664476
C        3.510815       -1.042876        0.706158
C        2.088636        0.774887       -0.063992
H        3.608800       -1.948922        1.295219
C        4.585114       -0.502425        0.005087
C        3.192229        1.321773       -0.719850
H        3.040364        2.258432       -1.248689
C        4.425809        0.683509       -0.709087
H        5.264591        1.113268       -1.247541
F        1.270580       -0.919741        1.380611
N        0.873134        1.540195       -0.095214
N       -0.183643        0.880838       -0.109188
C       -1.321672        0.233486       -0.160217
C       -2.610309        0.785370        0.067439
C       -1.586709       -1.155493       -0.466756
N       -2.877263       -1.402713       -0.428123
N       -3.472904       -0.220536       -0.078531
C       -4.894597       -0.215013        0.152651
H       -5.329945        0.722698       -0.199189
H       -5.320513       -1.049140       -0.404279
H       -5.121637       -0.339955        1.216576
C       -2.957095        2.173574        0.439555
H       -2.047536        2.776008        0.375751
H       -3.710146        2.597901       -0.233033
H       -3.344984        2.241758        1.462119
C       -0.606743       -2.202161       -0.853990
H       -0.084036       -1.922920       -1.774297
H        0.148872       -2.320164       -0.074539
H       -1.118916       -3.152218       -1.014871
H        5.547696       -1.003564        0.029827
30
4pzMeF1B-ts-B-m ; E = -783.003123653 Hartree
C       -2.604040       -0.921906       -1.257202
C       -3.882693       -0.410816       -1.434168
C       -1.874587       -0.625023       -0.079736
H       -4.410789       -0.651671       -2.352371
C       -4.498478        0.377381       -0.466433
C       -2.518310        0.196748        0.876761
F       -1.807348        0.513317        1.982902
C       -3.794034        0.676827        0.702447
H       -4.219210        1.295644        1.486349
H       -2.137946       -1.545609       -2.011454
```

N -0.651541 -1.066832 0.139614
N 0.489115 -1.502079 0.278881
C 1.550428 -0.626805 0.060446
C 1.573796 0.755325 -0.169683
C 2.907263 -1.042622 0.072095
N 3.703172 -0.010203 -0.143034
N 2.877266 1.060138 -0.281844
C 3.469818 2.360099 -0.490837
H 4.540259 2.199417 -0.607436
H 3.071462 2.829254 -1.394005
H 3.293873 3.016037 0.366725
C 0.472096 1.748185 -0.233144
H -0.162800 1.579881 -1.108678
H -0.169787 1.648613 0.646659
H 0.852913 2.770924 -0.273529
C 3.441622 -2.416100 0.269617
H 3.029075 -2.865934 1.176673
H 3.169199 -3.067602 -0.566386
H 4.529668 -2.381151 0.346362
H -5.503138 0.757423 -0.611311
30
4pzMeF1B-ts-B-n ; E = -783.004922906 Hartree
C 2.730967 1.074665 -1.139990
C 4.044569 0.655436 -1.302351
C 1.964776 0.608892 -0.044499
H 4.600520 1.021493 -2.160727
C 4.659204 -0.196457 -0.389673
C 2.608391 -0.276069 0.854255
F 1.868328 -0.745269 1.881528
C 3.919600 -0.658478 0.702014
H 4.344027 -1.333656 1.438310
H 2.263968 1.748857 -1.848894
N 0.706749 0.952964 0.153357
N -0.460260 1.287838 0.336727
C -1.456550 0.360747 0.037522
C -2.805860 0.694595 0.147159
C -1.424689 -1.002790 -0.380864
N -2.658205 -1.456033 -0.515443
N -3.474836 -0.419024 -0.193634
C -4.901602 -0.594092 -0.294340
H -5.399584 -0.167238 0.579582
H -5.296301 -0.120776 -1.199252
H -5.093438 -1.665330 -0.336336
C -3.439245 1.975732 0.542195
H -4.087404 1.851231 1.416473
H -2.653510 2.690707 0.791397
H -4.047247 2.393021 -0.267813
C -0.249237 -1.883010 -0.612594
H 0.381129 -1.504206 -1.422944
H 0.377245 -1.931384 0.282842
H -0.591345 -2.887731 -0.867663
H 5.690905 -0.502792 -0.518795
30
4pzMeF1B-ts-H-m ; E = -782.998764796 Hartree
C 1.817541 -0.791558 0.875763
C 2.884585 -1.660655 1.069013
C 1.963734 0.367755 0.110756
H 2.758646 -2.549280 1.680022

```
C        4.120994       -1.378284        0.491271
C        3.221541        0.642684       -0.430446
F        3.391315        1.752824       -1.153469
C        4.291867       -0.221473       -0.264943
H        5.241117        0.024458       -0.728825
H        0.851833       -0.973190        1.339852
N        0.934676        1.355725       -0.039452
N       -0.230887        0.924301        0.012599
C       -1.465196        0.477598        0.028898
C       -1.940310       -0.830802       -0.260240
C       -2.665360        1.230999        0.306259
N       -3.732593        0.469198        0.211557
N       -3.271847       -0.761396       -0.163529
C       -4.230988       -1.795507       -0.457733
H       -4.028390       -2.694102        0.131901
H       -5.212390       -1.402677       -0.195494
H       -4.214066       -2.054628       -1.521113
C       -1.199352       -2.035348       -0.695334
H       -0.129693       -1.872874       -0.543665
H       -1.507331       -2.923986       -0.134041
H       -1.355987       -2.245774       -1.759934
C       -2.728133        2.657935        0.707566
H       -2.506951        2.778134        1.773645
H       -1.977041        3.230031        0.155603
H       -3.721766        3.066669        0.515675
H        4.959193       -2.053064        0.634386
30
4pzMeF1B-ts-H-n ; E = -782.999188949 Hartree
C       -1.966820        0.884034        0.718549
C       -3.122800        1.646912        0.820582
C       -1.994027       -0.390434        0.151027
H       -3.086466        2.631774        1.276232
C       -4.332326        1.138081        0.350022
C       -3.223476       -0.887945       -0.286438
F       -3.279670       -2.111292       -0.821622
C       -4.384783       -0.135974       -0.208867
H       -5.309968       -0.558974       -0.585483
H       -1.015372        1.245537        1.096669
N       -0.859342       -1.267512        0.097030
N        0.250265       -0.702035        0.061720
C        1.428060       -0.128392       -0.035560
C        2.677159       -0.774447        0.161591
C        1.790887        1.228743       -0.382843
N        3.097958        1.373442       -0.393409
N        3.610575        0.156542       -0.037133
C        5.037119        0.032864        0.122568
H        5.496149        0.868818       -0.404067
H        5.320423        0.072402        1.179414
H        5.386739       -0.908804       -0.306919
C        2.928618       -2.180556        0.541875
H        3.358748       -2.265369        1.546148
H        1.971494       -2.707876        0.530091
H        3.612579       -2.673297       -0.157381
C        0.889956        2.352440       -0.749716
H        0.095873        2.013842       -1.419550
H        0.403988        2.775047        0.136546
H        1.461730        3.145249       -1.235243
H       -5.240466        1.728274        0.423939
```

30
4pzMeF2-ts-B-m ; E = -882.143178529 Hartree

C	-2.600534	-0.937043	-0.782663
C	-3.866910	-0.453508	-1.031242
C	-1.763493	-0.397486	0.223939
H	-4.440049	-0.912706	-1.829646
C	-4.387993	0.580671	-0.253135
C	-2.323980	0.668052	0.965445
F	-1.535651	1.219664	1.912597
C	-3.602689	1.137536	0.754882
H	-3.961553	1.947694	1.380680
F	-2.085078	-1.929741	-1.525895
N	-0.553284	-0.859090	0.455673
N	0.575785	-1.305128	0.652064
C	1.663483	-0.543704	0.241121
C	1.742282	0.745689	-0.304400
C	3.002430	-1.006191	0.340397
N	3.837521	-0.087341	-0.109453
N	3.054995	0.957926	-0.489062
C	3.698747	2.142310	-1.006730
H	3.327113	2.383359	-2.005999
H	3.540314	2.997860	-0.344004
H	4.763101	1.919799	-1.059708
C	0.683131	1.740873	-0.605329
H	0.028769	1.387358	-1.408275
H	0.053144	1.894362	0.275018
H	1.106256	2.702275	-0.903985
C	3.481049	-2.318700	0.848838
H	3.108908	-2.502872	1.860390
H	3.120644	-3.137334	0.218862
H	4.572158	-2.334395	0.858623
H	-5.390502	0.949730	-0.433566

30
4pzMeF2-ts-B-n ; E = -882.145235530 Hartree

C	2.433208	0.844680	0.793600
C	3.748891	1.183820	0.561423
C	1.841477	-0.331216	0.277029
H	4.131008	2.094426	1.010249
C	4.540928	0.372997	-0.250089
C	2.687224	-1.130002	-0.529208
F	2.144293	-2.238382	-1.058626
C	3.991548	-0.781768	-0.807178
H	4.569809	-1.438318	-1.448465
F	1.643349	1.632669	1.550128
N	0.593844	-0.662828	0.532657
N	-0.566666	-0.978787	0.784863
C	-1.587651	-0.243527	0.192906
C	-2.925109	-0.586560	0.394134
C	-1.603361	0.905432	-0.653815
N	-2.850885	1.230205	-0.940018
N	-3.631166	0.320660	-0.298802
C	-5.060808	0.383665	-0.468904
H	-5.303095	1.387542	-0.815017
H	-5.404166	-0.345831	-1.209583
H	-5.563156	0.192887	0.482139
C	-3.511699	-1.697101	1.182068
H	-4.090771	-2.378677	0.549803
H	-2.700969	-2.259081	1.648426

H -4.176129 -1.324726 1.969379
C -0.461547 1.708850 -1.163909
H 0.148396 2.082065 -0.336426
H 0.194447 1.107595 -1.800928
H -0.840513 2.553535 -1.742201
H 5.571864 0.639635 -0.450145
30
4pzMeF2-ts-H-m ; E = -882.139490459 Hartree
C 1.881563 -0.774770 0.752217
C 2.976028 -1.622711 0.816690
C 1.909149 0.448233 0.074527
H 2.891444 -2.552232 1.368668
C 4.156173 -1.244485 0.184422
C 3.126624 0.795816 -0.517795
F 3.203087 1.963006 -1.160163
C 4.238100 -0.030453 -0.491644
H 5.144370 0.287700 -0.994305
F 0.757697 -1.137300 1.391124
N 0.857083 1.427790 0.086758
N -0.298988 0.971185 0.029203
C -1.531125 0.532346 -0.013786
C -1.984835 -0.764159 -0.382890
C -2.744899 1.252478 0.293976
N -3.799770 0.484904 0.140691
N -3.314379 -0.722064 -0.288965
C -4.255927 -1.756392 -0.635447
H -3.977049 -2.702904 -0.165325
H -5.230578 -1.438431 -0.267588
H -4.305799 -1.895782 -1.720296
C -1.219614 -1.936968 -0.861066
H -0.161778 -1.676192 -0.904745
H -1.335831 -2.793012 -0.187564
H -1.541025 -2.250123 -1.860503
C -2.836082 2.653668 0.773040
H -2.571993 2.720797 1.833844
H -2.128269 3.282293 0.225633
H -3.849516 3.037591 0.644422
H 5.020405 -1.899380 0.221852
30
4pzMeF2-ts-H-n ; E = -882.140107338 Hartree
C -2.052512 -0.782106 -0.727564
C -3.238367 -1.498634 -0.709823
C -1.924611 0.484652 -0.153094
H -3.273288 -2.474124 -1.182032
C -4.353814 -0.933928 -0.099376
C -3.080582 1.021931 0.417921
F -3.006904 2.240148 0.960304
C -4.281107 0.332893 0.472705
H -5.134220 0.795630 0.955726
F -0.991510 -1.320417 -1.350584
N -0.760534 1.324381 -0.256522
N 0.329055 0.743618 -0.097610
C 1.501800 0.189208 0.082622
C 2.763059 0.781980 -0.194137
C 1.837364 -1.115563 0.609377
N 3.140094 -1.282937 0.644203
N 3.677130 -0.128340 0.137230
C 5.105602 -0.062828 -0.037413

H	5.454885	0.959681	0.118296
H	5.560080	-0.723170	0.700849
H	5.394423	-0.393654	-1.040362
C	3.037863	2.117843	-0.764976
H	2.086917	2.649660	-0.848729
H	3.712151	2.700568	-0.128320
H	3.488561	2.054193	-1.761505
C	0.903380	-2.154373	1.112829
H	0.315810	-1.771878	1.953341
H	0.198953	-2.441069	0.328798
H	1.461100	-3.032418	1.442423
H	-5.288312	-1.484498	-0.071500

34

4pzMeOMe1A-ts-B-m ; E = -798.262855518 Hartree

C	2.412689	0.229820	0.496066
C	3.692503	0.532637	0.071986
C	1.688809	-0.840628	-0.107280
H	4.234494	1.345013	0.544345
C	4.291838	-0.184768	-0.971275
C	2.305992	-1.557889	-1.153929
C	3.585580	-1.213771	-1.576841
H	4.033202	-1.778855	-2.389372
N	0.468900	-1.113742	0.320374
N	-0.667757	-1.408261	0.677778
C	-1.718008	-0.612697	0.216467
C	-1.716584	0.573349	-0.527620
C	-3.080642	-0.905759	0.478995
N	-3.859765	0.014905	-0.062146
N	-3.015125	0.895011	-0.660384
C	-3.580824	2.045952	-1.322602
H	-3.262378	2.091512	-2.367342
H	-4.662502	1.930236	-1.278339
H	-3.295320	2.972805	-0.816444
C	-0.592390	1.387929	-1.053244
H	0.112169	1.607795	-0.245404
H	-0.032402	0.841149	-1.818329
H	-0.942453	2.328078	-1.485220
C	-3.640310	-2.061620	1.228991
H	-3.440990	-3.002367	0.706787
H	-3.183543	-2.140271	2.219307
H	-4.719660	-1.942574	1.338437
H	5.295049	0.069616	-1.294559
H	1.760110	-2.372252	-1.616715
O	1.714072	0.882672	1.474368
C	2.391549	1.900456	2.168510
H	3.288909	1.515439	2.669715
H	2.683977	2.722913	1.501615
H	1.693792	2.276943	2.917879

34

4pzMeOMe1A-ts-B-n ; E = -798.264651160 Hartree

C	-2.487367	0.438370	-0.366438
C	-3.804931	0.535171	0.039565
C	-1.787810	-0.798773	-0.244499
H	-4.328843	1.479362	-0.064082
C	-4.464614	-0.563946	0.604346
C	-2.468527	-1.900785	0.312705
C	-3.783747	-1.764357	0.744238
H	-4.279311	-2.625451	1.183215

N	-0.529235	-0.864421	-0.642264
N	0.633737	-0.928288	-1.026856
C	1.616729	-0.359201	-0.214633
C	2.964630	-0.433530	-0.558879
C	1.567229	0.367037	1.011519
N	2.790815	0.705521	1.379513
N	3.617912	0.215650	0.420102
C	5.040776	0.386421	0.566398
H	5.484432	0.717336	-0.376032
H	5.197284	1.147326	1.329738
H	5.522483	-0.545099	0.881291
C	3.613136	-1.072071	-1.729652
H	4.301892	-1.868251	-1.426728
H	2.837311	-1.505117	-2.363184
H	4.181590	-0.346226	-2.321488
C	0.382159	0.764138	1.816602
H	-0.308211	1.359757	1.212013
H	-0.175714	-0.110655	2.164520
H	0.707195	1.346766	2.680778
H	-5.495512	-0.466668	0.926266
H	-1.939636	-2.842333	0.408207
O	-1.733824	1.445936	-0.898041
C	-2.366621	2.688369	-1.076396
H	-3.221176	2.610228	-1.761133
H	-2.713905	3.107698	-0.122509
H	-1.618134	3.353551	-1.509263

34

4pzMeOMe1A-ts-H-m ; E = -798.257489312 Hartree

C	-2.987070	0.273925	0.133586
C	-3.946719	-0.728480	-0.017323
C	-1.674360	0.049891	-0.329897
H	-4.954571	-0.583368	0.353896
C	-3.615422	-1.928404	-0.645537
C	-1.369395	-1.152402	-0.962757
C	-2.329640	-2.145330	-1.126874
H	-2.081322	-3.070462	-1.637770
N	-0.742603	1.135637	-0.245194
N	0.456810	0.809798	-0.191655
C	1.725659	0.483902	-0.096026
C	2.293471	-0.726987	0.382601
C	2.870457	1.302637	-0.415957
N	3.992808	0.660888	-0.173417
N	3.618972	-0.548470	0.339063
C	4.643949	-1.444705	0.808742
H	4.557013	-2.423597	0.328331
H	5.601851	-0.996601	0.547897
H	4.585328	-1.573888	1.894244
C	1.630070	-1.930201	0.931379
H	1.717387	-1.981768	2.023234
H	0.565714	-1.893194	0.685865
H	2.058675	-2.851007	0.521583
C	2.833395	2.666808	-0.998563
H	3.772282	3.190477	-0.809620
H	2.672613	2.630243	-2.081567
H	2.000014	3.228809	-0.567420
H	-4.379025	-2.691734	-0.762666
H	-0.362644	-1.273332	-1.354186
O	-3.216785	1.462793	0.731837

C -4.525601 1.731779 1.172480
H -5.248029 1.693821 0.346896
H -4.842453 1.031867 1.956835
H -4.503230 2.741829 1.583045
34
4pzMeOMe1A-ts-H-n ; E = -798.257844335 Hartree
C 3.013536 -0.419966 0.050158
C 4.059668 0.493657 -0.087477
C 1.708727 -0.038032 -0.323234
H 5.065032 0.226656 0.216711
C 3.818650 1.761682 -0.615044
C 1.494013 1.229366 -0.857560
C 2.538789 2.134722 -1.008455
H 2.358431 3.114121 -1.440506
N 0.678081 -1.030803 -0.254965
N -0.477709 -0.589661 -0.118253
C -1.702754 -0.158509 0.076778
C -2.895544 -0.848478 -0.258363
C -2.168552 1.078814 0.662048
N -3.484529 1.125483 0.669540
N -3.899586 -0.032191 0.077826
C -5.307159 -0.214899 -0.165632
H -5.550634 -0.051209 -1.220707
H -5.617270 -1.223287 0.119829
H -5.837064 0.516176 0.443900
C -3.040541 -2.168414 -0.908637
H -3.401065 -2.087253 -1.940745
H -2.056872 -2.644667 -0.926875
H -3.736533 -2.814056 -0.362542
C -1.343438 2.144685 1.283964
H -0.446602 2.338098 0.688205
H -1.925054 3.062499 1.388104
H -0.999502 1.840888 2.278663
H 4.647668 2.454822 -0.723617
H 0.485942 1.473044 -1.182094
O 3.152802 -1.665666 0.554840
C 4.447055 -2.085257 0.912396
H 5.133725 -2.060328 0.056263
H 4.864002 -1.472217 1.722206
H 4.344893 -3.113990 1.259802
34
4pzMeOMe1B-ts-B-m ; E = -798.262855555 Hartree
C -2.305136 -1.557357 -1.154731
C -3.584759 -1.213697 -1.577920
C -1.688700 -0.840276 -0.107521
H -4.031858 -1.778728 -2.390777
C -4.291654 -0.185160 -0.972310
C -2.413211 0.229714 0.495858
C -3.692945 0.532224 0.071326
H -4.235379 1.344314 0.543669
N -0.468895 -1.113131 0.320612
N 0.667722 -1.407658 0.678109
C 1.718123 -0.612376 0.216668
C 1.717037 0.573559 -0.527611
C 3.080679 -0.905757 0.479268
N 3.860060 0.014634 -0.061958
N 3.015667 0.894877 -0.660340
C 3.581676 2.045754 -1.322409

H	3.262907	2.091745	-2.367027
H	3.296758	2.972596	-0.815896
H	4.663315	1.929539	-1.278525
C	0.593084	1.388740	-1.052833
H	0.032968	0.842613	-1.818288
H	-0.111446	1.608301	-0.244881
H	0.943427	2.329064	-1.484199
C	3.639999	-2.061762	1.229312
H	4.719135	-1.942188	1.340282
H	3.181886	-2.141314	2.218925
H	3.441971	-3.002296	0.706217
H	-5.294858	0.068903	-1.295867
O	-1.715142	0.882660	1.474491
C	-2.393676	1.899104	2.169563
H	-2.686277	2.722234	1.503574
H	-3.291059	1.512841	2.669763
H	-1.696583	2.275101	2.919798
H	-1.758746	-2.371332	-1.617598

34
4pzMeOMe1B-ts-B-n ; E = -798.264651155 Hartree

C	2.468297	-1.900864	0.312575
C	3.783487	-1.764605	0.744246
C	1.787746	-0.798736	-0.244610
H	4.278915	-2.625784	1.183208
C	4.464493	-0.564252	0.604519
C	2.487449	0.438336	-0.366401
C	3.804979	0.534973	0.039755
H	4.328995	1.479120	-0.063765
N	0.529200	-0.864218	-0.642496
N	-0.633778	-0.928012	-1.027078
C	-1.616755	-0.359044	-0.214750
C	-2.964668	-0.433417	-0.558926
C	-1.567227	0.367061	1.011485
N	-2.790811	0.705419	1.379595
N	-3.617938	0.215587	0.420183
C	-5.040811	0.386224	0.566605
H	-5.484587	0.716959	-0.375830
H	-5.522385	-0.545308	0.881670
H	-5.197350	1.147212	1.329858
C	-3.613197	-1.071812	-1.729769
H	-4.180995	-0.345726	-2.321943
H	-2.837438	-1.505448	-2.362979
H	-4.302557	-1.867489	-1.426906
C	-0.382128	0.764195	1.816508
H	0.175713	-0.110584	2.164515
H	0.308254	1.359721	1.211843
H	-0.707128	1.346926	2.680629
H	5.495367	-0.467103	0.926554
O	1.734064	1.446025	-0.897999
C	2.367021	2.688395	-1.076226
H	2.714249	3.107640	-0.122281
H	3.221641	2.610190	-1.760874
H	1.618656	3.353683	-1.509141
H	1.939312	-2.842376	0.407936

34
4pzMeOMe1B-ts-H-m ; E = -798.257730523 Hartree

C	3.120486	1.463268	-0.791091
C	4.313096	0.757483	-0.891426

```
C        1.997880        0.913743       -0.177868
H        5.168945        1.194216       -1.395964
C        4.396138       -0.510757       -0.328328
C        2.099410       -0.362186        0.420916
C        3.302688       -1.064597        0.334716
H        3.397918       -2.044877        0.787772
N        0.849615        1.770915       -0.119496
N       -0.259371        1.207698       -0.125321
C       -1.451278        0.667210       -0.113614
C       -1.820565       -0.628011       -0.564029
C       -2.691937        1.242933        0.347419
N       -3.688069        0.395768        0.203643
N       -3.143538       -0.713114       -0.379550
C       -4.025454       -1.772283       -0.796028
H       -3.626139       -2.745286       -0.498052
H       -4.983211       -1.605139       -0.304507
H       -4.170165       -1.763559       -1.881729
C       -1.000332       -1.666471       -1.226764
H       -1.281191       -1.802145       -2.277903
H        0.046218       -1.360555       -1.186003
H       -1.101085       -2.637983       -0.729923
C       -2.858790        2.578661        0.972034
H       -3.902514        2.895104        0.927635
H       -2.547250        2.565003        2.022178
H       -2.227024        3.309234        0.458695
H        5.320772       -1.077294       -0.387071
O        1.009375       -0.820267        1.086377
C        1.131522       -2.025040        1.801781
H        1.316109       -2.877483        1.134273
H        1.935233       -1.975413        2.547230
H        0.176833       -2.169115        2.309325
H        3.013905        2.464849       -1.198211
34
4pzMeOMe1B-ts-H-n ; E = -798.257532508 Hartree
C       -3.088793        1.680476        0.514380
C       -4.347018        1.100162        0.619087
C       -1.994240        0.964499        0.038127
H       -5.181946        1.667105        1.018143
C       -4.521370       -0.212602        0.196837
C       -2.185650       -0.357703       -0.421041
C       -3.453390       -0.934947       -0.332093
H       -3.617829       -1.949292       -0.677310
N       -0.767805        1.703259       -0.046210
N        0.280644        1.052166        0.109041
C        1.417502        0.420980        0.263433
C        2.688099        0.836483       -0.209349
C        1.682476       -0.843632        0.910856
N        2.960750       -1.150788        0.831779
N        3.540093       -0.140546        0.119510
C        4.928490       -0.261380       -0.242036
H        5.453447        0.681176       -0.067000
H        5.358509       -1.040389        0.386563
H        5.041073       -0.542451       -1.294617
C        3.030156        2.051031       -0.980448
H        3.275758        1.826422       -2.025044
H        2.161101        2.713772       -0.970186
H        3.883913        2.580546       -0.544176
C        0.719345       -1.670499        1.681778
```

H	-0.276772	-1.582471	1.243641
H	1.033083	-2.716615	1.689076
H	0.655844	-1.331152	2.721593
H	-5.497356	-0.684405	0.262068
O	-1.112340	-0.983262	-0.964903
C	-1.304978	-2.260086	-1.524207
H	-1.605055	-2.994326	-0.765208
H	-2.056376	-2.243452	-2.323905
H	-0.339748	-2.552843	-1.938791
H	-2.908469	2.709148	0.813590

38

4pzMeOMe2-ts-B-m ; E = -912.662873854 Hartree

C	2.460387	-1.055712	0.140493
C	3.714023	-0.640407	0.568365
C	1.526342	-0.115680	-0.367849
H	4.416955	-1.365228	0.962702
C	4.082833	0.700853	0.472385
C	1.909853	1.249762	-0.415204
C	3.183417	1.641576	-0.024418
H	3.473147	2.684181	-0.089774
N	0.331732	-0.497006	-0.781014
N	-0.774837	-0.803808	-1.217725
C	-1.893327	-0.435906	-0.468249
C	-2.008214	0.235688	0.754382
C	-3.219612	-0.735599	-0.871633
N	-4.084957	-0.284830	0.020602
N	-3.330993	0.295281	0.990490
C	-4.005046	0.885343	2.121603
H	-5.071305	0.733611	1.961879
H	-3.705788	0.400614	3.055192
H	-3.797686	1.957149	2.188630
C	-0.970806	0.794617	1.656824
H	-0.289428	0.007843	1.994377
H	-0.363661	1.528585	1.119023
H	-1.412489	1.271240	2.534869
C	-3.662936	-1.443866	-2.101938
H	-4.749719	-1.544907	-2.098137
H	-3.359997	-0.896261	-2.999265
H	-3.210466	-2.437589	-2.164397
H	5.071778	1.013120	0.788809
O	0.929753	2.077124	-0.889327
C	1.265974	3.431471	-1.058795
H	1.537890	3.906856	-0.106506
H	2.097086	3.553796	-1.765143
H	0.376779	3.918842	-1.461377
O	1.981161	-2.329359	0.189305
C	2.861912	-3.324636	0.644840
H	3.764321	-3.383754	0.021944
H	3.162240	-3.154919	1.687696
H	2.317623	-4.267778	0.578329

38

4pzMeOMe2-ts-B-n ; E = -912.664763212 Hartree

C	2.497542	-1.133133	0.012137
C	3.796970	-0.824368	0.391763
C	1.593731	-0.103753	-0.357720
H	4.476350	-1.616195	0.685617
C	4.239427	0.497418	0.375397
C	2.056560	1.237014	-0.331300

C 3.372484 1.523824 0.006809
H 3.721560 2.550057 0.001933
N 0.353758 -0.383234 -0.716345
N -0.792370 -0.610673 -1.091621
C -1.838651 -0.235449 -0.245877
C -3.165332 -0.459254 -0.606067
C -1.884034 0.373514 1.042595
N -3.141006 0.511732 1.428777
N -3.897821 0.007431 0.420299
C -5.331485 -0.009396 0.556331
H -5.807920 0.449188 -0.314696
H -5.706646 -1.031484 0.671505
H -5.574527 0.565889 1.448482
C -3.727098 -1.070837 -1.834460
H -4.360397 -0.365503 -2.384069
H -2.900726 -1.371893 -2.480488
H -4.333079 -1.954321 -1.605452
C -0.760278 0.826807 1.903813
H -0.095967 -0.005668 2.155507
H -0.152772 1.571785 1.382289
H -1.157643 1.257227 2.825245
H 5.261643 0.727974 0.654123
O 1.106512 2.150924 -0.688114
C 1.499854 3.499959 -0.720368
H 1.839678 3.848250 0.264241
H 2.300609 3.668814 -1.452080
H 0.616823 4.068171 -1.016239
O 1.946949 -2.378375 -0.008825
C 2.785254 -3.451309 0.336447
H 3.652376 -3.521486 -0.333841
H 3.143698 -3.369487 1.371420
H 2.181663 -4.354722 0.237247
38
4pzMeOMe2-ts-H-m ; E = -912.657076258 Hartree
C 2.934613 -0.616354 0.222884
C 3.965387 0.306212 0.412079
C 1.667570 -0.197941 -0.225630
H 4.933684 -0.008754 0.780722
C 3.735942 1.647186 0.127323
C 1.469807 1.165191 -0.519748
C 2.505016 2.087587 -0.340994
H 2.359127 3.136614 -0.567823
N 0.711656 -1.227432 -0.511491
N -0.476582 -0.944637 -0.276935
C -1.745942 -0.693325 -0.078280
C -2.303948 0.319442 0.744959
C -2.896875 -1.367677 -0.629020
N -4.014669 -0.825204 -0.196509
N -3.630479 0.174818 0.653363
C -4.648484 0.878822 1.388642
H -4.467207 1.956511 1.360776
H -5.602159 0.656832 0.910943
H -4.682445 0.550057 2.432912
C -1.620192 1.296867 1.620150
H -1.795525 1.090641 2.682544
H -0.547046 1.236589 1.429287
H -1.955784 2.321007 1.421896
C -2.873479 -2.487887 -1.602153

H	-3.826511	-3.020123	-1.599069
H	-2.685683	-2.122663	-2.617738
H	-2.061624	-3.177313	-1.353659
H	4.536947	2.365910	0.272135
O	0.249958	1.503845	-1.008755
C	0.059651	2.824207	-1.453043
H	0.133168	3.545779	-0.628391
H	0.780933	3.099449	-2.232817
H	-0.949194	2.857067	-1.866710
O	3.051768	-1.938637	0.476318
C	4.312984	-2.420273	0.870816
H	5.083577	-2.206428	0.118996
H	4.627100	-1.997577	1.834258
H	4.203062	-3.500413	0.974327

38
4pzMeOMe2-ts-H-n ; E = -912.656984075 Hartree

C	2.927085	-0.779551	0.082036
C	4.052398	0.028417	0.256099
C	1.682175	-0.216279	-0.253191
H	5.006341	-0.398638	0.539497
C	3.936525	1.400604	0.068158
C	1.598867	1.174827	-0.452406
C	2.727627	1.983061	-0.289565
H	2.669961	3.053829	-0.441849
N	0.614393	-1.130694	-0.536725
N	-0.520990	-0.764895	-0.184302
C	-1.744312	-0.433403	0.146214
C	-2.945489	-0.964071	-0.388313
C	-2.193861	0.554732	1.099045
N	-3.508907	0.612417	1.131354
N	-3.939355	-0.293790	0.203117
C	-5.349830	-0.378943	-0.072264
H	-5.868786	0.118476	0.746440
H	-5.600801	0.118994	-1.014991
H	-5.667232	-1.423499	-0.125919
C	-3.108399	-1.998375	-1.432395
H	-3.494643	-1.584359	-2.371098
H	-2.125979	-2.434947	-1.629575
H	-3.791065	-2.793306	-1.113141
C	-1.342279	1.359478	2.011012
H	-0.410078	1.622983	1.506212
H	-1.866304	2.264222	2.326195
H	-1.080671	0.788927	2.908940
H	4.810344	2.031130	0.202439
O	0.390299	1.655311	-0.839142
C	0.294947	3.020340	-1.165240
H	0.492148	3.660498	-0.295510
H	0.983317	3.293917	-1.974958
H	-0.732970	3.176871	-1.494496
O	2.930344	-2.121712	0.247136
C	4.159756	-2.742093	0.532409
H	4.903089	-2.553515	-0.252996
H	4.568186	-2.412197	1.496705
H	3.952745	-3.811911	0.580446

42
4pzMePyr1A-ts-B-m ; E = -895.029461975 Hartree

C	2.248342	-0.247790	-0.003120
C	3.470780	-0.589031	0.560752

C	1.385049	-1.293878	-0.453866
H	4.121761	0.206373	0.909948
C	3.869820	-1.918258	0.713359
C	1.809891	-2.636392	-0.332387
C	3.029070	-2.929102	0.262843
H	3.319879	-3.970598	0.368338
N	0.196579	-0.997282	-0.956395
N	-0.928524	-0.812062	-1.415784
C	-1.954540	-0.513562	-0.518951
C	-1.926620	-0.244166	0.855439
C	-3.311657	-0.387930	-0.913177
N	-4.064300	-0.077258	0.127845
N	-3.207107	0.005533	1.179388
C	-3.737755	0.374553	2.469752
H	-3.461399	-0.361406	3.228971
H	-3.380304	1.362100	2.776604
H	-4.821296	0.400452	2.366296
C	-0.788228	-0.168152	1.803487
H	-1.087460	0.267037	2.759763
H	-0.361468	-1.158854	1.990239
H	0.008421	0.440105	1.357928
C	-3.891873	-0.579650	-2.269088
H	-3.830321	-1.627634	-2.578528
H	-4.940320	-0.276458	-2.268581
H	-3.345490	0.007812	-3.011913
H	4.824096	-2.151058	1.173122
C	1.079631	3.063068	-1.124306
C	1.576090	1.648495	-1.429181
N	1.761191	1.073475	-0.095648
C	2.433225	2.115374	0.658657
C	1.650517	3.375625	0.279090
H	1.412081	3.775875	-1.883112
H	0.874339	1.061631	-2.024333
H	2.415943	1.886450	1.728890
H	2.290828	4.261484	0.287121
H	2.537568	1.676764	-1.971277
H	-0.013314	3.085377	-1.108087
H	0.840697	3.550158	0.992763
H	3.490528	2.225626	0.354370
H	1.149905	-3.423291	-0.680175

42

4pzMePyr1A-ts-B-n ; E = -895.030481512 Hartree

C	-2.346148	0.114631	-0.052709
C	-3.639631	0.333003	0.403455
C	-1.572798	1.236491	-0.483207
H	-4.223323	-0.518975	0.738025
C	-4.191109	1.613902	0.472970
C	-2.152696	2.524221	-0.457687
C	-3.436023	2.698395	0.041793
H	-3.847462	3.702980	0.084518
N	-0.321642	1.053704	-0.876649
N	0.837738	0.884303	-1.245512
C	1.797904	0.612527	-0.269225
C	3.138337	0.442506	-0.610226
C	1.729644	0.435068	1.144672
N	2.935730	0.176172	1.619815
N	3.770821	0.189942	0.548103
C	5.172790	-0.076890	0.744205

H	5.783169	0.733001	0.334556
H	5.336299	-0.147052	1.818505
H	5.465387	-1.019349	0.270529
C	3.800433	0.508919	-1.935323
H	4.302161	-0.432764	-2.183593
H	3.042364	0.711280	-2.693724
H	4.551421	1.305892	-1.972344
C	0.540766	0.484782	2.034757
H	0.074579	1.474870	2.021576
H	-0.214995	-0.226400	1.686101
H	0.838903	0.244683	3.057176
H	-5.196817	1.753272	0.854534
C	-0.718557	-3.068268	-0.936693
C	-1.379434	-1.751366	-1.347110
N	-1.709148	-1.142478	-0.059166
C	-2.289011	-2.222630	0.716436
C	-1.337304	-3.391942	0.443842
H	-0.895774	-3.854708	-1.674841
H	-0.728899	-1.107570	-1.941703
H	-2.357202	-1.942498	1.772219
H	-1.866698	-4.348297	0.454935
H	-2.297564	-1.937543	-1.932592
H	0.362886	-2.933664	-0.848375
H	-0.560148	-3.436972	1.211282
H	-3.306749	-2.479997	0.368318
H	-1.559017	3.368211	-0.790948

42

4pzMePyr1A-ts-H-m ; E = -895.026095654 Hartree

C	2.896350	-2.179812	-0.103201
C	4.008023	-1.867226	0.666878
C	1.891334	-1.246183	-0.344112
H	4.763806	-2.617093	0.876961
C	4.115383	-0.580904	1.188090
C	2.056888	0.100884	0.072370
C	3.165671	0.388609	0.889725
H	3.304712	1.393098	1.272915
N	0.721351	-1.800102	-0.978142
N	-0.361454	-1.368981	-0.536615
C	-1.530395	-0.937655	-0.125612
C	-1.811010	-0.182324	1.044211
C	-2.820169	-1.103919	-0.751636
N	-3.764418	-0.511928	-0.053024
N	-3.134650	0.014129	1.040090
C	-3.937969	0.625873	2.066809
H	-3.497691	1.574642	2.384293
H	-4.924075	0.805370	1.639922
H	-4.035332	-0.031189	2.937594
C	-0.905720	0.239496	2.136020
H	-1.139841	-0.268204	3.079094
H	0.118705	-0.008571	1.852644
H	-0.967947	1.318709	2.317118
C	-3.085111	-1.784030	-2.043944
H	-2.476936	-2.689891	-2.119117
H	-4.142522	-2.039179	-2.134026
H	-2.812636	-1.143042	-2.889876
H	4.962889	-0.313210	1.812819
C	0.459791	2.616273	-1.979344
C	0.602525	1.127938	-1.664228

N	1.178760	1.098579	-0.314904
C	1.328117	2.445582	0.206721
C	0.311623	3.242549	-0.595480
H	1.369471	2.995187	-2.459018
H	1.251739	0.599434	-2.371654
H	1.147252	2.464919	1.288460
H	-0.697087	3.066222	-0.204374
H	2.341440	2.847139	0.032104
H	0.508028	4.317406	-0.575219
H	-0.382178	2.820324	-2.645595
H	-0.375298	0.636570	-1.684239
H	2.734387	-3.181426	-0.492254

42

4pzMePyr1A-ts-H-n ; E = -895.024254913 Hartree

C	3.175439	-2.012352	-0.114171
C	4.402927	-1.496558	0.278378
C	2.052546	-1.199140	-0.252058
H	5.258157	-2.150699	0.414673
C	4.509486	-0.128560	0.507569
C	2.174294	0.208364	-0.110252
C	3.418937	0.709438	0.300641
H	3.542710	1.778008	0.437687
N	0.846987	-1.928969	-0.537403
N	-0.196107	-1.439045	-0.065286
C	-1.339308	-0.941273	0.332708
C	-2.611456	-1.144195	-0.259131
C	-1.593227	0.000937	1.398730
N	-2.868303	0.333163	1.433578
N	-3.453121	-0.335446	0.398532
C	-4.831976	-0.058222	0.090578
H	-4.919775	0.586672	-0.790527
H	-5.378825	-0.986837	-0.091858
H	-5.258089	0.451923	0.953699
C	-2.960047	-1.975952	-1.431489
H	-3.172553	-1.374424	-2.323657
H	-2.108582	-2.624131	-1.654195
H	-3.837290	-2.602160	-1.237318
C	-0.619933	0.459411	2.421693
H	-0.632222	-0.197955	3.298135
H	0.386110	0.438343	1.997327
H	-0.860955	1.470025	2.759817
H	5.455880	0.301711	0.822618
C	-0.662701	2.087355	-1.532930
C	0.431877	1.022817	-1.658306
N	1.101652	1.064307	-0.362338
C	1.194410	2.467392	-0.012884
C	-0.189228	3.003226	-0.383107
H	-0.792652	2.629584	-2.473402
H	1.143758	1.285373	-2.459902
H	1.431667	2.584229	1.049378
H	-0.865605	2.911499	0.470481
H	1.977921	2.988899	-0.594158
H	-0.150110	4.057987	-0.667277
H	-1.619926	1.624529	-1.279939
H	0.040625	0.029584	-1.877948
H	3.029758	-3.076354	-0.279029

42

4pzMePyr1B-ts-B-m ; E = -895.029462007 Hartree

C	-1.808410	-2.636613	-0.333061
C	-3.027375	-2.930182	0.262191
C	-1.384351	-1.293823	-0.454228
H	-3.317580	-3.971875	0.367414
C	-3.868655	-1.919947	0.713073
C	-2.248227	-0.248348	-0.003167
C	-3.470393	-0.590452	0.560772
H	-4.121800	0.204485	0.910237
N	-0.196089	-0.996430	-0.956780
N	0.928953	-0.810916	-1.416212
C	1.954938	-0.512775	-0.519236
C	1.926699	-0.243941	0.855246
C	3.312115	-0.386895	-0.913144
N	4.064511	-0.076599	0.128174
N	3.207094	0.005690	1.179583
C	3.737452	0.374302	2.470181
H	4.821056	0.399461	2.367214
H	3.380545	1.362081	2.776920
H	3.460249	-0.361499	3.229238
C	0.787963	-0.168377	1.802911
H	-0.008389	0.440391	1.357511
H	0.360882	-1.159144	1.988668
H	1.086905	0.265973	2.759660
C	3.892606	-0.578093	-2.269012
H	4.940889	-0.274338	-2.268351
H	3.831665	-1.626062	-2.578626
H	3.345977	0.009187	-3.011799
H	-4.822747	-2.153418	1.172878
C	-1.080932	3.063197	-1.123443
C	-1.577085	1.648664	-1.428808
N	-1.761880	1.073227	-0.095427
C	-2.434530	2.114568	0.659103
C	-1.652885	3.375533	0.279567
H	0.012005	3.085382	-1.106293
H	-0.875272	1.062159	-2.024243
H	-3.491968	2.224051	0.355014
H	-2.294218	4.260660	0.286928
H	-2.416906	1.885523	1.729304
H	-0.843672	3.551286	0.993625
H	-1.412671	3.776132	-1.882439
H	-2.538644	1.676902	-1.970771
H	-1.148008	-3.423039	-0.681130

42

4pzMePyr1B-ts-B-n ; E = -895.029972383 Hartree

C	-2.071177	-2.557382	-0.557601
C	-3.318233	-2.807510	-0.002578
C	-1.510990	-1.260390	-0.515690
H	-3.701640	-3.823588	0.003282
C	-4.072087	-1.764985	0.521232
C	-2.297909	-0.175373	-0.012667
C	-3.565952	-0.464598	0.488843
H	-4.165785	0.341828	0.895864
N	-0.256802	-1.073078	-0.908766
N	0.921952	-0.957628	-1.246410
C	1.847293	-0.586411	-0.264028
C	3.185141	-0.374577	-0.591573
C	1.754226	-0.369101	1.143562
N	2.942744	-0.045593	1.625090

N	3.790106	-0.049505	0.564195
C	5.184213	0.244499	0.774856
H	5.804316	-0.644096	0.618504
H	5.519469	1.036442	0.099141
H	5.287659	0.578644	1.806153
C	3.868357	-0.465936	-1.904526
H	4.680527	-1.200964	-1.887210
H	3.137118	-0.771230	-2.654861
H	4.297071	0.496377	-2.206062
C	0.567884	-0.467231	2.035358
H	-0.216919	0.223298	1.711509
H	0.136203	-1.472413	2.007979
H	0.865172	-0.236115	3.060210
H	-5.054163	-1.948341	0.944515
C	-1.326814	3.146552	-1.130820
C	-0.999719	1.652324	-1.130365
N	-1.753218	1.110313	0.011627
C	-2.488781	2.174353	0.674443
C	-1.689620	3.419603	0.326558
H	-0.496228	3.754344	-1.499263
H	0.076833	1.497166	-0.994300
H	-3.517233	2.274206	0.284292
H	-2.260447	4.340525	0.470052
H	-2.559673	1.979253	1.750663
H	-0.786369	3.472399	0.944610
H	-2.196081	3.344307	-1.768121
H	-1.290921	1.161268	-2.066117
H	-1.467380	-3.357887	-0.971036

42

4pzMePyr1B-ts-H-m ; E = -895.028319125 Hartree

C	2.383951	0.714440	-0.199558
C	3.052384	1.959813	-0.240304
C	0.980429	0.732522	-0.432631
H	4.117938	1.998435	-0.049375
C	2.381564	3.142210	-0.506415
C	0.331351	1.939755	-0.704327
C	1.007796	3.147234	-0.745821
H	0.486340	4.068607	-0.984668
N	0.286863	-0.511674	-0.543930
N	-0.947709	-0.477303	-0.390997
C	-2.250018	-0.466082	-0.213961
C	-3.028523	0.434183	0.559142
C	-3.215801	-1.404245	-0.732009
N	-4.432883	-1.099999	-0.332613
N	-4.290865	-0.010931	0.474051
C	-5.449103	0.494641	1.163706
H	-5.601667	1.556145	0.947089
H	-6.305961	-0.074547	0.805474
H	-5.351079	0.363225	2.246288
C	-2.601614	1.570820	1.405678
H	-2.641624	1.324508	2.473741
H	-1.567081	1.827349	1.164094
H	-3.231654	2.452684	1.247050
C	-2.930415	-2.535590	-1.649131
H	-1.982264	-3.005440	-1.371527
H	-3.734477	-3.272869	-1.610045
H	-2.829410	-2.192625	-2.684742
H	2.944449	4.071144	-0.533055

C	3.721235	-2.406454	1.154612
C	2.546712	-1.717848	0.466028
N	3.096473	-0.423570	0.066276
C	4.543606	-0.390070	0.204098
C	4.925366	-1.851890	0.400525
H	3.769919	-2.107338	2.208120
H	2.196068	-2.283882	-0.403600
H	5.013174	0.053370	-0.682518
H	5.871985	-1.966732	0.934540
H	1.682930	-1.584052	1.122887
H	3.645022	-3.496104	1.115834
H	5.021235	-2.346931	-0.572227
H	4.848402	0.213424	1.075382
H	-0.728916	1.885331	-0.936057

42

4pzMePyr1B-ts-H-n ; E = -895.028306661 Hartree

C	2.448784	0.627282	-0.241023
C	3.217625	1.811200	-0.313991
C	1.039985	0.768047	-0.380000
H	4.293069	1.756003	-0.196890
C	2.633803	3.050756	-0.518964
C	0.479389	2.030192	-0.590962
C	1.252495	3.176405	-0.662385
H	0.796568	4.142989	-0.851083
N	0.237652	-0.410678	-0.462986
N	-0.977749	-0.271585	-0.232876
C	-2.258185	-0.166003	0.043539
C	-3.296509	-0.987770	-0.459817
C	-2.942872	0.774066	0.901582
N	-4.240905	0.543893	0.905275
N	-4.434989	-0.495655	0.047839
C	-5.783080	-0.897586	-0.257803
H	-5.888098	-1.983352	-0.183183
H	-6.434057	-0.418184	0.472391
H	-6.072539	-0.579930	-1.265206
C	-3.198979	-2.114286	-1.412972
H	-3.588288	-1.858941	-2.405803
H	-2.142763	-2.374830	-1.520323
H	-3.746057	-2.994274	-1.057929
C	-2.327432	1.803980	1.776716
H	-1.924242	1.352580	2.690089
H	-1.489482	2.292451	1.270851
H	-3.070969	2.549303	2.065636
H	3.271046	3.929050	-0.573783
C	3.594582	-2.603593	1.021986
C	2.443114	-1.814437	0.405829
N	3.076014	-0.572532	-0.034424
C	4.526169	-0.662526	0.018573
C	4.792957	-2.152214	0.193493
H	3.733958	-2.312587	2.069611
H	1.990543	-2.347633	-0.437187
H	4.979381	-0.256354	-0.894025
H	5.757361	-2.349425	0.668349
H	1.637774	-1.606596	1.115765
H	3.422971	-3.682656	0.991033
H	4.785843	-2.650510	-0.782340
H	4.931558	-0.091348	0.870487
H	-0.594805	2.069081	-0.749075

54
4pzMePyr2-ts-B-m ; E = -1106.20170178 Hartree

C	-2.541255	0.693174	0.307505
C	-3.082953	1.851553	0.895004
C	-1.129607	0.683928	-0.020771
H	-4.115646	1.866259	1.222750
C	-2.308835	2.994410	1.018634
C	-0.405630	1.934680	-0.006144
C	-0.997350	3.069483	0.552328
H	-0.455024	4.005322	0.616676
N	-0.501182	-0.460791	-0.188031
N	0.077637	-1.570072	-0.239949
C	1.437798	-1.593687	0.138229
C	2.146160	-0.852597	1.085245
C	2.358818	-2.556516	-0.349712
N	3.546728	-2.406201	0.219008
N	3.396857	-1.378448	1.082180
C	4.491146	-1.021134	1.944874
H	5.373627	-1.541924	1.575328
H	4.667991	0.058307	1.920775
H	4.302113	-1.324093	2.980894
C	1.733277	0.196221	2.054694
H	2.311820	1.118602	1.930972
H	0.682616	0.446066	1.915165
H	1.874364	-0.145709	3.087487
C	2.109477	-3.590598	-1.390377
H	2.060298	-3.146932	-2.391039
H	2.910580	-4.332527	-1.383068
H	1.150689	-4.085220	-1.211698
H	-2.757574	3.882026	1.457001
C	2.373260	1.988376	-2.334677
C	1.248779	1.165668	-1.705147
N	0.864366	1.945107	-0.517531
C	1.721762	3.105638	-0.355698
C	2.969927	2.725256	-1.138126
H	3.094998	1.361905	-2.864531
H	1.604709	0.170504	-1.428302
H	1.265178	4.013955	-0.783651
H	3.576347	3.592002	-1.412559
C	-4.336544	-2.162687	-1.040434
C	-2.985031	-1.479828	-0.828517
N	-3.287223	-0.430770	0.153307
C	-4.645414	-0.529130	0.654551
C	-5.050059	-1.952255	0.292461
H	-4.893720	-1.661950	-1.840779
H	-2.226767	-2.172374	-0.451920
H	-4.675586	-0.334160	1.732739
H	-6.134223	-2.077404	0.234433
H	-2.590309	-1.040273	-1.750782
H	-4.228954	-3.215100	-1.314571
H	-4.664518	-2.650621	1.043386
H	-5.311164	0.202420	0.165658
H	1.908676	3.298830	0.707506
H	3.589946	2.042646	-0.545377
H	1.966313	2.713888	-3.048394
H	0.386578	1.044047	-2.370531

54
4pzMePyr2-ts-B-n ; E = -1106.20126235 Hartree

C	-2.616508	0.413022	0.277167
C	-3.351051	1.448939	0.879973
C	-1.208969	0.629694	0.006697
H	-4.382203	1.289215	1.171750
C	-2.772891	2.695939	1.058740
C	-0.691628	1.976474	0.077154
C	-1.478885	2.987547	0.634847
H	-1.093141	3.994438	0.739003
N	-0.426741	-0.412916	-0.185258
N	0.305216	-1.424709	-0.252493
C	1.614837	-1.308243	0.262810
C	2.654465	-2.123211	-0.169466
C	2.148197	-0.566059	1.356035
N	3.421329	-0.889346	1.552000
N	3.711675	-1.819867	0.617374
C	5.050228	-2.338593	0.528955
H	5.040381	-3.431867	0.495738
H	5.569346	-1.962598	-0.359720
H	5.581405	-2.008804	1.421025
C	2.674782	-3.122006	-1.266515
H	3.064336	-4.089668	-0.931252
H	1.650083	-3.262513	-1.617109
H	3.286593	-2.794728	-2.115581
C	1.454658	0.365265	2.289302
H	1.150517	1.288154	1.791240
H	0.546769	-0.095748	2.690858
H	2.122599	0.606068	3.119186
H	-3.367293	3.488725	1.505138
C	2.123393	2.502423	-2.146742
C	1.169350	1.488063	-1.517670
N	0.569326	2.221600	-0.396515
C	1.190819	3.524116	-0.226504
C	2.516363	3.377955	-0.959719
H	2.974736	2.021664	-2.635353
H	1.715011	0.606239	-1.170929
H	0.583014	4.322855	-0.683497
H	2.945612	4.341221	-1.246401
C	-3.945607	-2.594170	-1.265104
C	-2.713457	-1.732294	-0.984280
N	-3.174328	-0.810363	0.062023
C	-4.517810	-1.126381	0.507674
C	-4.702979	-2.569923	0.059935
H	-4.559596	-2.134895	-2.048583
H	-1.862707	-2.328323	-0.642192
H	-4.606274	-0.996290	1.592360
H	-5.755748	-2.849043	-0.031518
H	-2.384915	-1.176963	-1.869893
H	-3.679291	-3.601324	-1.595802
H	-4.230151	-3.246211	0.780583
H	-5.269993	-0.473079	0.032171
H	1.305538	3.761045	0.837876
H	3.237803	2.852614	-0.324027
H	1.599858	3.107127	-2.896282
H	0.388535	1.150546	-2.208449

54

4pzMePyr2-ts-H-m ; E = -1106.19254424 Hartree

C	2.596031	0.562717	0.361256
C	3.034277	1.703548	1.071698

C	1.227096	0.521539	-0.035561
H	4.037545	1.736767	1.475144
C	2.177428	2.760555	1.316463
C	0.412488	1.684558	0.081588
C	0.883309	2.782558	0.815839
H	0.267091	3.661412	0.955426
N	0.722931	-0.734895	-0.494372
N	-0.440700	-1.007378	-0.132124
C	-1.682211	-1.322059	0.146465
C	-2.519651	-0.729420	1.127488
C	-2.521778	-2.322304	-0.468503
N	-3.723871	-2.327222	0.067190
N	-3.690225	-1.377447	1.047936
C	-4.837506	-1.232163	1.904940
H	-5.112396	-0.178727	2.006469
H	-5.657052	-1.779259	1.440156
H	-4.644556	-1.646541	2.900367
C	-2.201179	0.307061	2.133586
H	-2.187773	-0.099810	3.151863
H	-1.211769	0.712706	1.911364
H	-2.930519	1.125076	2.115873
C	-2.147257	-3.201249	-1.604526
H	-1.127552	-3.572953	-1.467791
H	-2.839057	-4.041784	-1.684823
H	-2.163013	-2.650941	-2.552293
H	2.541134	3.611610	1.885939
C	-2.067494	2.110293	-2.523149
C	-1.037338	1.182448	-1.880754
N	-0.824399	1.746068	-0.543115
C	-1.684614	2.897776	-0.337006
C	-2.822274	2.660974	-1.316682
H	-1.568973	2.931544	-3.050853
H	-0.091257	1.138821	-2.432695
H	-2.008232	2.958435	0.708839
H	-3.508453	1.904063	-0.919587
C	4.552234	-2.510387	-0.238651
C	3.251363	-1.751281	-0.474603
N	3.494186	-0.454697	0.153318
C	4.909784	-0.255777	0.434782
C	5.602910	-1.408500	-0.282307
H	4.537504	-2.976866	0.753235
H	3.034674	-1.639078	-1.545613
H	5.249494	0.719638	0.065957
H	6.551210	-1.681725	0.187748
H	2.383461	-2.243140	-0.035358
H	4.712481	-3.298226	-0.979275
H	5.809127	-1.128361	-1.321467
H	5.108432	-0.283678	1.518310
H	-1.173096	3.845076	-0.581329
H	-3.391205	3.569493	-1.530105
H	-2.706219	1.587566	-3.239742
H	-1.430191	0.162549	-1.821763

54

4pzMePyr2-ts-H-n ; E = -1106.19088792 Hartree

C	2.741646	0.541301	0.037642
C	3.370589	1.760343	0.374619
C	1.327021	0.544729	-0.124761
H	4.437053	1.786849	0.555594

C	2.636006	2.921458	0.520582
C	0.601556	1.766766	-0.060753
C	1.265664	2.944166	0.295123
H	0.724106	3.880291	0.351340
N	0.719100	-0.713374	-0.420978
N	-0.421481	-0.894666	0.050945
C	-1.664813	-1.103915	0.399101
C	-2.610407	-1.946892	-0.236966
C	-2.418340	-0.463697	1.451439
N	-3.673283	-0.867840	1.443540
N	-3.774252	-1.732121	0.392827
C	-5.075674	-2.232931	0.036624
H	-5.030100	-3.304205	-0.175753
H	-5.732574	-2.057870	0.888019
H	-5.475449	-1.712200	-0.840541
C	-2.422930	-2.810044	-1.423447
H	-2.907838	-2.402229	-2.318920
H	-1.350591	-2.883310	-1.622748
H	-2.819961	-3.817938	-1.261510
C	-1.871853	0.451548	2.483874
H	-1.558967	-0.099926	3.377510
H	-0.994317	0.962028	2.078939
H	-2.621975	1.184614	2.790270
H	3.148163	3.840018	0.793820
C	-2.709300	1.610865	-1.695654
C	-1.192489	1.380067	-1.709602
N	-0.763359	1.799981	-0.378595
C	-1.541313	2.984986	-0.079413
C	-2.947302	2.581992	-0.519540
H	-3.049210	2.022784	-2.649969
H	-0.710063	2.008926	-2.478123
H	-1.473063	3.231880	0.984603
H	-3.457314	2.063133	0.296102
C	4.359512	-2.751102	0.274199
C	3.085924	-1.980451	-0.062446
N	3.522102	-0.583876	-0.081365
C	4.972820	-0.482683	-0.038209
C	5.449631	-1.893073	-0.356558
H	4.499856	-2.793354	1.360627
H	2.677060	-2.281202	-1.033137
H	5.335485	0.258906	-0.760135
H	6.451049	-2.092289	0.033584
H	2.293405	-2.131612	0.673820
H	4.334804	-3.776877	-0.102946
H	5.469862	-2.045375	-1.441551
H	5.326116	-0.171287	0.959346
H	-1.200019	3.866567	-0.655059
H	-3.551051	3.449817	-0.798266
H	-3.244098	0.671770	-1.531252
H	-0.917844	0.344764	-1.913406

References

1. Calbo, J.; Weston, C. E.; White, A. J. P.; Rzepa, H. S.; Contreras-García, J.; Fuchter, M. J. *J. Am. Chem. Soc.* **2017**, *139*, 1261–1274. doi:10.1021/jacs.6b11626.
2. Stricker, L.; Fritz, E.-C.; Peterlechner, M.; Doltsinis, N. L.; Ravoo, B. J. *J. Am. Chem. Soc.* **2016**, *138*, 4547–4554. doi:10.1021/jacs.6b00484.
3. Devi, S.; Saraswat, M.; Grewal, S.; Venkataramani, S. *J. Org. Chem.* **2018**, *83*, 4307–4322. doi:10.1021/acs.joc.7b02604.
4. SHELXTL v5.1, Bruker AXS, Madison, WI, 1998.
5. Sheldrick, G. M.; IUCr. *Acta Crystallogr. Sect. C Struct. Chem.* **2015**, *71*, 3–8. doi:10.1107/S2053229614024218.
